# Immunomodulatory Effects of Endoscopic Ultrasound-Guided Thermal Ablation in Patients with Pancreatic Ductal Adenocarcinoma

**DOI:** 10.3390/cancers15143704

**Published:** 2023-07-21

**Authors:** Sabrina Gloria Giulia Testoni, Claudia Minici, Elisa Benetti, Francesca Clemente, Daniela Boselli, Clara Sciorati, Lucia De Monte, Maria Chiara Petrone, Markus Enderle, Walter Linzenbold, Maria Pia Protti, Angelo Manfredi, Francesco De Cobelli, Michele Reni, Massimo Falconi, Gabriele Capurso, Paolo Giorgio Arcidiacono, Emanuel Della-Torre

**Affiliations:** 1 Pancreatico-Biliary Endoscopy and Endosonography Division, Pancreas Translational & Clinical Research Center, IRCCS San Raffaele Scientific Institute, Vita-Salute San Raffaele University, Via Olgettina 60, 20132 Milan, Italy; 2 IRCCS San Raffaele Scientific Institute, Vita-Salute San Raffaele University, Via Olgettina 60, 20132 Milan, Italy; 3 Center for Omics Sciences, IRCCS San Raffaele Scientific Institute, Via Olgettina 60, 20132 Milan, Italy; 4 San Raffaele Telethon Institute for Gene Therapy (SR-Tiget) and Pediatric Immunohematology and Bone Marrow Transplantation Unit, IRCCS San Raffaele Scientific Institute, Via Olgettina 60, 20132 Milan, Italy; 5 Tumor Immunology Unit, Division of Immunology, Transplantation, and Infectious Disease, Pancreas Translational & Clinical Research Center, IRCCS San Raffaele Scientific Institute, Vita-Salute San Raffaele University, Via Olgettina 60, 20132 Milan, Italy; 6 FRACTAL (Flow Cytometry Resource, Advanced Cytometry Technical Applications Laboratory), IRCCS San Raffaele Scientific Institute, Via Olgettina 60, 20132 Milan, Italy; 7 Division of Immunology, Transplantation & Infectious Diseases, IRCCS San Raffaele Scientific Institute, Vita-Salute San Raffaele University, Via Olgettina 60, 20132 Milan, Italy; 8 Department of Research and Basic Technologies, Erbe Elektromedizin GmbH, Waldhörnlestraße 17, 72072 Tübingen, Germany; 9 Unit of Immunology, Rheumatology, Allergy and Rare Diseases, Pancreas Translational & Clinical Research Center, IRCCS San Raffaele Scientific Institute, Vita-Salute San Raffaele University, Via Olgettina 60, 20132 Milan, Italydellatorre.emanuel@hsr.it (E.D.-T.); 10 Department of Radiology, Experimental Imaging Center, IRCCS San Raffaele Scientific Institute, Vita-Salute San Raffaele University, Via Olgettina 60, 20132 Milan, Italy; 11 Department of Medical Oncology, Pancreas Translational & Clinical Research Center, IRCCS San Raffaele Scientific Institute, Vita-Salute San Raffaele University, Via Olgettina 60, 20132 Milan, Italy; 12 Division of Pancreatic Surgery, Pancreas Translational & Clinical Research Center, IRCCS San Raffaele Scientific Institute, Vita-Salute San Raffaele University, Via Olgettina 60, 20132 Milan, Italy

**Keywords:** pancreatic cancer, ablation techniques, immunomodulation, T Lymphocytes, B Lymphocytes, monocytes, cytokines

## Abstract

**Simple Summary:**

Thermal ablation under endoscopic ultrasound (EUS)-guidance has been investigated in pancreatic ductal adenocarcinoma (PDAC) based on its potential to boost local and systemic anti-tumor immune response. In a recent phase II randomized controlled trial, ablation of borderline resectable (BR) and locally advanced (LA) PDAC using the HybridTherm Probe (HTP) under EUS-guidance in combination with chemotherapy was shown to ameliorate disease progression at 6 months compared to chemotherapy alone. In this work, we aimed to explore the effects of EUS-ablation with HTP on the systemic immune response in patients with BR and LA PDAC. In contrast to chemotherapy, EUS-HTP selectively affected immunological predictors of poor outcome such as serum levels of APRIL/TNFSF13 and inflammatory monocytes, reinforcing its potential use in selected PDAC patients.

**Abstract:**

Immunological consequences of endoscopic ultrasound (EUS)-local thermal ablation (LTA) for pancreatic ductal adenocarcinoma (PDAC) have not been extensively assessed. We aimed to explore EUS-LTA effects on the systemic immune response in PDAC. Peripheral blood was collected from 10 treatment-naïve patients with borderline resectable and locally advanced PDAC, randomly allocated to Nab-paclitaxel plus Gemcitabine chemotherapy (CT-arm, n = 5) or EUS-LTA with HybridTherm Probe plus CT (HTP + CT-arm, n = 5). Twenty healthy donors were included as controls. Flow-cytometry and multiplex assays were used to profile immune cell subsets and measure serum cytokines/chemokines, respectively. At baseline, PDAC patients showed increased circulating monocytes and lower circulating lymphocytes and CD19+ B cells counts compared to healthy controls. After 4 months, CT induced decrease of B regulatory cells, CD4+ cytotoxic T cells and IL-1β. The addition of EUS-HTP to CT selectively decreased the serum levels of APRIL/TNFSF13 as well as T regulatory cells, total, classic and inflammatory monocytes. Serum levels of APRIL/TNFSF13 and total, classic and inflammatory monocytes counts at baseline were associated with worse overall survival. EUS-HTP has the potential to selectively impact on immune cells and cytokines associated with poor outcomes in PDAC.

## 1. Introduction

Pancreatic ductal adenocarcinoma (PDAC) has the shortest 5-year survival rate among solid cancers and is expected to become the second leading causes of cancer-related death in Western Countries by 2030 [1,2].

A multidisciplinary approach that includes surgery represents the only potentially curative treatment for PDAC, but it can be offered to less than 20% of patients with resectable lesions [3]. All other patients, including those with metastatic, borderline resectable (BR), and locally advanced (LA) PDAC, are initially treated with chemotherapy (CT) [4,5,6]. Yet, despite a number of possible combined polychemotherapy regimens, the prognosis of patients with PDAC remains dismal and, as opposed to other solid tumors, has only marginally improved with targeted therapies such as monoclonal antibodies, small molecules, and immune checkpoint inhibitors [3,7,8,9,10,11].

Failure of PDAC to respond to available systemic therapies has been largely attributed to its characteristic desmoplastic stromal reaction that acts as a physical barrier to drug delivery and provides an immunosuppressive microenvironment [11]. To increase intra-tumor drug concentrations and to boost anti-tumor immune responses, thermo-ablative therapy has been proposed as an alternative and minimally invasive approach [12]. Indeed, local thermo-ablation (LTA) has already been successfully used in other solid cancers, such as renal, breast, head, neck, and liver lesions. In these settings, LTA has been shown to control tumor spreading by inducing necrosis of neoplastic cells and by increasing their radio/chemosensitivity [13,14]. In addition, as a result of immunogenic cell death of malignant cells, cytokines release, and exposure of tumor-derived antigens, LTA has been postulated to act as an “in situ cancer vaccine” thus amplifying anti-tumor immune responses [12,15,16,17,18].

Based on these premises, LTA therapy has been optimized in preclinical models of PDAC and recently assessed in clinical trials [19,20,21]. In preclinical models, the sequential combination of local cooling and heating of PDAC lesion (“cryo-thermal ablation”) induces the release of damage-associated molecular patterns (DAMPs) and polarization of anti-tumor macrophages. Moreover, cryo-thermal ablation generated a long-lasting neoantigen-specific CD4+ T cell response that protects against tumor re-challenge [22,23,24].

In a recent phase II randomized controlled trial (RCT), a carbon dioxide (CO_2_)-cooled bipolar radiofrequency (RF)-energy HybridTherm Probe (HTP) device was used under endoscopic ultrasound (EUS)-guidance to treat patients with BR and LA PDAC in combination with CT [25]. The rate of patients free from disease progression and that of patients with a ≥50% decrease of the carbohydrate antigen 19.9 (CA19.9) at 6-month follow-up was 11.2% and 10.7% higher in the EUS-HTP plus CT treated arm than in the arm treated with CT alone, respectively [25]. Moreover, the tumor volume reduction was 21.6% higher compared to CT alone, suggesting a better local control of tumor burden when EUS-HTP was added to CT [25]. Yet, the immunological consequences of local thermal ablation in patients with PDAC have not been extensively assessed.

In the present work, we aimed to explore the effects of EUS-guided ablation with HTP on the systemic immune response in patients with PDAC and to correlate these effects with clinical outcomes.

## 2. Materials and Methods

### 2.1. Patients and Study Design

This study was conducted at San Raffaele Hospital (Milan, Italy) as an explorative study in the context of a multicenter randomized controlled trial (RCT) of EUS-HTP in addition to CT for the treatment of BR and LA PDAC (protocol N. HTP2014; ClinicalTrials.gov ID NCT02336672).

This RCT included patients fulfilling the inclusion and exclusion criteria outlined elsewhere [25]. Specifically, eligible patients were treatment-naïve and received a cytological or histological diagnosis of locally advanced (LA) or borderline resectable (BR) PDAC. Disease staging was performed by contrast-enhanced (CE) total-body multidetector computed-tomography (MDCT) scan, abdominal double-weighed magnetic resonance imaging (DW-MRI), and EUS. For the current exploratory study, patients with underlying autoimmune disorders, concomitant solid or hematologic tumors, and/or on immunosuppressive/corticosteroid treatment were excluded.

Eligible patients were randomly allocated (1:1) within 3 weeks from diagnosis to receive upfront EUS-HTP plus CT (HTP-CT arm) or CT alone (CT arm). Randomization was performed using a computer-generated random list generator held by ERBE (Elektromedizin GmbH, Tübingen, Germany), responsible also for monitoring data accuracy. Investigators and patients were not masked to the treatment allocation due to specific technical equipment required for the additional treatment performed in the HTP-CT arm and not in the CT arm.

Patients allocated to the HTP-CT arm were treated with EUS-HTP followed by CT within one week. Three monthly EUS-HTP sessions were planned (time-points 0, 1 month, and 2 months). Patients allocated to the CT arm started chemotherapy after oncological evaluation. In both treatment arms, Nab-paclitaxel (125 mg/m^2^ on days 1, 8, and 15) plus Gemcitabine (1000 mg/m^2^ on days 1, 8, and 15) was administered for a minimum of 6 cycles according to the Medical Oncology Italian Association (AIOM) guidelines.

Radiological response at 4 and 6 months was defined according to the Choi criteria as complete response, partial response, or stable disease [26,27]. Based on disease response and/or evidence of progression, patients were candidate to surgical resection or to further CT cycles as outlined in Figure S1. All patients were followed up for a minimum of 6 months after therapy or until death.

Serum samples and peripheral blood mononuclear cells (PBMCs) were collected before EUS-HTP and/or CT (baseline) and after 4 months (namely, 30 days after the third EUS-HTP session in the HTP-CT arm). Serum samples and PBMCs from 20 age- and sex-matched healthy donors (HDs) were collected as controls.

The study was approved by the local Ethics Committee of San Raffaele Hospital (Milan, Italy) and conducted after written informed consent in accordance with the Declaration of Helsinki.

### 2.2. Endoscopic Ultrasound-Guided HybridTherm Probe Ablation

The EUS-HTP ablation procedures were performed by two expert endosonographers (>400/year EUS-guided biopsy). Pancreatic lesions were treated under real-time EUS visualization using a needle-shaped (14-gauge) HTP device with a 26-mm length active tip as previously described [28,29,30]. Ablation parameters were set as follows: fixed RF power of 18 W (VIO 300D RF-surgery system, ERBE Elektromedizin GmbH) and cooling CO_2_ flow of 9 L/min (ERBECRYO2 system, ERBE Elektromedizin GmbH), with application time varying between 240 s and 480 s for a 2-cm up to >3-cm mass, respectively, or until the rise of the electric resistance induced by tumor tissue desiccation and devitalization or the predefined ablation time has elapsed.

### 2.3. Flow-Cytometry

PBMCs were freshly isolated from patients with PDAC and from HDs by using Ficoll-Hypaque centrifugation on the same day of the blood draw and subsequently frozen in liquid nitrogen, as described in standard protocols (see Supplementary Material).

Five panels of fluorochrome-conjugated anti-human monoclonal antibodies (mAbs) were designed for immunophenotyping different cell subsets of monocytes, T cytotoxic lymphocytes, T regulatory cells, T helper lymphocytes, T follicular helper lymphocytes, B lymphocytes, and B regulatory cells. CD4+ SLAMF7+ cytotoxic T cells were also analyzed [31,32]. Fluorochrome-conjugated anti-human mAbs were purchased from BD Biosciences-Pharmingen (San José, CA, USA) and BioLegend (San Diego, CA, USA). Details on flow-cytometry panels and gating strategies are reported in Table S1 and Figure S2.

PBMCs were live/dead single stained with Viakrome 808 fixable viability dye (Beckman Coulter, 2.5 µL for 5 × 10^6^ cells), then stained with titled surface mAbs, and fixed, according to the manufacturer’s instructions (see Supplementary Material). For intracellular staining, after surface staining, cells were first permeabilized, and then stained with intracellular mAbs. All stained PBMCs were then resuspended and stored at 4 °C in dark room and acquired using the CytoFLEX LX Flow Cytometer (Beckman Coulter, Brea, CA, USA). Results from the flow-cytometric acquisition were analyzed using the FCS Express 7 Flow Research software (De Novo Software, Los Angeles, CA, USA).

### 2.4. Multiplex Immunoassays

Serum samples obtained from PDAC patients and HDs were studied using three different Luminex multiplex assays from Bio-Rad Laboratories Inc. (Hercules, CA, USA): the Pro Human Inflammation Panel I Assay 37-BioPlex, the Pro Human Cytokine Immunoassay 27-BioPlex, and the Pro TGF-β Immunoassay 3-BioPlex (Table S2). Measurements were performed in duplicate and in a single experimental session for each multiplex assay, according to manufacturer instructions (see Supplementary Material). Magnetic separation was performed using the BioPlex Pro Wash Station (Bio-Rad Laboratories). Bead fluorescence readings were taken using the BioPlex Manager version 6.1.0.727 (Bio-Rad Laboratories) with Low PMT (Low RP1) setting on the BioPlex 200 System (Bio-Rad Laboratories). A calibration of signal output and for IQ/OQ of the reader was performed before each analysis using the Bio-Plex Calibration Kit (Bio-Rad Laboratories).

### 2.5. Study Endpoints

The primary endpoint of this study was to explore the systemic immunomodulatory effects of EUS-HTP ablation in patients with BR and LA PDAC. 

Secondary endpoints were (i) to compare the immunomodulatory effects induced by EUS-HTP added to CT with those induced by CT alone, and (ii) to correlate these effects with the patients’ PFS and OS.

### 2.6. Statistical Analysis

Continuous variables were expressed as mean (standard deviation; SD) or median (range). Categorical variables were expressed as numbers and percentages. Comparisons of the survival times were performed using the Log-rank Mantel–Cox test, with determination of the hazard ratio (HR), according to Mantel–Haenszel method, and 95% confidence interval (CI) of ratio. Survival curves for progression-free survival (PFS) and overall survival (OS) were plotted using the Kaplan–Meier method. PFS and OS were evaluated as the time interval from the randomization to the first radiological evidence of disease progression (or death if occurred earlier) and to the death (or last follow-up assessment), respectively. Inter-group and intra-group differences were determined using unpaired (Welch two sample *t*-test) and paired (paired *t*-test) analyses, respectively. Specifically, the *t*-test was used for continuous variables and the Chi-squared test was used for categorical variables. Correlations between the baseline immunological parameters and PFS and OS were performed using the Spearman’s rho rank correlation test and are represented by scatter plots. A *p*-value < 0.05 was considered statistically significant. Statistical analysis was performed using the R (version 4.1.3) and R-studio (version 2022.2.1.461) software.

According to the RCT within which the immunological exploratory analysis was performed, data analysis was planned both as Intention-To-Treat (ITT–set) including all randomized patients meeting eligibility criteria, and as Per-Protocol (PP-set) including patients who did not violate the protocol and did not have missing data. Dropout patients were considered as excluded from both the ITT-set and PP-set analysis.

## 3. Results

### 3.1. Patients and Clinical Outcomes

Ten consecutive patients enrolled in the RCT were included in the present explorative study [24]. Five patients were treated with upfront EUS-HTP and Nab-paclitaxel plus Gemcitabine (HTP-CT arm): four of them received six cycles of CT and one patient received five cycles of CT. The other five patients (CT arm) were treated with Nab-paclitaxel plus Gemcitabine alone: two of them received four cycles of CT and three patients received six cycles of CT. Patients in the two treatment arms had similar epidemiological, clinical, and oncological features, yet with a large variability in tumor size and CA19.9 serum levels (Table 1).

One patient of the HTP-CT arm with LA PDAC was resected after induction CT and EUS-HTP, with R0 resection. Three patients of the CT arm (one with BR PDAC and two with LA PDAC) underwent surgical resection (*p* = 0.22 vs. HTP-CT arm), despite the best response was stable disease in all of them, with R0 resection in all patients but one with LA PDAC. The resected patient of the HTP-CT arm relapsed after a median of 16.5 months and survived for a median of 36 months. In the CT arm, relapse occurred after median of 14.4 months; among these patients, one with LA PDAC deceased after 26 months and the other two (one with BR PDAC and one with LA PDAC) were still alive after a median of 30 months. The other patients who did not undergo surgery progressed through CT and moved on to second line therapy. One patient of the CT arm did not undergo the 4-month follow-up due to progression-related death.

No statistically significant difference was observed between the two treatment arms in terms of mean PFS (11.45 months, 95% CI: 4.59 to 18.31, in the HTP-CT arm vs. 10.56 months, 95% CI: 7.11 to 14.02, in the CT arm; *p* = 0.79) and OS (19.88 months, 95% CI: 10 to 29.76, in the HTP-CT arm vs. 23.22 months, 95% CI: 12.61 to 33.83, in the CT arm; *p* = 0.85) (Figure S3A,B).

### 3.2. Immunological Landscape of PDAC Patients at Baseline Compared to Healthy Donors

**Figure 1:**
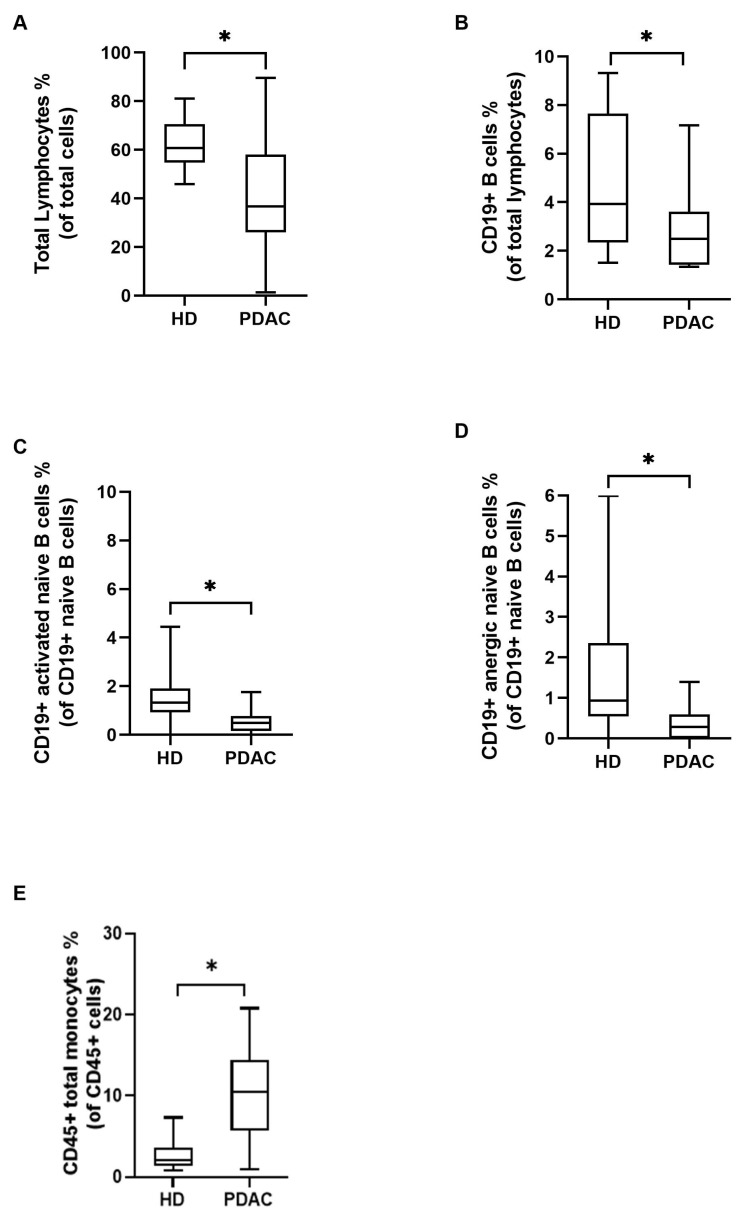
Immunological variables among patients with pancreatic adenocarcinoma (PDAC) and sex- and age-matched healthy donors (HD) at baseline. The mean percentage of circulating total lymphocytes (**A**), CD19+ B cells (**B**), activated naïve B cells (**C**), and anergic naïve B cells (**D**) was significantly lower in PDAC patients than in HD. The mean percentage of circulating total CD45+ monocytes was significantly higher in PDAC patients than in HD (**E**). Inter-group variables were compared using the Welch two sample *t*-test: significant (*) = *p* < 0.05.

In order to define the baseline immunological landscape of PDAC patients before treatment, PDAC patients were compared to 20 HDs. PDAC patients showed statistically significantly lower lymphocytes counts compared to HDs (*p* = 0.027) (Figure 1A). In particular, PDAC patients exhibited significantly lower percentage of circulating CD19+ B cells, activated and anergic naïve B cells (*p* = 0.03, *p* = 0.002 and *p* = 0.004, respectively) (Figure 1B–D). On the other hand, the percentage of total monocytes was significantly increased in PDAC patients (*p* = 0.004) (Figure 1E).

Circulating T cell subsets and serum inflammatory cytokines and chemokines were not significantly different between PDAC patients and HDs (Figure S4A–F).

### 3.3. Correlation of Immunological Variables with Clinical Outcomes

**Figure 2:**
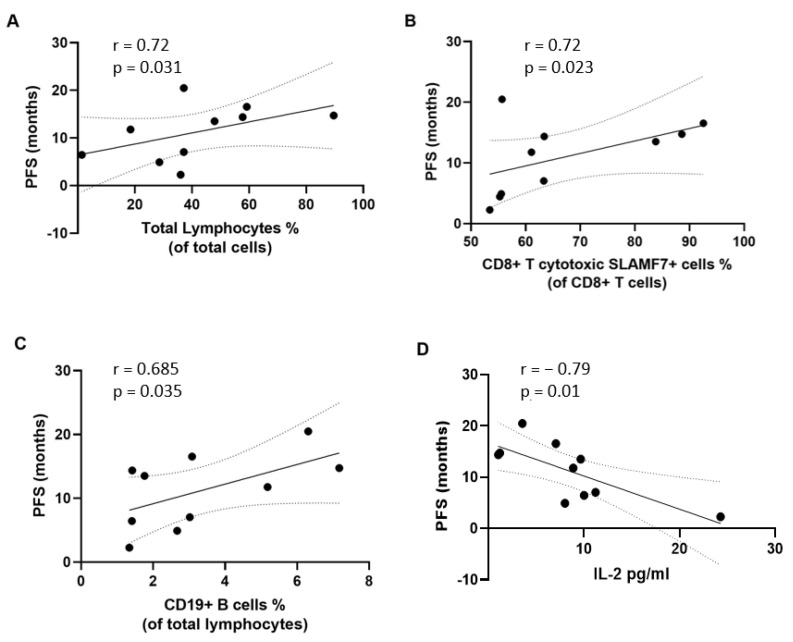
Correlation between immunological variables at baseline and progression-free survival (PFS). The percentage of circulating total lymphocytes (**A**), CD8+ cytotoxic T cells (**B**), and CD19+ B cells (**C**) significantly positively correlated with PFS. The serum concentration of interleukin (IL)-2 (**D**) showed a significant negative correlation with PFS. Correlation studies were performed using the Spearman’s rho rank correlation test.

As shown in Figure 2A–C, the percentage of total lymphocytes, effector memory CD8+ cytotoxic T cells, and CD19+ B cells at baseline positively correlated with the PFS (r = 0.72, *p* = 0.031; r = 0.72, *p* = 0.023; and r = 0.68, *p* = 0.035, respectively) (Figure S5A–F).

**Figure 3:**
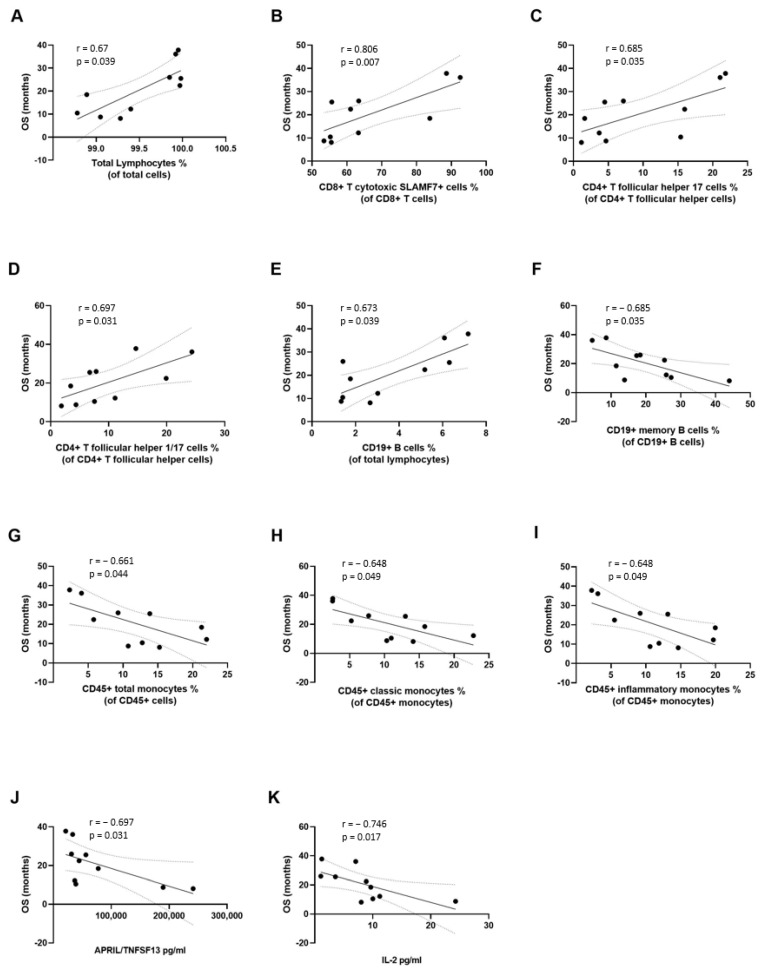
Correlation between immunological variables at baseline and overall survival (OS). The percentage of circulating total lymphocytes (**A**), CD8+ T cytotoxic cells (**B**), CD4+ follicular T helper 17 cells (**C**), CD4+ follicular T helper 1/17 cells (**D**), CD19+ B cells (**E**) showed significant positive correlation with OS. The percentage of circulating CD19+ memory B cells (**F**), CD45+ total monocytes (**G**), classic monocytes (**H**), inflammatory monocytes (**I**), and the serum concentration of APRIL/TNFSF13 (**J**), and interleukin (IL)-2 (**K**), showed a significant negative correlation with OS. Correlation studies were performed using the Spearman’s rho rank correlation test.

Similarly, the percentage of total lymphocytes, effector memory CD8+ cytotoxic T cells, CD4+ T follicular helper 17 cells and 1/17 cells at baseline positively correlated with OS (r = 0.67, *p* = 0.039; r = 0.81, *p* = 0.007; r = 0.68, *p* = 0.035; and r = 0.7, *p* = 0.031, respectively) (Figure 3A–D and Figure S6A–F). CD19+ B cells also positively correlated with OS (r = 0.67, *p* = 0.039) (Figure 3E). Conversely, a negative correlation was found between the percentage of circulating memory B cells, CD45+ total monocytes, classic monocytes, and inflammatory monocytes and OS (r = −0.68, *p* = 0.035; r = −0.66, *p* = 0.044; r = −0.65, *p* = 0.049; and r = −0.65, *p* = 0.049, respectively) (Figure 3F–I). Finally, as shown in Figure 3J, baseline serum levels of APRIL/TNFSF13 negatively correlated with OS (r = −0.7, *p* = 0.03) whereas serum levels of IL-2 negatively correlated with both OS and PFS (r = −0.75, *p* = 0.017 and r = −0.79, *p* = 0.01, respectively) (Figure 3K and Figure 2D, respectively).

**Figure 4:**
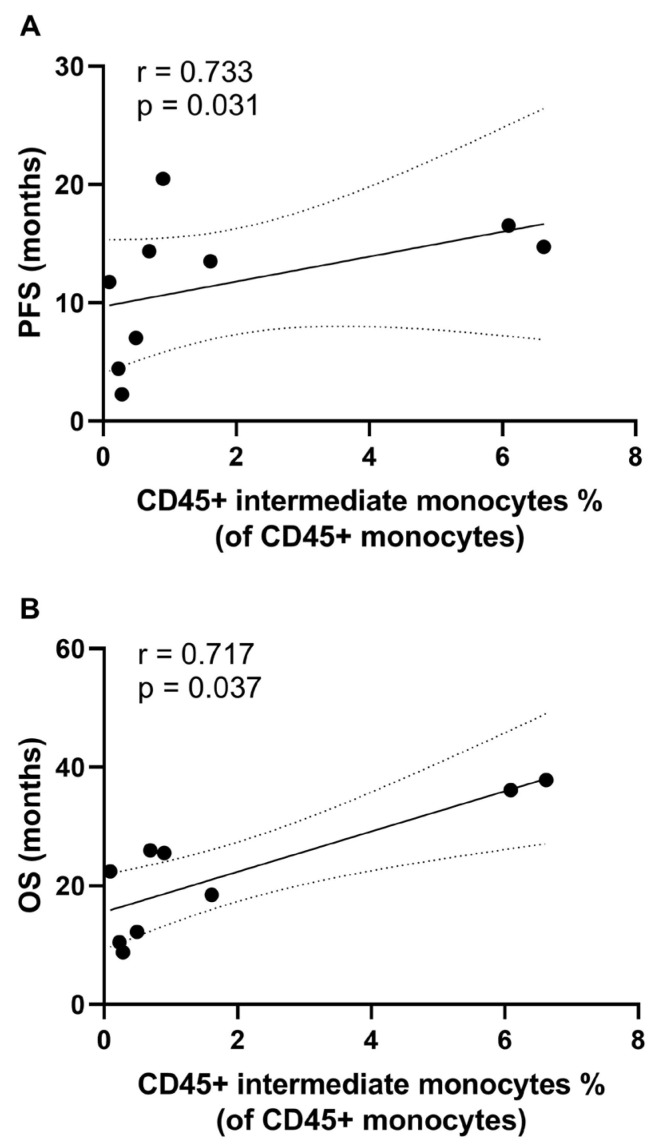
Correlation between immunological variables at 4-month follow-up and progression-free survival (PFS) and overall survival (OS). The percentage of circulating CD45+ intermediate monocytes showed significant positive correlation with PFS (**A**) and OS (**B**). Correlation studies were performed using the Spearman’s rho rank correlation test.

We then performed correlation studies between the immunological variables at 4-month follow-up and PFS (Figure S7A–F) and OS (Figure S8A–F). When considering all PDAC patients included in this study (n = 10), a statistically significant positive correlation was found between the percentage of circulating intermediate monocytes at 4-month follow-up and PFS (r = 0.73, *p* = 0.031) and OS (r = 0.72, *p* = 0.037) (Figure 4A,B).

No significant correlation was observed when considering the HTP-CT arm (Figure S9A–L) and CT arm (Figure S10A–L) groups separately.

### 3.4. Effects of EUS-Guided Thermal Ablation on Circulating Immune Cells and Inflammatory Markers

At baseline, patients in the HTP-CT arm and in the CT arm were similar with regard to the mean percentage of circulating B and T cell subsets, monocytes, and cytokines (Figure S11A–F).

**Figure 5:**
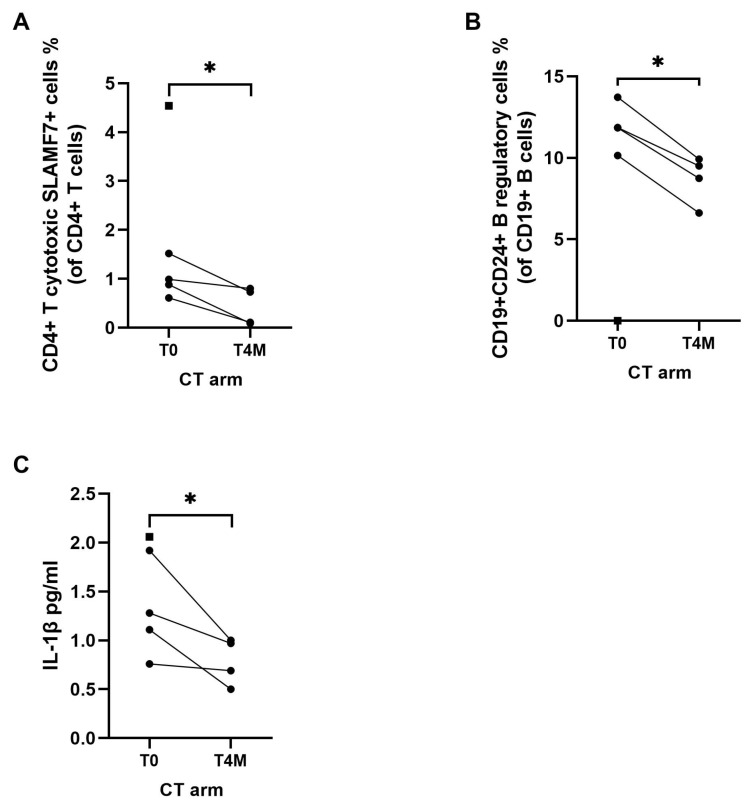
Effects of chemotherapy (CT arm) on immune cells and cytokines after 4 months of therapy. The mean percentage of circulating CD4+ cytotoxic T lymphocytes (**A**) and CD24+ B regulatory cells (**B**) were significantly lower at 4 month (T4M) compared to baseline (T0). The mean serum concentration of interleukin (IL)-1β (**C**) was significantly lower at 4 months (T4M) compared to baseline (T0). Square dot represents the patient in the CT arm who did not reach 4-month follow-up. Intra-group variables were compared using the paired *t*-test: significant (*) = *p* < 0.05.

To assess the impact of EUS-HTP in addition to CT on circulating immune cells and on inflammatory markers, we first assessed the immunological effects of standard CT alone. After four months of treatment, Nab-paclitaxel plus gemcitabine induced a significant decrease of CD4+ cytotoxic T lymphocytes (*p* = 0.03) and of B regulatory cells (*p* = 0.002) (Figure 5A,B). A statistically significant decrease of the serum concentration of interleukin (IL)-1β compared to baseline was also observed (*p* = 0.04) (Figure 5C).

Monocytes subsets were unaffected by CT (Figure S12A–F). Of note, the percentage of B regulatory cells, CD4+ cytotoxic T lymphocytes, and IL-1β in the CT arm four months after treatment was not statistically different from that observed in the HTP-CT arm (Figure S13A–F).

**Figure 6:**
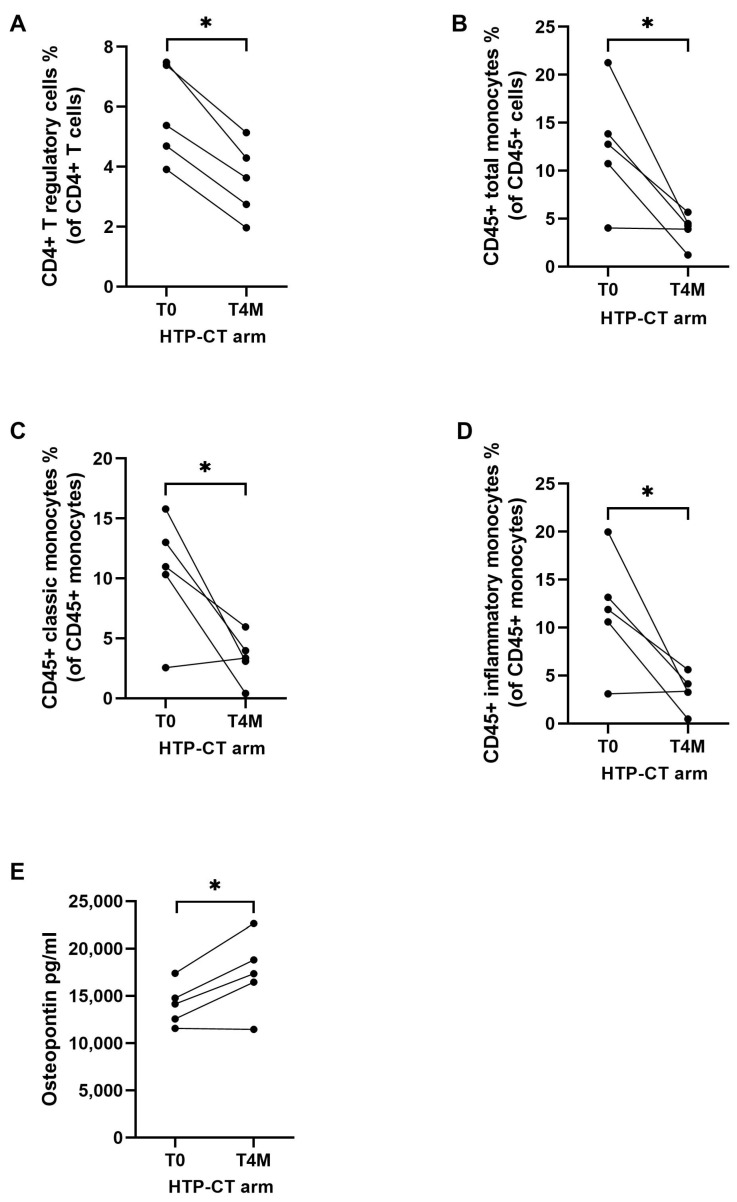
Effects of HybridTherm Probe ablation plus chemotherapy (HTP-CT arm) on immune cells and cytokines after 4 months of therapy. The mean percentage of circulating CD4+ T regulatory cells (**A**), CD45+ monocytes (**B**), CD45+ classic monocytes (**C**), and CD45+ inflammatory monocytes (**D**) were significantly lower at 4 months (T4M) compared to baseline (T0). The mean serum concentration of osteopontin was significantly higher at 4 months (T4M) compared to baseline (T0) (**E**). Intra-group variables were compared using the paired *t*-test: significant (*) = *p* < 0.05.

Addition of EUS-HTP to CT led to a selective significant decrease of the percentage of T regulatory cells, total monocytes, classic monocytes, and inflammatory monocytes (*p* = 0.007, *p* = 0.01, *p* = 0.01, and *p* = 0.02, respectively) compared with baseline (Figure 6A–D). Conversely, the mean serum levels of osteopontin significantly increased compared with baseline in patients treated with HTP plus CT (*p* = 0.002) (Figure 6E and Figure S14A–F).

**Figure 7:**
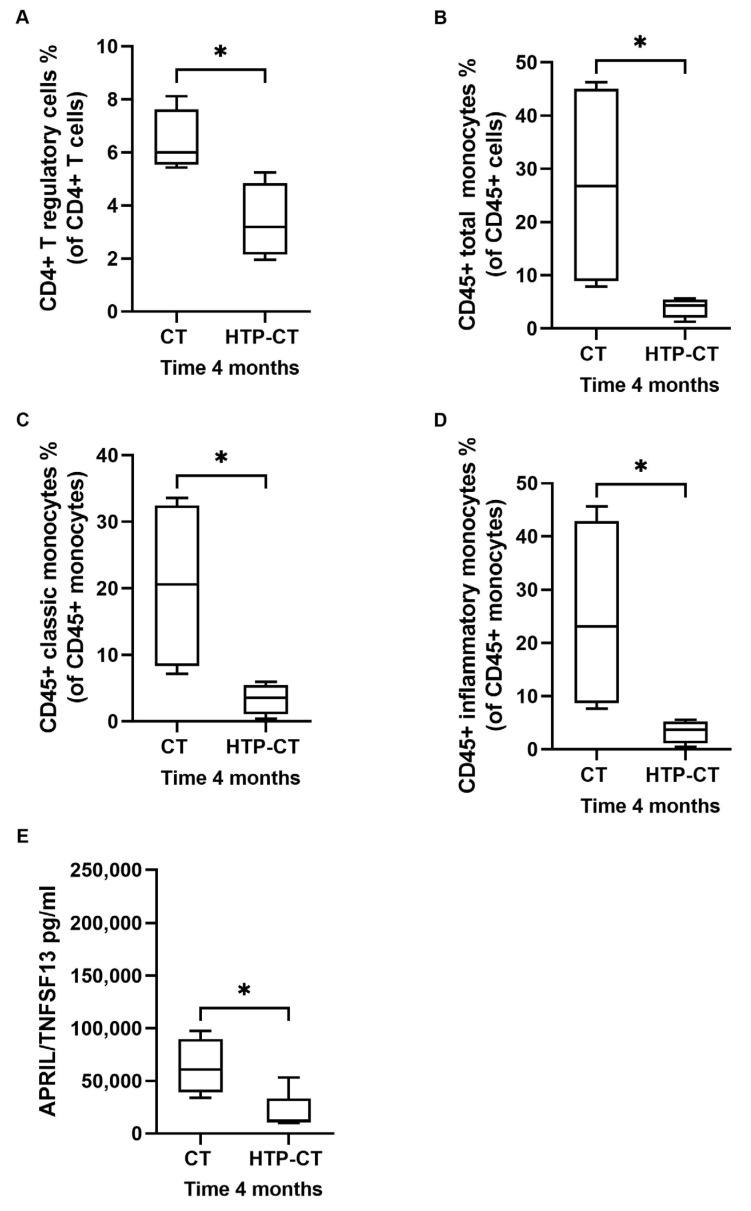
Effects of HybridTherm Probe ablation plus chemotherapy (HTP-CT arm) over chemotherapy alone (CT arm) on immune cells and cytokines after 4 months of therapy. The mean percentage of circulating CD4+ T regulatory cells (**A**), CD45+ monocytes (**B**), CD45+ classic monocytes (**C**), and CD45+ inflammatory monocytes (**D**) were significantly lower at 4 months in HTP-CT arm than CT arm. The mean concentration of soluble A proliferation-inducing ligand/tumor necrosis factor ligand superfamily member 13 (APRIL/TNFSF13) (**E**) was significantly lower at 4 months in HTP-CT arm than CT arm. Inter-group variables were compared using the Welch two sample *t*-test: significant (*) = *p* < 0.05.

Indeed, T regulatory cells, total monocytes, classic and inflammatory monocytes in the HTP-CT arm four months after treatment were significantly decreased compared to patients treated with CT alone (*p* = 0.02 for T regulatory cells and *p* = 0.03 for all monocytes comparisons) (Figure 7A–D). At four months, patients in the HTP-CT arm also showed significantly reduced serum levels of APRIL/TNFSF13 compared to patients in the CT arm (*p* = 0.04) (Figure 7E and Figure S13A–F).

We then correlated the immunological variables that significantly differed between the HTP-CT arm and the CT arm at four months with the tumor volume and serum levels of CA19-9 at four months, but no statistically significant correlation was observed (Figure S15A,B).

## 4. Discussion

Profound perturbations of the immune system occur in patients with PDAC leading to impaired mechanisms of immune surveillance [33,34,35]. Immune alterations characteristic of PDAC patients include expansion of T and B regulatory cells, increased production of immunosuppressive cytokines, differentiation of tumor promoting inflammatory macrophages, and impairment of anti-tumor cytotoxic CD8 cells, T helper 17 cells, and B lymphocytes [36,37,38,39,40,41,42,43].

Due to the poor penetration of available systemic chemotherapies into the neoplastic tissue, local reprogramming of this tumor promoting microenvironment and activation of effective anti-tumor immune responses represent highly relevant unmet needs in the treatment of PDAC [39,44].

In this regard, EUS-guided LTA is increasingly investigated as it offers the unprecedented opportunity to lyse neoplastic cells directly in the pancreatic gland, thus releasing tumor-specific antigens that may boost a T cell-mediated anti-tumor immune response [12,18,45,46]. In murine models of mammary cancer and melanoma, cryo-thermal therapy reshaped the immunosuppressive tumor microenvironment by decreasing T helper 2 cells, T regulatory cells, and myeloid-derived suppressive cells infiltrate. In addition, it promoted the differentiation of anti-tumor M1 macrophages and antigen-specific CD4+ T helper 1 cells [22,23,24]. In mouse models of PDAC, intra-tumor radiofrequency ablation (RFA) reduced tumor progression by increasing neutrophil and dendritic cell infiltrate and by promoting a significant CD4+ and CD8+ T cell abscopal response [44,47].

We recently completed a phase II RCT comparing EUS-HTP plus CT with CT alone for the treatment of BR and LA PDAC patients [25]. Addition of EUS-HTP to CT improved PFS at 6 months compared with CT alone (41.2% vs. 30%, respectively). Moreover, tumor volume reduction was achieved in 64% of patients in the HTP-CT arm compared to 47% of patients in the CT arm [25].

In the present work, we first compared the immunological landscape of PDAC patients with that of healthy controls. We then investigated the correlation between PDAC patients’ outcomes, in terms of PFS and OS, and their baseline immunological profile and identified inflammatory monocytes and APRIL/TNFSF13 as predictors of poor OS. Next, we observed the effects of local cryo-thermal ablation on the systemic immune response in patients with PDAC and demonstrated that local cryo-thermal ablation of pancreatic lesions modulates the immune system at the systemic level over and beyond the effect induced by CT.

In particular, CT decreased the level of immune cell subsets and cytokines known to be associated with worse PDAC prognosis such as B regulatory cells and IL-1β [48,49,50]. This effect was likely exclusively attributable to CT because the percentage of B regulatory cells and IL-1β in the CT arm four months after treatment was not statistically different from that observed in the HTP-CT arm.

On the other hand, addition of EUS-HTP to Nab-paclitaxel and Gemcitabine selectively decreased other immunological biomarkers associated with worse PDAC outcomes including T regulatory cells, inflammatory monocytes, and serum levels of APRIL/TNFSF13, suggesting a specific effect of thermal HTP ablation [51,52,53]. Indeed, the level of T regulatory cells, inflammatory monocytes, and APRIL/TNFSF13 in patients randomized to the HTP-CT arm after four months of treatment was significantly lower than that observed in patients treated with CT alone.

Interestingly, the capability of EUS-HTP to modulate factors associated with disease progression such as inflammatory monocytes and APRIL/TNFSF13 reinforces the potential clinical relevance of this thermo-ablative technique. Monocytes play, in fact, a fundamental role in tumor development, and the “inflammatory” subtype of monocytes has the highest capacity to secrete cytokines associated with PDAC progression [54]. Indeed, inflammatory monocytes are significantly increased in the PB of PDAC patients, infiltrate PDAC tissue, promote epithelial-to-mesenchymal transition of PDAC cells, and inversely correlate with patients’ survival [52,55]. Monocyte mobilization from the bone marrow to PDAC appears to be driven by the CCL2/CCR2 chemokine axis, ultimately leading to their differentiation into tumor-associated macrophages (TAMs) [52]. It is therefore tempting to speculate that, although we did not directly assess the CCL2/CCR2 axis, HTP might have decreased circulating inflammatory monocytes by intercepting their mobilization from the bone marrow and homing into the pancreas [56].

On the other hand, the survival factor APRIL/TNFSF13 has been involved in the proliferation of T regulatory cells and elevated serum level of APRIL/TNFSF13, which are associated with poor prognosis in PDAC patients [57,58]. By decreasing the availability of APRIL/TNFSF13 in the tumor microenvironment, HTP might have also affected T regulatory cells’ expansion.

Whether these immunomodulatory effects reflect the improvement of the clinical outcomes at six months that we observed in the RCT in patients randomized to HTP-CT remains to be clarified [25]. In the present explorative study, we could not observe any significant impact of HTP-CT on PFS and OS, but the sample size was not powered to specifically address treatment outcomes.

Our work has both strengths and limitations. Strengths include (i) a first extensive profiling of the systemic immune response following cryo-thermal ablation in PDAC patients; and (ii) the enrolment of a uniform and tightly selected population of treatment-naïve PDAC patients through a randomization process that ensured similar baseline clinical and immunological features between the HTP-CT and CT arms. On the other hand, major limitations include (i) the exploratory nature of the study that does not include mechanistic analysis; (ii) the small sample size that prevents solid statistical conclusions and limits the generalizability of the findings; (iii) the lack of a longer follow-up period providing better insights into EUS-HTP effects on the immune system and patients’ outcomes; (iv) the confounder effect of CT that does not allow full characterization of the immunomodulatory impact of EUS-HTP; and (v) the lack of information about EUS-HTP mediated immune modulation at pancreatic level, about changes in the clonality of the T cell receptor repertoire, and about antigen-specific immune responses.

## 5. Conclusions

Our study provides the first evidence that EUS-HTP has the potential to selectively modulate immune cells and cytokines associated with poor outcomes in patients with locally advanced and borderline resectable PDAC. Further studies on a larger number of patients with longer follow-up and repeated sampling of serum and PBMCs overtime are required to understand whether these immunological effects also have a clinical impact on patients’ survival as well as to establish EUS-guided LTA as an additional therapeutic option for PDAC patients.

## Figures and Tables

**Table 1:** Baseline features of enrolled patients with borderline resectable and locally advanced pancreatic ductal adenocarcinoma.

Features	HTP-CT Arm						CT Arm						
	Pat. 1	Pat. 2	Pat. 3	Pat. 4	Pat. 5	Median (Range)	Pat. 1	Pat. 2	Pat. 3	Pat. 4	Pat. 5	Median (Range)	*p*-Value
Sex, M/F, n (%)	M	M	F	F	F	-	M	M	F	M	F	-	0.55
Age (years)	68	73	77	61	74	64 (61–68)	67	54	57	65	59	59 (54–67)	0.21
Tumor site, H/B/T, n (%)	H	B	H	B	B	-	B	H	B	H	H	-	0.55
Tumor size (mm) at MDCT													
-short axis	20.3	34.1	29.9	42.7	22.1	29.9 (20.3–42.7)	64.5	21.9	29.7	34.7	33	33 (21.9–65.5)	0.60
-long axis	33.8	58	44.7	50.1	32.3	44.7 (32.3–58)	95.4	29.3	37.4	49.9	44.7	44.7 (29.3–95.4)	1.00
Tumor volume (cc) at MDCT	12.14	31.34	25.29	35.97	10.15	25.3 (10.1–36)	101.4	5.3	16.85	29.4	21.8	21.8 (5.3–101.4)	0.92
Tumor staging, BR/LA, n (%)	BR	LA	LA	LA	LA	-	LA	LA	LA	LA	BR	-	1.00
CA19.9 serum levels (U/mL)	362	2495	43	602	281	362 (43–2495)	3503	243.8	361	5192	20	361 (20–5192)	0.92
WBC serum levels (10^9^/L)	7.7	7.45	9.9	6.8	9.1	7.7 (6.8–9.9)	11.5	7.46	6.7	9.9	7.6	7.9 (6.7–11.5)	0.69
PLT serum levels (10^9^/L)	316	215	253	259	361	259 (215–361)	213	255	288	256	188	255 (188–325)	0.23
Neutrophils serum levels (10^9^/L)	4.9	3.99	6.5	4.6	6.7	4.9 (3.99–6.7)	8.4	5.63	2	6.3	4.3	4.6 (2–8.4)	0.99
Lymphocytes serum levels (10^9^/L)	1.9	1.99	2.5	1.5	1.6	1.5 (1.9–2.5)	2.1	1.2	3.2	2.3	2.1	2.15 (1.2–3.2)	0.46
Monocytes serum levels (10^9^/L)	0.6	0.83	0.8	0.6	0.7	0.7 (0.6–0.8)	0.8	0.54	0.5	0.9	0.8	0.65 (0.5–0.9)	0.98
Neutrophil/Lymphocyte ratio	2.58	2.01	2.6	3.07	4.19	2.6 (2–4.19)	4	4.69	0.625	2.74	2.05	2.74 (0.62–4.69)	0.92
PLT/Lymphocyte ratio	166.32	108.04	101.2	172.67	225.63	166.3 (101–226)	101.43	212.5	90	111.3	89.52	101.43 (90–212.5)	0.17
Lymphocyte/Monocyte ratio	3.17	2.397	3.13	2.5	2.29	2.5 (2.29–3.17)	2.625	2.22	6.4	2.56	2.62	2.62 (2.22–6.4)	0.75

HTP-CT: HybridTherm Probe ablation and chemotherapy; CT: Chemotherapy; M: males; F: females; H: pancreatic head; B: pancreatic body; T: pancreatic tail; MDCT: multidetector computed-tomography scan; BR: borderline resectable; LA: locally advanced; WBC: white blood cells; PLT: platelets.

## Data Availability

The datasets analyzed for this study are available upon request to the corresponding author.

## Supplementary Materials

The following supporting information can be downloaded at: https://www.mdpi.com/article/10.3390/cancers15143704/s1. Supplementary Material: (1) Peripheral blood samples processing; (2) Cell samples processing for flow-cytometry analysis; (3) Serum samples processing for multiplex immunoassays; Table S1. Detailed immune phenotypes of cell subsets analysed by flow-cytometry; Table S2. Detail of soluble analytes quantified by multiplex Luminex assays; Figure S1. Clinical flow-chart of randomized controlled study; Figure S2. Flow-cytometry gating strategies for immunophenotyping of cytotoxic T cells and T regulatory cells (A), T helper and T follicular helper lymphocytes (B), B-cell subsets (C), B regulatory cell subsets (D) and monocyte subsets (E); Figure S3. Progression free and overall survival in patients treated with HybridTherm Probe ablation plus chemotherapy (HTP-CT arm) and those with chemotherapy alone (CT arm). Kaplan Mejer curves showing the progression-free survival (A) and overall survival (B) of patients with borderline resectable and locally advanced pancreatic ductal adenocarcinoma treated with HybridTherm Probe ablation plus chemotherapy and those treated with chemotherapy only. Survival curves were compared using the Log-rank Mantel–Cox test: not significant (NS) = p > 0.05; Figure S4. Differences of circulating immune cells (A: T-cell compartment; B: B-cell compartment; C: monocyte compartment) and concentrations of inflammatory and immune-related cytokines and chemokines (D: Pro Human Inflammation Panel I Assay; E: Pro TGF-β Immunoassay; F: Pro Human Cytokine Immunoassay) between patients with pancreatic adenocarcinoma (PDAC) and sex- and age-matched healthy donors (HD) at baseline. Inter-group variables were compared using the Welch two sample *t*-test: * = *p* < 0.05; Figure S5. Correlation studies between circulating immune cells (A: T-cell compartment; B: B-cell compartment; C: monocyte compartment) and concentrations of inflammatory and immune-related cytokines and chemokines (D: Pro Human Inflammation Panel I Assay; E: Pro TGF-β Immunoassay; F: Pro Human Cytokine Immunoassay) at baseline and progression-free survival (PFS) after therapy onset in patients with pancreatic adenocarcinoma (PDAC). Correlation studies were performed using the Spearman’s rho rank correlation test; Figure S6. Correlation studies between circulating immune cells (A: T-cell compartment; B: B-cell compartment; C: monocyte compartment) and concentrations of inflammatory and immune-related cytokines and chemokines (D: Pro Human Inflammation Panel I Assay; E: Pro TGF-β Immunoassay; F: Pro Human Cytokine Immunoassay) at baseline and overall survival (OS) after therapy onset in patients with pancreatic adenocarcinoma (PDAC). Correlation studies were performed using the Spearman’s rho rank correlation test; Figure S7. Correlation studies between circulating immune cells (A: T-cell compartment; B: B-cell compartment; C: monocyte compartment) and concentrations of inflammatory and immune-related cytokines and chemokines (D: Pro Human Inflammation Panel I Assay; E: Pro TGF-β Immunoassay; F: Pro Human Cytokine Immunoassay) at 4-month follow-up and progression-free survival (PFS) after therapy onset in patients with pancreatic adenocarcinoma (PDAC). Correlation studies were performed using the Spearman’s rho rank correlation test; Figure S8. Correlation studies between circulating immune cells (A: T-cell compartment; B: B-cell compartment; C: monocyte compartment) and concentrations of inflammatory and immune-related cytokines and chemokines (D: Pro Human Inflammation Panel I Assay; E: Pro TGF-β Immunoassay; F: Pro Human Cytokine Immunoassay) at 4-month follow-up and overall survival (OS) after therapy onset in patients with pancreatic adenocarcinoma (PDAC). Correlation studies were performed using the Spearman’s rho rank correlation test; Figure S9. Correlation studies between circulating immune cells (A, G: T-cell compartment; B, H: B-cell compartment; C, I: monocyte compartment) and concentrations of inflammatory and immune-related cytokines and chemokines (D, J: Pro Human Inflammation Panel I Assay; E, K: Pro TGF-β Immunoassay; F, L: Pro Human Cytokine Immunoassay) at 4-month follow-up and progression-free survival (PFS) (A-F) and overall survival (OS) (G-L) after therapy onset in patients with pancreatic adenocarcinoma (PDAC) of the HybridTherm ablation plus chemotherapy (HTP-CT) arm. Correlation studies were performed using the Spearman’s rho rank correlation test; Figure S10. Correlation studies between circulating immune cells (A, G: T-cell compartment; B, H: B-cell compartment; C, I: monocyte compartment) and concentrations of inflammatory and immune-related cytokines and chemokines (D, J: Pro Human Inflammation Panel I Assay; E, K: Pro TGF-β Immunoassay; F, L: Pro Human Cytokine Immunoassay) at 4-month follow-up and progression-free survival (PFS) (A-F) and overall survival (OS) (G-L) after therapy onset in patients with pancreatic adenocarcinoma (PDAC) of the chemotherapy (CT) arm. Correlation studies were performed using the Spearman’s rho rank correlation test; Figure S11. Differences of circulating immune cells (A: T-cell compartment; B: B-cell compartment; C: monocyte compartment) and concentrations of inflammatory and immune-related cytokines and chemokines (D: Pro Human Inflammation Panel I Assay; E: Pro TGF-β Immunoassay; F: Pro Human Cytokine Immunoassay) between patients with pancreatic adenocarcinoma (PDAC) treated with HybridTherm Probe ablation plus chemotherapy (HTP-CT arm) and those treated with chemotherapy only (CT arm) at baseline. Inter-group variables were compared using the Welch two sample *t*-test: * = *p* < 0.05; Figure S12. Changes of circulating immune cells (A: T-cell compartment; B: B-cell compartment; C: monocyte compartment) and concentrations of inflammatory and immune-related cytokines and chemokines (D: Pro Human Inflammation Panel I Assay; E: Pro TGF-β Immunoassay; F: Pro Human Cytokine Immunoassay) between baseline (T0) and 4 months (T4M) after therapy onset in patients with pancreatic adenocarcinoma (PDAC) treated with chemotherapy only (CT arm). Square dot represents the patient in the CT arm who did not reach 4-month follow-up. Intra-group variables were compared using the paired *t*-test: significant (*) = *p* < 0.05; Figure S13. Differences of circulating immune cells (A: T-cell compartment; B: B-cell compartment; C: monocyte compartment) and concentrations of inflammatory and immune-related cytokines and chemokines (D: Pro Human Inflammation Panel I Assay; E: Pro TGF-β Immunoassay; F: Pro Human Cytokine Immunoassay) between patients with pancreatic adenocarcinoma (PDAC) treated with HybridTherm Probe ablation plus chemotherapy (HTP-CT arm) and those treated with chemotherapy only (CT arm) at 4-months follow-up after therapy onset. Inter-group variables were compared using the Welch two sample *t*-test: * = *p* < 0.05; Figure S14. Changes of circulating immune cells (A: T-cell compartment; B: B-cell compartment; C: monocyte compartment) and concentrations of inflammatory and immune-related cytokines and chemokines (D: Pro Human Inflammation Panel I Assay; E: Pro TGF-β Immunoassay; F: Pro Human Cytokine Immunoassay) between baseline (T0) and 4 months (T4M) after therapy onset in patients with pancreatic adenocarcinoma (PDAC) treated with HybridTherm Probe ablation plus chemotherapy (HTP-CT arm). Intra-group variables were compared using the paired *t*-test: * = *p* < 0.05; Figure S15. Correlation studies between circulating immune cells and cytokines significantly different in patients with pancreatic adenocarcinoma (PDAC) treated with HybridTherm Probe ablation plus chemotherapy (HTP-CT arm) from those treated with chemotherapy only (CT arm) at 4-month follow-up, and the tumor volume (A) and serum levels of tumor marker CA19-9 (B). Correlation studies were performed using the Spearman’s rho rank correlation test.
