# Supplementary material for: Immunomodulatory Effects of Endoscopic Ultrasound-Guided Thermal Ablation in Patients with Pancreatic Ductal Adenocarcinoma
Source: Cancers (Basel). 2023 Jul 21;15(14):3704. doi: 10.3390/cancers15143704 (PMC10378428; doi:10.3390/cancers15143704)
Supplement: Supplementary file 1 [file cancers-15-03704-s001.zip › cancers-2455601-supplementary.pdf]

cancers

Article

Immunomodulatory effects of endoscopic ultrasound-guided thermal ablation in patients with pancreatic ductal adenocarcinoma

Sabrina Gloria Giulia Testoni, Claudia Minici, Elisa Benetti, Francesca Clemente, Daniela Boselli, Clara Sciorati, Lucia De Monte, Maria Chiara Petrone, Markus Enderle, Walter Linzenbold, Maria Pia Protti, Angelo Manfredi, Francesco De Cobelli, Michele Reni, Massimo Falconi, Gabriele Capurso, Paolo Giorgio Arcidiacono, and Emanuel Della-Torre

Supplementary Material

1. Peripheral blood samples processing

PBMCs were isolated from fresh heparinized blood samples, previously diluted with buffered saline phosphate (PBS; ratio of 1 volume of PB for 2 volumes of PBS), by Ficoll-Hypaque density gradient (GE Healthcare Srl), after stratification on Ficoll-Hypaque (ratio of 3 volumes of PB for 1 volume of Ficoll-Hypaque) and centrifugation at 2500 x g for 20 minutes. The obtained PBMCs were subjected to repeated washing with buffered saline phosphate (PBS) and resuspended in a Roswell Park Memorial Institute (RPMI) 1640 medium integrated with 4-(2-hydroxyethyl)-1-piperazineethanesulfonic acid (HEPES) 25 mmol/L, L-glutamine 2 mmol/L, Gentamicin 50 μg/mL and 8% human serum. PBMCs were then immediately titled through an automatic cell counter and frozen in liquid nitrogen in a medium consisting of 90% fetal bovine serum (FBS) and 10% dimethyl sulfoxide (DMSO).

Serum samples were obtained through centrifugation of PB samples at 3000 rpm for 20 minutes and immediately frozen in liquid nitrogen.

2. Cell samples processing for flow-cytometry analysis

The following five flow-cytometric panels were designed:

- Panel I: CX3CR1-FITC (clone: 2A9-1); CD115-PE (clone:9-4D2-1E4); CCR2-PerCP-Cy5.5 (clone: K036C2); CD45-PE/Cy7 (clone: HI30); CD16-APC (clone: 3G8); CD14-APC/Cy7 (clone: M5E2); CD11b-Pacific Blue (clone: ICRF44); HLA-DR-Brilliant Violet 510 (clone: L243).
- Panel II: CXCR3-FITC (clone: G025H7); CCR6-PE (clone: 11A9); PD1-PerCP/Cy5.5 (clone: EH12.2H7); CXCR5-PE/Cy7 (clone: J252D4); CD45RO-APC (clone: UCHL1); ICOS-Super Bright 436 (clone: ISA-3); CD4-Brilliant Violet 510 (clone: A161A1).
- Panel III: CD8a-FITC (clone: HIT8a); CD25-PE (clone: RPA-T4); CD127-PerCP/Cy5.5 (clone: HIL-7R-M21); CD62L-PE/Cy7 (clone: DREG-56); SLAMF7 (CD319)-APC (clone: 162.1); CD27-APC/Cy7 (clone: O323); CD45RO-Pacific Blue (clone: UCHL1); CD4-Brilliant Violet 510 (clone: A161A1).
- Panel IV: CD14-Alexa Fluor 488 (clone: 63D3); CD138-PE dazzle (clone: MI15); CD45RB-PE (clone: MEM-55); IgM-Brilliant Violet 650 (clone: G20-127); CD21-Brilliant Ultra Violet 496 (clone: B-ly4); CD27- Brilliant Ultra Violet 737 (clone: L128); CD3-PerCP-Cy5.5 (clone: HIT3a); CD10-Alexa Fluor 700 (clone: HI10a); IgD-Brilliant Violet 421 (clone: IA6-2); CD38-Brilliant Violet 510 (clone: HB-7); CD19-Brilliant Violet 605 (clone: HIB19); CD73-Brilliant Ultra Violet 395 (clone: AD2); CXCR5-PE/Cy7 (clone: J252D4); CD11c-APC (clone: B-ly6).
- Panel V: CD14-Alexa Fluor 488 (clone: 63D3); CD3-PerCP-Cy5.5 (clone: HIT3a); CD24-APC/Cy7 (clone: ML5); CD38-Brilliant Violet 510 (clone: HB-7); CD19-Brilliant Violet 605 (clone: HIB19); CD27- Brilliant Ultra Violet 737 (clone: L128); TGFβ1-PE (clone: TW4-9E7); IL10-APC (clone: JES3-19F1).

After thawing, stored PBMCs suspensions were revitalized in a cell medium consisting of RPMI 1640-GlutaMax medium (Gibco), 10% heated-inactivated FBS (Euroclone SpA), 1% P/S (Gibco), 1% non-essential aminoacids (100x) (GibcoTM), 1 mM Sodium-pyruvate (100x) (Gibco), 50 uM 1-mercaptoethanol (GibcoTM) and 1% Penicillin-Streptomycin mixture (PenStrep, Gibco). Subsequently, revitalized PBMCs were counted adding Trypan blue (dilution 1:4) and resuspended at 5 x 106 cells/ml.

After repeated washing with PBS, all PBMCs suspensions were live/dead single stained with Viakrome 808 fixable viability dye (Beckman Coulter, 2.5 µl for 5 x 106 cells) for identification of viable cells, and incubated for 20 minutes in dark room. Therefore, incubation with human FcR blocking reagent (Miltenyi Biotec) for 10 minutes at 4°C and staining with titled surface mAbs for 10 minutes in dark room were performed. After washing of fluorochrome-labeled PBMCs with a FACS buffer, consisting of Dulbecco's phosphate-buffered saline (DPBS) without Ca2+ e Mg2+ and 2% FBS, they were incubated with Fixation buffer (ThermoFisher Scientific, 200 µl) for 20 minutes at 4°C.

PBMCs suspensions intended for immunophenotyping of B regulatory cells, before staining with the fixable viability dye, were first incubated for 5 hours at 37°C with CpG-ODN 2006 (Aurogene, 1 µl for 0.5 x 106 cells), Ionomycin (Sigma-Aldrich, 1 µl), Phorbol 12-myristate 13-acetate (PMA, Sigma-Aldrich, 1 µl) and GolgiStop (Beckton Dickinson, 1 µl). For the intracellular staining, after performing surface staining, cells were incubated with the Cytofix/Cytoperm fixation/permeabilization solution (Beckton Dickinson, 200 µl) for 20 minutes at 4°C in dark room, washed with the Perm/Wash Buffer (Beckton Dickinson, 1 ml diluted 1:10 in H2O), incubated with the intracellular mAbs for 30 minutes in dark room, and still washed with the Perm/Wash Buffer.

All stained PBMCs were then resuspended in 200 µl of FACS buffer and stored at 4°C in dark room until acquisition using the CytoFLEX LX Flow Cytometer.

For compensation adjustments the CompBeads Compensation Particles Set (Beckton Dickinson) was used. Before each flow cytometric acquisition, an instrument calibration was performed using the SpheroTM Rainbow Calibration Particles (Spherotech Inc.) for long-term performance tracking.

3. Serum samples processing for multiplex immunoassays

After thawing samples, 25 µg of diluted serum (1:4 for the 37-BioPlex and 27-BioPlex assays, 1:16 for the 3-BioPlex assay) and an eight¬point standard curve (plus blank) provided by the commercial assays were analysed for each of the different factors.

Using the 27-BioPlex and 37-BioPlex assays, diluted serum was incubated with the antibody-conjugated magnetic beads at room temperature for 1 hour on a plate shaker at 850 ± 50 rpm. Using the 3-BioPlex assay, serum samples were first activated by adding 1 N-HCL (1:5) for 10 minutes at room temperature and neutralized by adding same volume of the 1.2 N NaOH/0.5 M HEPES buffer base. Diluted samples were then incubated with the antibody-conjugated magnetic beads on plate shaker at 850 ± 50 rpm at room temperature for 2 hours.

Bead-complexes were then incubated with the detection antibody diluent (25 µl) for 30 minutes (27-BioPlex and 37-BioPlex) or 1 hour (3-BioPlex) and with streptavidin-phycoerythrin (50 µl) for 10 minutes (27-BioPlex and 37-BioPlex) or 30 minutes (3-BioPlex) on a plate shaker at 850 ± 50 rpm at room temperature, after rinsing three times before each of these passages. After washing three times, beads were re-suspended in the assay buffer (125 µl) for 30 seconds on a plate shaker at 850 ± 50 rpm at room temperature and, thus, immediately read.

Supplementary Tables

Table S1. Detailed immune phenotypes of cell subsets analysed by flow-cytometry.

	**Immune cell subsets**
**Panel I**	*CD45+CD115+CD11b+HLA-DR+* monocytes
	CD45+CD115+CD11b+HLA-DR- immunosuppressive monocytes
	CD45+CD115+CD11b+HLA-DR+CD16-CD14+ classical monocytes
	CD45+CD115+CD11b+HLA-DR+CD16+CD14+ intermediate monocytes
	CD45+CD115+CD11b+HLA-DR+CD16+CD14low non-classical monocytes
	CD45+CD115+CD11b+HLA-DR+CD16+CX3CR1high resident monocytes
	CD45+CD115+CD11b+HLA-DR+CD16-CX3CR1lowCD14+CCR2+ inflammatory monocytes
**Panel II**	*CD4+ T* cells
	CD4+CD45RO+ T central memory cells
	CD4+CD45RO+CD62L-CD27- T effector memory cells
	CD4+CD45RO+CD62L-CD27-CD319(SLAMF7)+ T CD4+ cytotoxic cells
	CD8+CD45RO+CD62L-CD27- CD319(SLAMF7)+ T CD8+ cytotoxic cells
	CD4+CD127lowCD25+ T regulatory cells
**Panel III**	*CD4+CD45RO+CXCR3+CCR6-* T helper 1 cells
	CD4+CD45RO+CXCR3-CCR6- T helper 2 cells
	CD4+CD45RO+CXCR3-CCR6+ T helper 17 cells
	CD4+CD45RO+CXCR3+CCR6+ T helper 1/17 cells
	CD4+CD45RO+CXCR5+ T follicular helper cells
	CD4+CD45RO+CXCR5+CXCR3+CCR6- T follicular helper 1 cells
	CD4+CD45RO+CXCR5+CXCR3-CCR6- T follicular helper 2 cells
	CD4+CD45RO+CXCR5+CXCR3-CCR6+ T follicular helper 17 cells
	CD4+CD45RO+CXCR5+CXCR3+CCR6+ T follicular helper 1/17 cells
	CD4+CD45RO+CXCR5+ICOS+PD-1+ activated T follicular helper cells
**Panel IV**	*CD3-CD14-CD19+* B cells
	CD3-CD14-CD19+CD38+CD27+ antibody secreting B cells
	CD3-CD14-CD19+CD38+CD27+CD138- antibody secreting plasmablasts
	CD3-CD14-CD19+CD38+CD27+CD138+ antibody secreting plasmacells
	CD3-CD14-CD19+CD38-CD27+ memory B cells
	CD3-CD14-CD19+CD38+/-CD27+IgD+ unswitched memory B cells
	CD3-CD14-CD19+CD38+/-CD27+IgD- switched memory B cells
	CD3-CD14-CD19+CD38+/-CD27+IgD-CD11c+CD21- activated switched memory B cells
	CD3-CD14-CD19+CD38+/-CD27-IgD- double negative B cells
	CD3-CD14-CD19+CD38+/-CD27-IgD-CD27-CXCR5+CD11c- double negative 1 B cells

	CD3-CD14-CD19+CD38+/-CD27-IgD-CD27-CXCR5-CD11c+ double negative 2 B cells<br>*CD3-CD14-CD19+CD38+/-CD27-IgD-CD27-CXCR5-CD11c-* double negative 3 B cells<br>*CD3-CD14-CD19+CD38+/-CD27-IgD-CD27-CXCR5+CD11c+* double negative 4 B cells<br>*CD3-CD14-CD19+CD38+/-CD27-IgD+* naïve B cells<br>*CD3-CD14-CD19+CD38+/-CD27-IgD+CD10-CD45RB-CD21-CD11c+* activated naïve B cells<br>*CD3-CD14-CD19+CD38+/-CD27-IgD+CD10+CD45RB-* transitional 1/2 B cells<br>*CD3-CD14-CD19+CD38+/-CD27-IgD+CD10-CD45RB+* marginal zone peripheral B cells<br>*CD3-CD14-CD19+CD38+/-CD27-IgD+CD27-CD10-CD45RB-CD21+/-CD11c-CXCR5+CD73+* follicular B cells<br>*CD3-CD14-CD19+CD38+/-CD27-IgD+CD27-CD10-CD45RB-CD21+/-CD11c-CXCR5+CD73*+CD21+IgM+ resting naïve B cells<br>*CD3-CD14-CD19+CD38+/-CD27-IgD+CD27-CD10-CD45RB-CD21+/-CD11c-CXCR5+CD73*+CD21-IgM- anergic naïve B cells<br>*CD3-CD14-CD19+CD38+/-CD27-IgD+CD27-CD10-CD45RB-CD21+/-CD11c-CXCR5+CD73-* transitional CXCR5+ T3a B cells<br>*CD3-CD14-CD19+CD38+/-IgD+CD27-CD10-CD54RB-CD21+/-CD11c-CXCR5-CD73-* transitional CXCR5- T3a B cells
**Panel V**	*CD3-CD14-CD19+CD24+CD38-* regulatory B cells<br>*CD3-CD14-CD19+CD24+CD38+* immature regulatory B cells<br>*CD3-CD14-CD19+CD24+CD27+* B10 regulatory B cells<br>*CD3-CD14-CD19+CD38+CD27+* antibody secreting regulatory B cells<br>*CD3-CD14-CD19+CD24+CD38-IL-10+* regulatory B cells expressing IL-10<br>*CD3-CD14-CD19+CD24+CD38+IL-10+* immature regulatory B cells expressing IL-10<br>*CD3-CD14-CD19+CD24+CD27+IL-10+* B10 regulatory B cells expressing IL-10<br>*CD3-CD14-CD19+CD38+CD27+IL-10+* antibody secreting regulatory B cells expressing IL-10<br>*CD3-CD14-CD19+CD24+CD38-TGF-β+* regulatory B cells expressing TGF-β<br>*CD3-CD14-CD19+CD24+CD38+TGF-β+* immature regulatory B cells expressing TGF-β<br>*CD3-CD14-CD19+CD24+CD27+TGF-β+* B10 regulatory B cells expressing TGF-β<br>*CD3-CD14-CD19+CD38+CD27+TGF-β+* antibody secreting regulatory B cells expressing TGF-β

Table S2. Detail of soluble analytes quantified by multiplex Luminex assays.

	**Soluble analytes**		**Soluble analytes**
**Pro Human Inflammation Panel I Assay 37-BioPlex**	APRIL / TNFSF13	**Pro Human Cytokine Immunoassay 27-BioPlex**	FGF basic
	BAFF / TNFSF13B		Eotaxin
	sCD30 / TNFRSF8		G-CSF
	sCD163		GM-CSF
	Chitinase-3-like 1		IFN-γ
	gp130 / sIL-6Rβ		IL-1β
	IFN-α2		IL-1ra
	IFN-β		IL-2
	IFN-γ		IL-4
	IL-2		IL-5
	sIL-6Rα		IL-6
	IL-8		IL-7
	IL-10		IL-8
	IL-11		IL-9
	IL-12 (p40)		IL-10
	IL-12 (p70)		IL-12 (p70)
	IL-19		IL-13
	IL-20		IL-15
	IL-22		IL-17A
	IL-26		IP-10
	IL-27 (p28)		MCP-1 (MCAF)
	IL-28A / IFN-λ2		MIP-1α
	IL-29 / IFN-λ1		MIP-1β
	IL-32		PDGF-BB
	IL-34		RANTES
	IL-35		TNF-α
	LIGHT / TNFSF14		VEGF
	MMP-1	**Pro TGF-β Immunoassay 3-BioPlex**	TGF-β1
	MMP-2		TGF-β2
	MMP-3		TGF-β3
	Osteocalcin		
	Osteopontin		
	Pentraxin-3		
	sTNF-R1		
	sTNF-R2		
	TSLP		
	TWEAK / TNFSF12		

Supplementary Figure legends

Figure S1. Clinical flow-chart of the randomized controlled study.

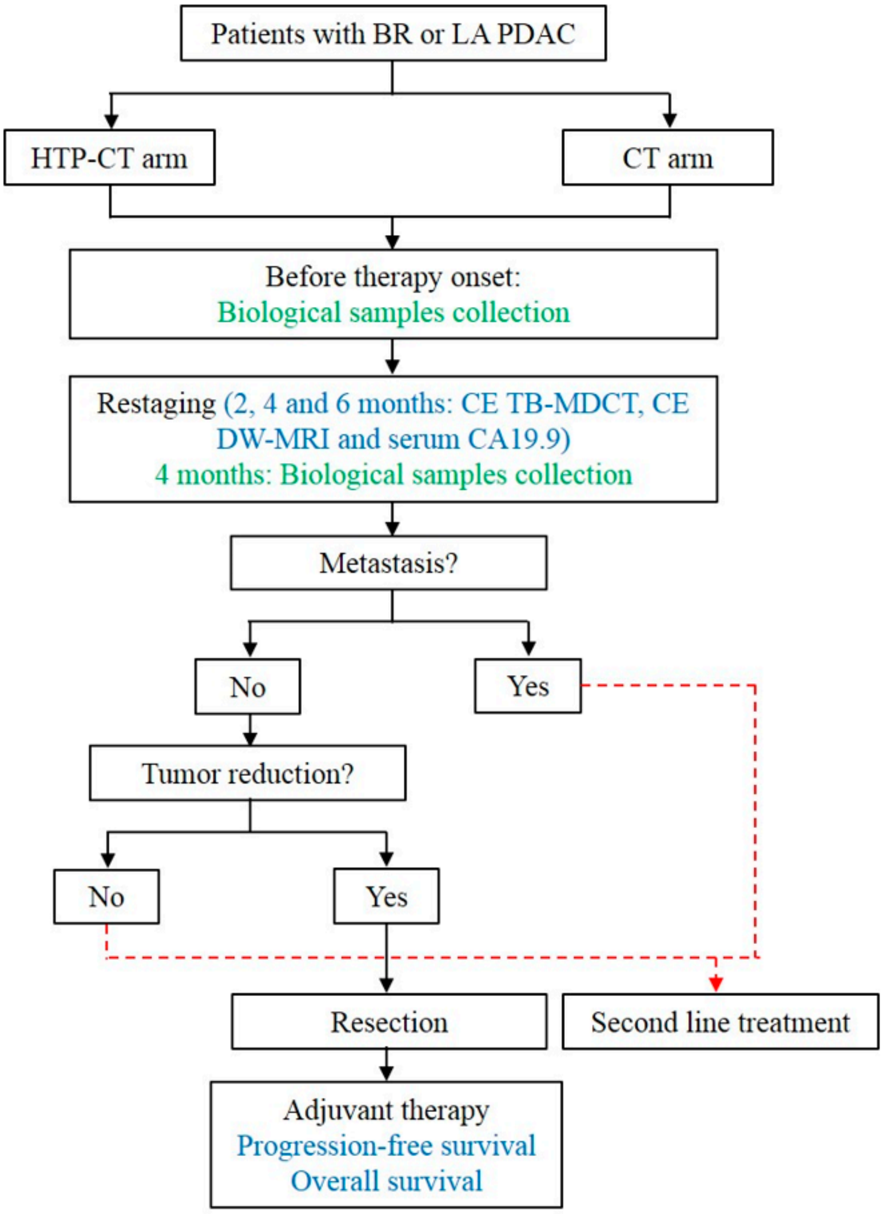

Footnote: BR: Borderline Resectable; LA: Locally Advanced; PDAC: Pancreatic Adenocarcinoma; HTP-CT: HybridTherm Probe ablation plus Chemotherapy; CT: Chemotherapy; CE: Contrast Enhanced; TB-MDCT: Total Body-MultiDetector Computed Tomography; DW-MRI: Double-Weighed Magnetic Resonance Imaging; CA19.9: Carbohydrate Antigen 19.9.

Figure S2. Flow-cytometry gating strategies for immunophenotyping of cytotoxic T cells and T regulatory cells (**A**), T helper and T follicular helper lymphocytes (**B**), B-cell subsets (**C**), B regulatory cell subsets (**D**) and monocyte subsets (**E**).

A

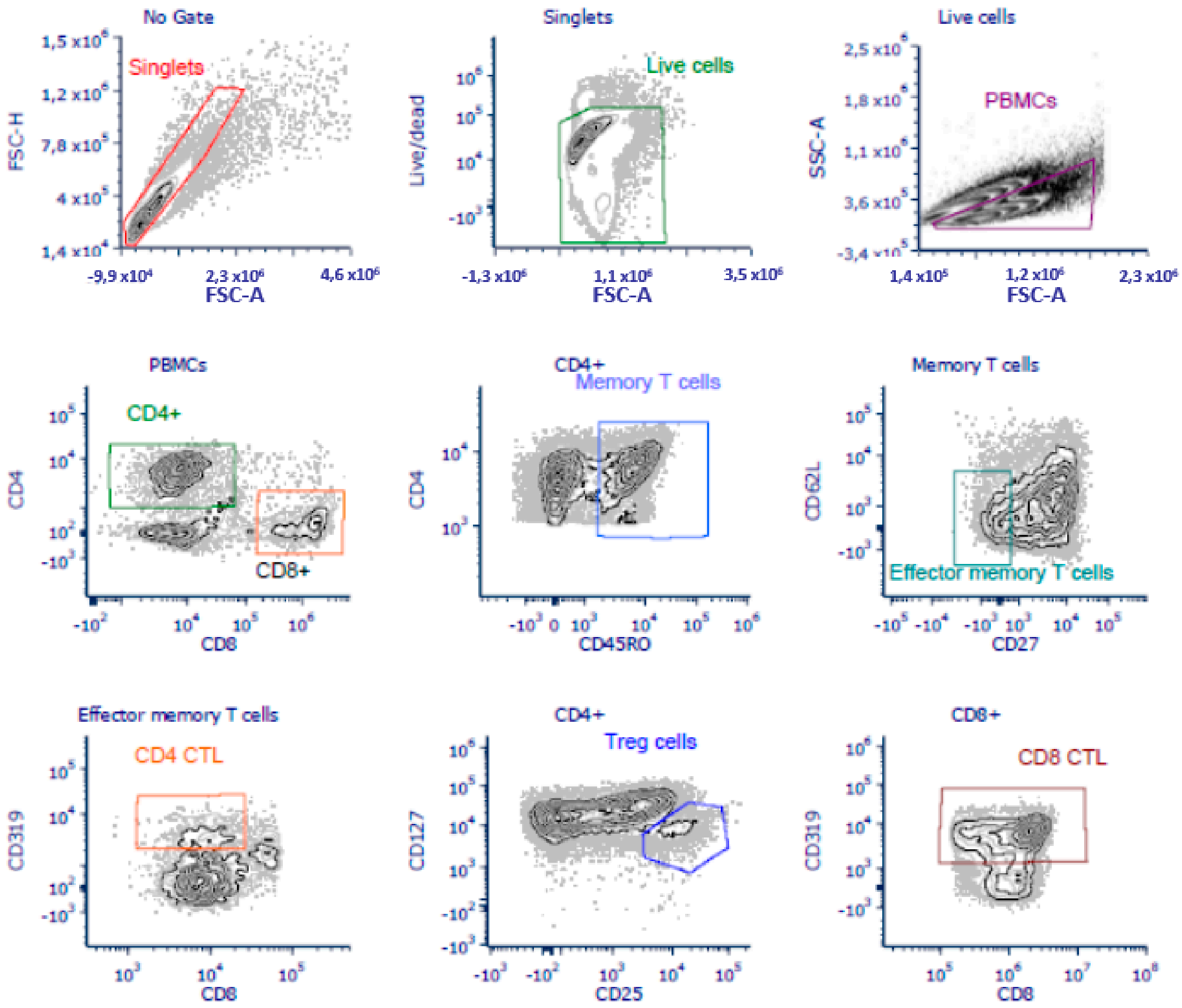

B

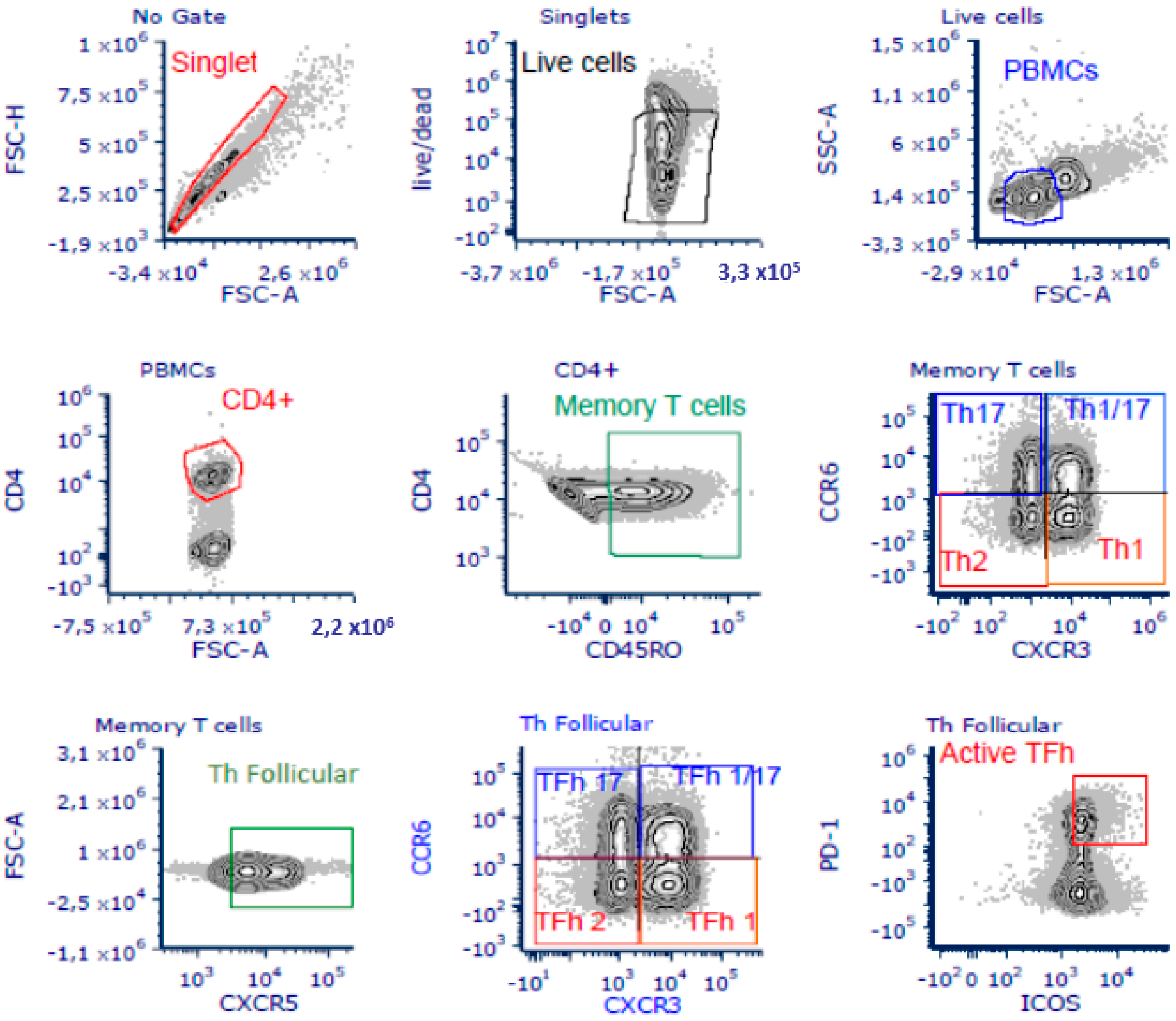
No Gate
Singlet
FSC-H
FSC-A
Singlets
Live cells
live/dead
FSC-A
Live cells
PBMCs
SSC-A
FSC-A
PBMCs
CD4+
CD4
FSC-A
CD4+
Memory T cells
CD4
CD45RO
Memory T cells
Th17
Th1/17
Th2
Th1
CCR6
CXCR3
Memory T cells
Th Follicular
FSC-A
CXCR5
Th Follicular
TFh 17
TFh 1/17
TFh 2
TFh 1
CCR6
CXCR3
Th Follicular
Active TFh
PD-1
ICOS

C

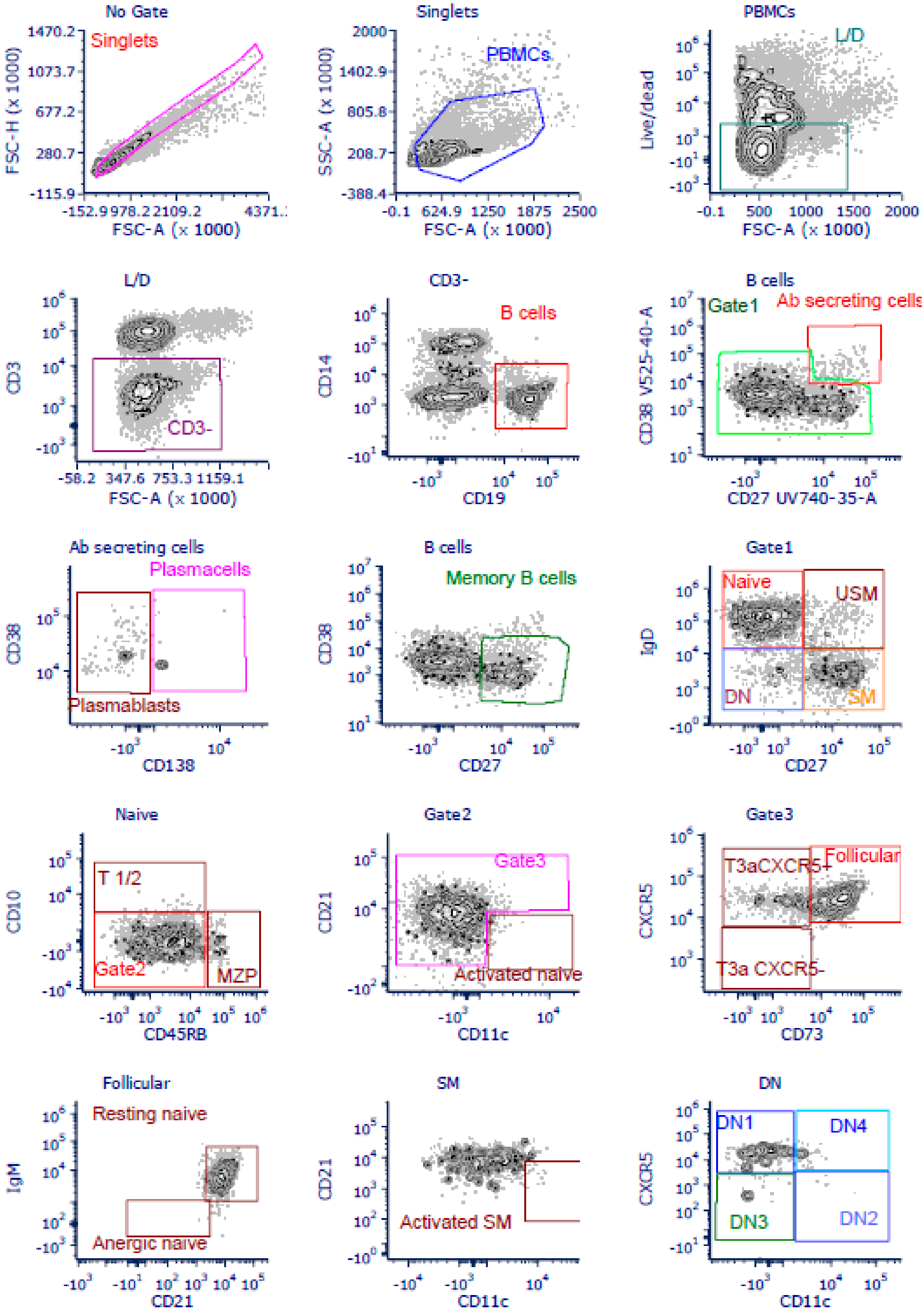
No Gate
Singlets
FSC-H (x 1000)
FSC-A (x 1000)
Singlets
PBMCs
SSC-A (x 1000)
FSC-A (x 1000)
PBMCs
L/D
Live/dead
FSC-A (x 1000)
L/D
CD3
CD3-
FSC-A (x 1000)
CD3-
B cells
CD14
CD19
B cells
Gate1
Ab secreting cells
CD38 V525-40-A
CD27 UV740-35-A
Ab secreting cells
Plasmacells
Plasmablasts
CD38
CD138
B cells
Memory B cells
CD38
CD27
Gate1
Naive
USM
DN
SM
IgD
CD27
Naive
T 1/2
Gate2
MZP
CD10
CD45RB
Gate2
Gate3
Activated naive
CD21
CD11c
Gate3
T3aCXCR5+
Follicular
T3a CXCR5-
CXCR5
CD73
Follicular
Resting naive
Anergic naive
IgM
CD21
SM
Activated SM
CD21
CD11c
DN
DN1
DN4
DN3
DN2
CXCR5
CD11c

D

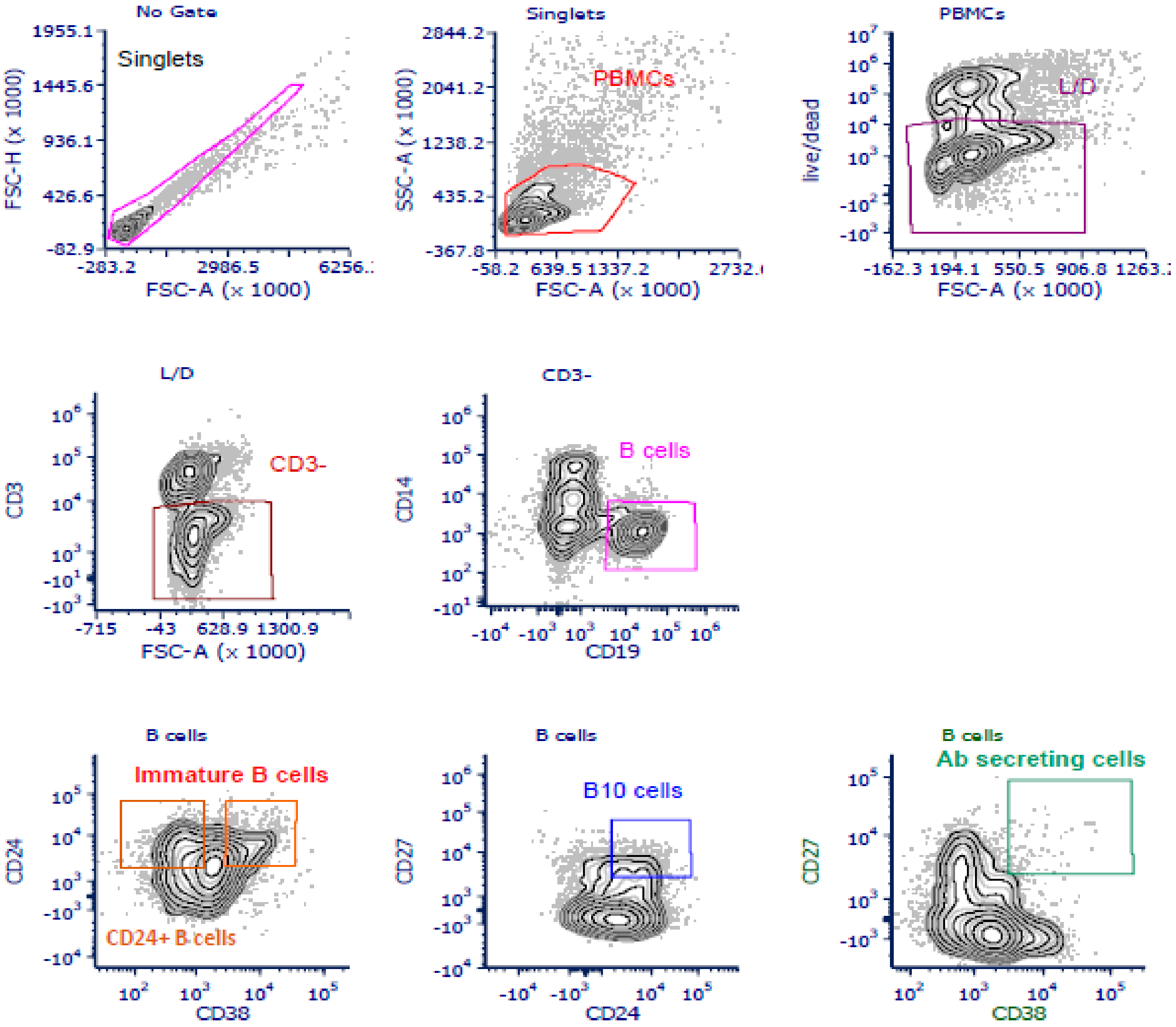
No Gate
Singlets
FSC-H (x 1000)
FSC-A (x 1000)
Singlets
PBMCs
SSC-A (x 1000)
FSC-A (x 1000)
PBMCs
L/D
live/dead
FSC-A (x 1000)
L/D
CD3-
CD3
FSC-A (x 1000)
CD3-
B cells
CD14
CD19
B cells
Immature B cells
CD24+ B cells
CD24
CD38
B cells
B10 cells
CD27
CD24
B cells
Ab secreting cells
CD27
CD38

E

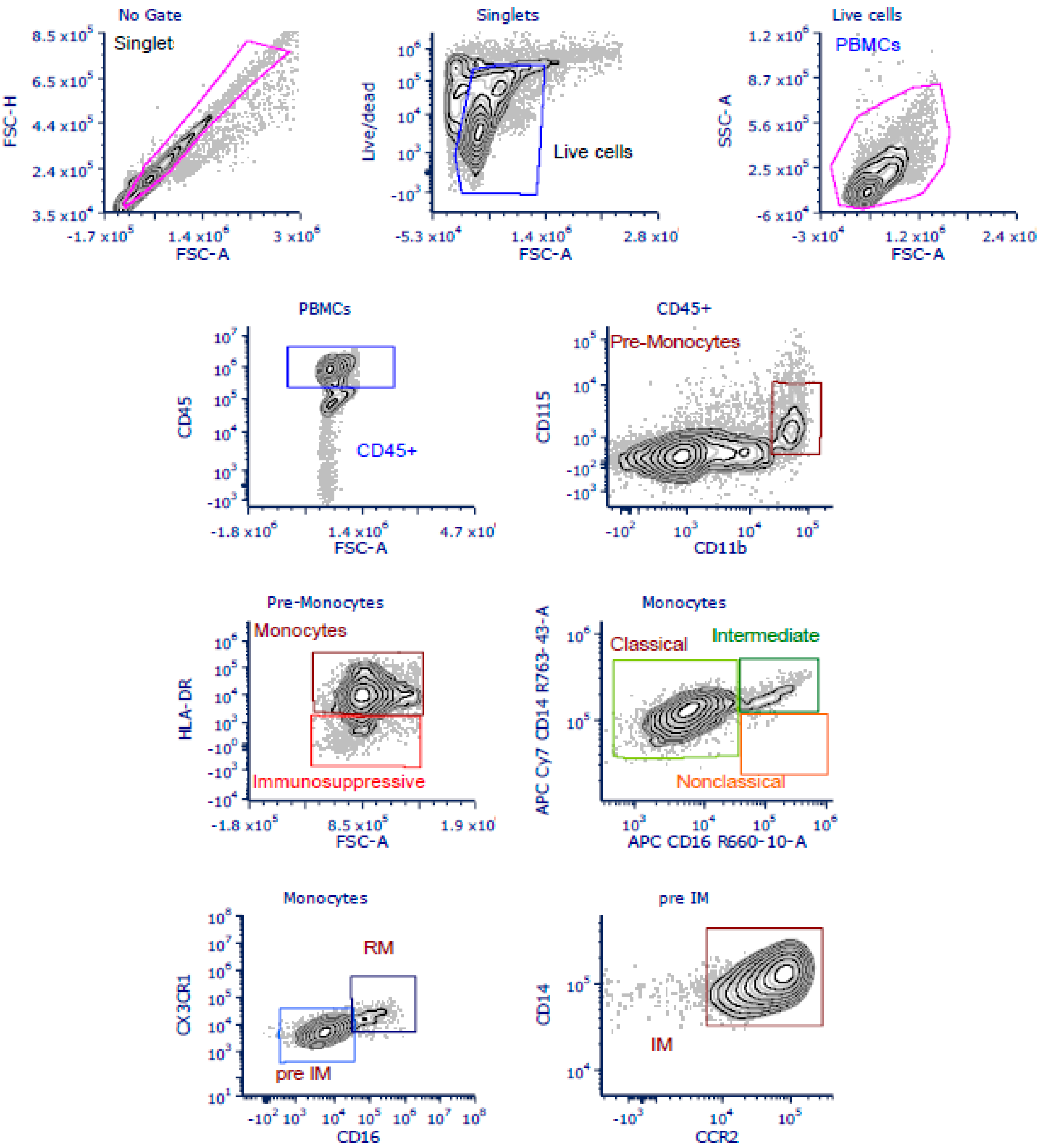

Footnote: PBMCs: peripheral mononucleated cells; CTLs: cytotoxic T lymphocytes; Treg: T regulatory; Th: T helper; Tfh: T follicular helper; L/D: live/dead; Ab: antibody; USM: unswitched memory; SM: switched memory; DN: double negative; T 1/2: transitional 1/2; MZP: marginal zone peripheral; T3a: transitional 3a; RM: resident monocytes; IM: inflammatory monocytes.

Figure S3. Progression free and overall survival in patients treated with HybridTherm Probe ablation plus chemotherapy (HTP-CT arm) and those with chemotherapy alone (CT arm). Kaplan Mejer curves showing the progression-free survival (**A**) and overall survival (**B**) of patients with borderline resectable and locally advanced pancreatic ductal adenocarcinoma treated with HybridTherm Probe ablation plus chemotherapy and those treated with chemotherapy only. Survival curves were compared using the Log-rank Mantel-Cox test: not significant (NS) = p > 0.05.

A

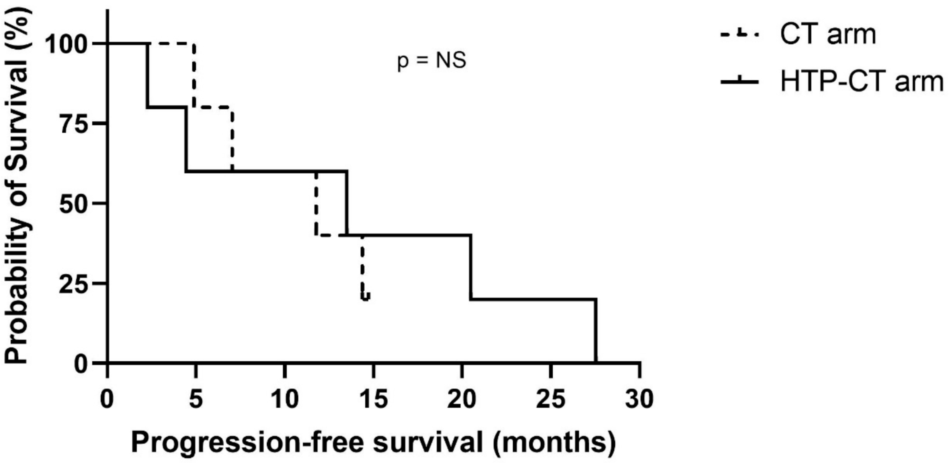

B

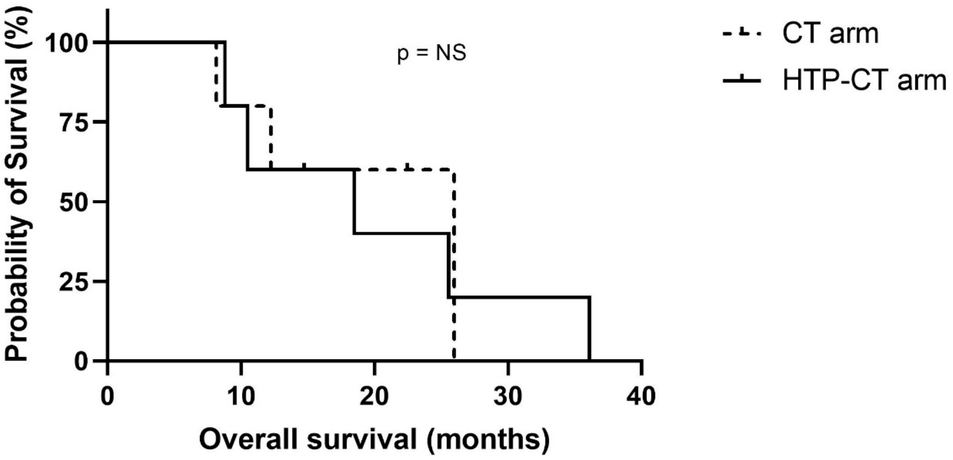

Figure S4. Differences of circulating immune cells (**A**: T-cell compartment; **B**: B-cell compartment; **C**: monocyte compartment) and concentrations of inflammatory and immune-related cytokines and chemokines (**D**: Pro Human Inflammation Panel I Assay; **E**: Pro TGF-β Immunoassay; **F**: Pro Human Cytokine Immunoassay) between patients with pancreatic adenocarcinoma (PDAC) and sex- and age-matched healthy donors (HD) at baseline. Inter-group variables were compared using the Welch two sample t-test: * = $p < 0.05$.

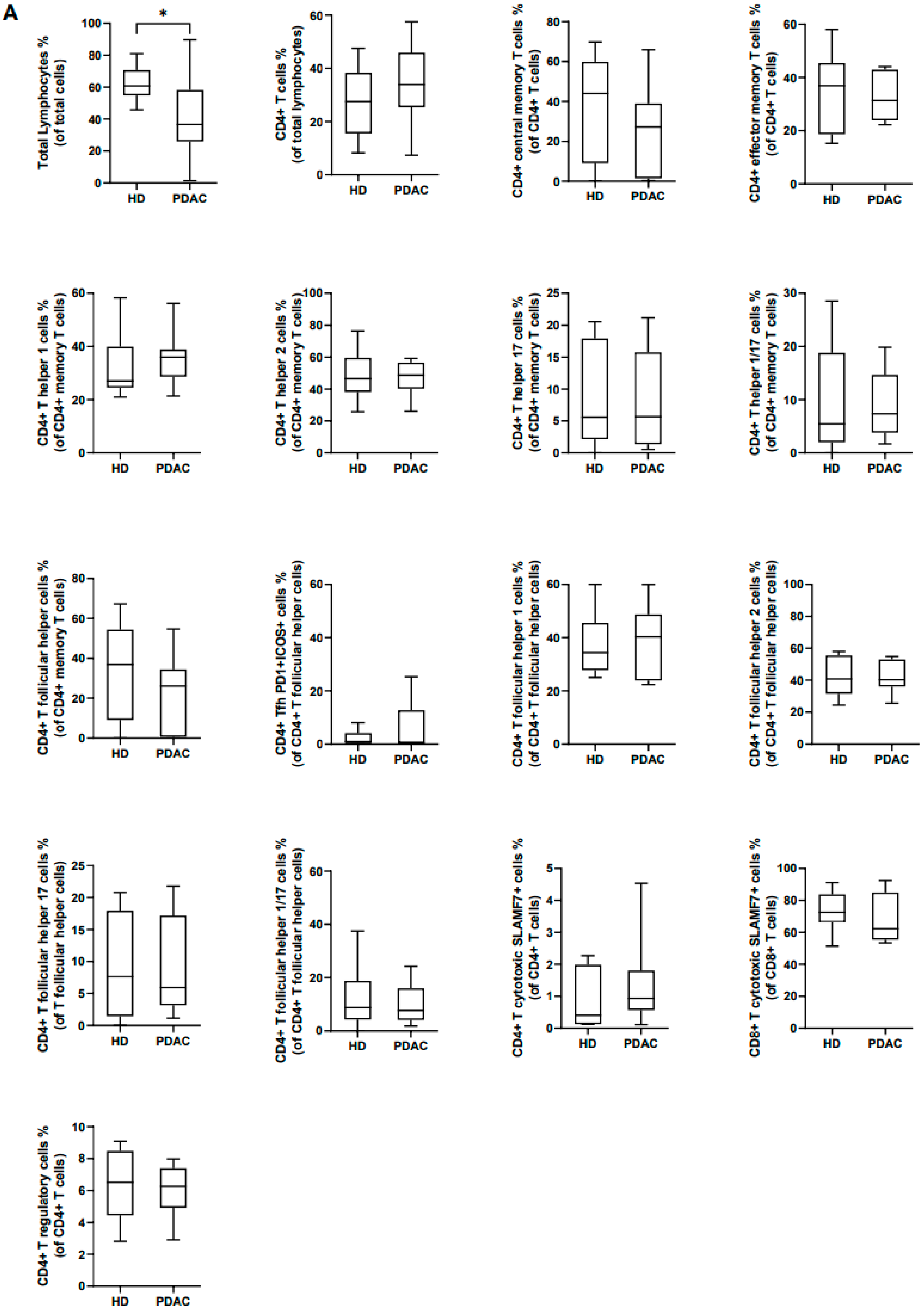

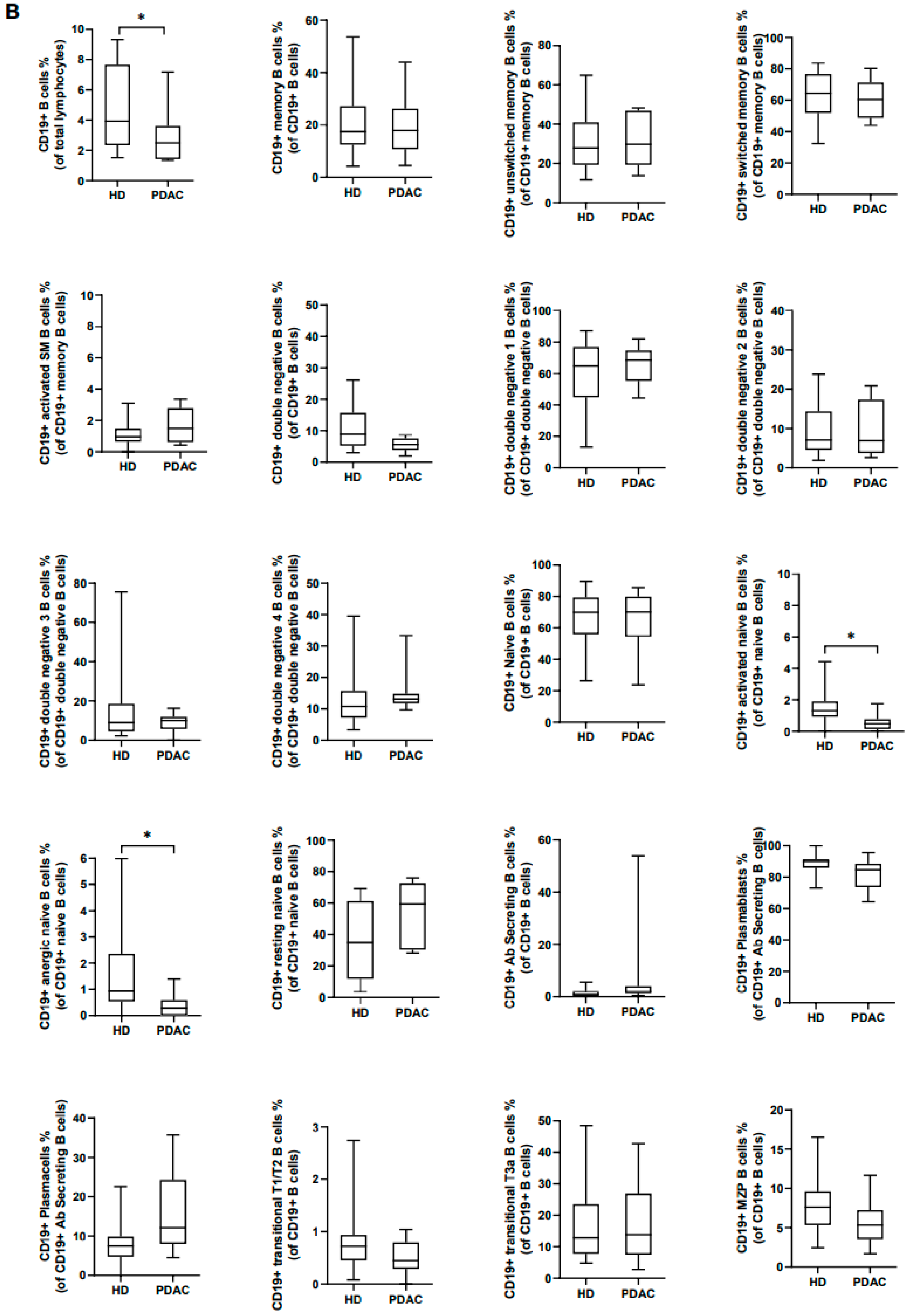
B
CD19+ B cells % (of total lymphocytes)
CD19+ memory B cells % (of CD19+ B cells)
CD19+ unswitched memory B cells % (of CD19+ memory B cells)
CD19+ switched memory B cells % (of CD19+ memory B cells)
CD19+ activated SM B cells % (of CD19+ memory B cells)
CD19+ double negative B cells % (of CD19+ B cells)
CD19+ double negative 1 B cells % (of CD19+ double negative B cells)
CD19+ double negative 2 B cells % (of CD19+ double negative B cells)
CD19+ double negative 3 B cells % (of CD19+ double negative B cells)
CD19+ double negative 4 B cells % (of CD19+ double negative B cells)
CD19+ Naive B cells % (of CD19+ B cells)
CD19+ activated naive B cells % (of CD19+ naive B cells)
CD19+ anergic naive B cells % (of CD19+ naive B cells)
CD19+ resting naive B cells % (of CD19+ naive B cells)
CD19+ Ab Secreting B cells % (of CD19+ B cells)
CD19+ Plasmablasts % (of CD19+ Ab Secreting B cells)
CD19+ Plasmacells % (of CD19+ Ab Secreting B cells)
CD19+ transitional T1/T2 B cells % (of CD19+ B cells)
CD19+ transitional T3a B cells % (of CD19+ B cells)
CD19+ MZP B cells % (of CD19+ B cells)
HD
PDAC
*

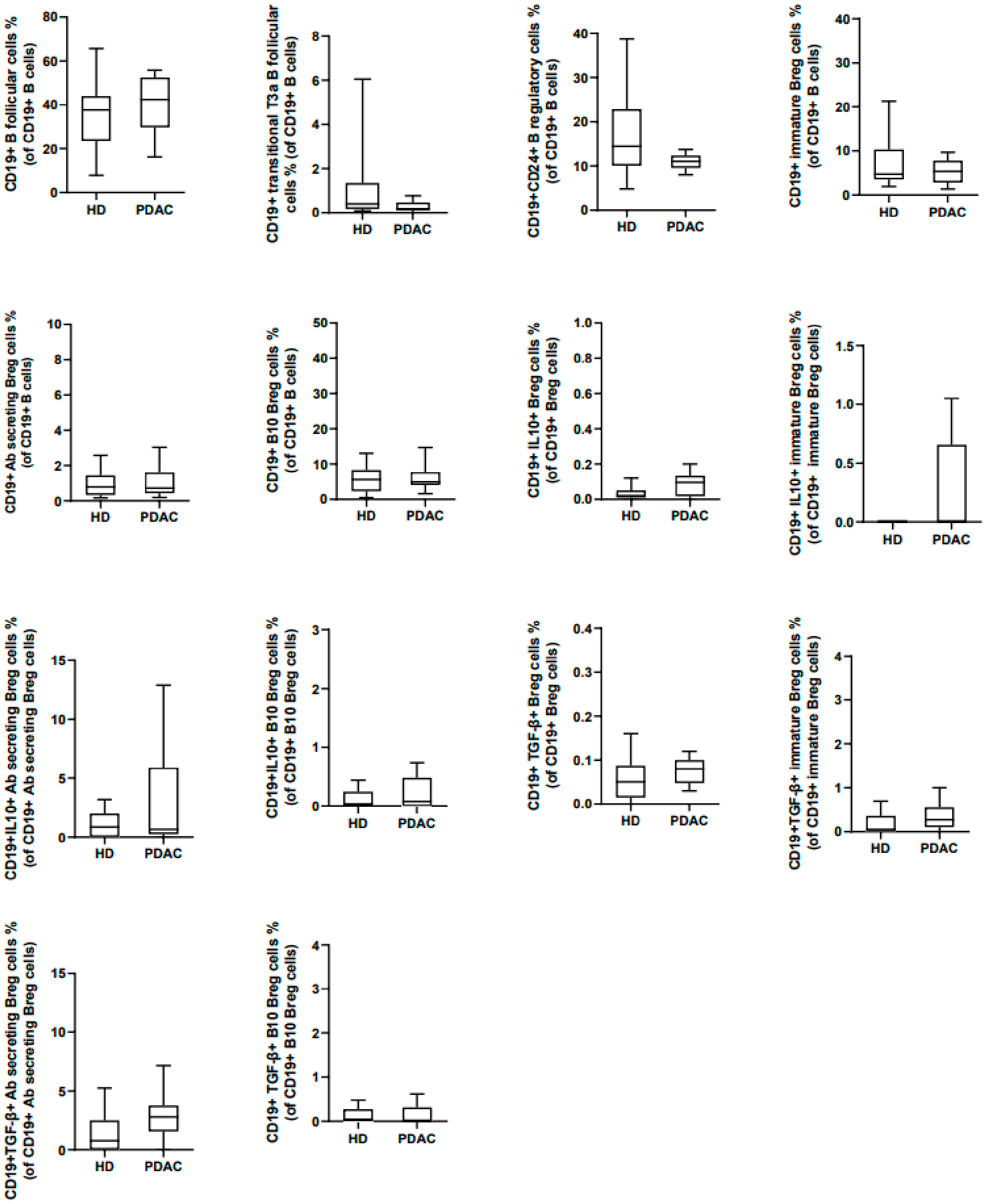
CD19+ B follicular cells % (of CD19+ B cells)
CD19+ transitional T3a B follicular cells % (of CD19+ B cells)
CD19+CD24+ B regulatory cells % (of CD19+ B cells)
CD19+ immature Breg cells % (of CD19+ B cells)
CD19+ Ab secreting Breg cells % (of CD19+B cells)
CD19+ B10 Breg cells % (of CD19+ B cells)
CD19+ IL10+ Breg cells % (of CD19+ Breg cells)
CD19+ IL10+ immature Breg cells % (of CD19+ immature Breg cells)
CD19+IL10+ Ab secreting Breg cells % (of CD19+ Ab secreting Breg cells)
CD19+IL10+ B10 Breg cells % (of CD19+ B10 Breg cells)
CD19+ TGF-β+ Breg cells % (of CD19+ Breg cells)
CD19+TGF-β+ immature Breg cells % (of CD19+ immature Breg cells)
CD19+TGF-β+ Ab secreting Breg cells % (of CD19+ Ab secreting Breg cells)
CD19+ TGF-β+ B10 Breg cells % (of CD19+ B10 Breg cells)
HD
PDAC

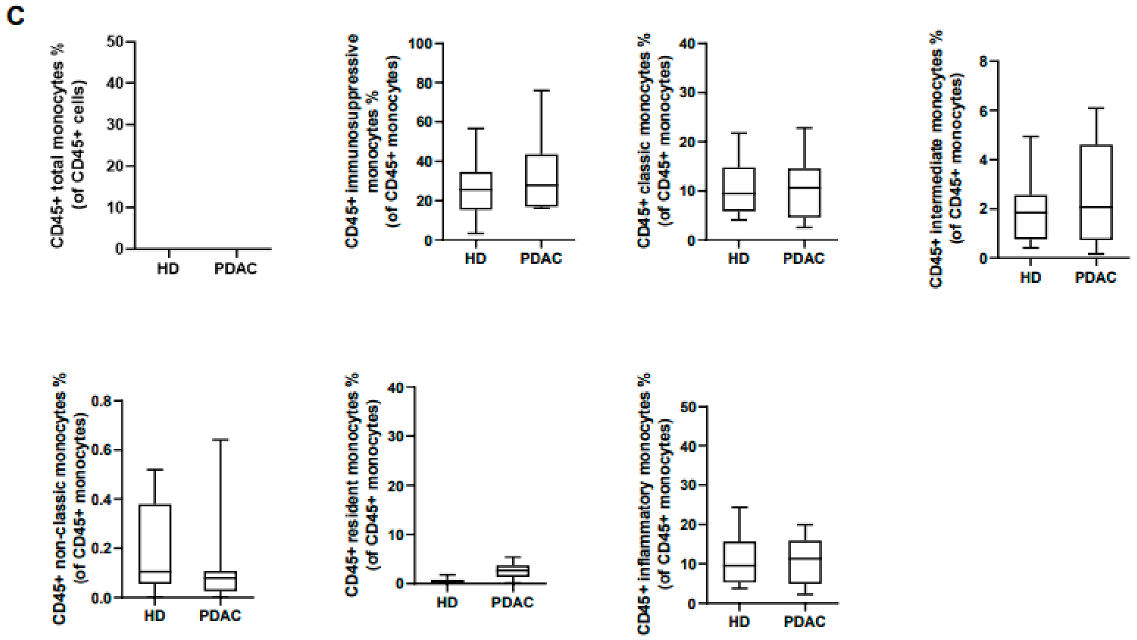
C
CD45+ total monocytes % (of CD45+ cells)
0
10
20
30
40
50
HD
PDAC
CD45+ immunosuppressive monocytes % (of CD45+ monocytes)
0
20
40
60
80
100
HD
PDAC
CD45+ classic monocytes % (of CD45+ monocytes)
0
10
20
30
40
HD
PDAC
CD45+ intermediate monocytes % (of CD45+ monocytes)
0
2
4
6
8
HD
PDAC
CD45+ non-classic monocytes % (of CD45+ monocytes)
0.0
0.2
0.4
0.6
0.8
HD
PDAC
CD45+ resident monocytes % (of CD45+ monocytes)
0
10
20
30
40
HD
PDAC
CD45+ inflammatory monocytes % (of CD45+ monocytes)
0
10
20
30
40
50
HD
PDAC

D

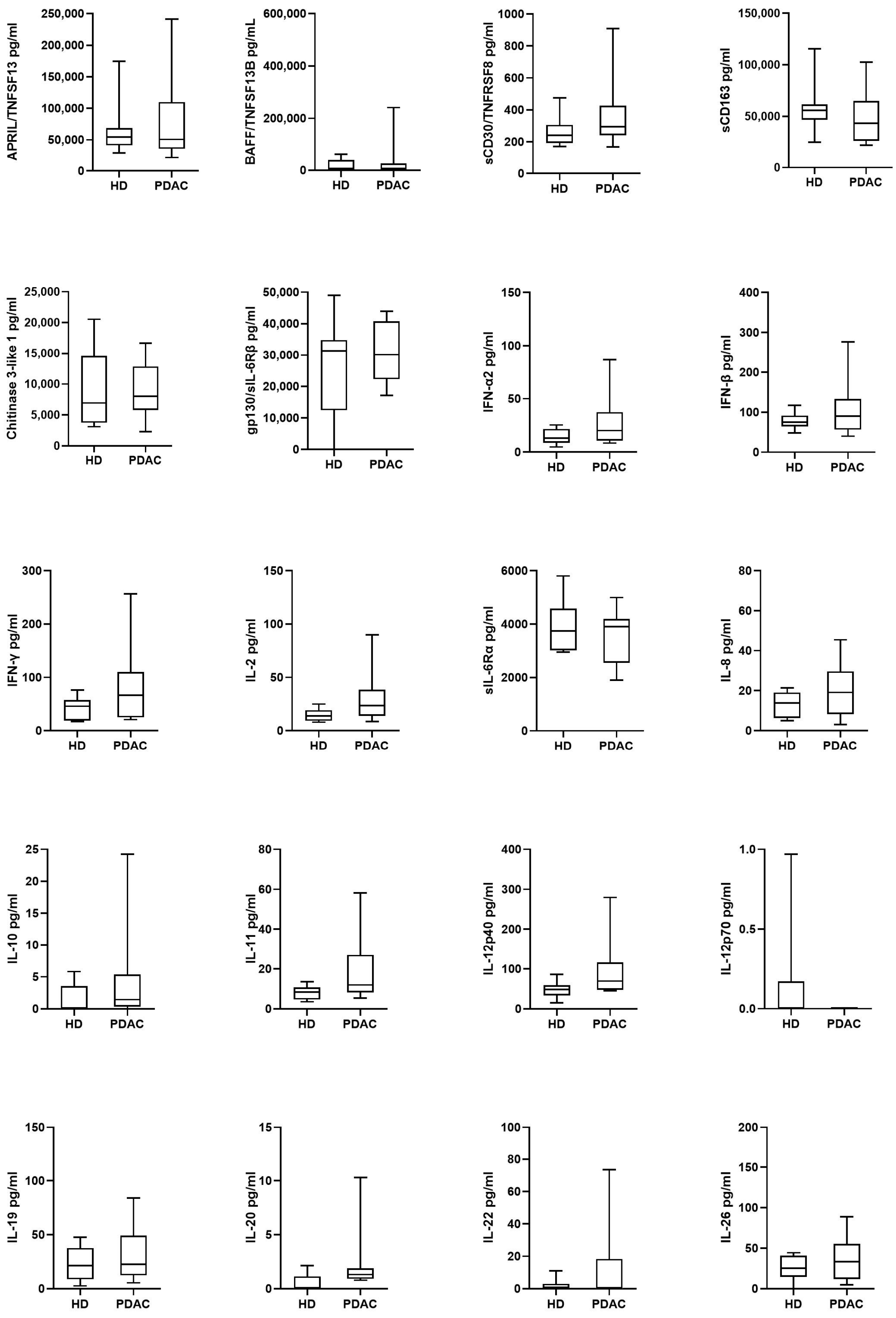
APRIL/TNFSF13 pg/ml
BAFF/TNFSF13B pg/mL
sCD30/TNFRSF8 pg/ml
sCD163 pg/ml
Chitinase 3-like 1 pg/ml
gp130/sIL-6Rβ pg/ml
IFN-α2 pg/ml
IFN-β pg/ml
IFN-γ pg/ml
IL-2 pg/ml
sIL-6Rα pg/ml
IL-8 pg/ml
IL-10 pg/ml
IL-11 pg/ml
IL-12p40 pg/ml
IL-12p70 pg/ml
IL-19 pg/ml
IL-20 pg/ml
IL-22 pg/ml
IL-26 pg/ml
HD
PDAC

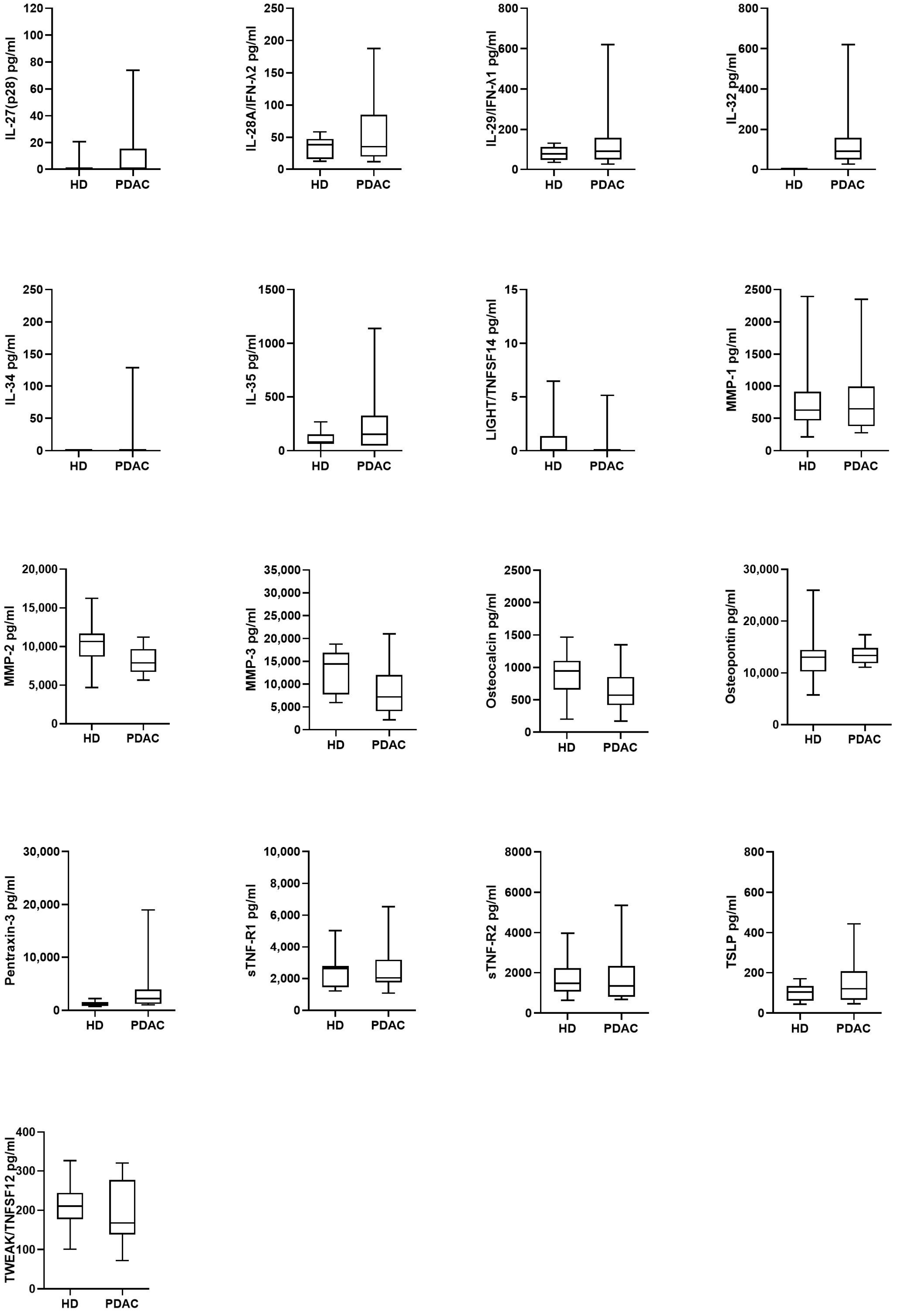
IL-27(p28) pg/ml
IL-28A/IFN-λ2 pg/ml
IL-29/IFN-λ1 pg/ml
IL-32 pg/ml
IL-34 pg/ml
IL-35 pg/ml
LIGHT/TNFSF14 pg/ml
MMP-1 pg/ml
MMP-2 pg/ml
MMP-3 pg/ml
Osteocalcin pg/ml
Osteopontin pg/ml
Pentraxin-3 pg/ml
sTNF-R1 pg/ml
sTNF-R2 pg/ml
TSLP pg/ml
TWEAK/TNFSF12 pg/ml
HD
PDAC

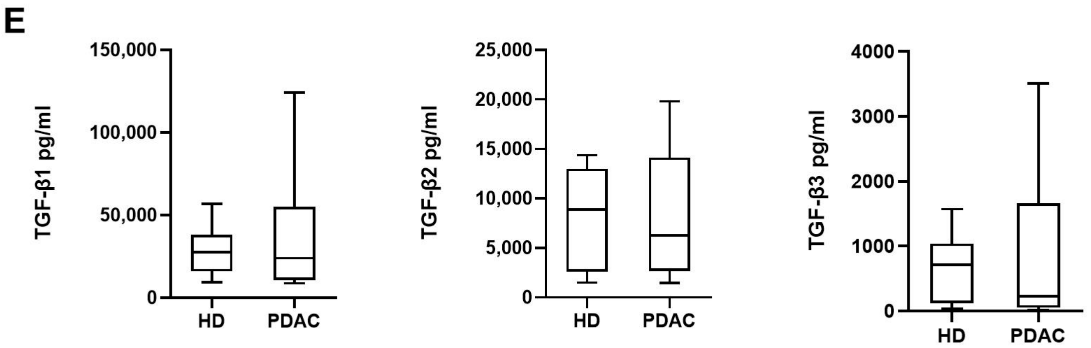
E
150,000
100,000
50,000
0
TGF-β1 pg/ml
HD
PDAC
25,000
20,000
15,000
10,000
5,000
0
TGF-β2 pg/ml
HD
PDAC
4000
3000
2000
1000
0
TGF-β3 pg/ml
HD
PDAC

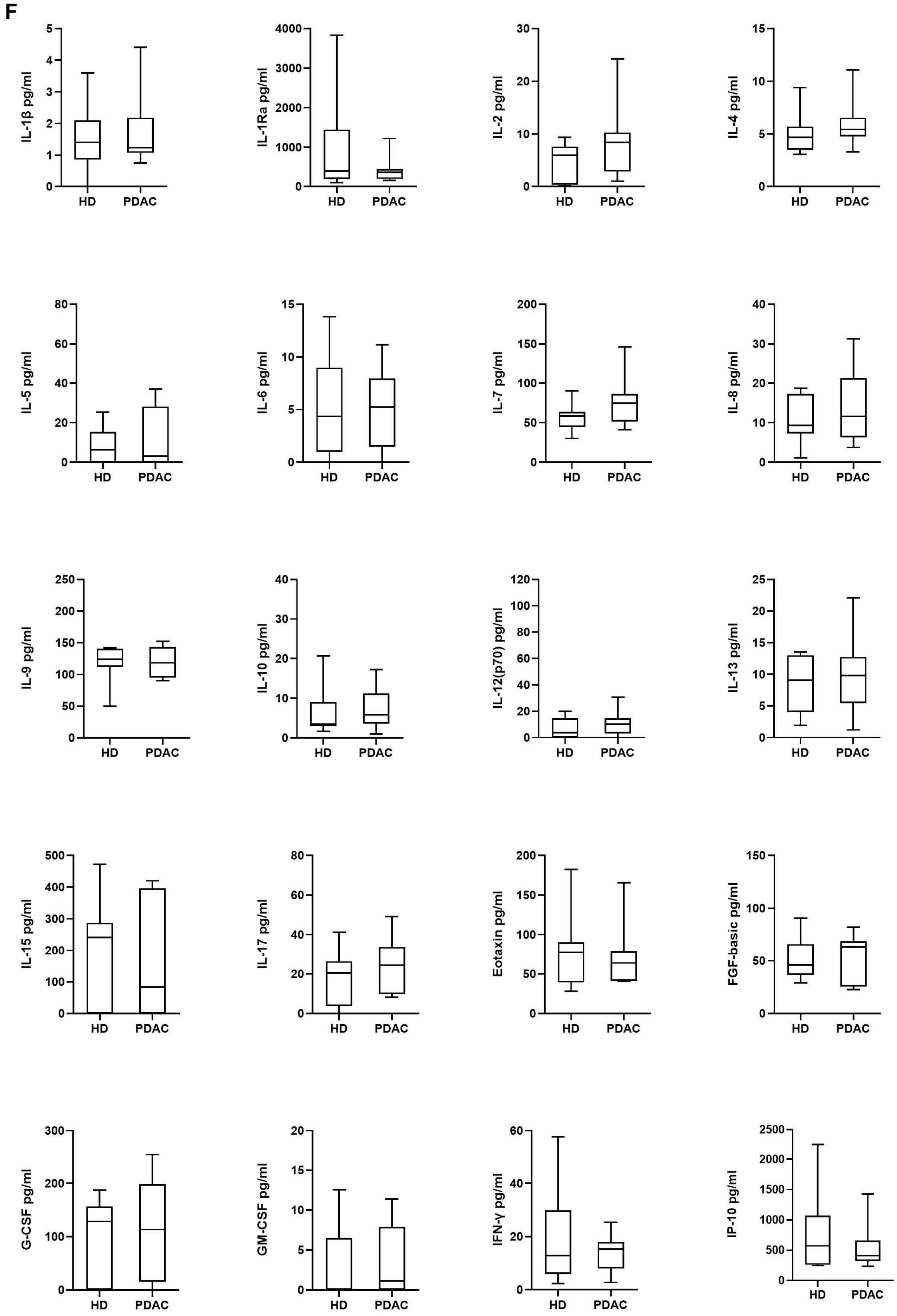
F
IL-1β pg/ml
IL-1Ra pg/ml
IL-2 pg/ml
IL-4 pg/ml
IL-5 pg/ml
IL-6 pg/ml
IL-7 pg/ml
IL-8 pg/ml
IL-9 pg/ml
IL-10 pg/ml
IL-12(p70) pg/ml
IL-13 pg/ml
IL-15 pg/ml
IL-17 pg/ml
Eotaxin pg/ml
FGF-basic pg/ml
G-CSF pg/ml
GM-CSF pg/ml
IFN-γ pg/ml
IP-10 pg/ml
HD
PDAC

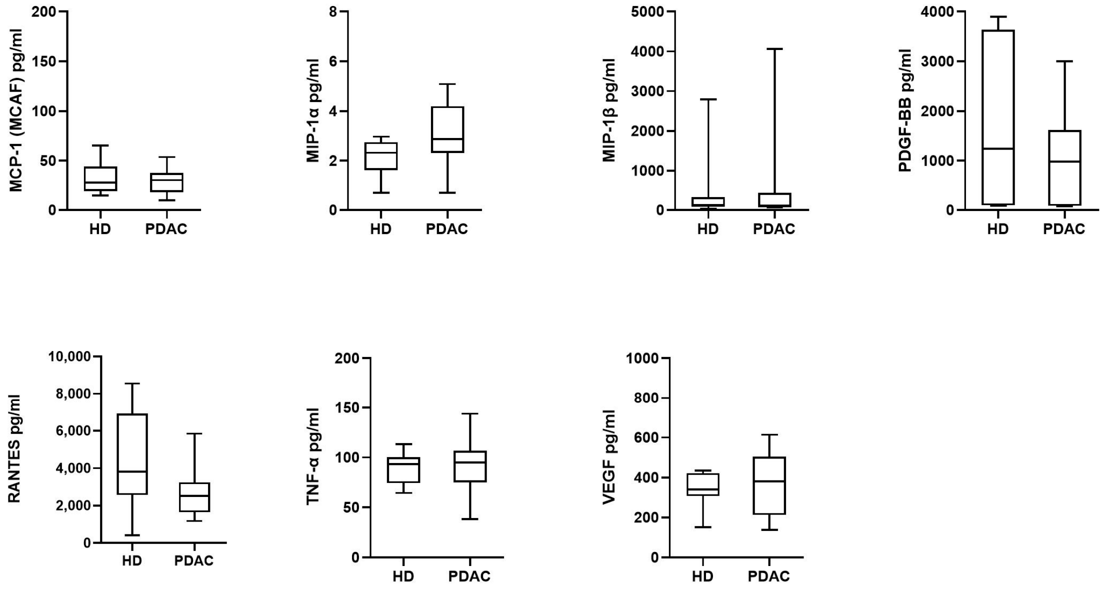

Footnote: Tfh: T follicular helper; SM: switched memory; Ab: antibody secreting; T1/2: type 1/2; T3a: type 3a; MZP: marginal zone peripheral; Breg: B regulatory.

Figure S5. Correlation studies between circulating immune cells (A: T-cell compartment; B: B-cell compartment; C: monocyte compartment) and concentrations of inflammatory and immune-related cytokines and chemokines (D: Pro Human Inflammation Panel I Assay; E: Pro TGF-β Immunoassay; F: Pro Human Cytokine Immunoassay) at baseline and progression-free survival (PFS) after therapy onset in patients with pancreatic adenocarcinoma (PDAC). Correlation studies were performed using the Spearman's rho rank correlation test.

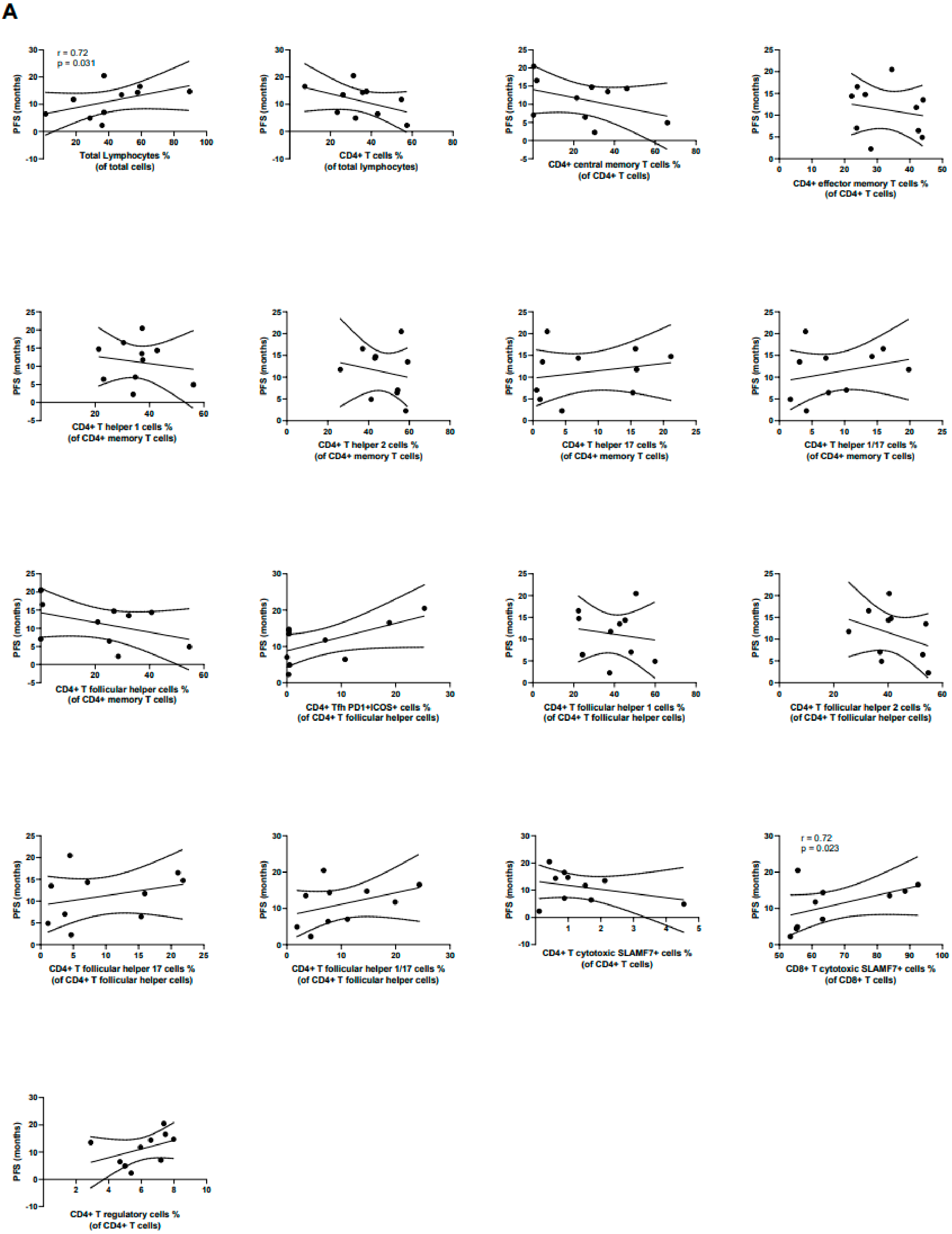
A
r = 0.72
p = 0.031
PFS (months)
Total Lymphocytes %
(of total cells)
CD4+ T cells %
(of total lymphocytes)
CD4+ central memory T cells %
(of CD4+ T cells)
CD4+ effector memory T cells %
(of CD4+ T cells)
CD4+ T helper 1 cells %
(of CD4+ memory T cells)
CD4+ T helper 2 cells %
(of CD4+ memory T cells)
CD4+ T helper 17 cells %
(of CD4+ memory T cells)
CD4+ T helper 1/17 cells %
(of CD4+ memory T cells)
CD4+ T follicular helper cells %
(of CD4+ memory T cells)
CD4+ Tfh PD1+ICOS+ cells %
(of CD4+ T follicular helper cells)
CD4+ T follicular helper 1 cells %
(of CD4+ T follicular helper cells)
CD4+ T follicular helper 2 cells %
(of CD4+ T follicular helper cells)
CD4+ T follicular helper 17 cells %
(of CD4+ T follicular helper cells)
CD4+ T follicular helper 1/17 cells %
(of CD4+ T follicular helper cells)
CD4+ T cytotoxic SLAMF7+ cells %
(of CD4+ T cells)
r = 0.72
p = 0.023
CD8+ T cytotoxic SLAMF7+ cells %
(of CD8+ T cells)
CD4+ T regulatory cells %
(of CD4+ T cells)

227

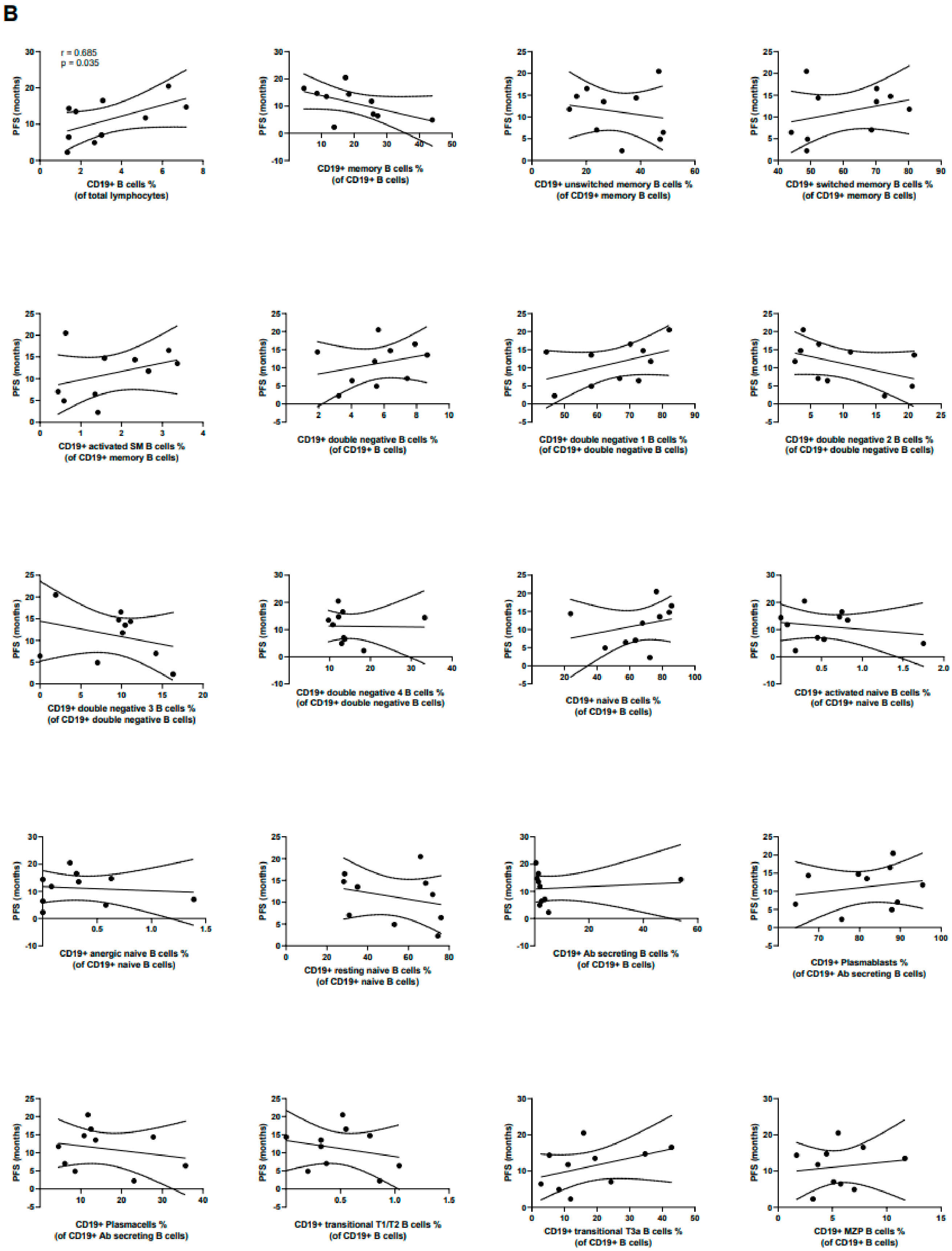
B
r = 0.685
p = 0.035
PFS (months)
CD19+ B cells %
(of total lymphocytes)
CD19+ memory B cells %
(of CD19+ B cells)
CD19+ unswitched memory B cells %
(of CD19+ memory B cells)
CD19+ switched memory B cells %
(of CD19+ memory B cells)
CD19+ activated SM B cells %
(of CD19+ memory B cells)
CD19+ double negative B cells %
(of CD19+ B cells)
CD19+ double negative 1 B cells %
(of CD19+ double negative B cells)
CD19+ double negative 2 B cells %
(of CD19+ double negative B cells)
CD19+ double negative 3 B cells %
(of CD19+ double negative B cells)
CD19+ double negative 4 B cells %
(of CD19+ double negative B cells)
CD19+ naive B cells %
(of CD19+ B cells)
CD19+ activated naive B cells %
(of CD19+ naive B cells)
CD19+ anergic naive B cells %
(of CD19+ naive B cells)
CD19+ resting naive B cells %
(of CD19+ naive B cells)
CD19+ Ab secreting B cells %
(of CD19+ B cells)
CD19+ Plasmablasts %
(of CD19+ Ab secreting B cells)
CD19+ Plasmacells %
(of CD19+ Ab secreting B cells)
CD19+ transitional T1/T2 B cells %
(of CD19+ B cells)
CD19+ transitional T3a B cells %
(of CD19+ B cells)
CD19+ MZP B cells %
(of CD19+ B cells)

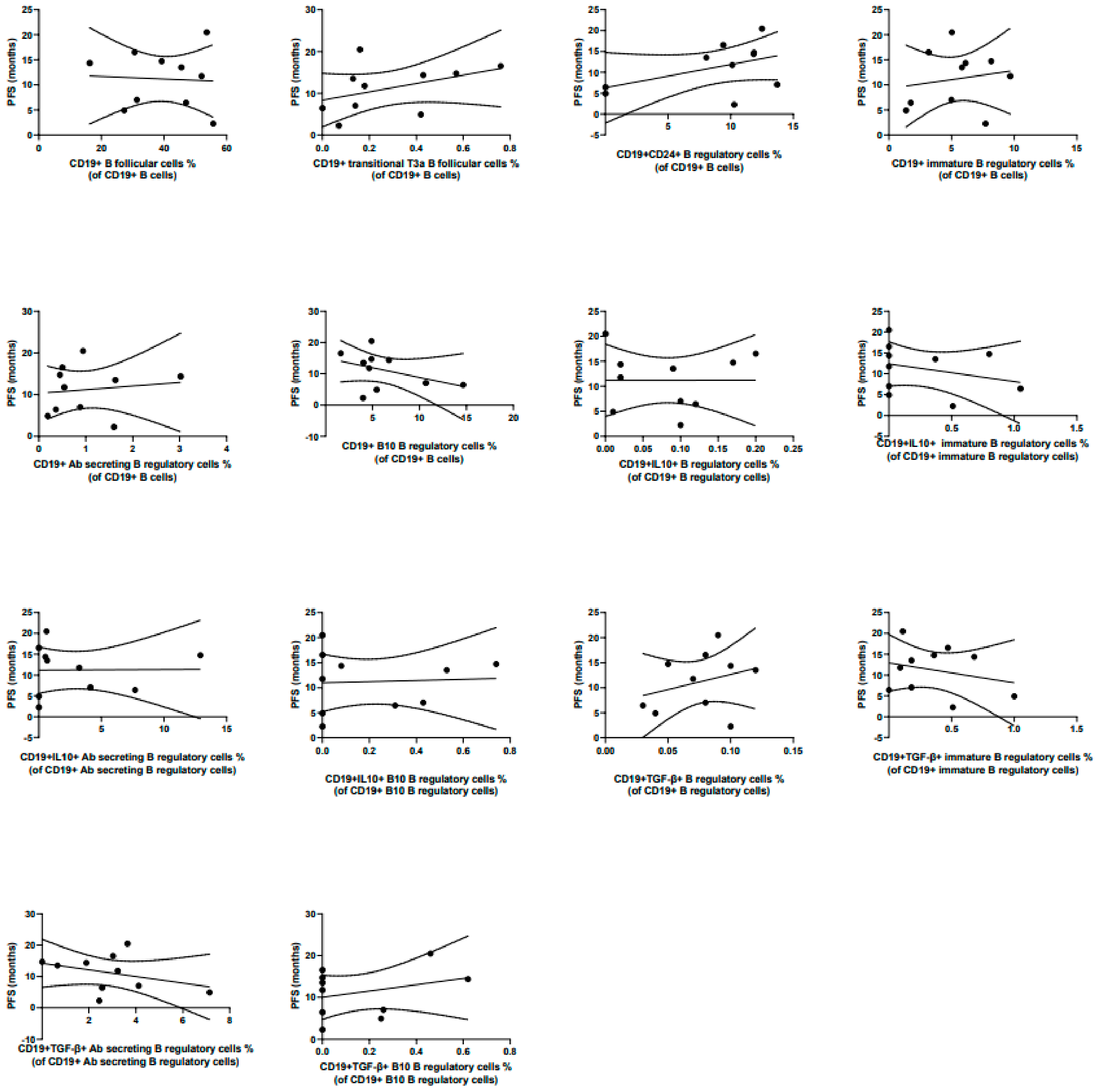
PFS (months)
CD19+ B follicular cells % (of CD19+ B cells)
CD19+ transitional T3a B follicular cells % (of CD19+ B cells)
CD19+CD24+ B regulatory cells % (of CD19+ B cells)
CD19+ immature B regulatory cells % (of CD19+ B cells)
CD19+ Ab secreting B regulatory cells % (of CD19+ B cells)
CD19+ B10 B regulatory cells % (of CD19+ B cells)
CD19+IL10+ B regulatory cells % (of CD19+ B regulatory cells)
CD19+IL10+ immature B regulatory cells % (of CD19+ immature B regulatory cells)
CD19+IL10+ Ab secreting B regulatory cells % (of CD19+ Ab secreting B regulatory cells)
CD19+IL10+ B10 B regulatory cells % (of CD19+ B10 B regulatory cells)
CD19+TGF-β+ B regulatory cells % (of CD19+ B regulatory cells)
CD19+TGF-β+ immature B regulatory cells % (of CD19+ immature B regulatory cells)
CD19+TGF-β+ Ab secreting B regulatory cells % (of CD19+ Ab secreting B regulatory cells)
CD19+TGF-β+ B10 B regulatory cells % (of CD19+ B10 B regulatory cells)

C

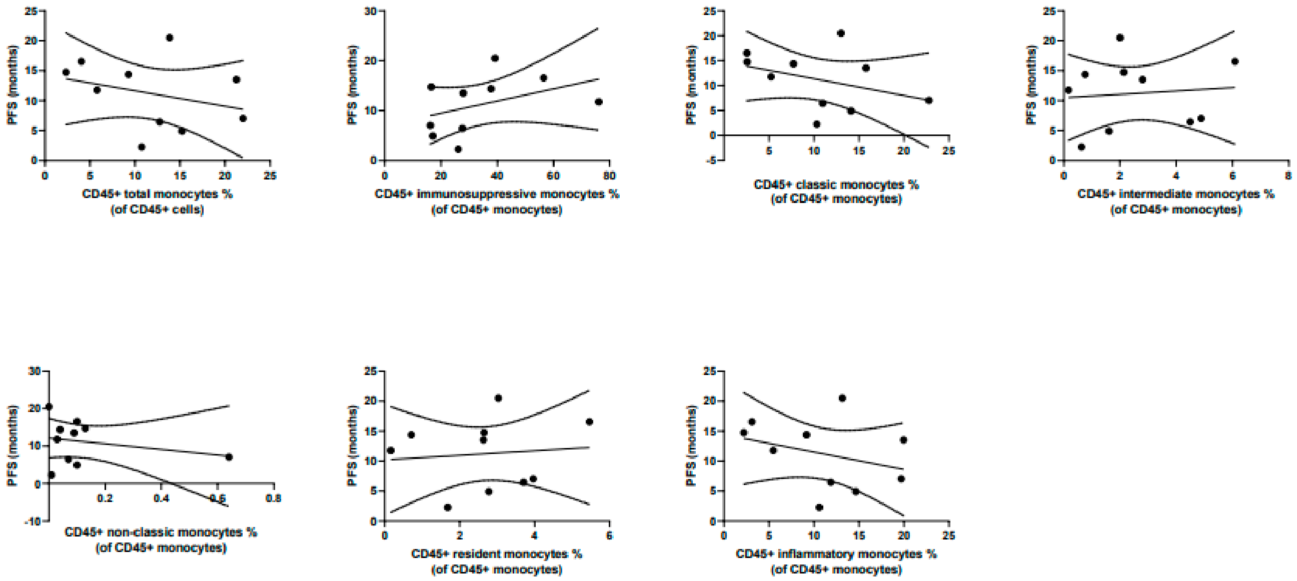
PFS (months)
CD45+ total monocytes %
(of CD45+ cells)
CD45+ immunosuppressive monocytes %
(of CD45+ monocytes)
CD45+ classic monocytes %
(of CD45+ monocytes)
CD45+ intermediate monocytes %
(of CD45+ monocytes)
CD45+ non-classic monocytes %
(of CD45+ monocytes)
CD45+ resident monocytes %
(of CD45+ monocytes)
CD45+ inflammatory monocytes %
(of CD45+ monocytes)

D

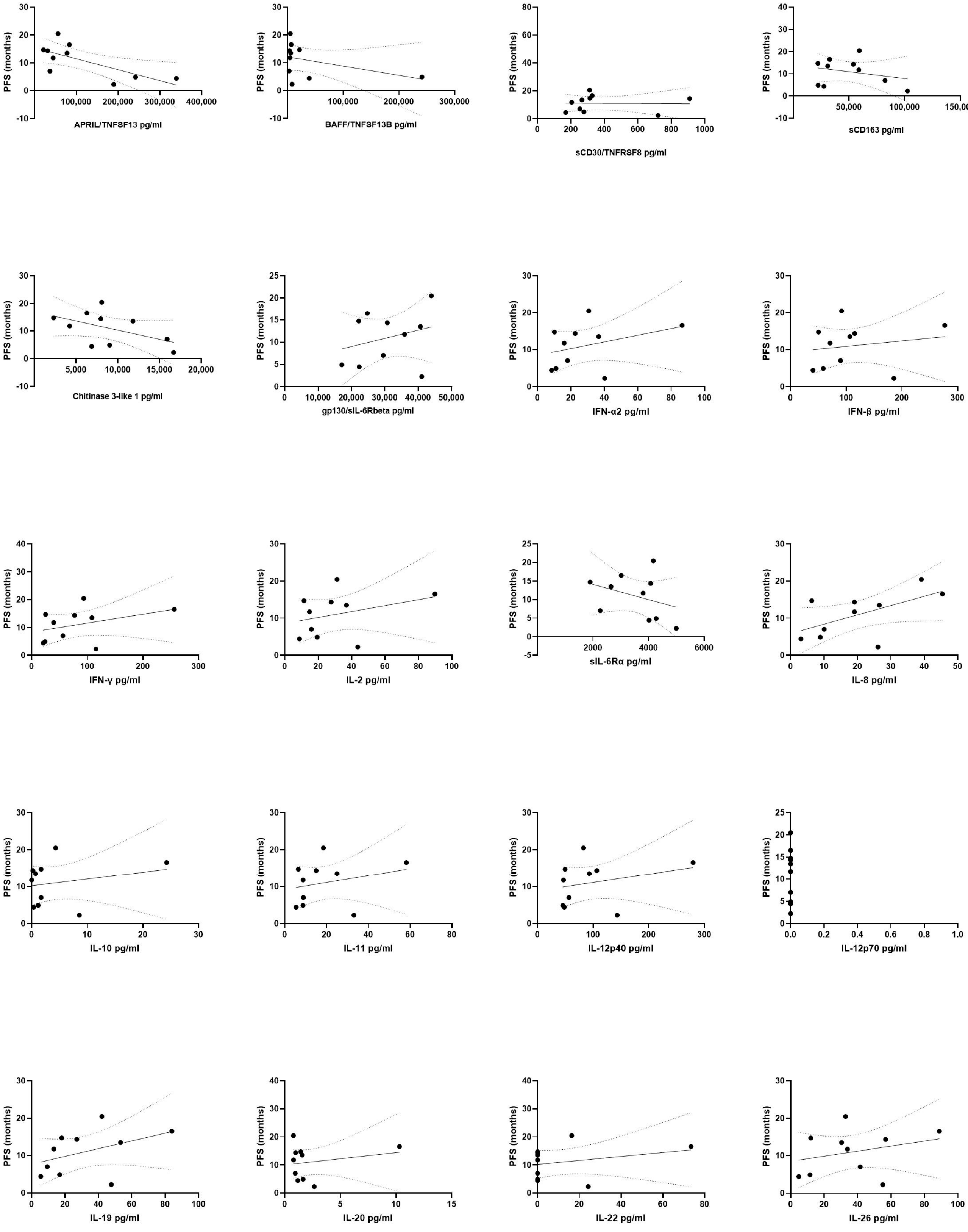
PFS (months)
APRIL/TNFSF13 pg/ml
BAFF/TNFSF13B pg/ml
sCD30/TNFRSF8 pg/ml
sCD163 pg/ml
Chitinase 3-like 1 pg/ml
gp130/sIL-6Rbeta pg/ml
IFN-α2 pg/ml
IFN-β pg/ml
IFN-γ pg/ml
IL-2 pg/ml
sIL-6Rα pg/ml
IL-8 pg/ml
IL-10 pg/ml
IL-11 pg/ml
IL-12p40 pg/ml
IL-12p70 pg/ml
IL-19 pg/ml
IL-20 pg/ml
IL-22 pg/ml
IL-26 pg/ml

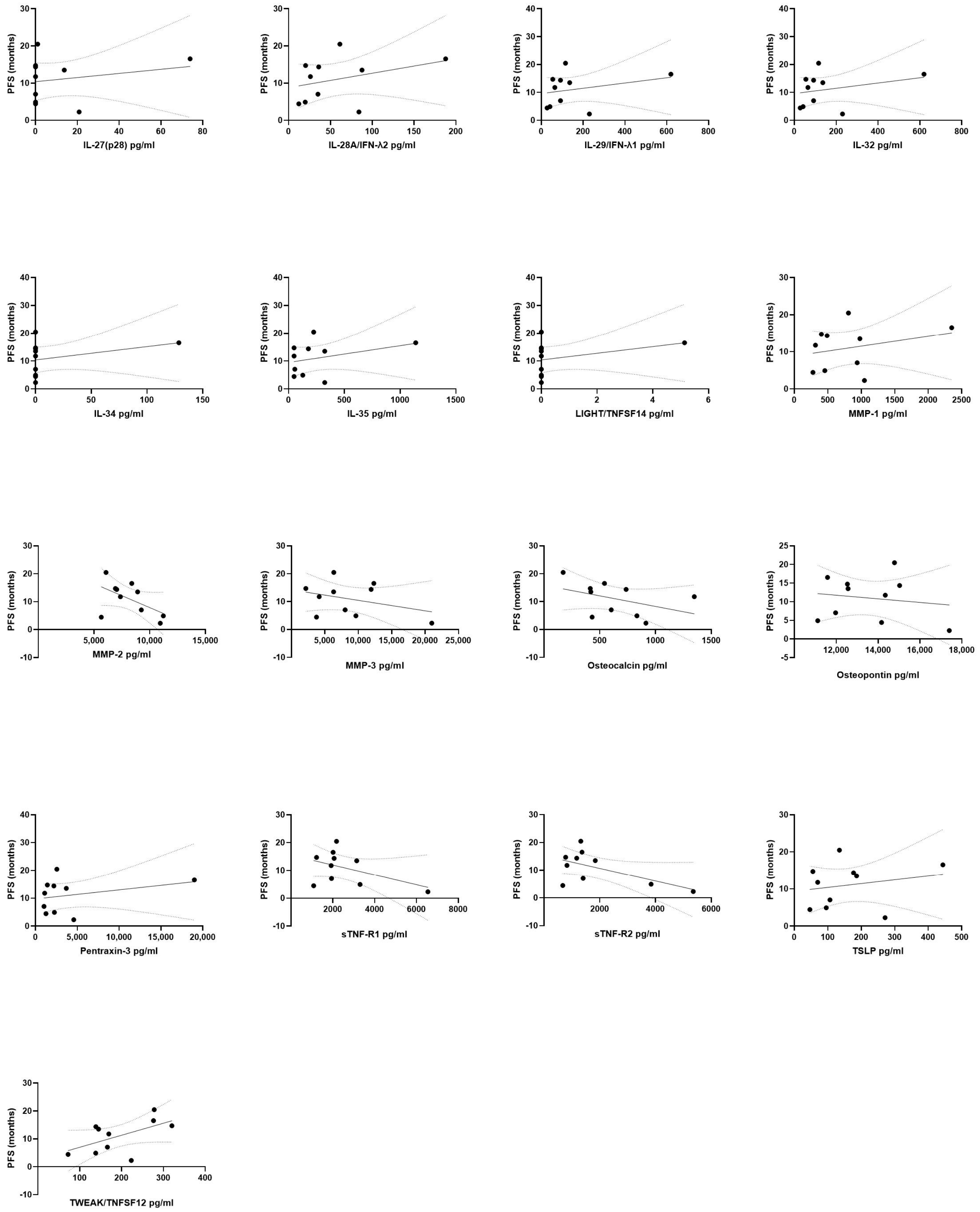
PFS (months)
IL-27(p28) pg/ml
IL-28A/IFN-λ2 pg/ml
IL-29/IFN-λ1 pg/ml
IL-32 pg/ml
IL-34 pg/ml
IL-35 pg/ml
LIGHT/TNFSF14 pg/ml
MMP-1 pg/ml
MMP-2 pg/ml
MMP-3 pg/ml
Osteocalcin pg/ml
Osteopontin pg/ml
Pentraxin-3 pg/ml
sTNF-R1 pg/ml
sTNF-R2 pg/ml
TSLP pg/ml
TWEAK/TNFSF12 pg/ml

E

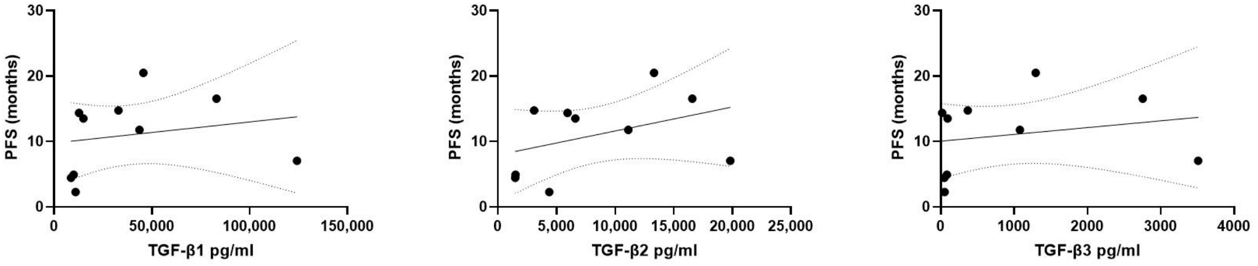

30
20
10
0
PFS (months)
0
50,000
100,000
150,000
TGF-β1 pg/ml
30
20
10
0
PFS (months)
0
5,000
10,000
15,000
20,000
25,000
TGF-β2 pg/ml
30
20
10
0
PFS (months)
0
1000
2000
3000
4000
TGF-β3 pg/ml

239

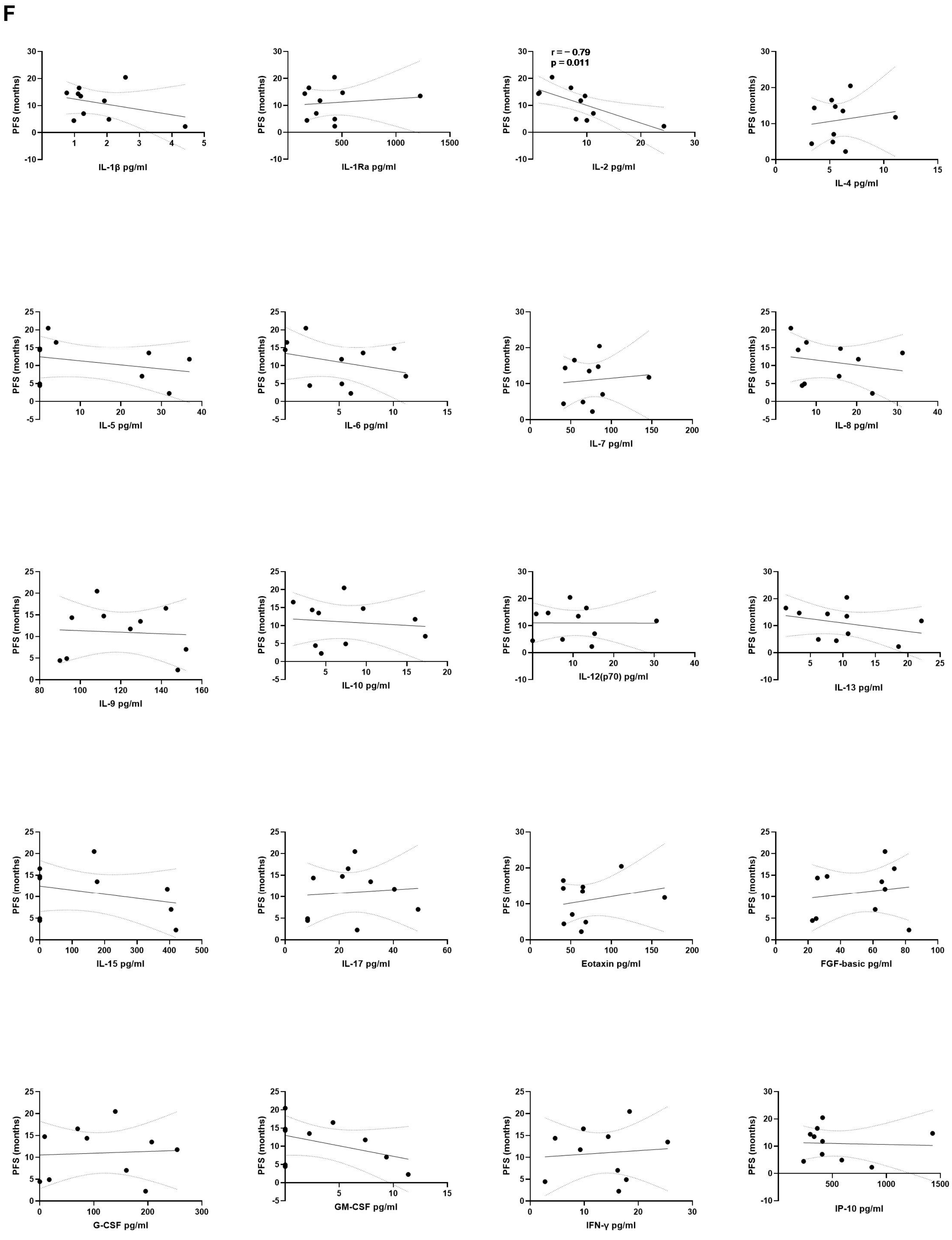
F
PFS (months)
IL-1β pg/ml
IL-1Ra pg/ml
r = − 0.79
p = 0.011
IL-2 pg/ml
IL-4 pg/ml
IL-5 pg/ml
IL-6 pg/ml
IL-7 pg/ml
IL-8 pg/ml
IL-9 pg/ml
IL-10 pg/ml
IL-12(p70) pg/ml
IL-13 pg/ml
IL-15 pg/ml
IL-17 pg/ml
Eotaxin pg/ml
FGF-basic pg/ml
G-CSF pg/ml
GM-CSF pg/ml
IFN-γ pg/ml
IP-10 pg/ml

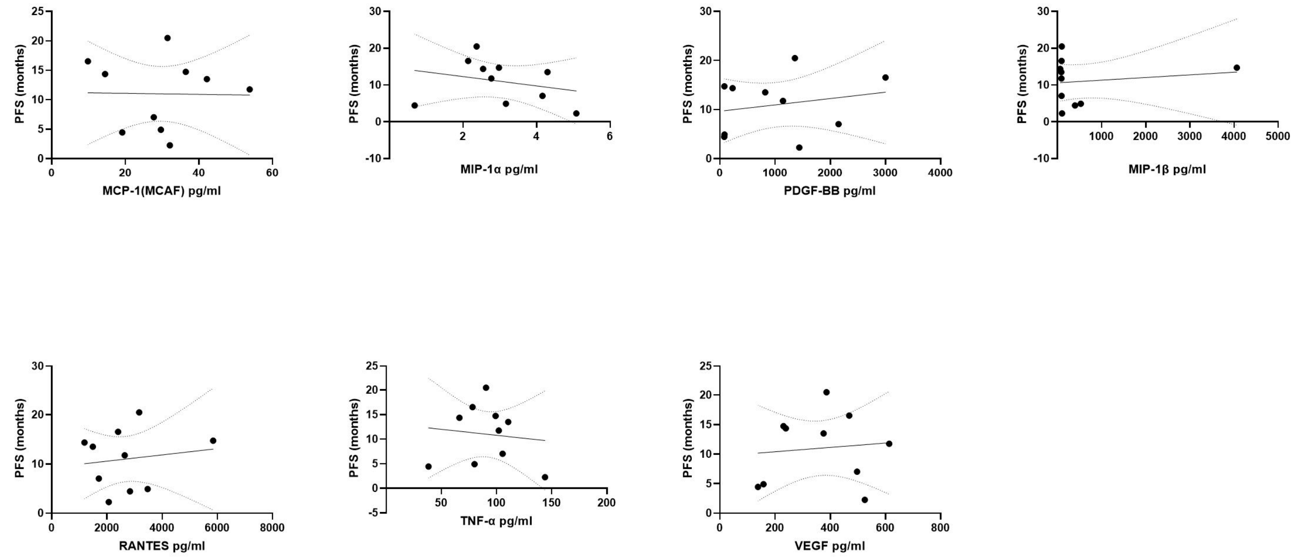

Footnote: Te: T effector; Th1: T helper 1; Th2: T helper 2; Th17: T helper 17; Th1/17: T helper 1/17; Tfh: T follicular helper; Tfh1: T follicular helper 1; Tfh2: T follicular helper 2; Tfh17: T follicular helper 17; Tfh1/17: T follicular helper 1/17; Treg: T regulatory; UM: unswitched memory; SM: switched memory; ASM: activated switched memory; DN: double negative; DN1: double negative 1; DN2: double negative 2; DN3: double negative 3; DN4: double negative 4; AcN: activated naïve; AnN: anergic naïve; RN: resting naïve; Ab: antibody secreting; T1/2: transitional 1/2; T3a: transitional 3a; MZP: marginal zone peripheral; Breg: B regulatory; AbS: antibody secreting; Imm: immature; Mon: monocytes.

Figure S6. Correlation studies between circulating immune cells (A: T-cell compartment; B: B-cell compartment; C: monocyte compartment) and concentrations of inflammatory and immune-related cytokines and chemokines (D: Pro Human Inflammation Panel I Assay; E: Pro TGF-β Immunoassay; F: Pro Human Cytokine Immunoassay) at baseline and overall survival (OS) after therapy onset in patients with pancreatic adenocarcinoma (PDAC). Correlation studies were performed using the Spearman's rho rank correlation test.

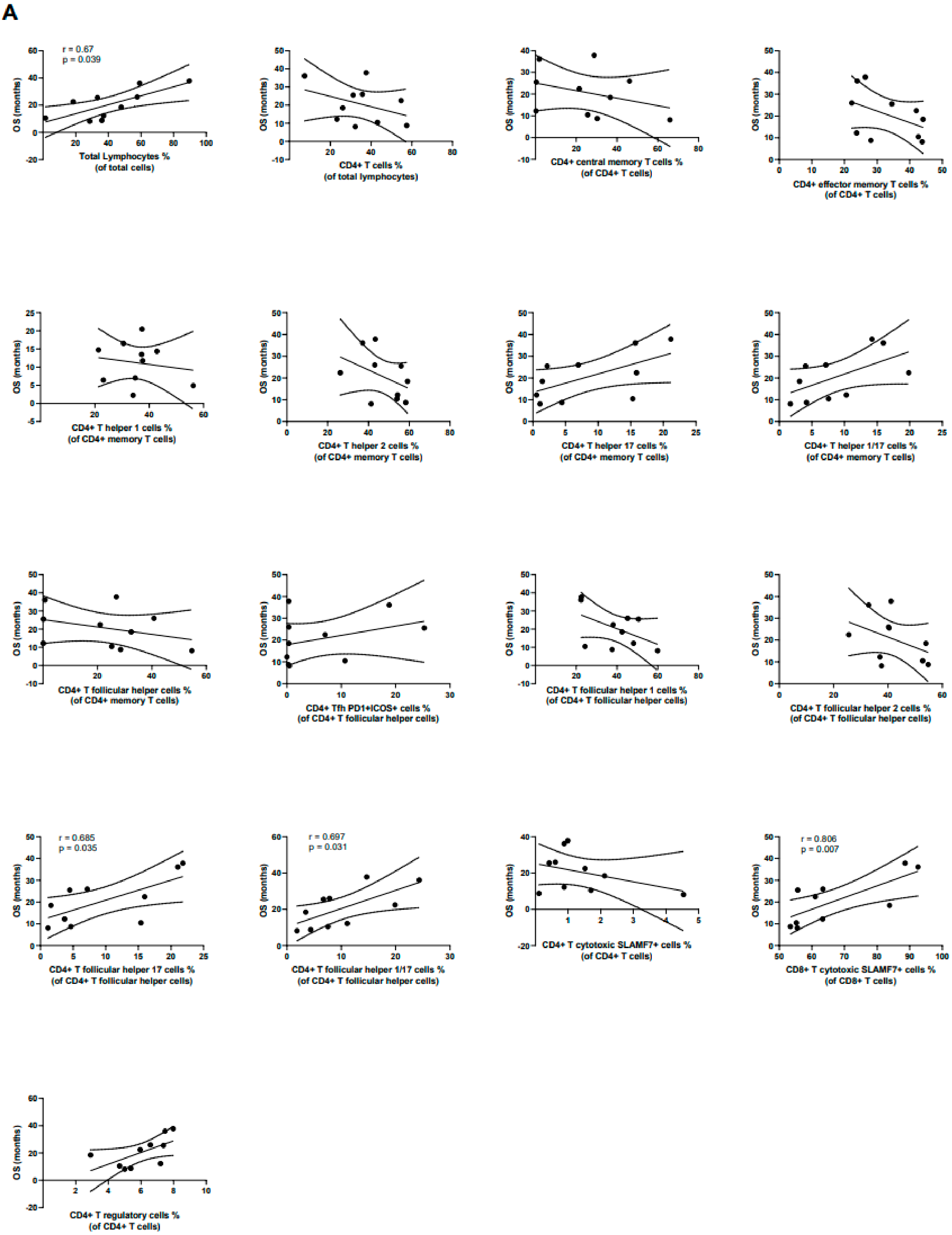
A
r = 0.67
p = 0.039
OS (months)
Total Lymphocytes %
(of total cells)
CD4+ T cells %
(of total lymphocytes)
CD4+ central memory T cells %
(of CD4+ T cells)
CD4+ effector memory T cells %
(of CD4+ T cells)
CD4+ T helper 1 cells %
(of CD4+ memory T cells)
CD4+ T helper 2 cells %
(of CD4+ memory T cells)
CD4+ T helper 17 cells %
(of CD4+ memory T cells)
CD4+ T helper 1/17 cells %
(of CD4+ memory T cells)
CD4+ T follicular helper cells %
(of CD4+ memory T cells)
CD4+ Tfh PD1+ICOS+ cells %
(of CD4+ T follicular helper cells)
CD4+ T follicular helper 1 cells %
(of CD4+ T follicular helper cells)
CD4+ T follicular helper 2 cells %
(of CD4+ T follicular helper cells)
r = 0.685
p = 0.035
CD4+ T follicular helper 17 cells %
(of CD4+ T follicular helper cells)
r = 0.697
p = 0.031
CD4+ T follicular helper 1/17 cells %
(of CD4+ T follicular helper cells)
CD4+ T cytotoxic SLAMF7+ cells %
(of CD4+ T cells)
r = 0.806
p = 0.007
CD8+ T cytotoxic SLAMF7+ cells %
(of CD8+ T cells)
CD4+ T regulatory cells %
(of CD4+ T cells)

B

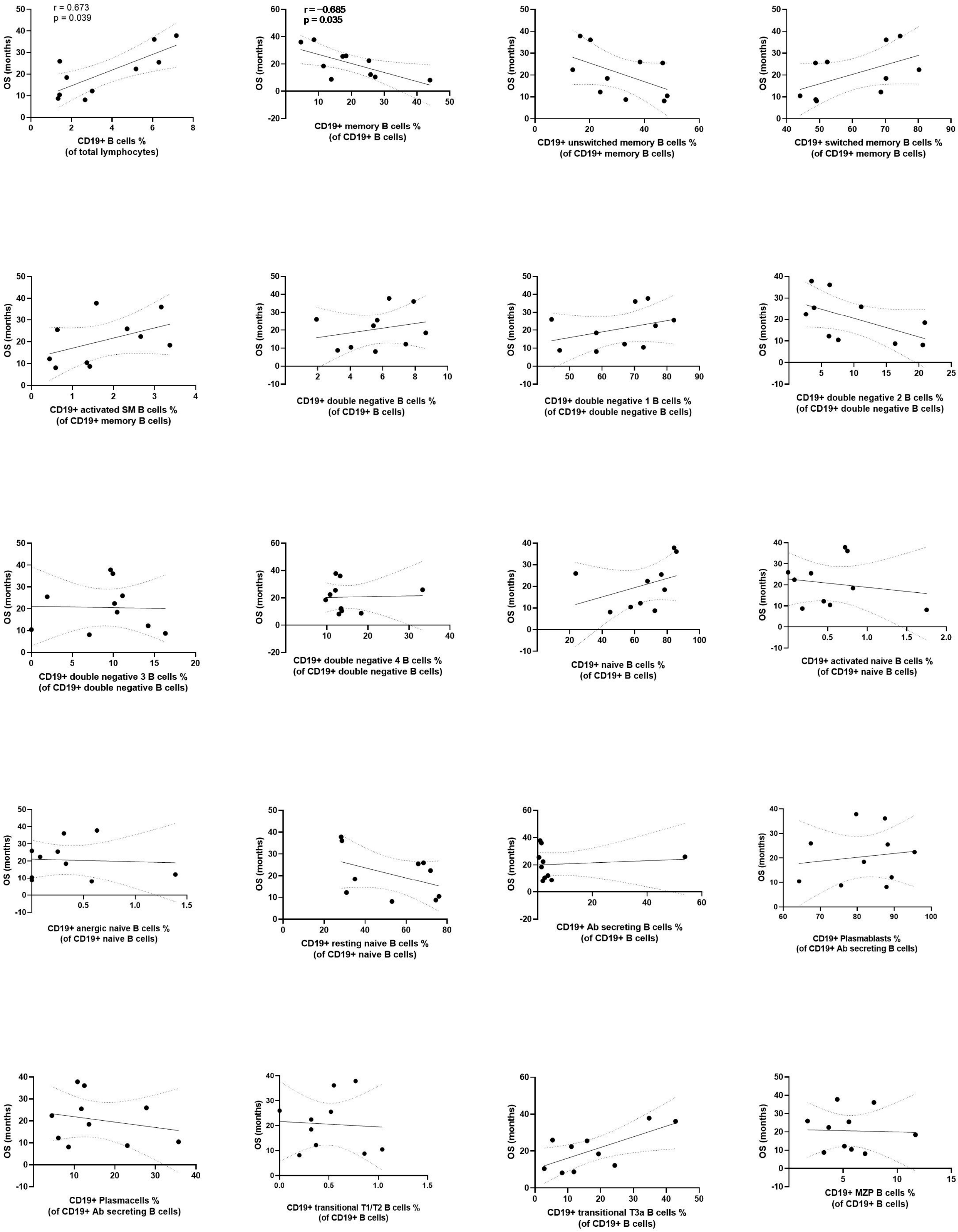
r = 0.673
p = 0.039
OS (months)
CD19+ B cells %
(of total lymphocytes)
r = −0.685
p = 0.035
CD19+ memory B cells %
(of CD19+ B cells)
CD19+ unswitched memory B cells %
(of CD19+ memory B cells)
CD19+ switched memory B cells %
(of CD19+ memory B cells)
CD19+ activated SM B cells %
(of CD19+ memory B cells)
CD19+ double negative B cells %
(of CD19+ B cells)
CD19+ double negative 1 B cells %
(of CD19+ double negative B cells)
CD19+ double negative 2 B cells %
(of CD19+ double negative B cells)
CD19+ double negative 3 B cells %
(of CD19+ double negative B cells)
CD19+ double negative 4 B cells %
(of CD19+ double negative B cells)
CD19+ naive B cells %
(of CD19+ B cells)
CD19+ activated naive B cells %
(of CD19+ naive B cells)
CD19+ anergic naive B cells %
(of CD19+ naive B cells)
CD19+ resting naive B cells %
(of CD19+ naive B cells)
CD19+ Ab secreting B cells %
(of CD19+ B cells)
CD19+ Plasmablasts %
(of CD19+ Ab secreting B cells)
CD19+ Plasmacells %
(of CD19+ Ab secreting B cells)
CD19+ transitional T1/T2 B cells %
(of CD19+ B cells)
CD19+ transitional T3a B cells %
(of CD19+ B cells)
CD19+ MZP B cells %
(of CD19+ B cells)

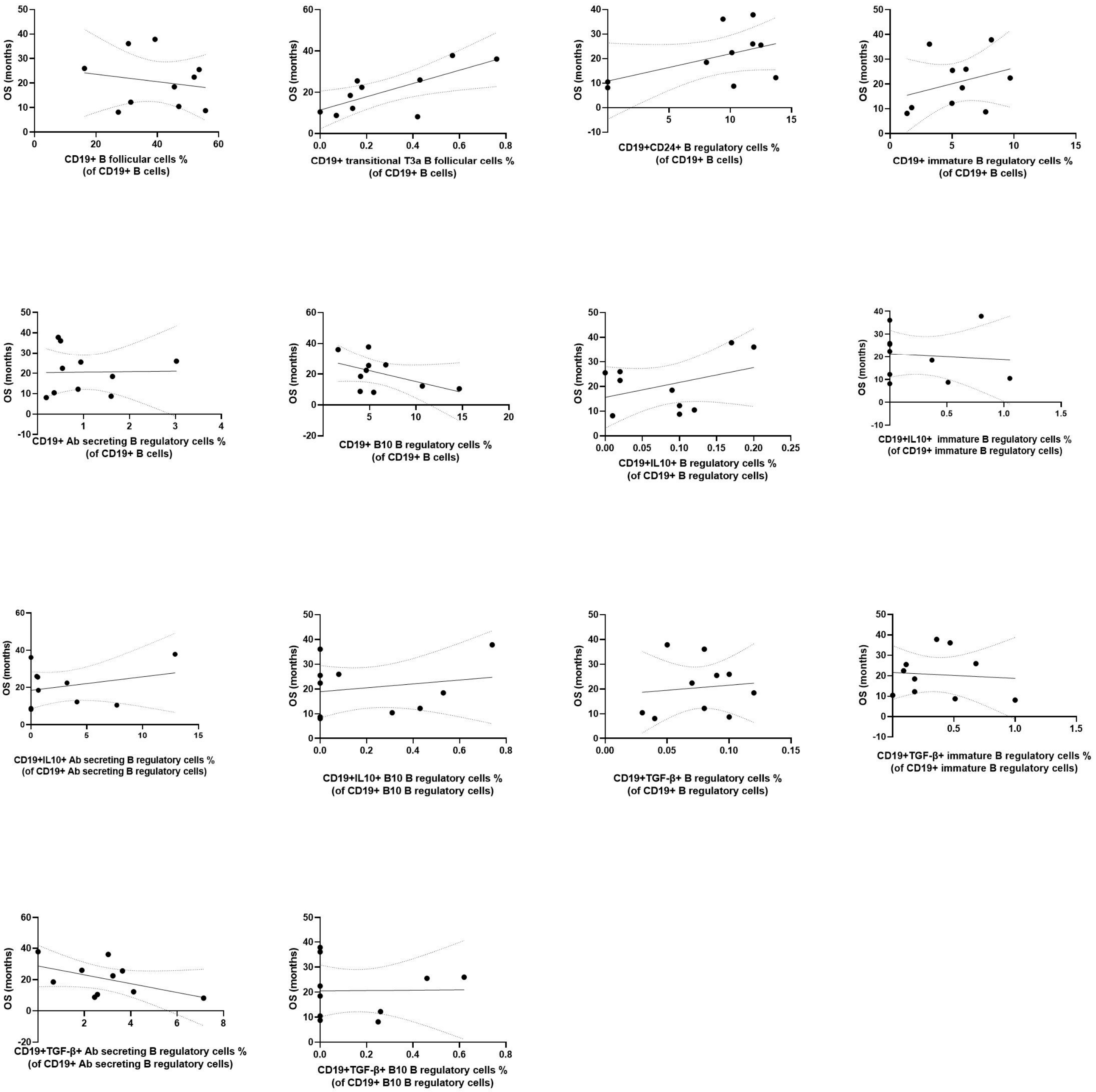
OS (months)
CD19+ B follicular cells %
(of CD19+ B cells)
CD19+ transitional T3a B follicular cells %
(of CD19+ B cells)
CD19+CD24+ B regulatory cells %
(of CD19+ B cells)
CD19+ immature B regulatory cells %
(of CD19+ B cells)
CD19+ Ab secreting B regulatory cells %
(of CD19+ B cells)
CD19+ B10 B regulatory cells %
(of CD19+ B cells)
CD19+IL10+ B regulatory cells %
(of CD19+ B regulatory cells)
CD19+IL10+ immature B regulatory cells %
(of CD19+ immature B regulatory cells)
CD19+IL10+ Ab secreting B regulatory cells %
(of CD19+ Ab secreting B regulatory cells)
CD19+IL10+ B10 B regulatory cells %
(of CD19+ B10 B regulatory cells)
CD19+TGF-β+ B regulatory cells %
(of CD19+ B regulatory cells)
CD19+TGF-β+ immature B regulatory cells %
(of CD19+ immature B regulatory cells)
CD19+TGF-β+ Ab secreting B regulatory cells %
(of CD19+ Ab secreting B regulatory cells)
CD19+TGF-β+ B10 B regulatory cells %
(of CD19+ B10 B regulatory cells)

C

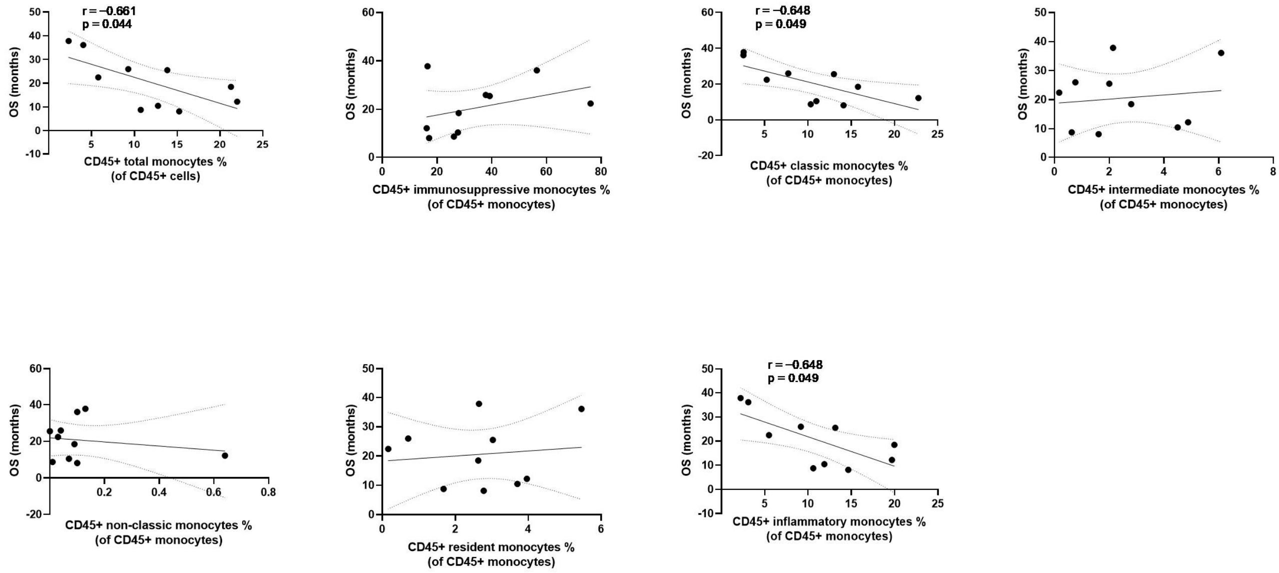
r = −0.661
p = 0.044
OS (months)
CD45+ total monocytes %
(of CD45+ cells)
OS (months)
CD45+ immunosuppressive monocytes %
(of CD45+ monocytes)
r = −0.648
p = 0.049
OS (months)
CD45+ classic monocytes %
(of CD45+ monocytes)
OS (months)
CD45+ intermediate monocytes %
(of CD45+ monocytes)
OS (months)
CD45+ non-classic monocytes %
(of CD45+ monocytes)
OS (months)
CD45+ resident monocytes %
(of CD45+ monocytes)
r = −0.648
p = 0.049
OS (months)
CD45+ inflammatory monocytes %
(of CD45+ monocytes)

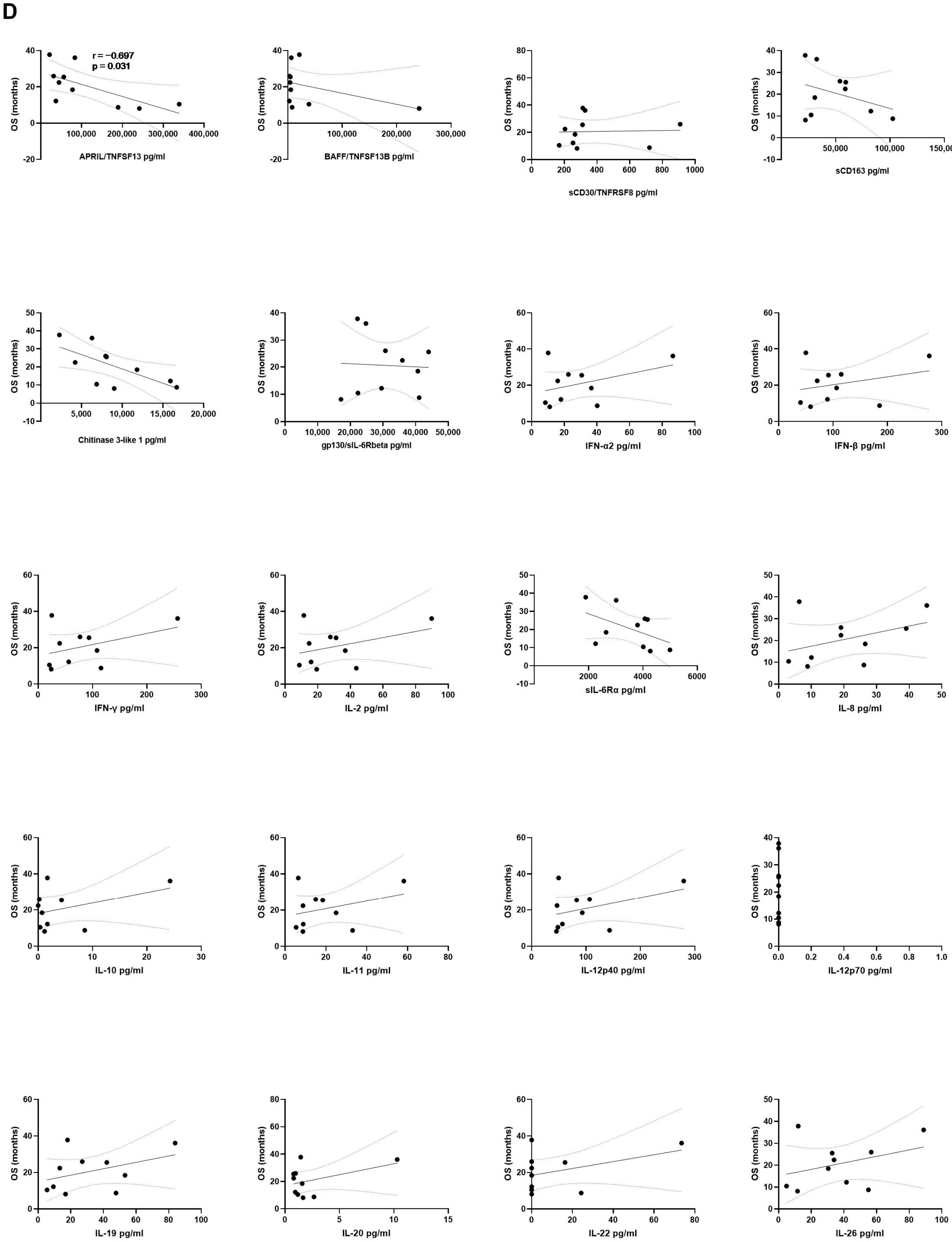
D
r = −0.697
p = 0.031
OS (months)
APRIL/TNFSF13 pg/ml
BAFF/TNFSF13B pg/ml
sCD30/TNFRSF8 pg/ml
sCD163 pg/ml
Chitinase 3-like 1 pg/ml
gp130/sIL-6Rbeta pg/ml
IFN-α2 pg/ml
IFN-β pg/ml
IFN-γ pg/ml
IL-2 pg/ml
sIL-6Rα pg/ml
IL-8 pg/ml
IL-10 pg/ml
IL-11 pg/ml
IL-12p40 pg/ml
IL-12p70 pg/ml
IL-19 pg/ml
IL-20 pg/ml
IL-22 pg/ml
IL-26 pg/ml

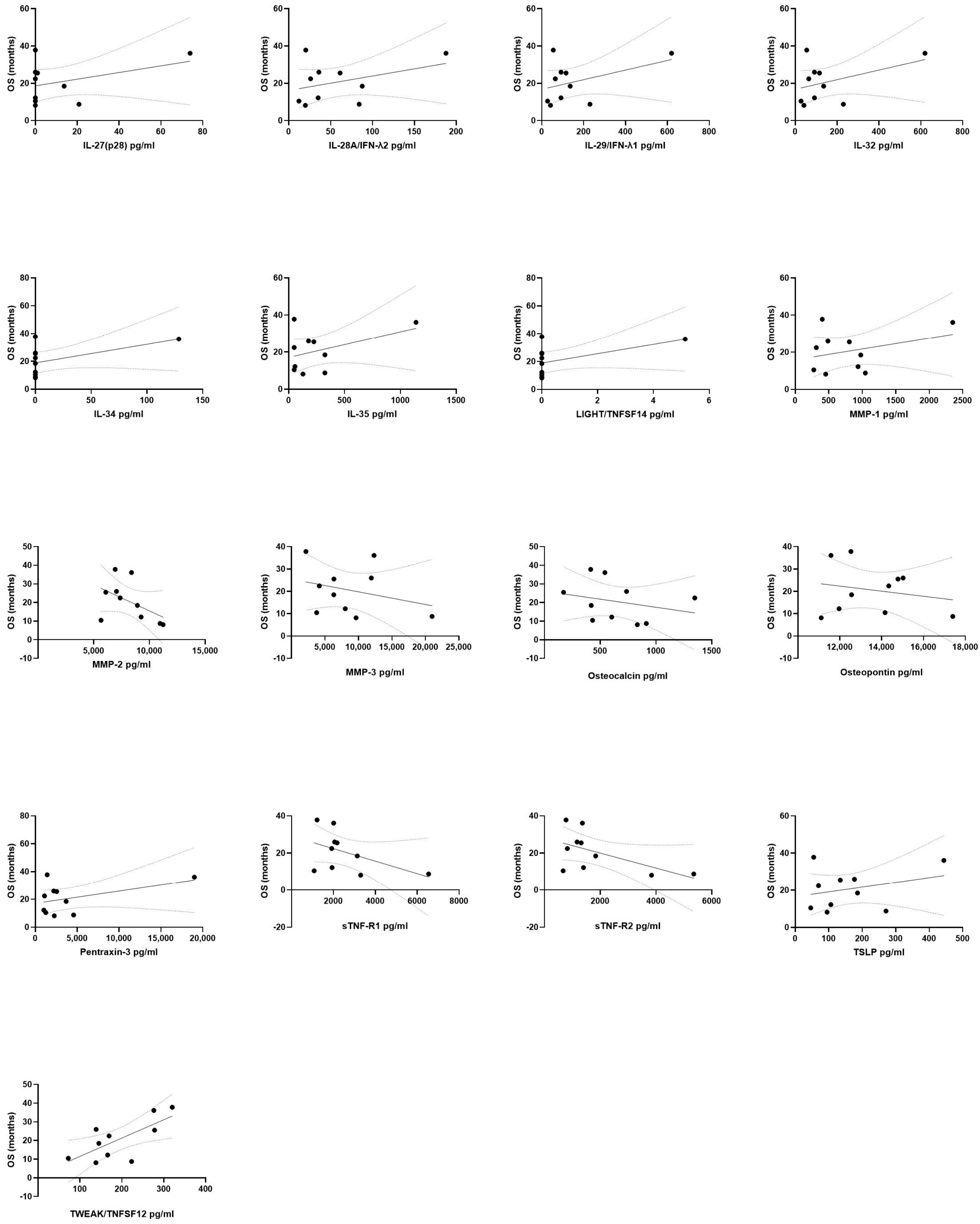
OS (months)
IL-27(p28) pg/ml
IL-28A/IFN-λ2 pg/ml
IL-29/IFN-λ1 pg/ml
IL-32 pg/ml
IL-34 pg/ml
IL-35 pg/ml
LIGHT/TNFSF14 pg/ml
MMP-1 pg/ml
MMP-2 pg/ml
MMP-3 pg/ml
Osteocalcin pg/ml
Osteopontin pg/ml
Pentraxin-3 pg/ml
sTNF-R1 pg/ml
sTNF-R2 pg/ml
TSLP pg/ml
TWEAK/TNFSF12 pg/ml

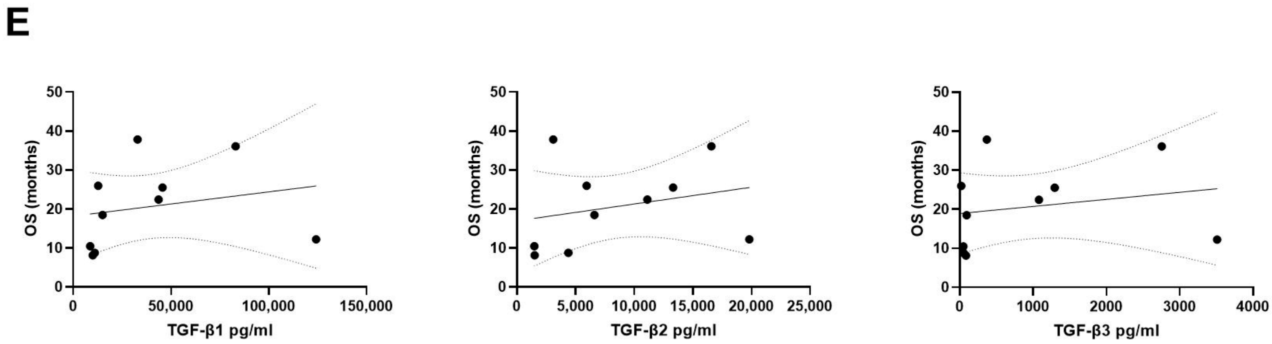
E
50
40
30
20
10
0
OS (months)
0
50,000
100,000
150,000
TGF-β1 pg/ml
0
5,000
10,000
15,000
20,000
25,000
TGF-β2 pg/ml
0
1000
2000
3000
4000
TGF-β3 pg/ml

279

F

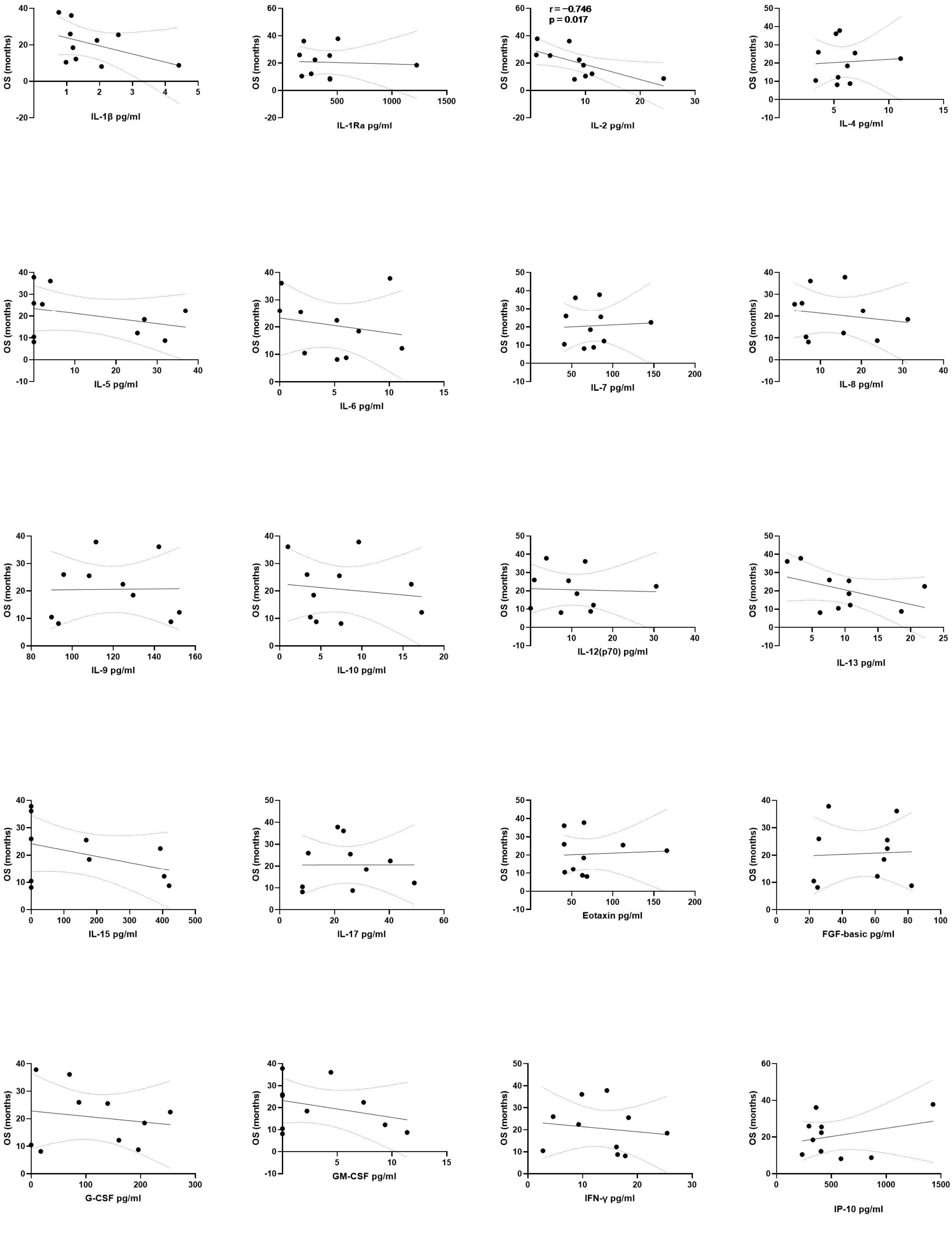
OS (months)
r = −0.746
p = 0.017
IL-1β pg/ml
IL-1Ra pg/ml
IL-2 pg/ml
IL-4 pg/ml
IL-5 pg/ml
IL-6 pg/ml
IL-7 pg/ml
IL-8 pg/ml
IL-9 pg/ml
IL-10 pg/ml
IL-12(p70) pg/ml
IL-13 pg/ml
IL-15 pg/ml
IL-17 pg/ml
Eotaxin pg/ml
FGF-basic pg/ml
G-CSF pg/ml
GM-CSF pg/ml
IFN-γ pg/ml
IP-10 pg/ml

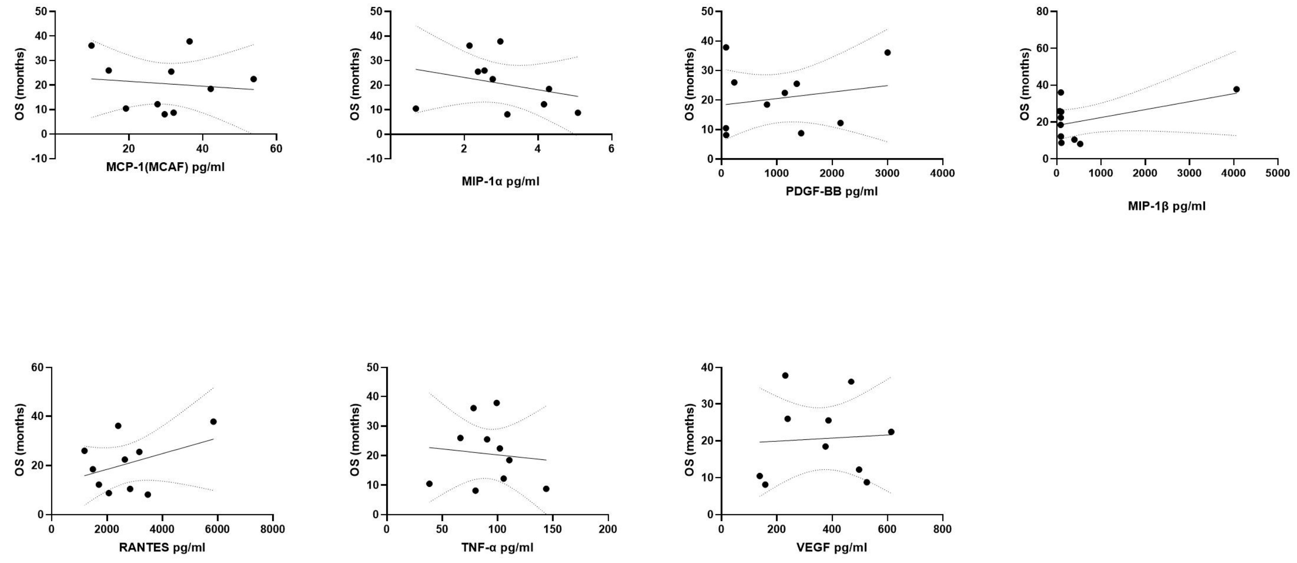

Footnote: Te: T effector; Th1: T helper 1; Th2: T helper 2; Th17: T helper 17; Th1/17: T helper 1/17; Tfh: T follicular helper; Tfh1: T follicular helper 1; Tfh2: T follicular helper 2; Tfh17: T follicular helper 17; Tfh1/17: T follicular helper 1/17; Treg: T regulatory; UM: unswitched memory; SM: switched memory; ASM: activated switched memory; DN: double negative; DN1: double negative 1; DN2: double negative 2; DN3: double negative 3; DN4: double negative 4; AcN: activated naïve; AnN: anergic naïve; RN: resting naïve; Ab: antibody secreting; T1/2: transitional 1/2; T3a: transitional 3a; MZP: marginal zone peripheral; Breg: B regulatory; AbS: antibody secreting; Imm: immature; Mon: monocytes.

Figure S7. Correlation studies between circulating immune cells (A: T-cell compartment; B: B-cell compartment; C: monocyte compartment) and concentrations of inflammatory and immune-related cytokines and chemokines (D: Pro Human Inflammation Panel I Assay; E: Pro TGF-β Immunoassay; F: Pro Human Cytokine Immunoassay) at 4-month follow-up and progression-free survival (PFS) after therapy onset in patients with pancreatic adenocarcinoma (PDAC). Correlation studies were performed using the Spearman's rho rank correlation test.

A

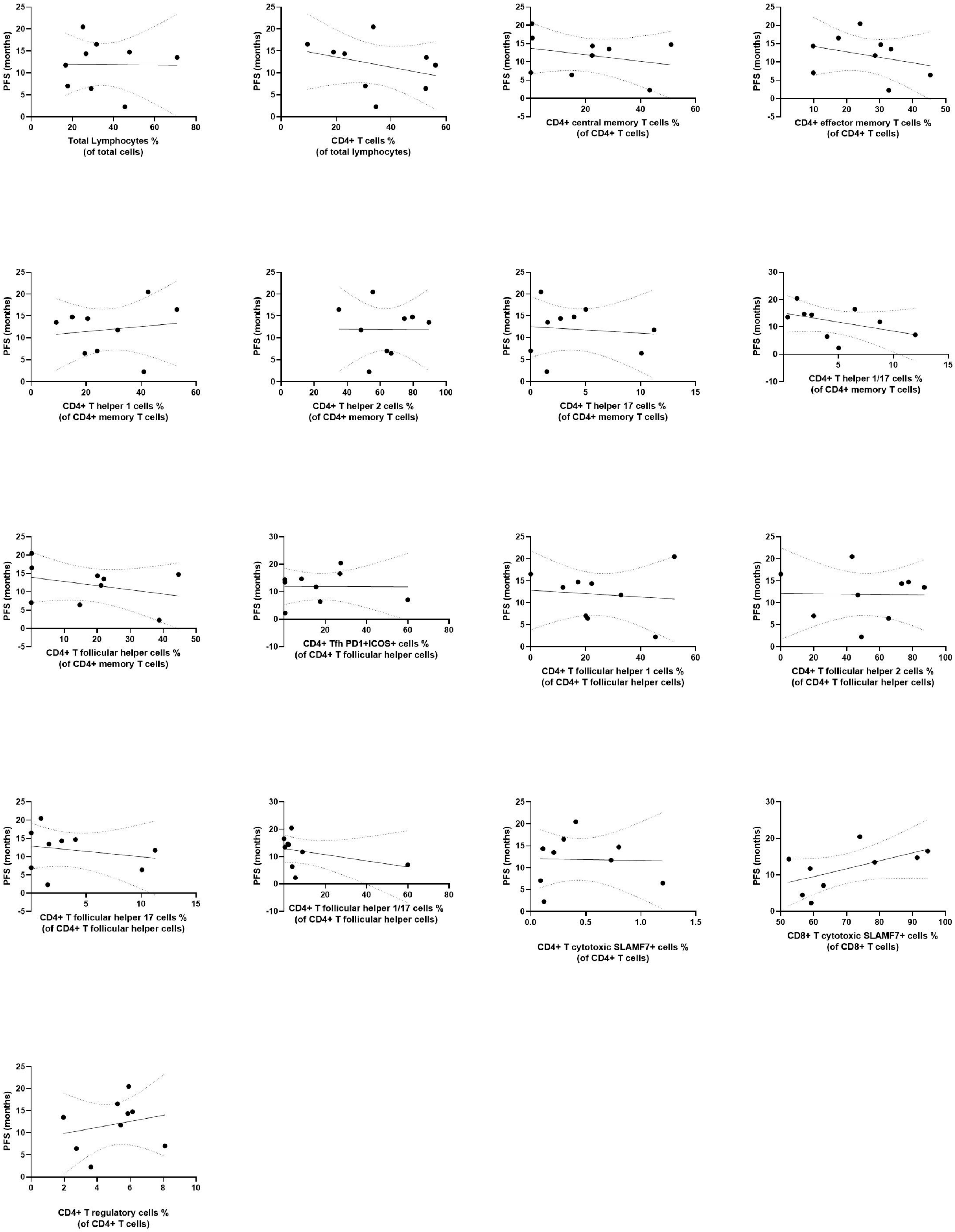
PFS (months)
Total Lymphocytes %
(of total cells)
CD4+ T cells %
(of total lymphocytes)
CD4+ central memory T cells %
(of CD4+ T cells)
CD4+ effector memory T cells %
(of CD4+ T cells)
CD4+ T helper 1 cells %
(of CD4+ memory T cells)
CD4+ T helper 2 cells %
(of CD4+ memory T cells)
CD4+ T helper 17 cells %
(of CD4+ memory T cells)
CD4+ T helper 1/17 cells %
(of CD4+ memory T cells)
CD4+ T follicular helper cells %
(of CD4+ memory T cells)
CD4+ Tfh PD1+ICOS+ cells %
(of CD4+ T follicular helper cells)
CD4+ T follicular helper 1 cells %
(of CD4+ T follicular helper cells)
CD4+ T follicular helper 2 cells %
(of CD4+ T follicular helper cells)
CD4+ T follicular helper 17 cells %
(of CD4+ T follicular helper cells)
CD4+ T follicular helper 1/17 cells %
(of CD4+ T follicular helper cells)
CD4+ T cytotoxic SLAMF7+ cells %
(of CD4+ T cells)
CD8+ T cytotoxic SLAMF7+ cells %
(of CD8+ T cells)
CD4+ T regulatory cells %
(of CD4+ T cells)

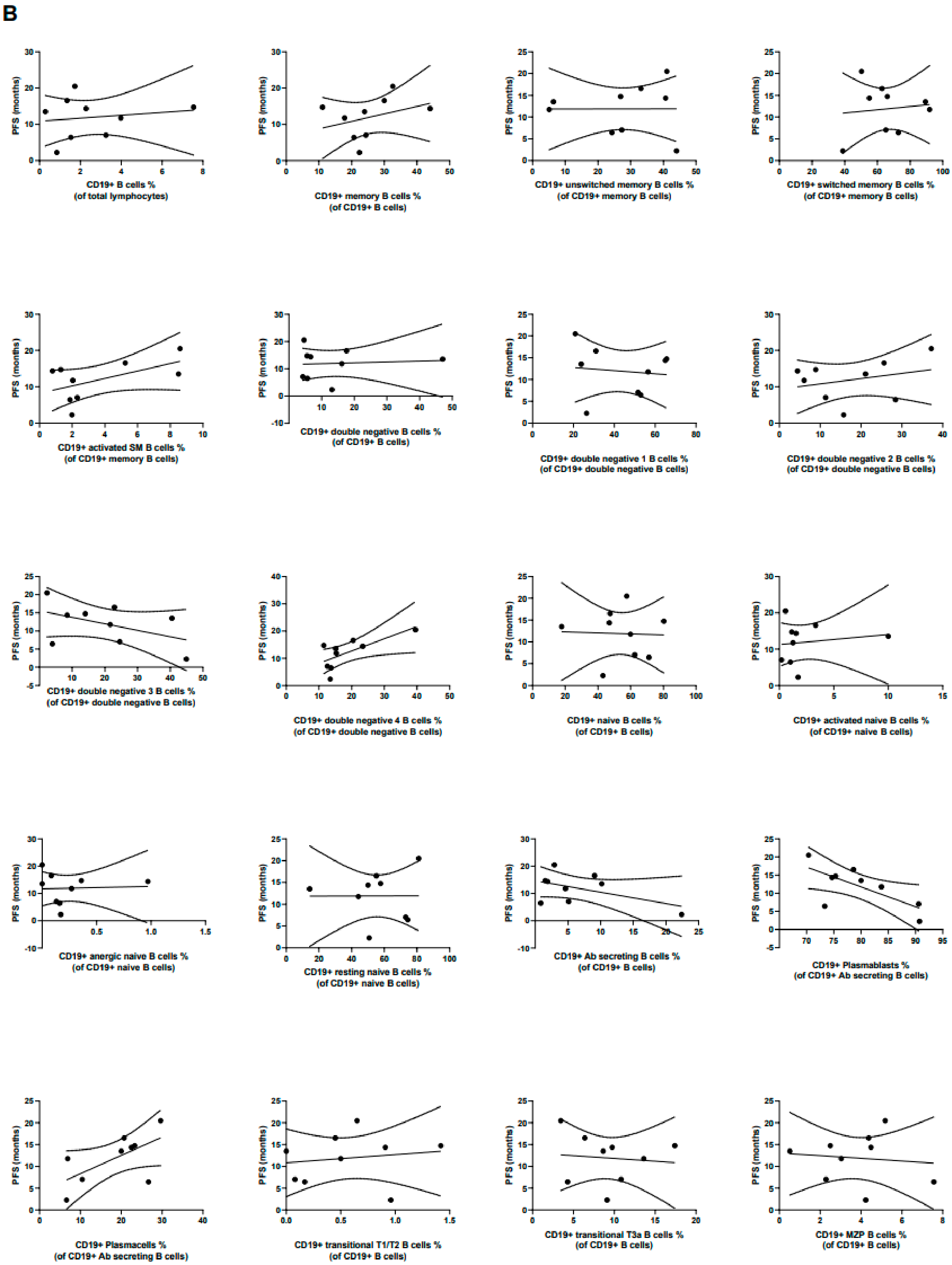
B
PFS (months)
CD19+ B cells % (of total lymphocytes)
CD19+ memory B cells % (of CD19+ B cells)
CD19+ unswitched memory B cells % (of CD19+ memory B cells)
CD19+ switched memory B cells % (of CD19+ memory B cells)
CD19+ activated SM B cells % (of CD19+ memory B cells)
CD19+ double negative B cells % (of CD19+ B cells)
CD19+ double negative 1 B cells % (of CD19+ double negative B cells)
CD19+ double negative 2 B cells % (of CD19+ double negative B cells)
CD19+ double negative 3 B cells % (of CD19+ double negative B cells)
CD19+ double negative 4 B cells % (of CD19+ double negative B cells)
CD19+ naive B cells % (of CD19+ B cells)
CD19+ activated naive B cells % (of CD19+ naive B cells)
CD19+ anergic naive B cells % (of CD19+ naive B cells)
CD19+ resting naive B cells % (of CD19+ naive B cells)
CD19+ Ab secreting B cells % (of CD19+ B cells)
CD19+ Plasmablasts % (of CD19+ Ab secreting B cells)
CD19+ Plasmacells % (of CD19+ Ab secreting B cells)
CD19+ transitional T1/T2 B cells % (of CD19+ B cells)
CD19+ transitional T3a B cells % (of CD19+ B cells)
CD19+ MZP B cells % (of CD19+ B cells)

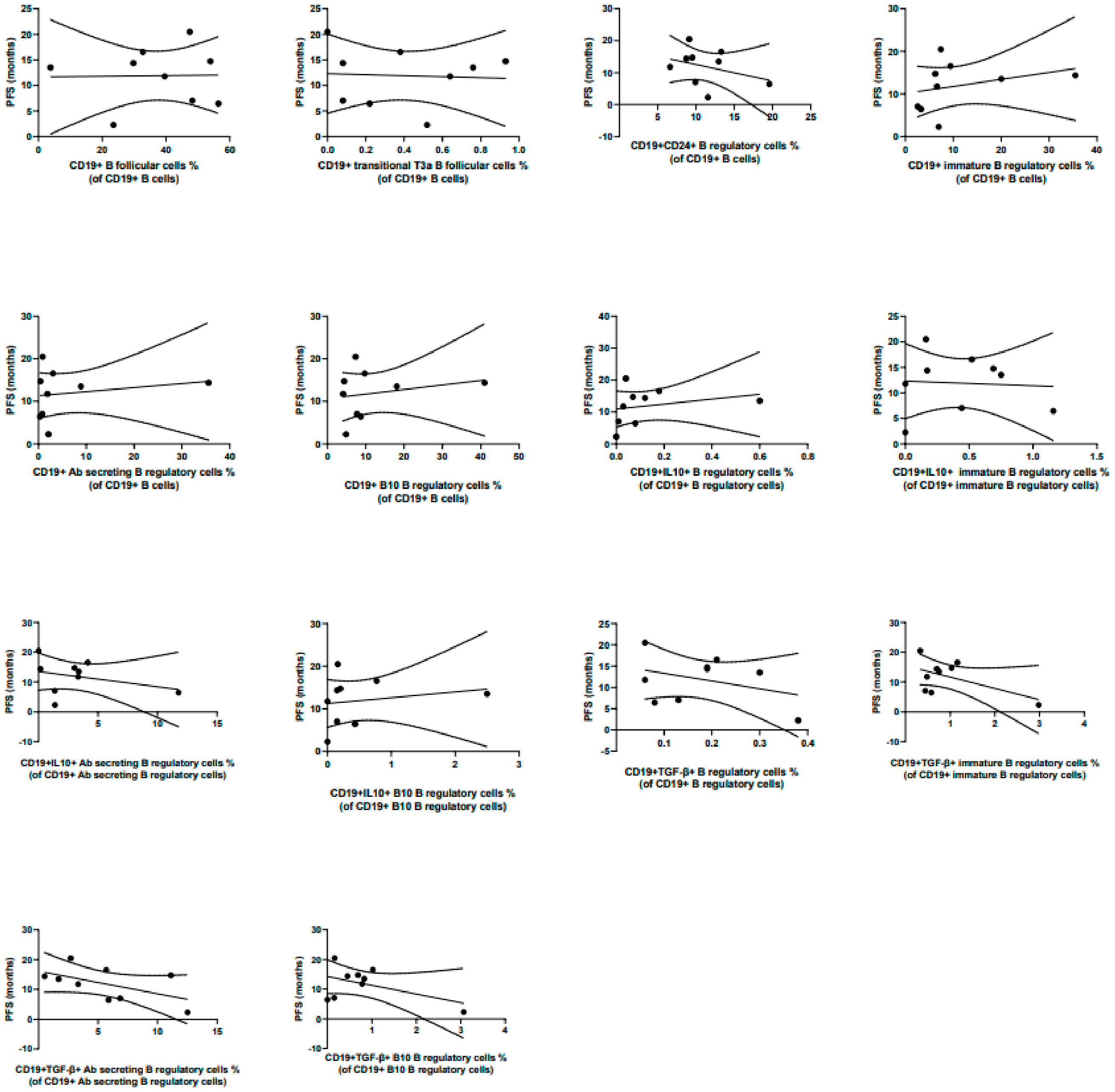
PFS (months)
CD19+ B follicular cells % (of CD19+ B cells)
CD19+ transitional T3a B follicular cells % (of CD19+ B cells)
CD19+CD24+ B regulatory cells % (of CD19+ B cells)
CD19+ immature B regulatory cells % (of CD19+ B cells)
CD19+ Ab secreting B regulatory cells % (of CD19+ B cells)
CD19+ B10 B regulatory cells % (of CD19+ B cells)
CD19+IL10+ B regulatory cells % (of CD19+ B regulatory cells)
CD19+IL10+ immature B regulatory cells % (of CD19+ immature B regulatory cells)
CD19+IL10+ Ab secreting B regulatory cells % (of CD19+ Ab secreting B regulatory cells)
CD19+IL10+ B10 B regulatory cells % (of CD19+ B10 B regulatory cells)
CD19+TGF-β+ B regulatory cells % (of CD19+ B regulatory cells)
CD19+TGF-β+ immature B regulatory cells % (of CD19+ immature B regulatory cells)
CD19+TGF-β+ Ab secreting B regulatory cells % (of CD19+ Ab secreting B regulatory cells)
CD19+TGF-β+ B10 B regulatory cells % (of CD19+ B10 B regulatory cells)

C

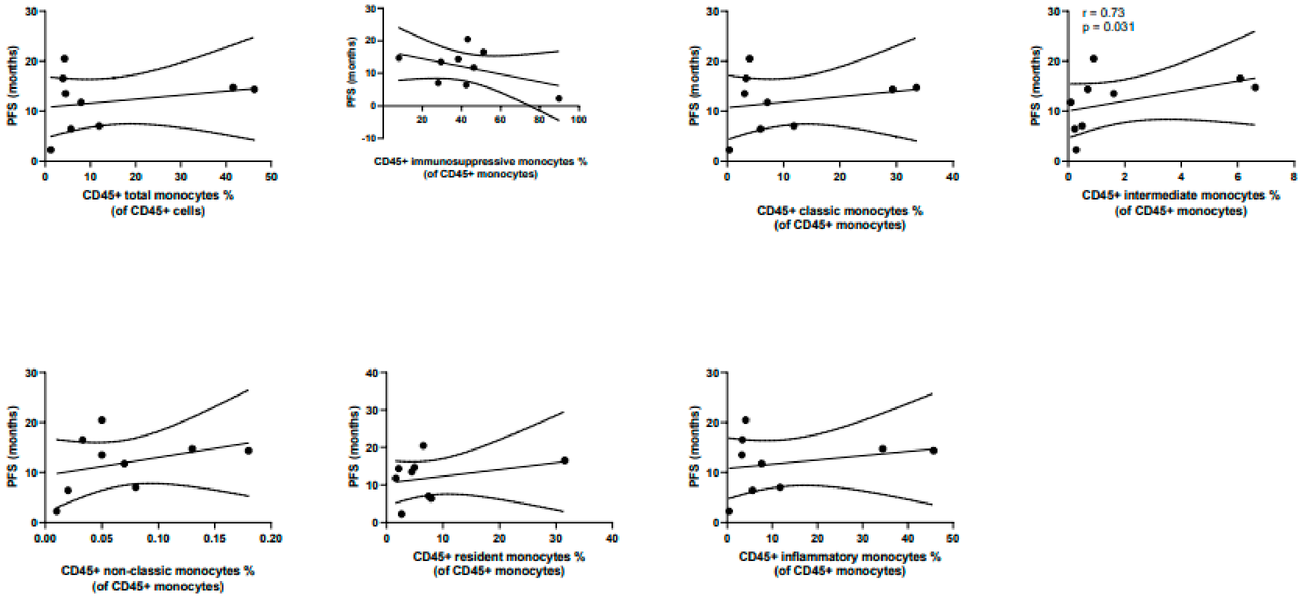
PFS (months)
CD45+ total monocytes %
(of CD45+ cells)
CD45+ immunosuppressive monocytes %
(of CD45+ monocytes)
CD45+ classic monocytes %
(of CD45+ monocytes)
r = 0.73
p = 0.031
CD45+ intermediate monocytes %
(of CD45+ monocytes)
CD45+ non-classic monocytes %
(of CD45+ monocytes)
CD45+ resident monocytes %
(of CD45+ monocytes)
CD45+ inflammatory monocytes %
(of CD45+ monocytes)

D

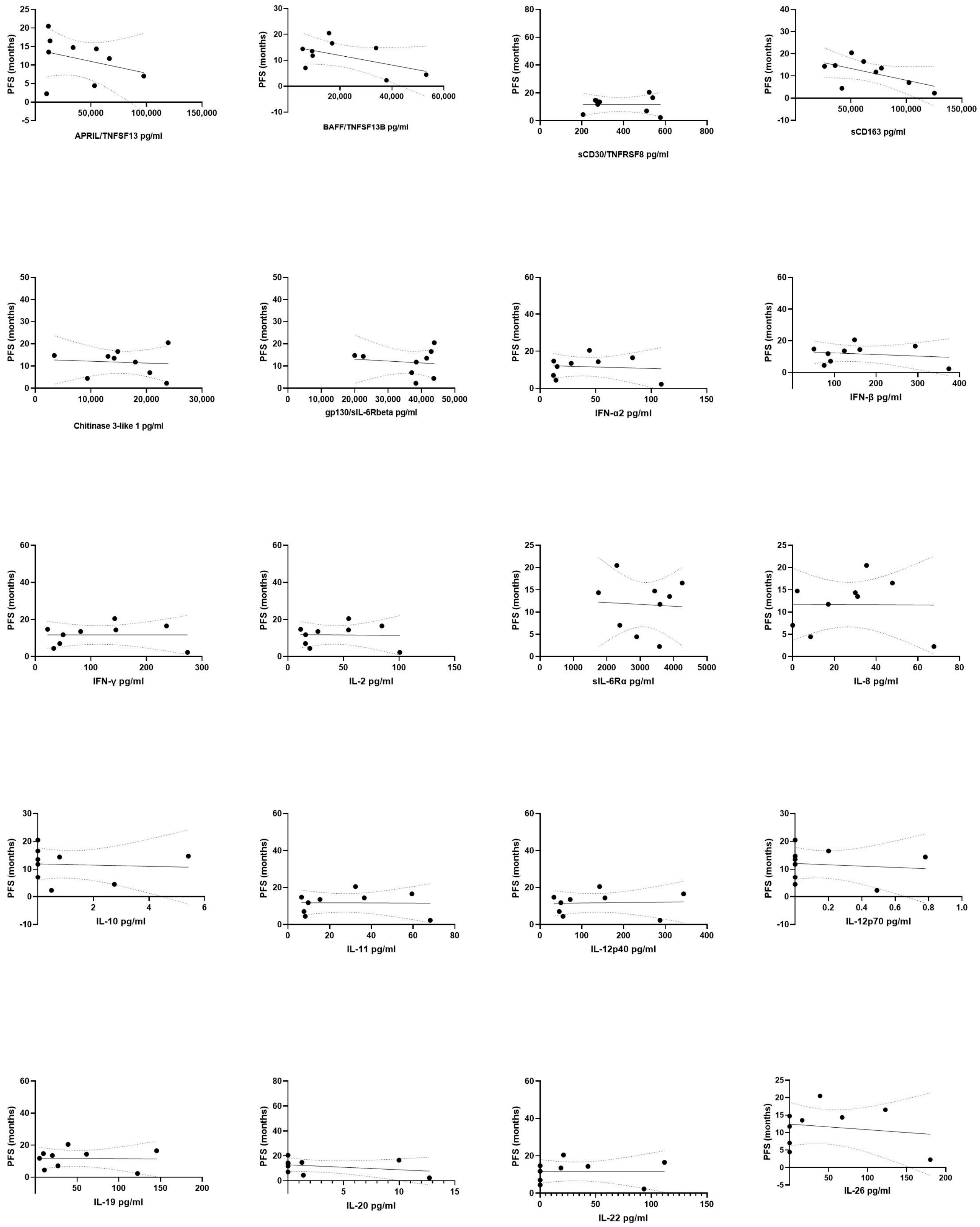
PFS (months)
APRIL/TNFSF13 pg/ml
BAFF/TNFSF13B pg/ml
sCD30/TNFRSF8 pg/ml
sCD163 pg/ml
Chitinase 3-like 1 pg/ml
gp130/sIL-6Rbeta pg/ml
IFN-α2 pg/ml
IFN-β pg/ml
IFN-γ pg/ml
IL-2 pg/ml
sIL-6Rα pg/ml
IL-8 pg/ml
IL-10 pg/ml
IL-11 pg/ml
IL-12p40 pg/ml
IL-12p70 pg/ml
IL-19 pg/ml
IL-20 pg/ml
IL-22 pg/ml
IL-26 pg/ml

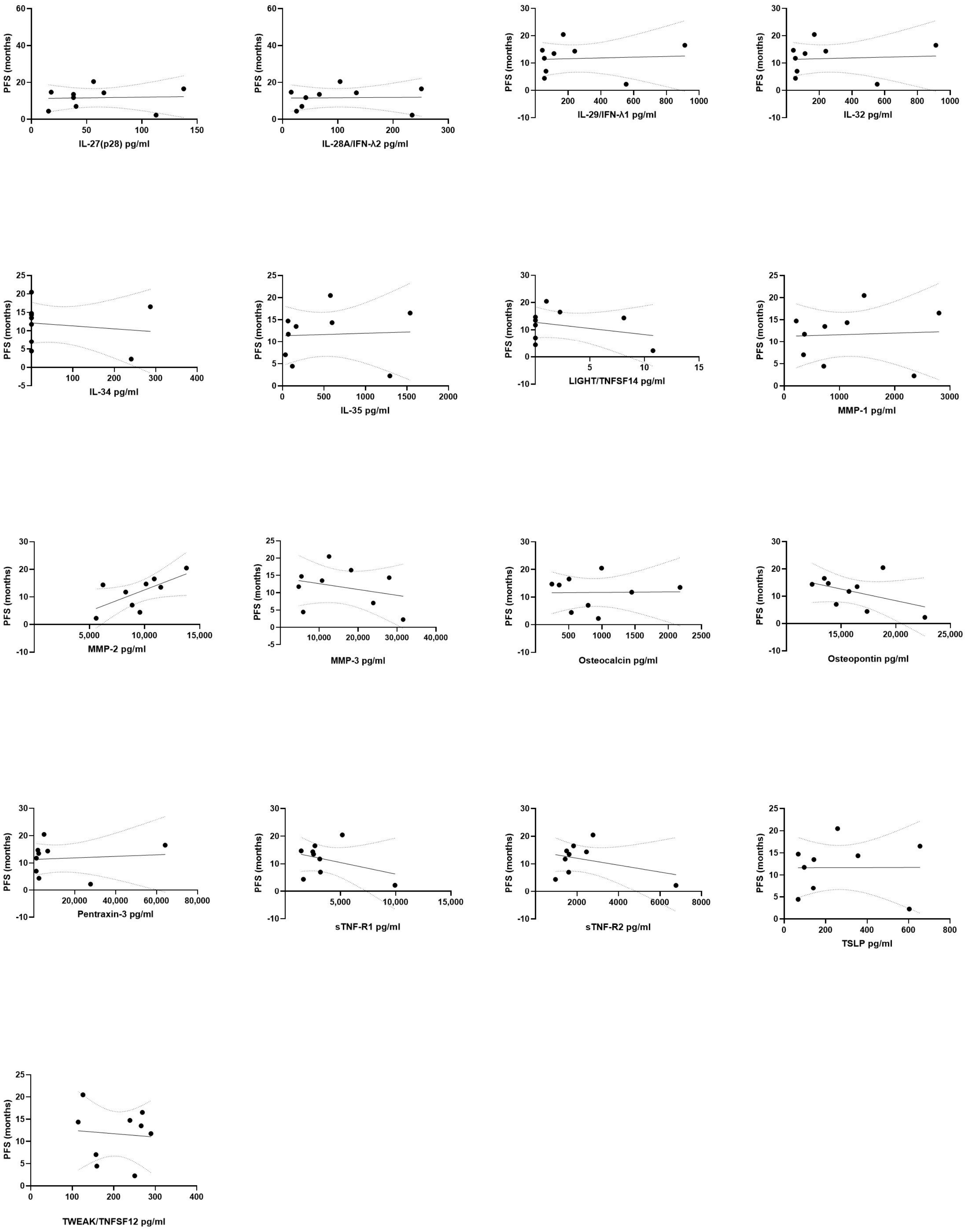
PFS (months)
IL-27(p28) pg/ml
IL-28A/IFN-λ2 pg/ml
IL-29/IFN-λ1 pg/ml
IL-32 pg/ml
IL-34 pg/ml
IL-35 pg/ml
LIGHT/TNFSF14 pg/ml
MMP-1 pg/ml
MMP-2 pg/ml
MMP-3 pg/ml
Osteocalcin pg/ml
Osteopontin pg/ml
Pentraxin-3 pg/ml
sTNF-R1 pg/ml
sTNF-R2 pg/ml
TSLP pg/ml
TWEAK/TNFSF12 pg/ml

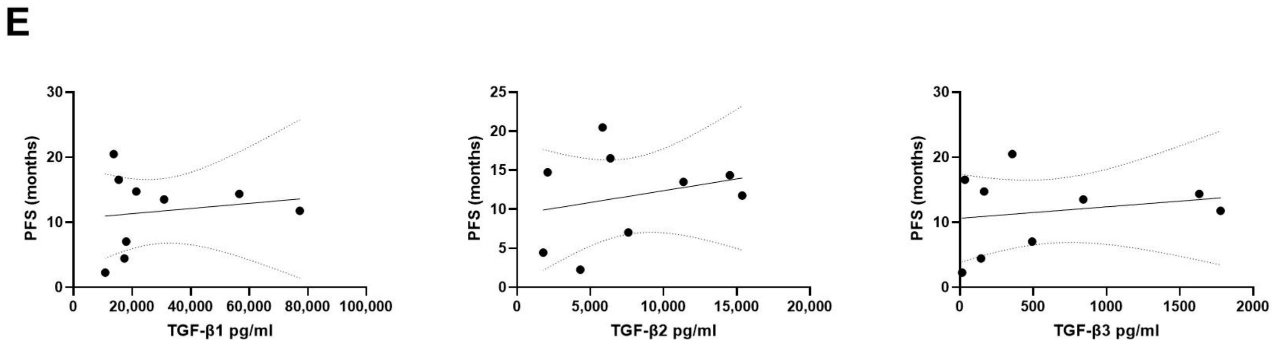
E
PFS (months)
TGF-β1 pg/ml
PFS (months)
TGF-β2 pg/ml
PFS (months)
TGF-β3 pg/ml

320

F

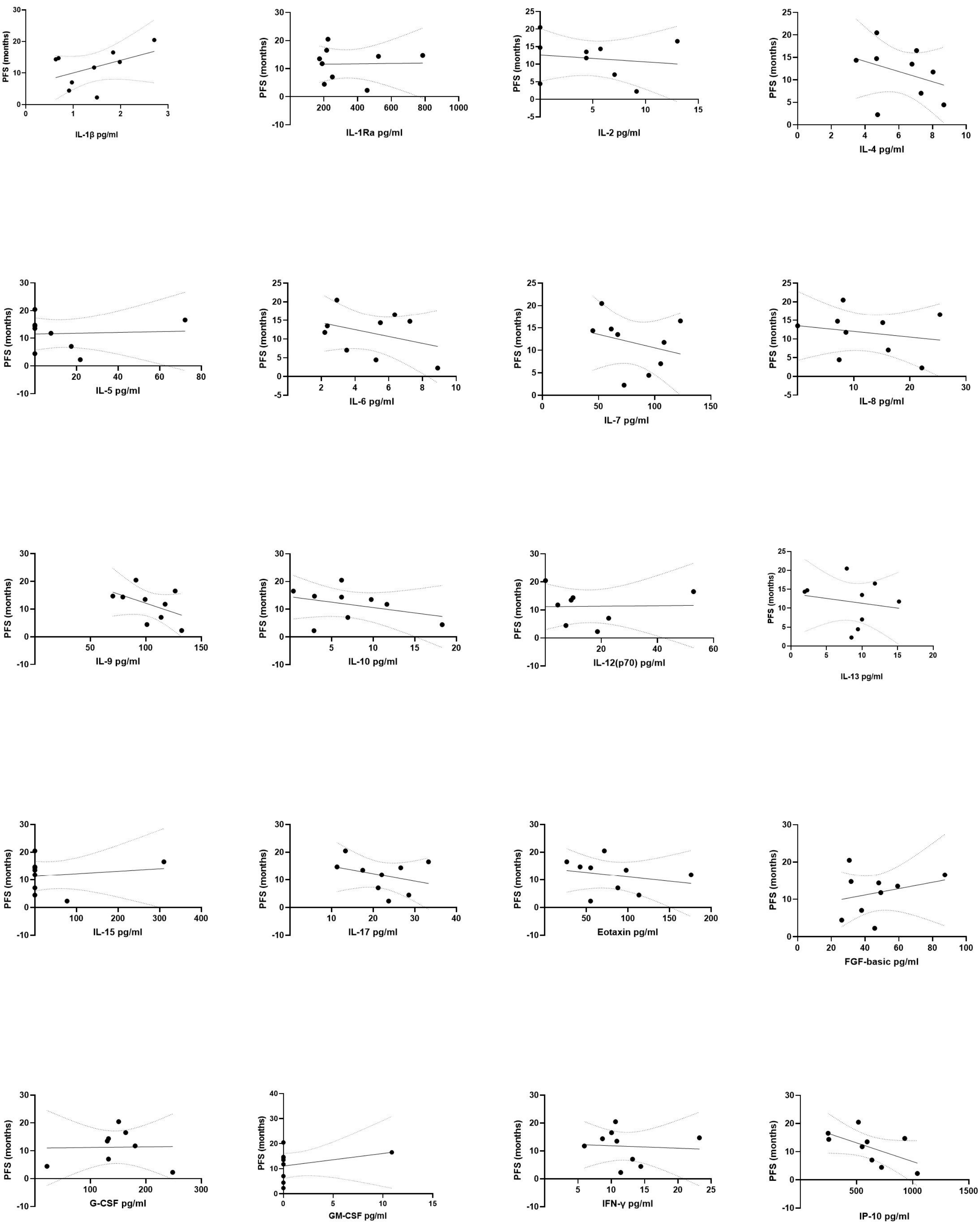
PFS (months)
IL-1β pg/ml
IL-1Ra pg/ml
IL-2 pg/ml
IL-4 pg/ml
IL-5 pg/ml
IL-6 pg/ml
IL-7 pg/ml
IL-8 pg/ml
IL-9 pg/ml
IL-10 pg/ml
IL-12(p70) pg/ml
IL-13 pg/ml
IL-15 pg/ml
IL-17 pg/ml
Eotaxin pg/ml
FGF-basic pg/ml
G-CSF pg/ml
GM-CSF pg/ml
IFN-γ pg/ml
IP-10 pg/ml

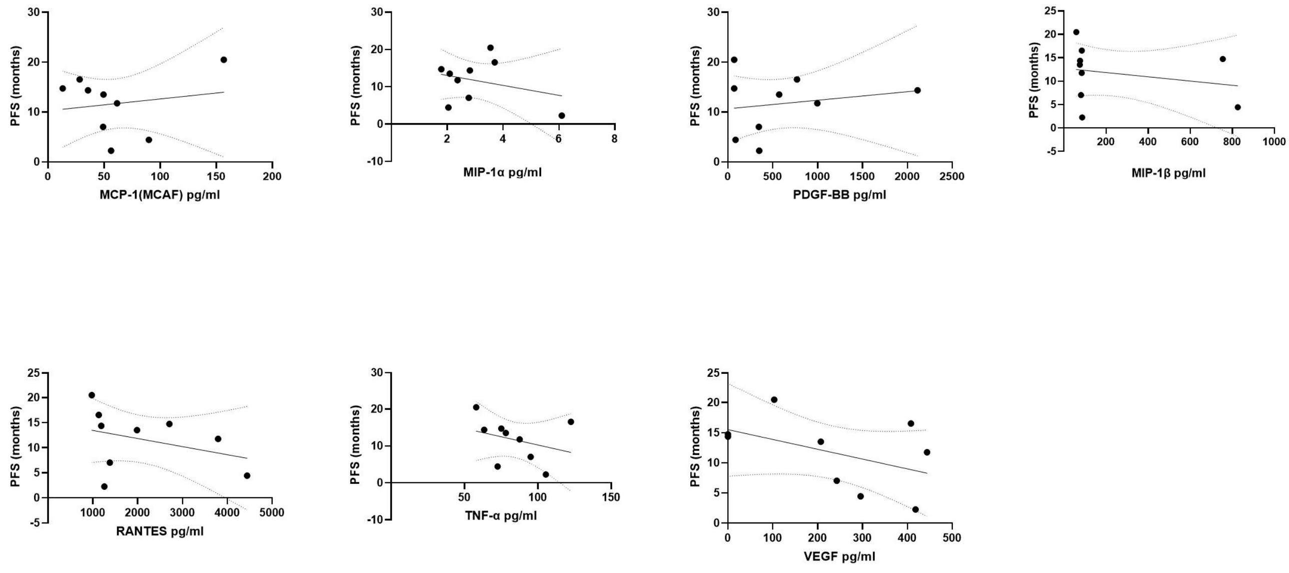

Footnote: Te: T effector; Th1: T helper 1; Th2: T helper 2; Th17: T helper 17; Th1/17: T helper 1/17; Tfh: T follicular helper; Tfh1: T follicular helper 1; Tfh2: T follicular helper 2; Tfh17: T follicular helper 17; Tfh1/17: T follicular helper 1/17; Treg: T regulatory; UM: unswitched memory; SM: switched memory; ASM: activated switched memory; DN: double negative; DN1: double negative 1; DN2: double negative 2; DN3: double negative 3; DN4: double negative 4; AcN: activated naïve; AnN: anergic naïve; RN: resting naïve; Ab: antibody secreting; T1/2: transitional 1/2; T3a: transitional 3a; MZP: marginal zone peripheral; Breg: B regulatory; AbS: antibody secreting; Imm: immature; Mon: monocytes.

Figure S8. Correlation studies between circulating immune cells (A: T-cell compartment; B: B-cell compartment; C: monocyte compartment) and concentrations of inflammatory and immune-related cytokines and chemokines (D: Pro Human Inflammation Panel I Assay; E: Pro TGF-β Immunoassay; F: Pro Human Cytokine Immunoassay) at 4-month follow-up and overall survival (OS) after therapy onset in patients with pancreatic adenocarcinoma (PDAC). Correlation studies were performed using the Spearman's rho rank correlation test.

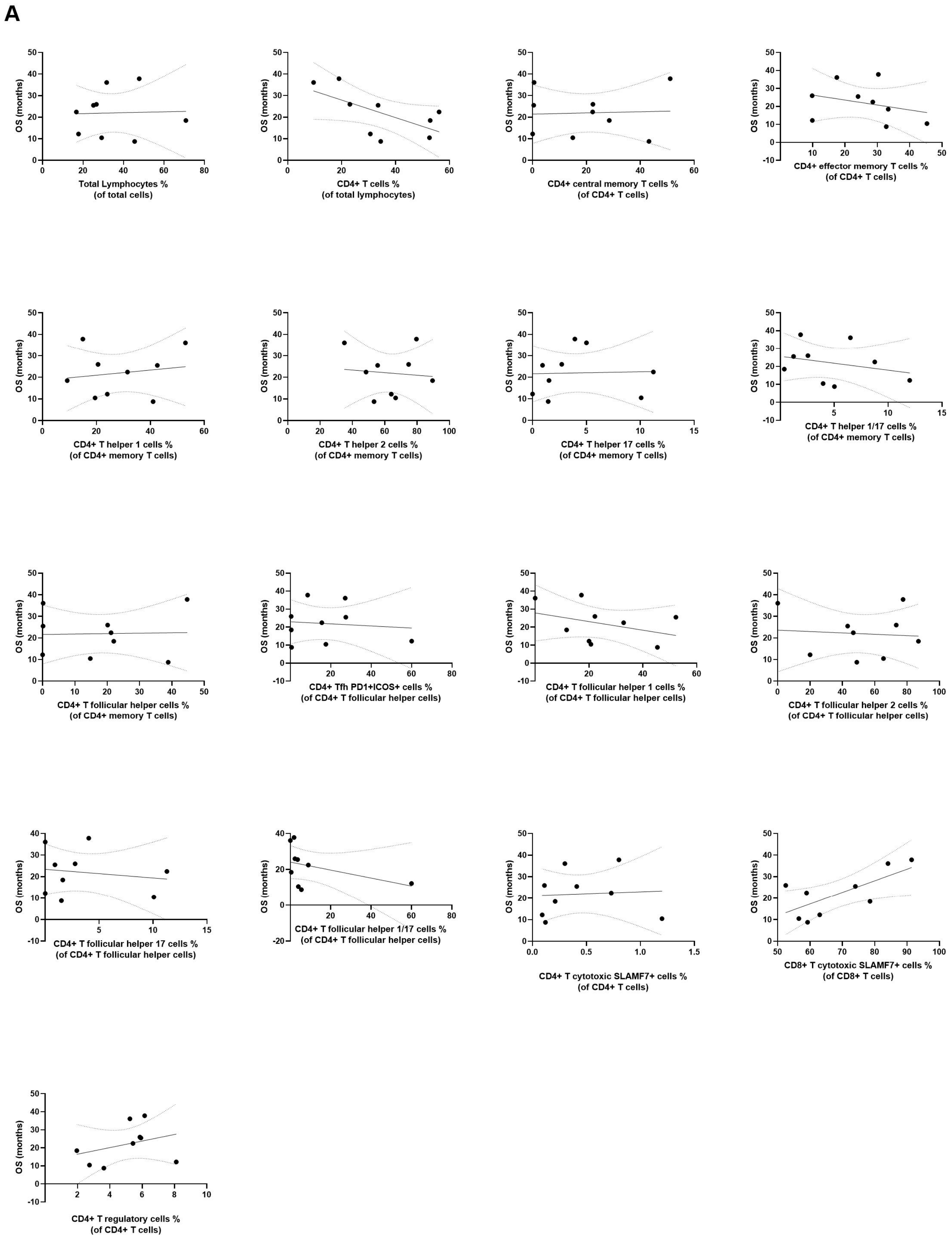
A
OS (months)
Total Lymphocytes %
(of total cells)
CD4+ T cells %
(of total lymphocytes)
CD4+ central memory T cells %
(of CD4+ T cells)
CD4+ effector memory T cells %
(of CD4+ T cells)
CD4+ T helper 1 cells %
(of CD4+ memory T cells)
CD4+ T helper 2 cells %
(of CD4+ memory T cells)
CD4+ T helper 17 cells %
(of CD4+ memory T cells)
CD4+ T helper 1/17 cells %
(of CD4+ memory T cells)
CD4+ T follicular helper cells %
(of CD4+ memory T cells)
CD4+ Tfh PD1+ICOS+ cells %
(of CD4+ T follicular helper cells)
CD4+ T follicular helper 1 cells %
(of CD4+ T follicular helper cells)
CD4+ T follicular helper 2 cells %
(of CD4+ T follicular helper cells)
CD4+ T follicular helper 17 cells %
(of CD4+ T follicular helper cells)
CD4+ T follicular helper 1/17 cells %
(of CD4+ T follicular helper cells)
CD4+ T cytotoxic SLAMF7+ cells %
(of CD4+ T cells)
CD8+ T cytotoxic SLAMF7+ cells %
(of CD8+ T cells)
CD4+ T regulatory cells %
(of CD4+ T cells)

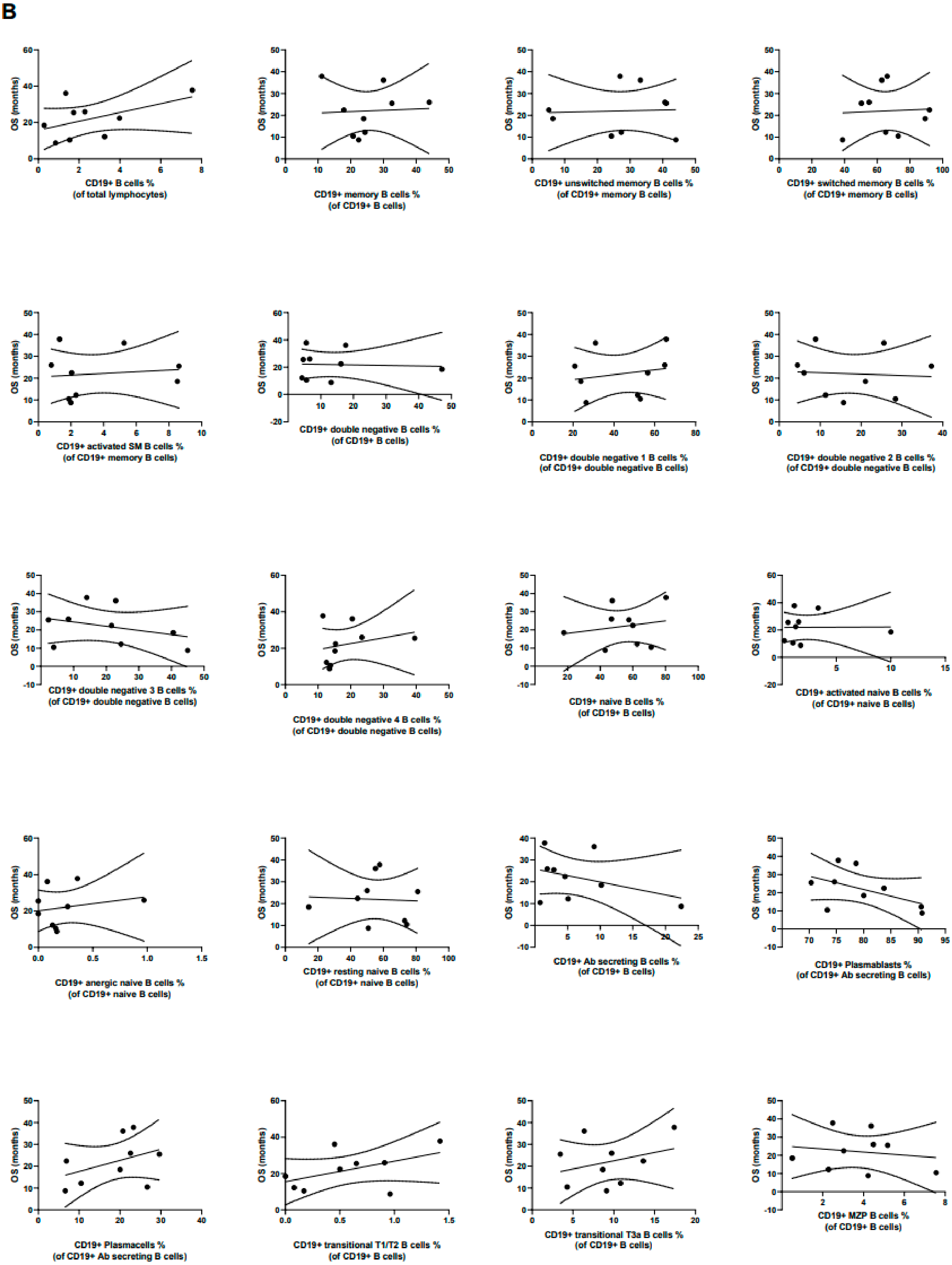
B
OS (months)
CD19+ B cells %
(of total lymphocytes)
CD19+ memory B cells %
(of CD19+ B cells)
CD19+ unswitched memory B cells %
(of CD19+ memory B cells)
CD19+ switched memory B cells %
(of CD19+ memory B cells)
CD19+ activated SM B cells %
(of CD19+ memory B cells)
CD19+ double negative B cells %
(of CD19+ B cells)
CD19+ double negative 1 B cells %
(of CD19+ double negative B cells)
CD19+ double negative 2 B cells %
(of CD19+ double negative B cells)
CD19+ double negative 3 B cells %
(of CD19+ double negative B cells)
CD19+ double negative 4 B cells %
(of CD19+ double negative B cells)
CD19+ naive B cells %
(of CD19+ B cells)
CD19+ activated naive B cells %
(of CD19+ naive B cells)
CD19+ anergic naive B cells %
(of CD19+ naive B cells)
CD19+ resting naive B cells %
(of CD19+ naive B cells)
CD19+ Ab secreting B cells %
(of CD19+ B cells)
CD19+ Plasmablasts %
(of CD19+ Ab secreting B cells)
CD19+ Plasmacells %
(of CD19+ Ab secreting B cells)
CD19+ transitional T1/T2 B cells %
(of CD19+ B cells)
CD19+ transitional T3a B cells %
(of CD19+ B cells)
CD19+ MZP B cells %
(of CD19+ B cells)

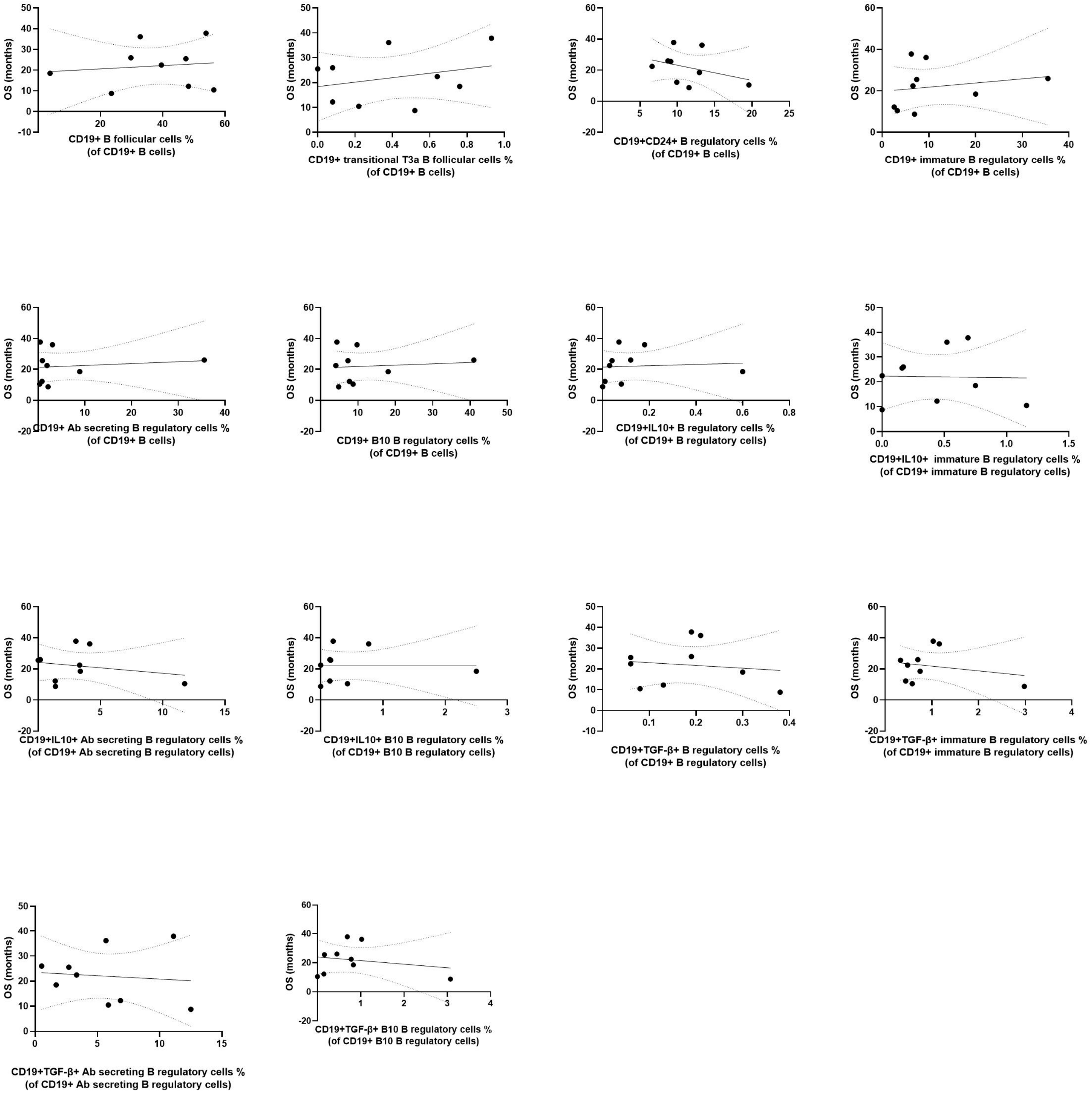
OS (months)
CD19+ B follicular cells %
(of CD19+ B cells)
CD19+ transitional T3a B follicular cells %
(of CD19+ B cells)
CD19+CD24+ B regulatory cells %
(of CD19+ B cells)
CD19+ immature B regulatory cells %
(of CD19+ B cells)
CD19+ Ab secreting B regulatory cells %
(of CD19+ B cells)
CD19+ B10 B regulatory cells %
(of CD19+ B cells)
CD19+IL10+ B regulatory cells %
(of CD19+ B regulatory cells)
CD19+IL10+ immature B regulatory cells %
(of CD19+ immature B regulatory cells)
CD19+IL10+ Ab secreting B regulatory cells %
(of CD19+ Ab secreting B regulatory cells)
CD19+IL10+ B10 B regulatory cells %
(of CD19+ B10 B regulatory cells)
CD19+TGF-β+ B regulatory cells %
(of CD19+ B regulatory cells)
CD19+TGF-β+ immature B regulatory cells %
(of CD19+ immature B regulatory cells)
CD19+TGF-β+ Ab secreting B regulatory cells %
(of CD19+ Ab secreting B regulatory cells)
CD19+TGF-β+ B10 B regulatory cells %
(of CD19+ B10 B regulatory cells)

C

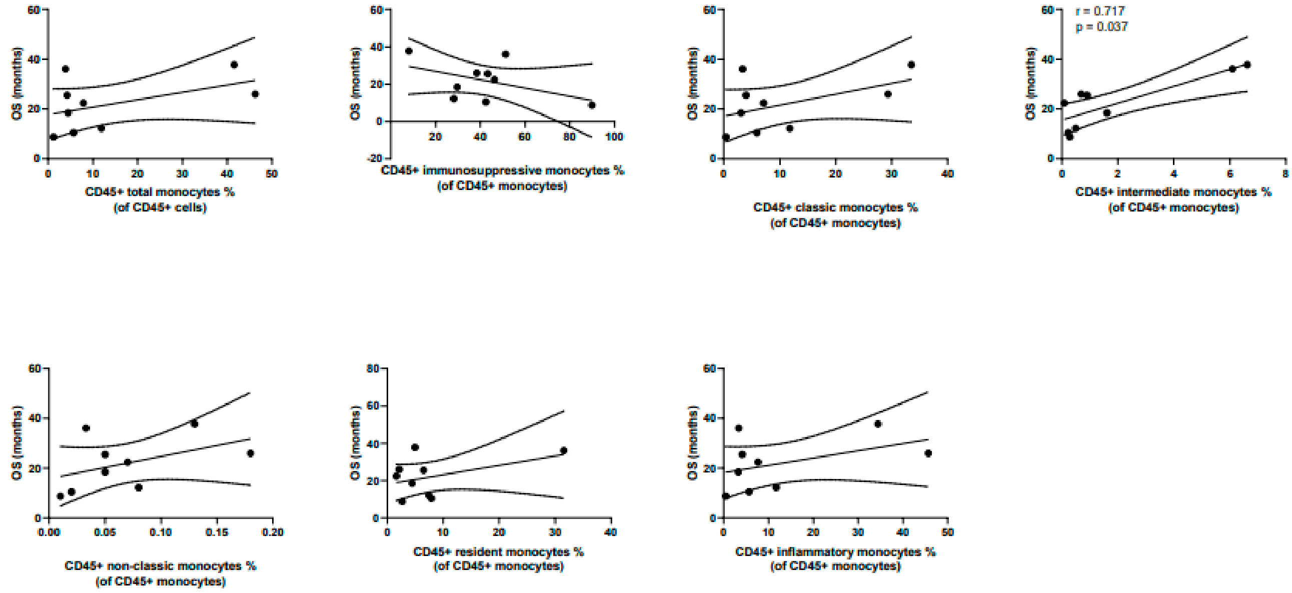
OS (months)
CD45+ total monocytes %
(of CD45+ cells)
CD45+ immunosuppressive monocytes %
(of CD45+ monocytes)
CD45+ classic monocytes %
(of CD45+ monocytes)
r = 0.717
p = 0.037
CD45+ intermediate monocytes %
(of CD45+ monocytes)
CD45+ non-classic monocytes %
(of CD45+ monocytes)
CD45+ resident monocytes %
(of CD45+ monocytes)
CD45+ inflammatory monocytes %
(of CD45+ monocytes)

D

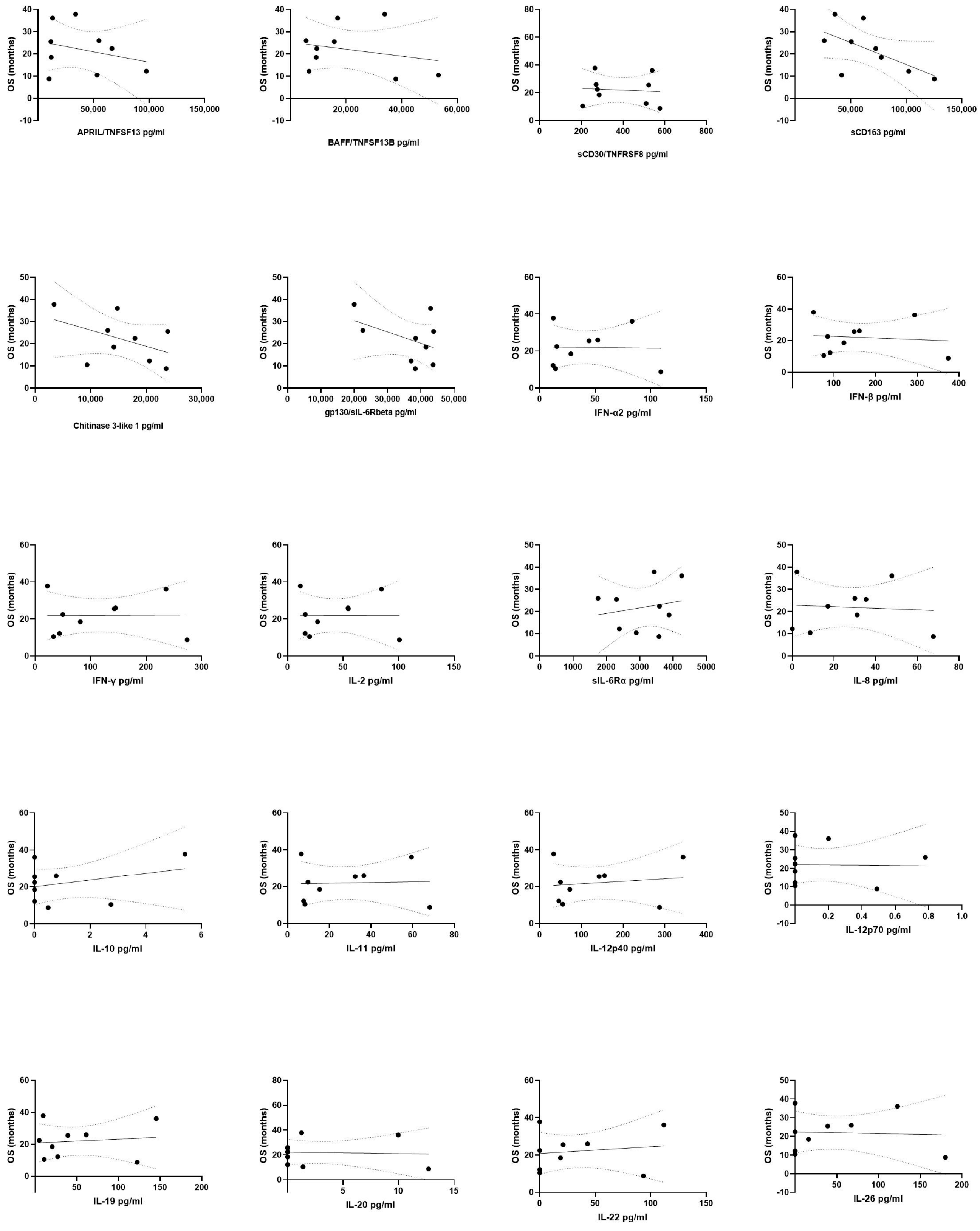
OS (months)
APRIL/TNFSF13 pg/ml
BAFF/TNFSF13B pg/ml
sCD30/TNFRSF8 pg/ml
sCD163 pg/ml
Chitinase 3-like 1 pg/ml
gp130/sIL-6Rbeta pg/ml
IFN-α2 pg/ml
IFN-β pg/ml
IFN-γ pg/ml
IL-2 pg/ml
sIL-6Rα pg/ml
IL-8 pg/ml
IL-10 pg/ml
IL-11 pg/ml
IL-12p40 pg/ml
IL-12p70 pg/ml
IL-19 pg/ml
IL-20 pg/ml
IL-22 pg/ml
IL-26 pg/ml

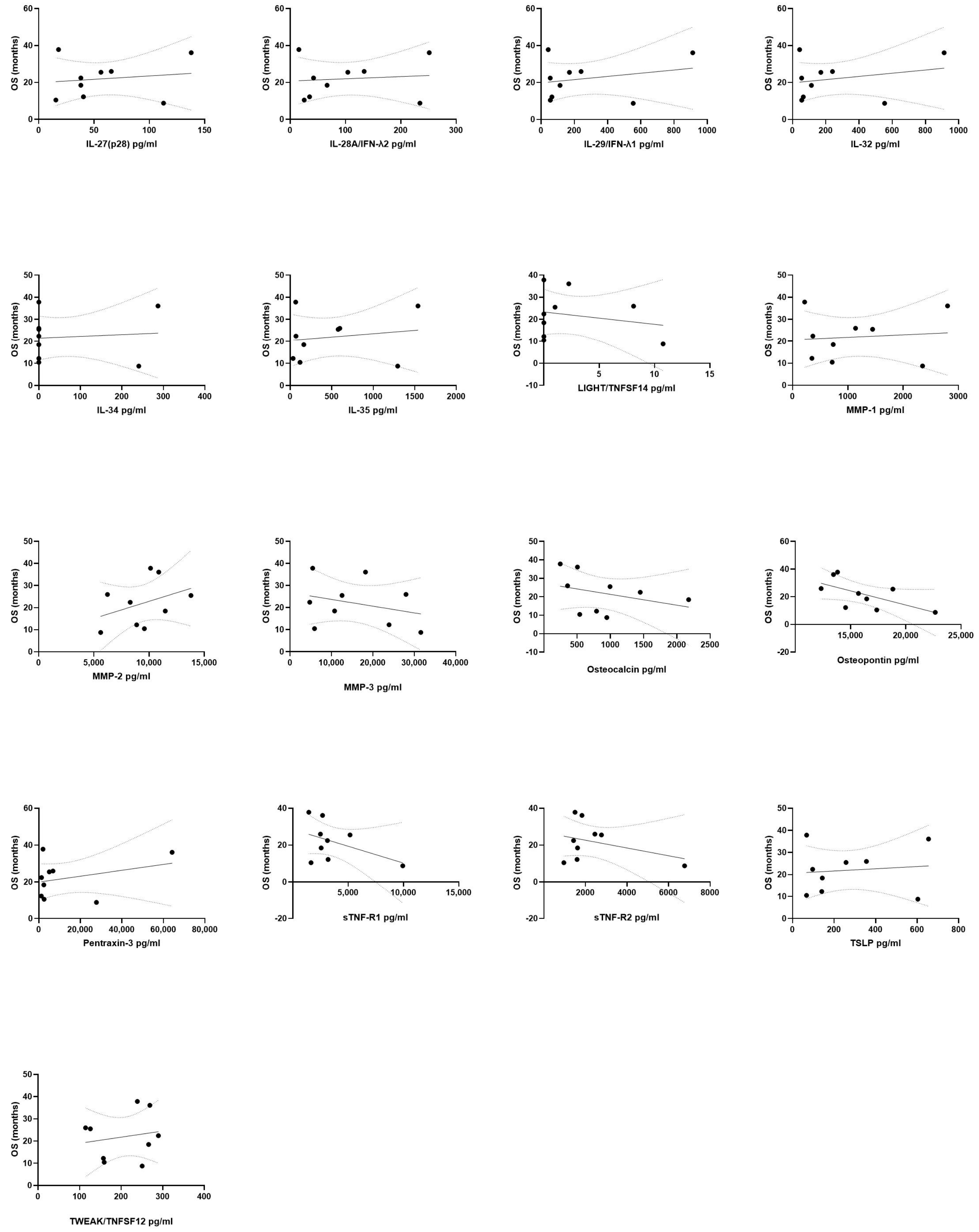
OS (months)
IL-27(p28) pg/ml
IL-28A/IFN-λ2 pg/ml
IL-29/IFN-λ1 pg/ml
IL-32 pg/ml
IL-34 pg/ml
IL-35 pg/ml
LIGHT/TNFSF14 pg/ml
MMP-1 pg/ml
MMP-2 pg/ml
MMP-3 pg/ml
Osteocalcin pg/ml
Osteopontin pg/ml
Pentraxin-3 pg/ml
sTNF-R1 pg/ml
sTNF-R2 pg/ml
TSLP pg/ml
TWEAK/TNFSF12 pg/ml

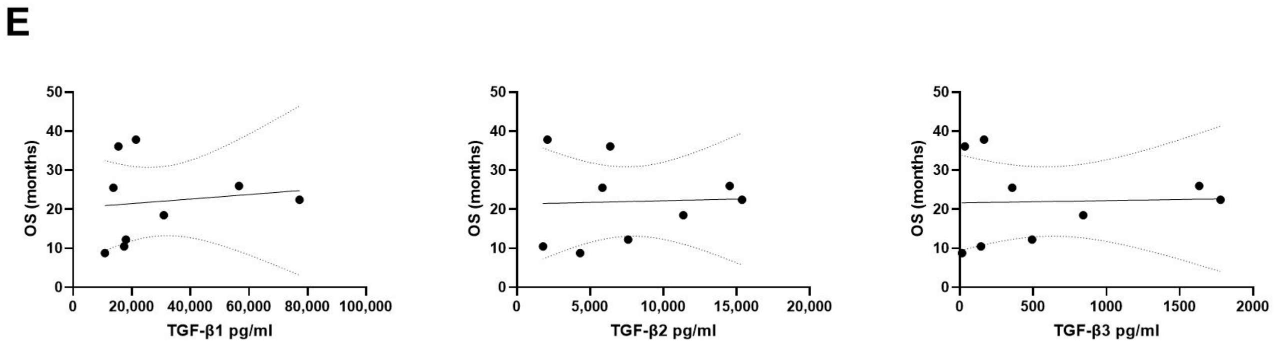
E
50
40
30
20
10
0
OS (months)
0
20,000
40,000
60,000
80,000
100,000
TGF-β1 pg/ml
0
5,000
10,000
15,000
20,000
TGF-β2 pg/ml
500
1000
1500
2000
TGF-β3 pg/ml

359

F

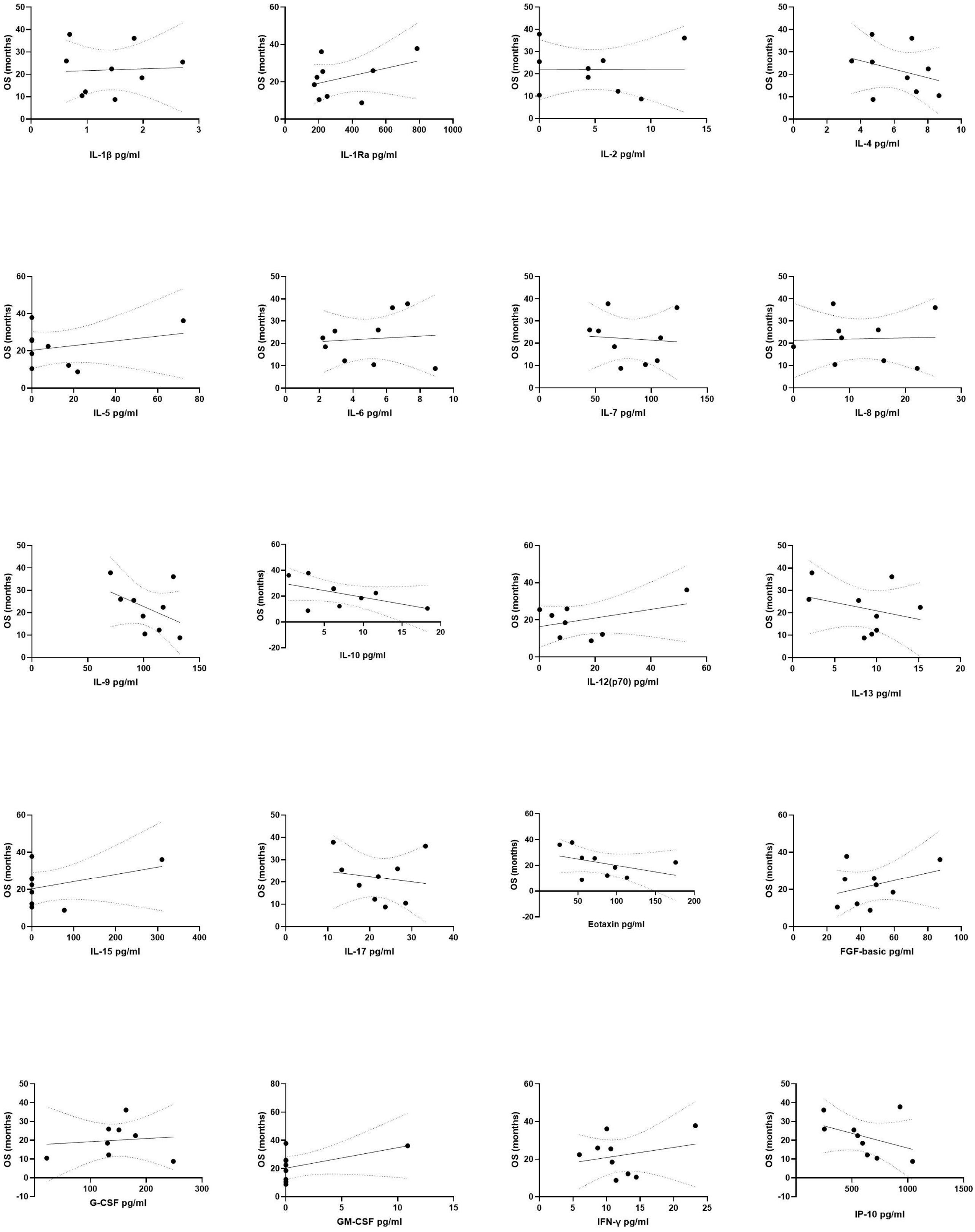
OS (months)
IL-1β pg/ml
IL-1Ra pg/ml
IL-2 pg/ml
IL-4 pg/ml
IL-5 pg/ml
IL-6 pg/ml
IL-7 pg/ml
IL-8 pg/ml
IL-9 pg/ml
IL-10 pg/ml
IL-12(p70) pg/ml
IL-13 pg/ml
IL-15 pg/ml
IL-17 pg/ml
Eotaxin pg/ml
FGF-basic pg/ml
G-CSF pg/ml
GM-CSF pg/ml
IFN-γ pg/ml
IP-10 pg/ml

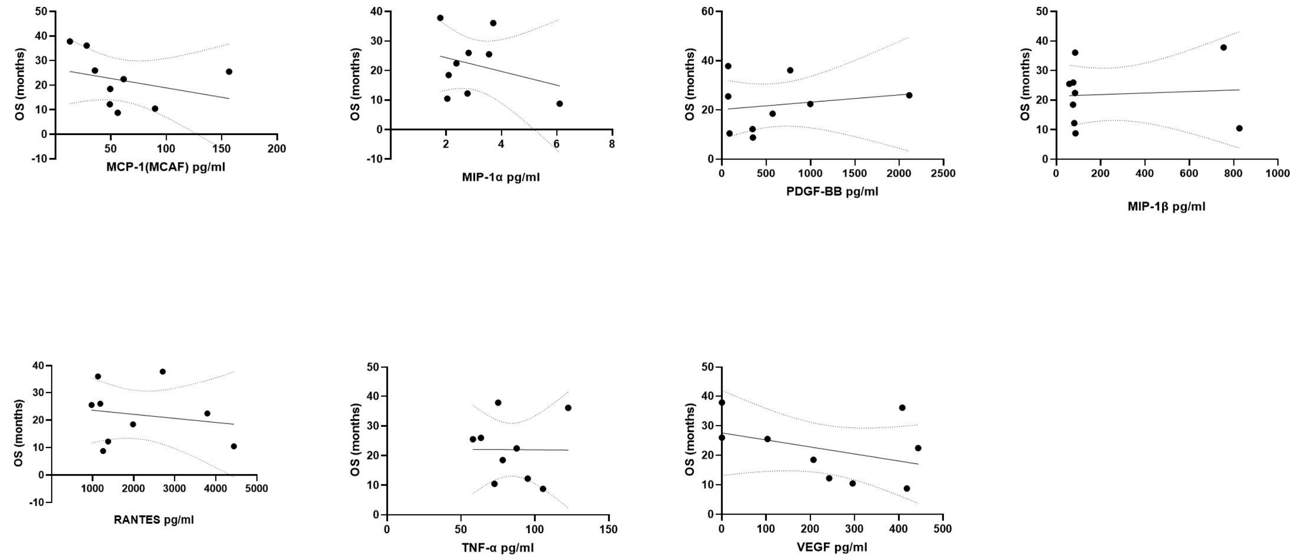

Footnote: Te: T effector; Th1: T helper 1; Th2: T helper 2; Th17: T helper 17; Th1/17: T helper 1/17; Tfh: T follicular helper; Tfh1: T follicular helper 1; Tfh2: T follicular helper 2; Tfh17: T follicular helper 17; Tfh1/17: T follicular helper 1/17; Treg: T regulatory; UM: unswitched memory; SM: switched memory; ASM: activated switched memory; DN: double negative; DN1: double negative 1; DN2: double negative 2; DN3: double negative 3; DN4: double negative 4; AcN: activated naïve; AnN: anergic naïve; RN: resting naïve; Ab: antibody secreting; T1/2: transitional 1/2; T3a: transitional 3a; MZP: marginal zone peripheral; Breg: B regulatory; AbS: antibody secreting; Imm: immature; Mon: monocytes.

Figure S9. Correlation studies between circulating immune cells (A, G: T-cell compartment; B, H: B-cell compartment; C, I: monocyte compartment) and concentrations of inflammatory and immune-related cytokines and chemokines (D, J: Pro Human Inflammation Panel I Assay; E, K: Pro TGF-β Immunoassay; F, L: Pro Human Cytokine Immunoassay) at 4-month follow-up and progression-free survival (PFS) (A-F) and overall survival (OS) (G-L) after therapy onset in patients with pancreatic adenocarcinoma (PDAC) of the HybridTherm ablation plus chemotherapy (HTP-CT) arm. Correlation studies were performed using the Spearman's rho rank correlation test.

A

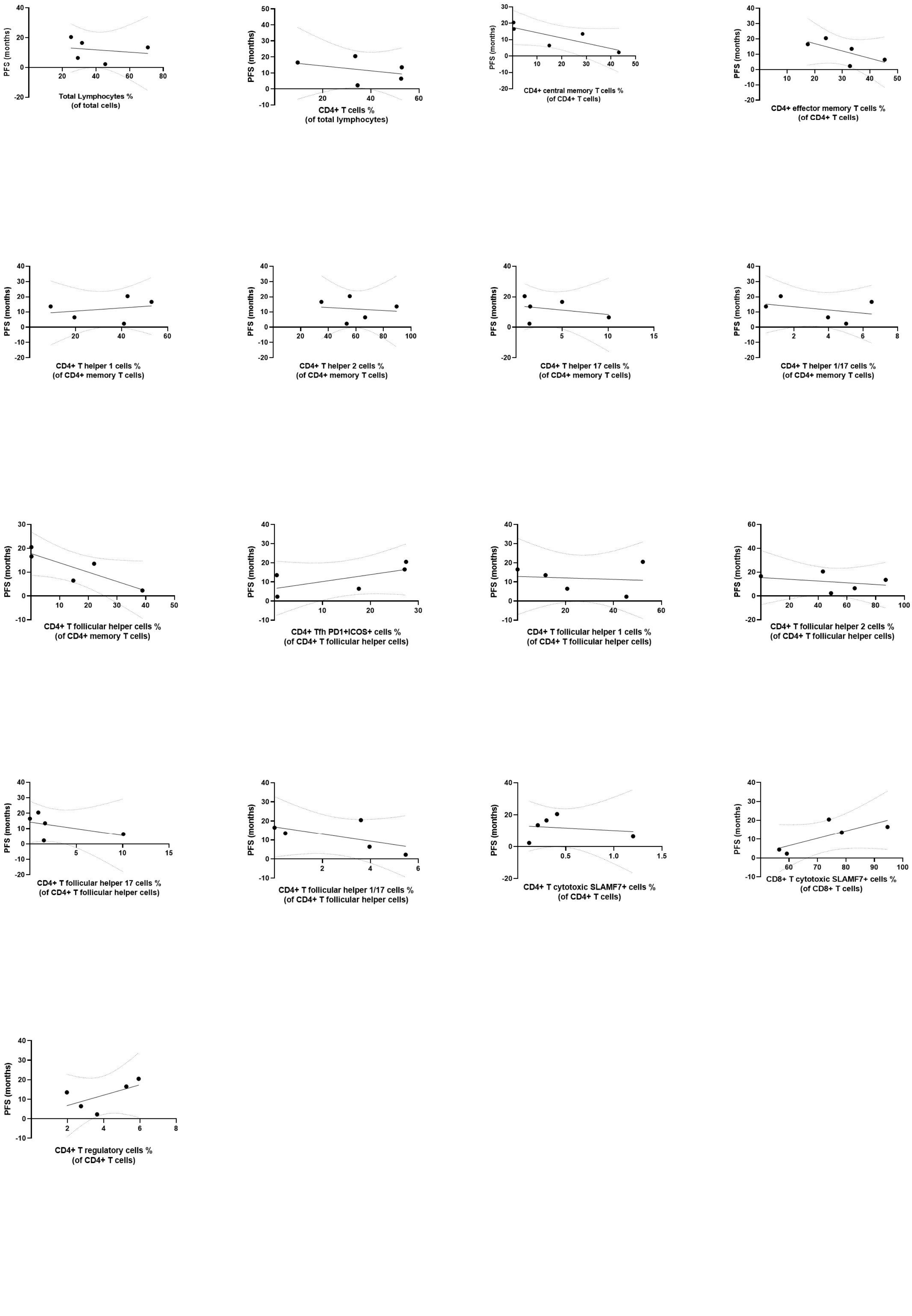
PFS (months)
Total Lymphocytes %
(of total cells)
CD4+ T cells %
(of total lymphocytes)
CD4+ central memory T cells %
(of CD4+ T cells)
CD4+ effector memory T cells %
(of CD4+ T cells)
CD4+ T helper 1 cells %
(of CD4+ memory T cells)
CD4+ T helper 2 cells %
(of CD4+ memory T cells)
CD4+ T helper 17 cells %
(of CD4+ memory T cells)
CD4+ T helper 1/17 cells %
(of CD4+ memory T cells)
CD4+ T follicular helper cells %
(of CD4+ memory T cells)
CD4+ Tfh PD1+ICOS+ cells %
(of CD4+ T follicular helper cells)
CD4+ T follicular helper 1 cells %
(of CD4+ T follicular helper cells)
CD4+ T follicular helper 2 cells %
(of CD4+ T follicular helper cells)
CD4+ T follicular helper 17 cells %
(of CD4+ T follicular helper cells)
CD4+ T follicular helper 1/17 cells %
(of CD4+ T follicular helper cells)
CD4+ T cytotoxic SLAMF7+ cells %
(of CD4+ T cells)
CD8+ T cytotoxic SLAMF7+ cells %
(of CD8+ T cells)
CD4+ T regulatory cells %
(of CD4+ T cells)

B

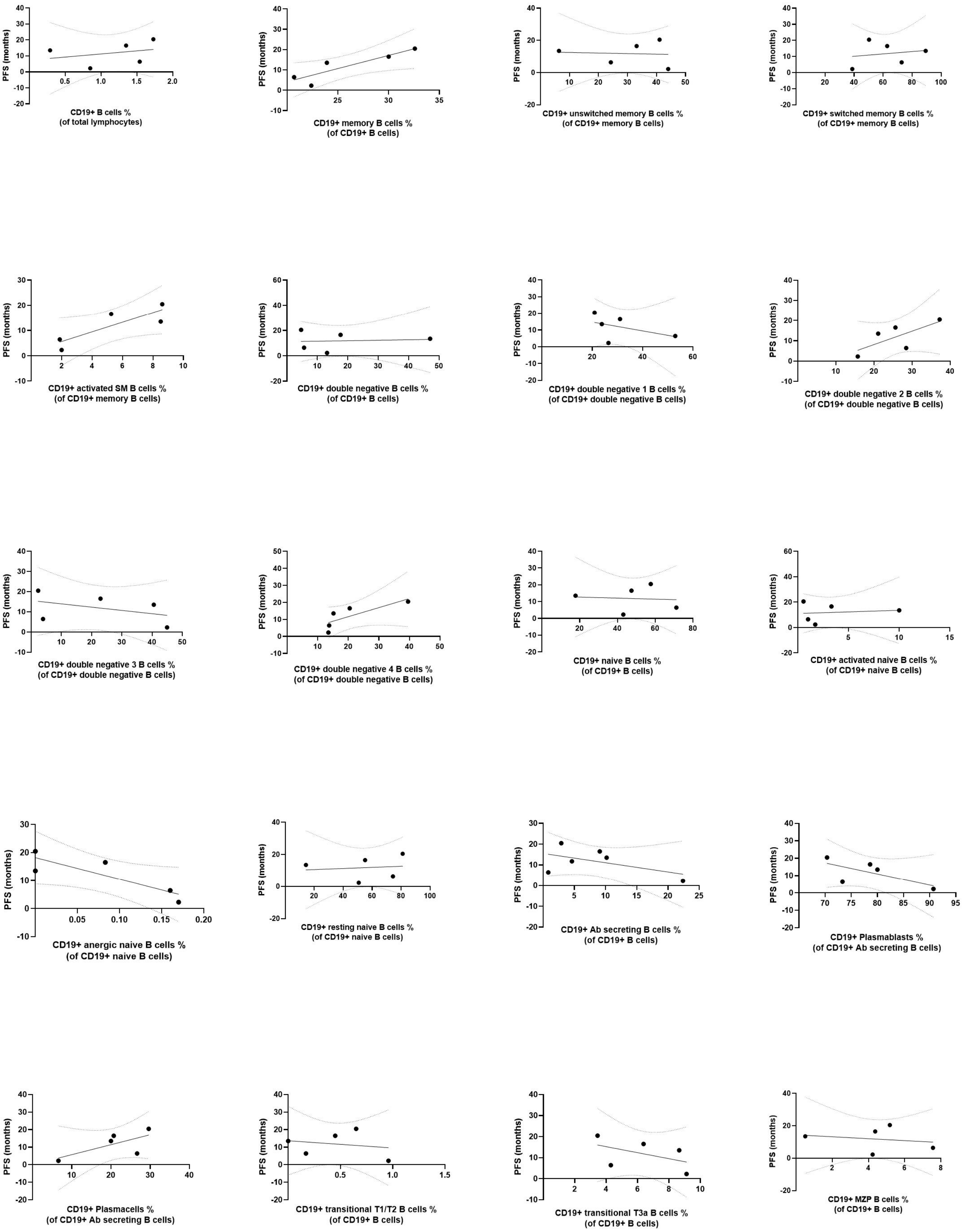
PFS (months)
CD19+ B cells %
(of total lymphocytes)
CD19+ memory B cells %
(of CD19+ B cells)
CD19+ unswitched memory B cells %
(of CD19+ memory B cells)
CD19+ switched memory B cells %
(of CD19+ memory B cells)
CD19+ activated SM B cells %
(of CD19+ memory B cells)
CD19+ double negative B cells %
(of CD19+ B cells)
CD19+ double negative 1 B cells %
(of CD19+ double negative B cells)
CD19+ double negative 2 B cells %
(of CD19+ double negative B cells)
CD19+ double negative 3 B cells %
(of CD19+ double negative B cells)
CD19+ double negative 4 B cells %
(of CD19+ double negative B cells)
CD19+ naive B cells %
(of CD19+ B cells)
CD19+ activated naive B cells %
(of CD19+ naive B cells)
CD19+ anergic naive B cells %
(of CD19+ naive B cells)
CD19+ resting naive B cells %
(of CD19+ naive B cells)
CD19+ Ab secreting B cells %
(of CD19+ B cells)
CD19+ Plasmablasts %
(of CD19+ Ab secreting B cells)
CD19+ Plasmacells %
(of CD19+ Ab secreting B cells)
CD19+ transitional T1/T2 B cells %
(of CD19+ B cells)
CD19+ transitional T3a B cells %
(of CD19+ B cells)
CD19+ MZP B cells %
(of CD19+ B cells)

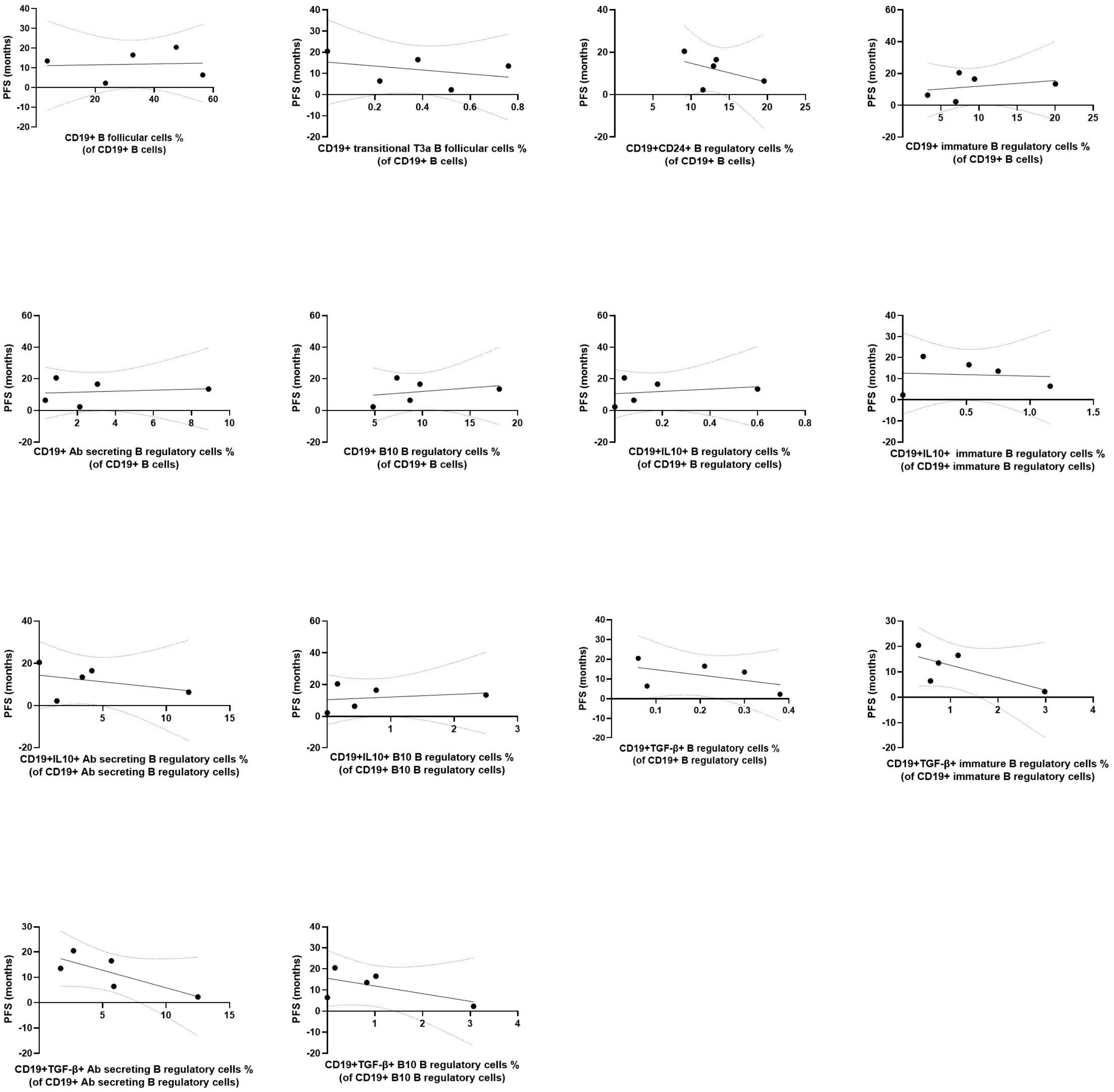
PFS (months)
CD19+ B follicular cells %
(of CD19+ B cells)
CD19+ transitional T3a B follicular cells %
(of CD19+ B cells)
CD19+CD24+ B regulatory cells %
(of CD19+ B cells)
CD19+ immature B regulatory cells %
(of CD19+ B cells)
CD19+ Ab secreting B regulatory cells %
(of CD19+ B cells)
CD19+ B10 B regulatory cells %
(of CD19+ B cells)
CD19+IL10+ B regulatory cells %
(of CD19+ B regulatory cells)
CD19+IL10+ immature B regulatory cells %
(of CD19+ immature B regulatory cells)
CD19+IL10+ Ab secreting B regulatory cells %
(of CD19+ Ab secreting B regulatory cells)
CD19+IL10+ B10 B regulatory cells %
(of CD19+ B10 B regulatory cells)
CD19+TGF-β+ B regulatory cells %
(of CD19+ B regulatory cells)
CD19+TGF-β+ immature B regulatory cells %
(of CD19+ immature B regulatory cells)
CD19+TGF-β+ Ab secreting B regulatory cells %
(of CD19+ Ab secreting B regulatory cells)
CD19+TGF-β+ B10 B regulatory cells %
(of CD19+ B10 B regulatory cells)

C

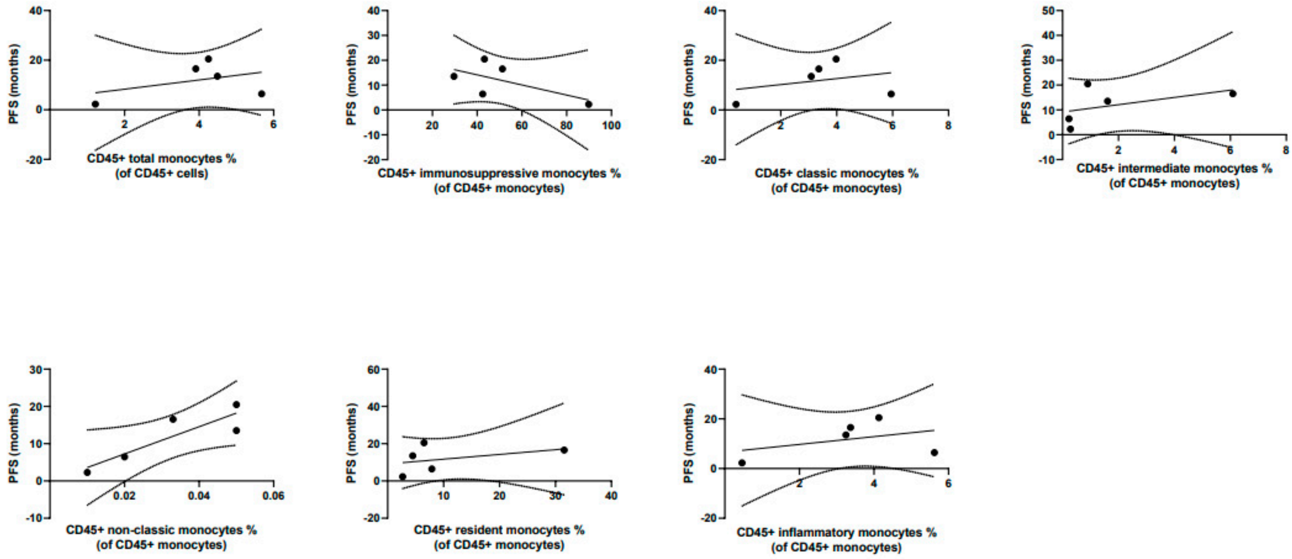
PFS (months)
CD45+ total monocytes %
(of CD45+ cells)
CD45+ immunosuppressive monocytes %
(of CD45+ monocytes)
CD45+ classic monocytes %
(of CD45+ monocytes)
CD45+ intermediate monocytes %
(of CD45+ monocytes)
CD45+ non-classic monocytes %
(of CD45+ monocytes)
CD45+ resident monocytes %
(of CD45+ monocytes)
CD45+ inflammatory monocytes %
(of CD45+ monocytes)

D

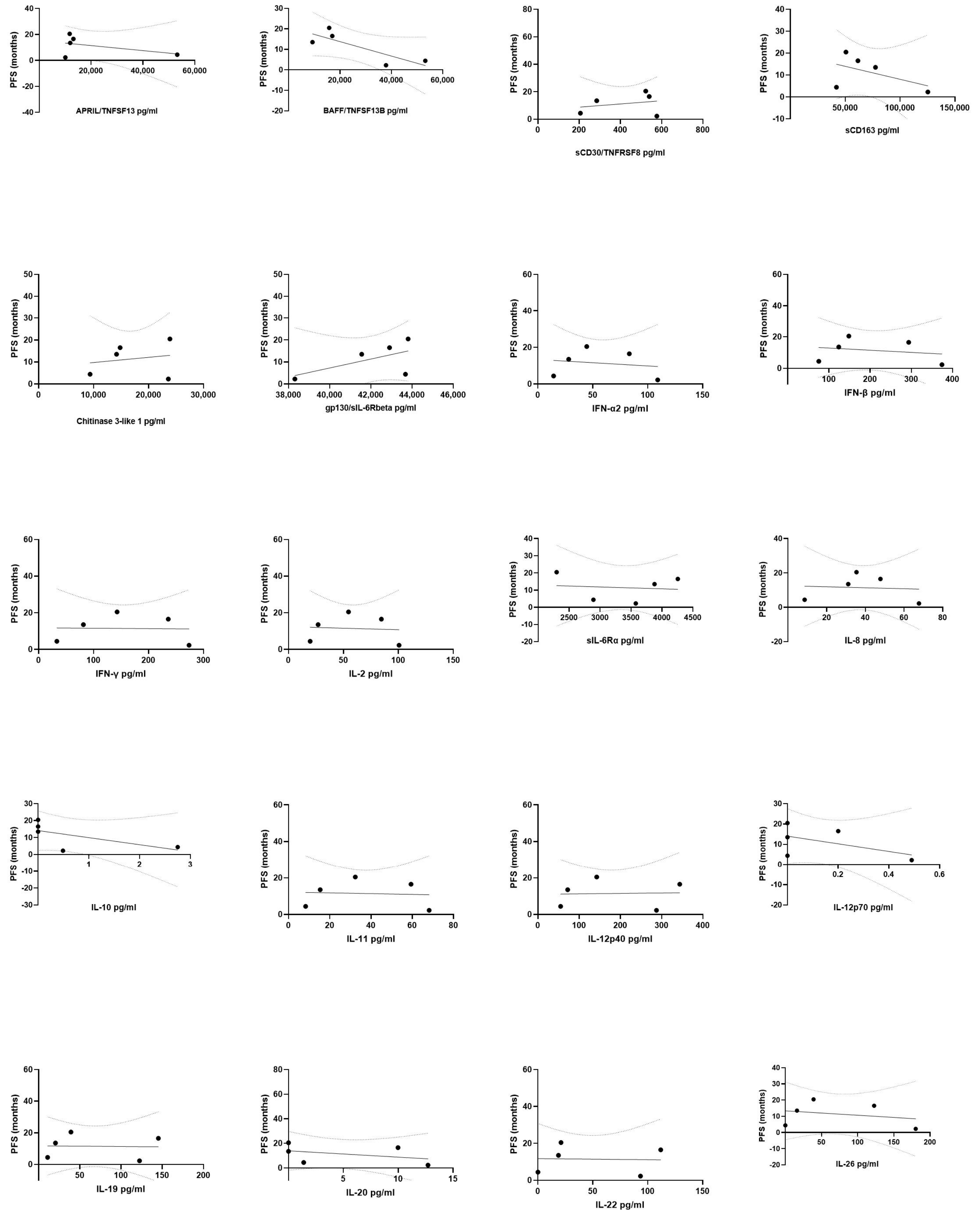
PFS (months)
APRIL/TNFSF13 pg/ml
BAFF/TNFSF13B pg/ml
sCD30/TNFRSF8 pg/ml
sCD163 pg/ml
Chitinase 3-like 1 pg/ml
gp130/sIL-6Rbeta pg/ml
IFN-α2 pg/ml
IFN-β pg/ml
IFN-γ pg/ml
IL-2 pg/ml
sIL-6Rα pg/ml
IL-8 pg/ml
IL-10 pg/ml
IL-11 pg/ml
IL-12p40 pg/ml
IL-12p70 pg/ml
IL-19 pg/ml
IL-20 pg/ml
IL-22 pg/ml
IL-26 pg/ml

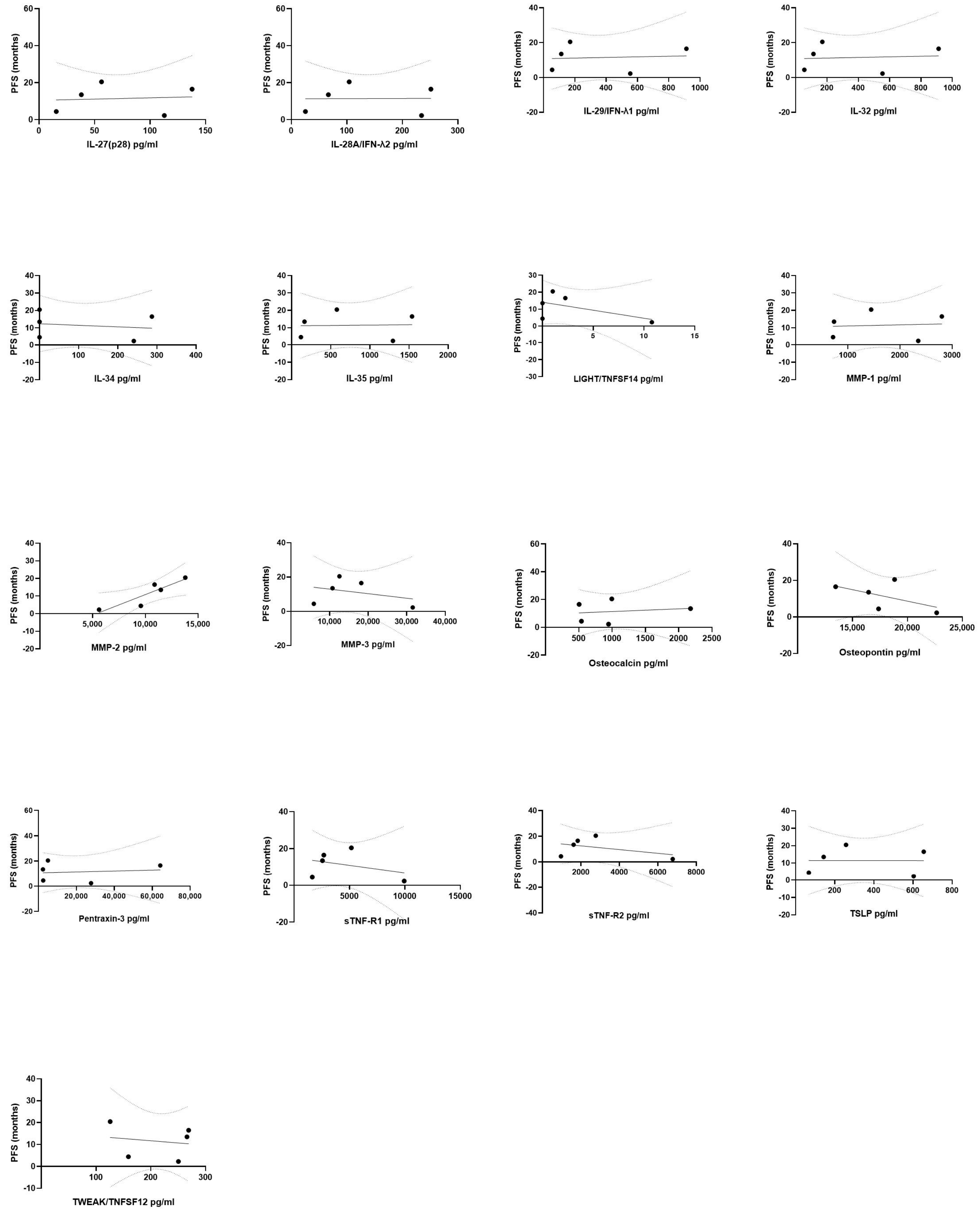
PFS (months)
IL-27(p28) pg/ml
IL-28A/IFN-λ2 pg/ml
IL-29/IFN-λ1 pg/ml
IL-32 pg/ml
IL-34 pg/ml
IL-35 pg/ml
LIGHT/TNFSF14 pg/ml
MMP-1 pg/ml
MMP-2 pg/ml
MMP-3 pg/ml
Osteocalcin pg/ml
Osteopontin pg/ml
Pentraxin-3 pg/ml
sTNF-R1 pg/ml
sTNF-R2 pg/ml
TSLP pg/ml
TWEAK/TNFSF12 pg/ml

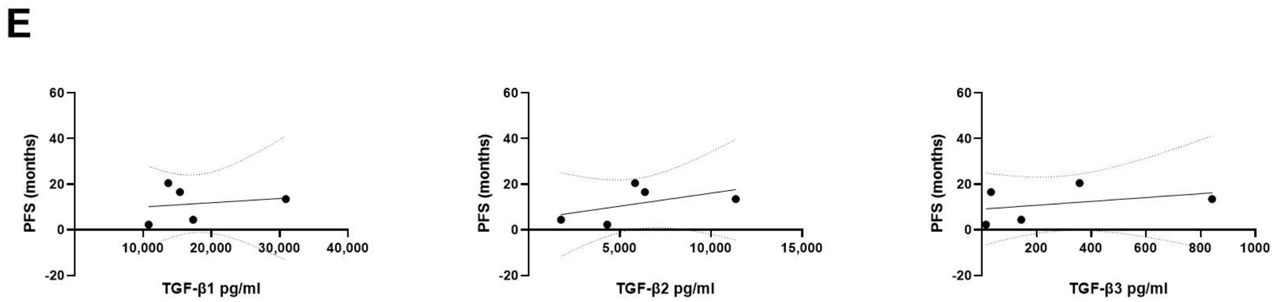
E
PFS (months)
60
40
20
0
-20
10,000
20,000
30,000
40,000
TGF-β1 pg/ml
5,000
10,000
15,000
TGF-β2 pg/ml
200
400
600
800
1000
TGF-β3 pg/ml

397

F

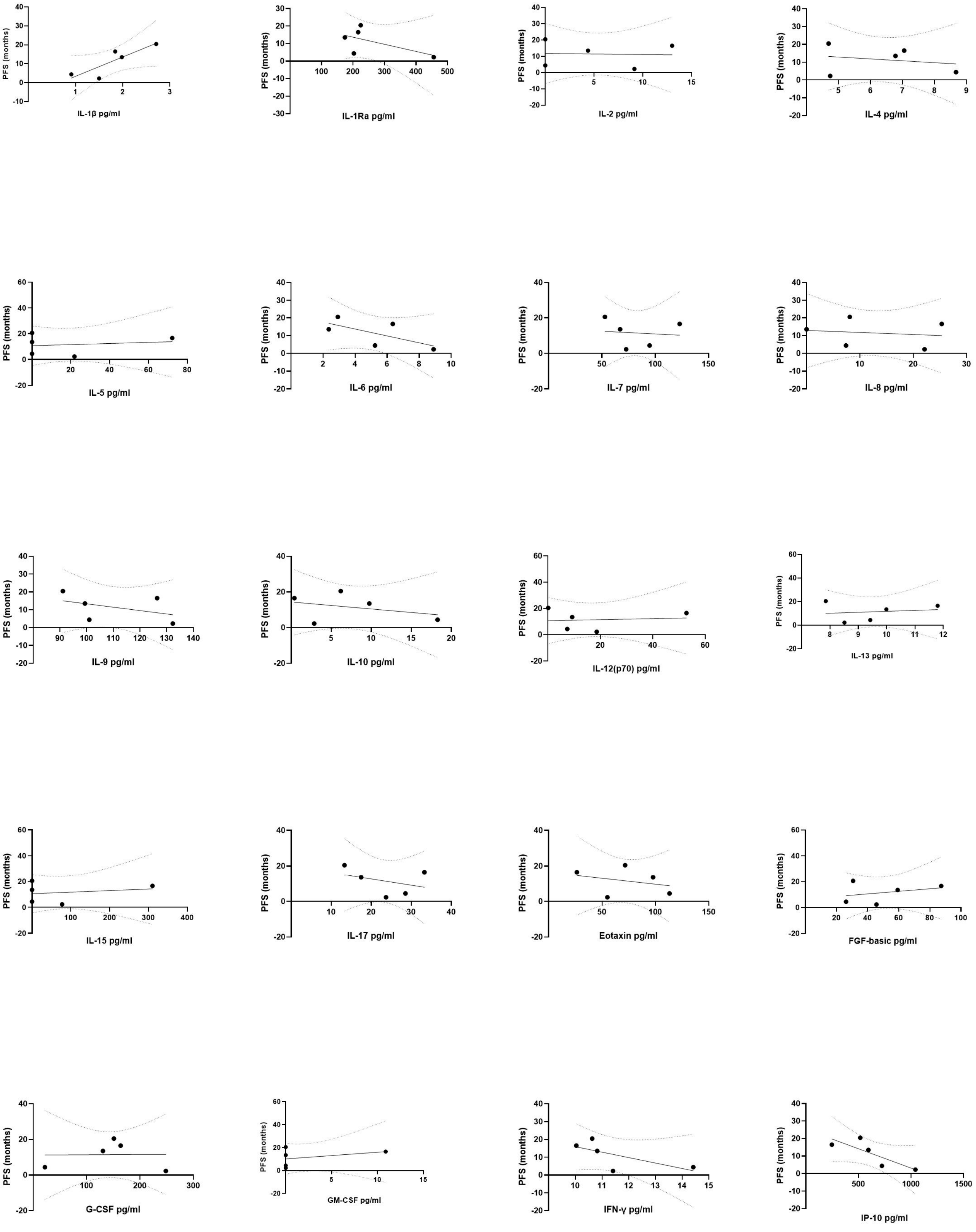
PFS (months)
IL-1β pg/ml
IL-1Ra pg/ml
IL-2 pg/ml
IL-4 pg/ml
IL-5 pg/ml
IL-6 pg/ml
IL-7 pg/ml
IL-8 pg/ml
IL-9 pg/ml
IL-10 pg/ml
IL-12(p70) pg/ml
IL-13 pg/ml
IL-15 pg/ml
IL-17 pg/ml
Eotaxin pg/ml
FGF-basic pg/ml
G-CSF pg/ml
GM-CSF pg/ml
IFN-γ pg/ml
IP-10 pg/ml

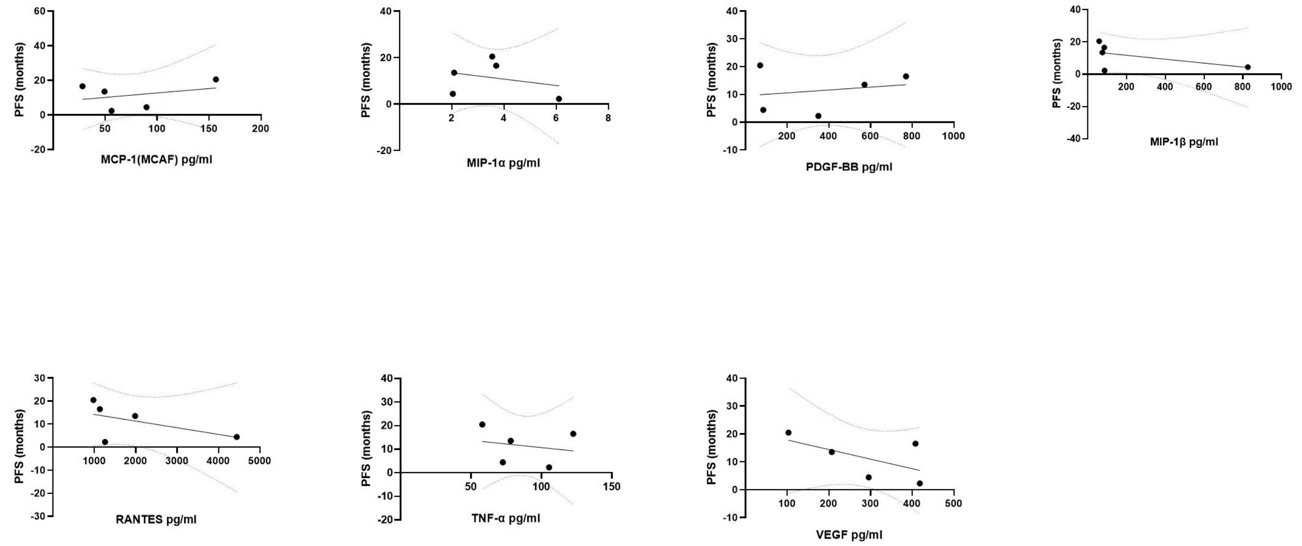
PFS (months)
MCP-1(MCAF) pg/ml
MIP-1α pg/ml
PDGF-BB pg/ml
MIP-1β pg/ml
RANTES pg/ml
TNF-α pg/ml
VEGF pg/ml

G

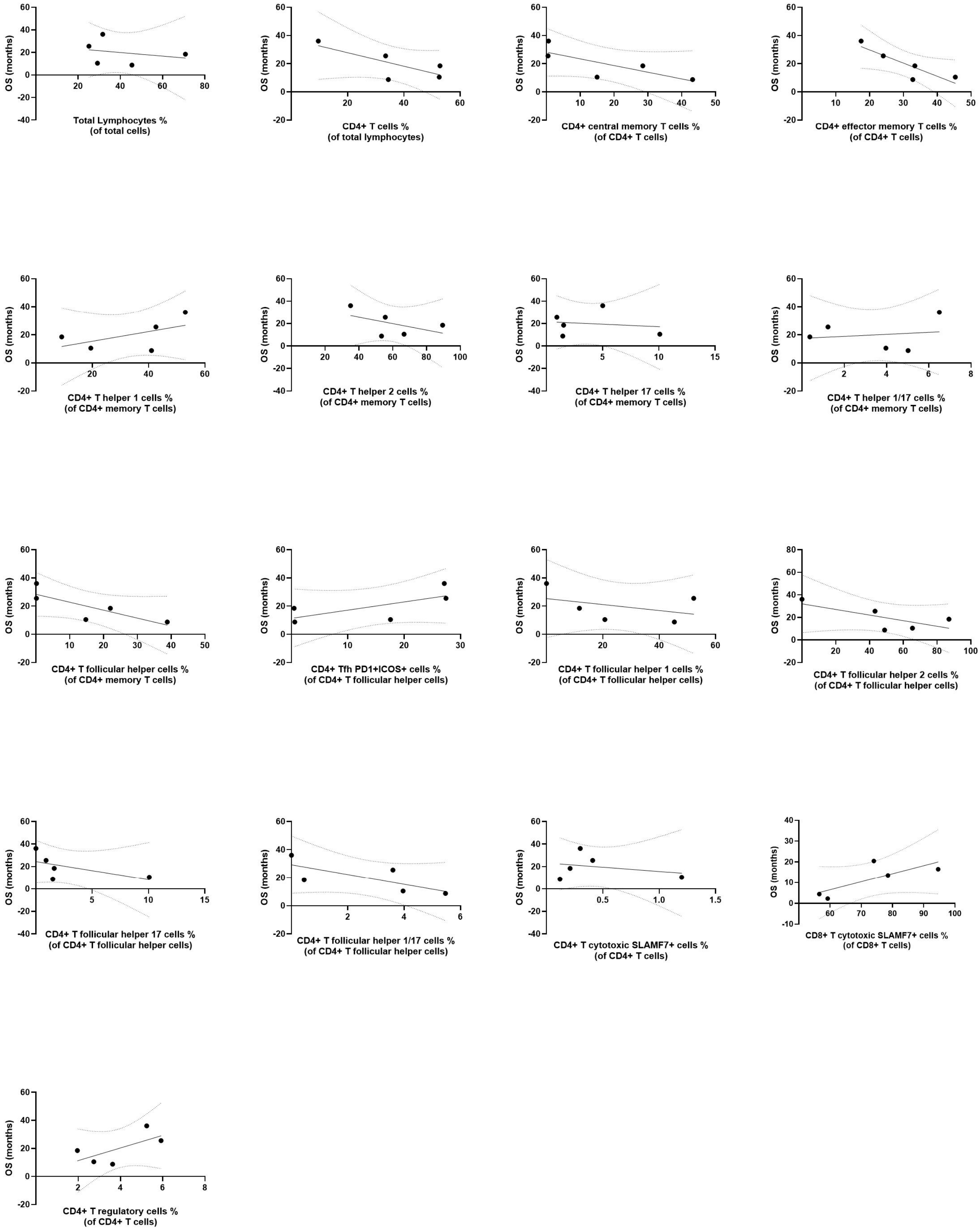
OS (months)
Total Lymphocytes % (of total cells)
CD4+ T cells % (of total lymphocytes)
CD4+ central memory T cells % (of CD4+ T cells)
CD4+ effector memory T cells % (of CD4+ T cells)
CD4+ T helper 1 cells % (of CD4+ memory T cells)
CD4+ T helper 2 cells % (of CD4+ memory T cells)
CD4+ T helper 17 cells % (of CD4+ memory T cells)
CD4+ T helper 1/17 cells % (of CD4+ memory T cells)
CD4+ T follicular helper cells % (of CD4+ memory T cells)
CD4+ Tfh PD1+ICOS+ cells % (of CD4+ T follicular helper cells)
CD4+ T follicular helper 1 cells % (of CD4+ T follicular helper cells)
CD4+ T follicular helper 2 cells % (of CD4+ T follicular helper cells)
CD4+ T follicular helper 17 cells % (of CD4+ T follicular helper cells)
CD4+ T follicular helper 1/17 cells % (of CD4+ T follicular helper cells)
CD4+ T cytotoxic SLAMF7+ cells % (of CD4+ T cells)
CD8+ T cytotoxic SLAMF7+ cells % (of CD8+ T cells)
CD4+ T regulatory cells % (of CD4+ T cells)

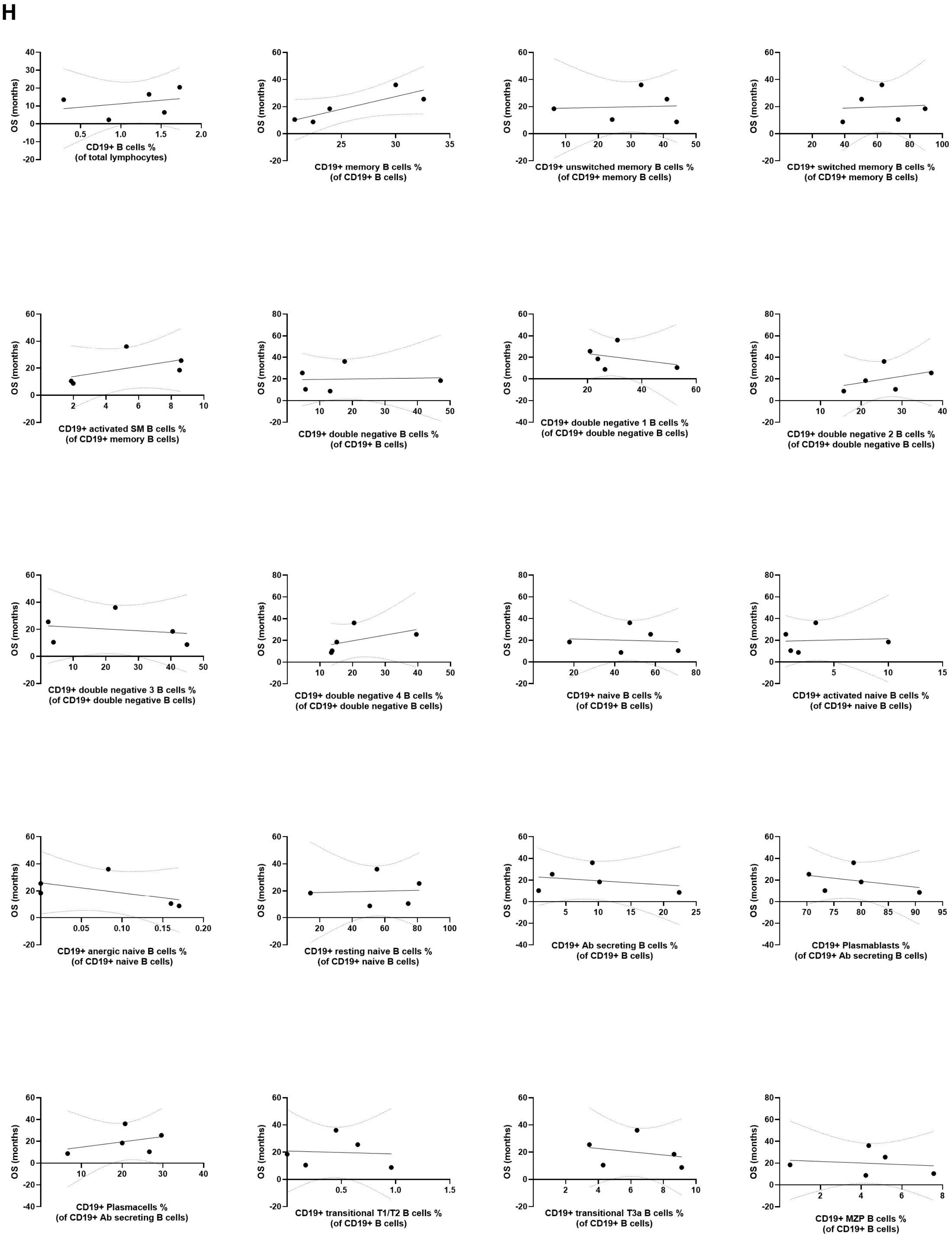
H
OS (months)
CD19+ B cells %
(of total lymphocytes)
CD19+ memory B cells %
(of CD19+ B cells)
CD19+ unswitched memory B cells %
(of CD19+ memory B cells)
CD19+ switched memory B cells %
(of CD19+ memory B cells)
CD19+ activated SM B cells %
(of CD19+ memory B cells)
CD19+ double negative B cells %
(of CD19+ B cells)
CD19+ double negative 1 B cells %
(of CD19+ double negative B cells)
CD19+ double negative 2 B cells %
(of CD19+ double negative B cells)
CD19+ double negative 3 B cells %
(of CD19+ double negative B cells)
CD19+ double negative 4 B cells %
(of CD19+ double negative B cells)
CD19+ naive B cells %
(of CD19+ B cells)
CD19+ activated naive B cells %
(of CD19+ naive B cells)
CD19+ anergic naive B cells %
(of CD19+ naive B cells)
CD19+ resting naive B cells %
(of CD19+ naive B cells)
CD19+ Ab secreting B cells %
(of CD19+ B cells)
CD19+ Plasmablasts %
(of CD19+ Ab secreting B cells)
CD19+ Plasmacells %
(of CD19+ Ab secreting B cells)
CD19+ transitional T1/T2 B cells %
(of CD19+ B cells)
CD19+ transitional T3a B cells %
(of CD19+ B cells)
CD19+ MZP B cells %
(of CD19+ B cells)

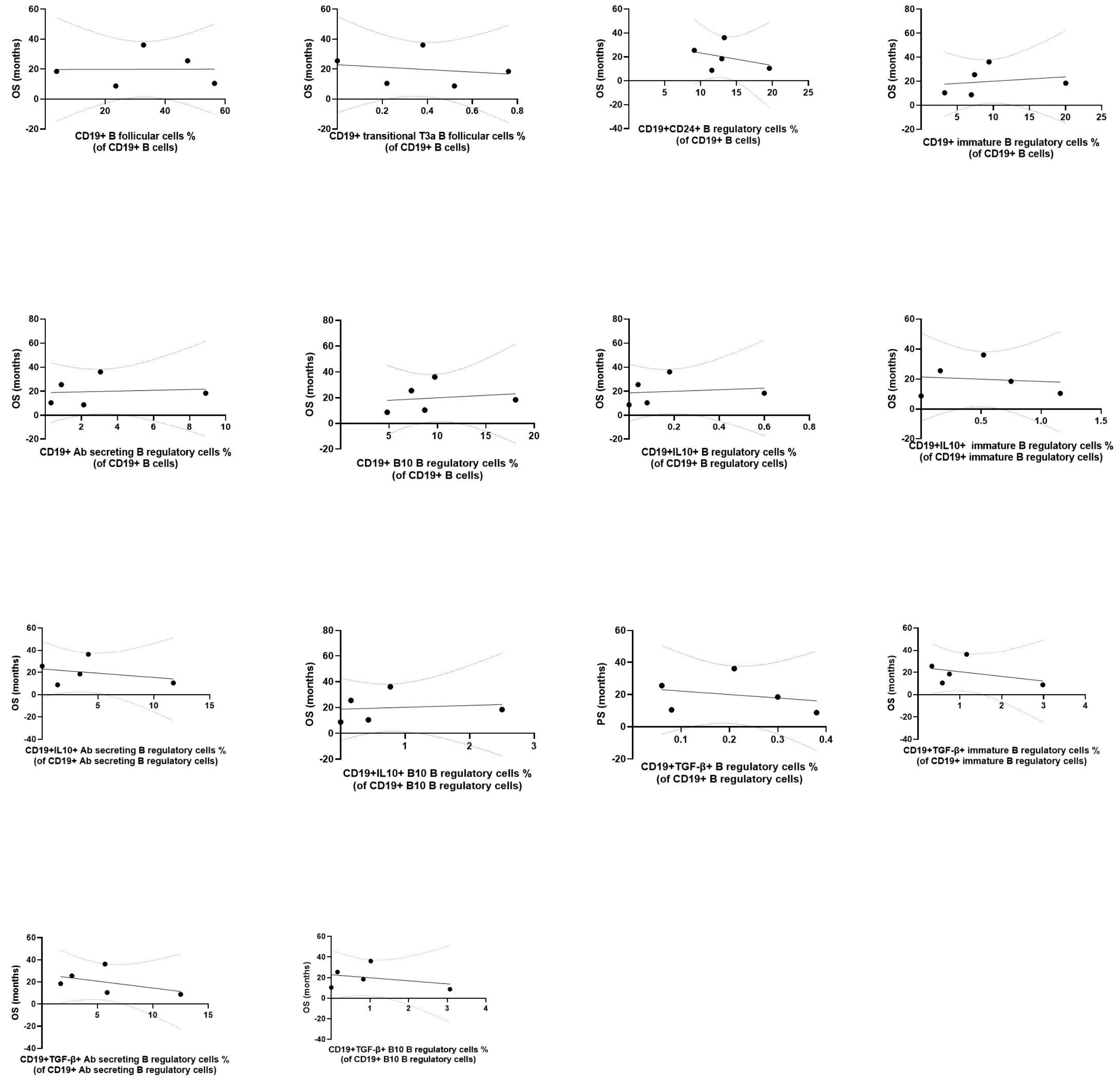
OS (months)
CD19+ B follicular cells %
(of CD19+ B cells)
OS (months)
CD19+ transitional T3a B follicular cells %
(of CD19+ B cells)
OS (months)
CD19+CD24+ B regulatory cells %
(of CD19+ B cells)
OS (months)
CD19+ immature B regulatory cells %
(of CD19+ B cells)
OS (months)
CD19+ Ab secreting B regulatory cells %
(of CD19+ B cells)
OS (months)
CD19+ B10 B regulatory cells %
(of CD19+ B cells)
OS (months)
CD19+IL10+ B regulatory cells %
(of CD19+ B regulatory cells)
OS (months)
CD19+IL10+ immature B regulatory cells %
(of CD19+ immature B regulatory cells)
OS (months)
CD19+IL10+ Ab secreting B regulatory cells %
(of CD19+ Ab secreting B regulatory cells)
OS (months)
CD19+IL10+ B10 B regulatory cells %
(of CD19+ B10 B regulatory cells)
PS (months)
CD19+TGF-β+ B regulatory cells %
(of CD19+ B regulatory cells)
OS (months)
CD19+TGF-β+ immature B regulatory cells %
(of CD19+ immature B regulatory cells)
OS (months)
CD19+TGF-β+ Ab secreting B regulatory cells %
(of CD19+ Ab secreting B regulatory cells)
OS (months)
CD19+TGF-β+ B10 B regulatory cells %
(of CD19+ B10 B regulatory cells)

I

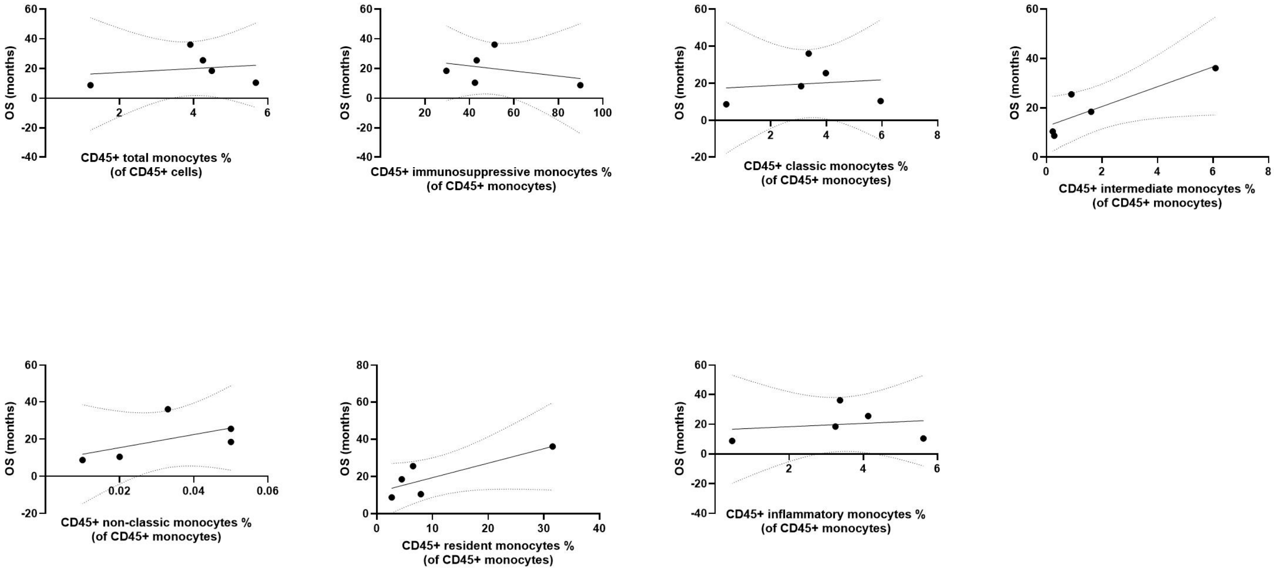

OS (months)
CD45+ total monocytes %
(of CD45+ cells)
CD45+ immunosuppressive monocytes %
(of CD45+ monocytes)
CD45+ classic monocytes %
(of CD45+ monocytes)
CD45+ intermediate monocytes %
(of CD45+ monocytes)
CD45+ non-classic monocytes %
(of CD45+ monocytes)
CD45+ resident monocytes %
(of CD45+ monocytes)
CD45+ inflammatory monocytes %
(of CD45+ monocytes)

J

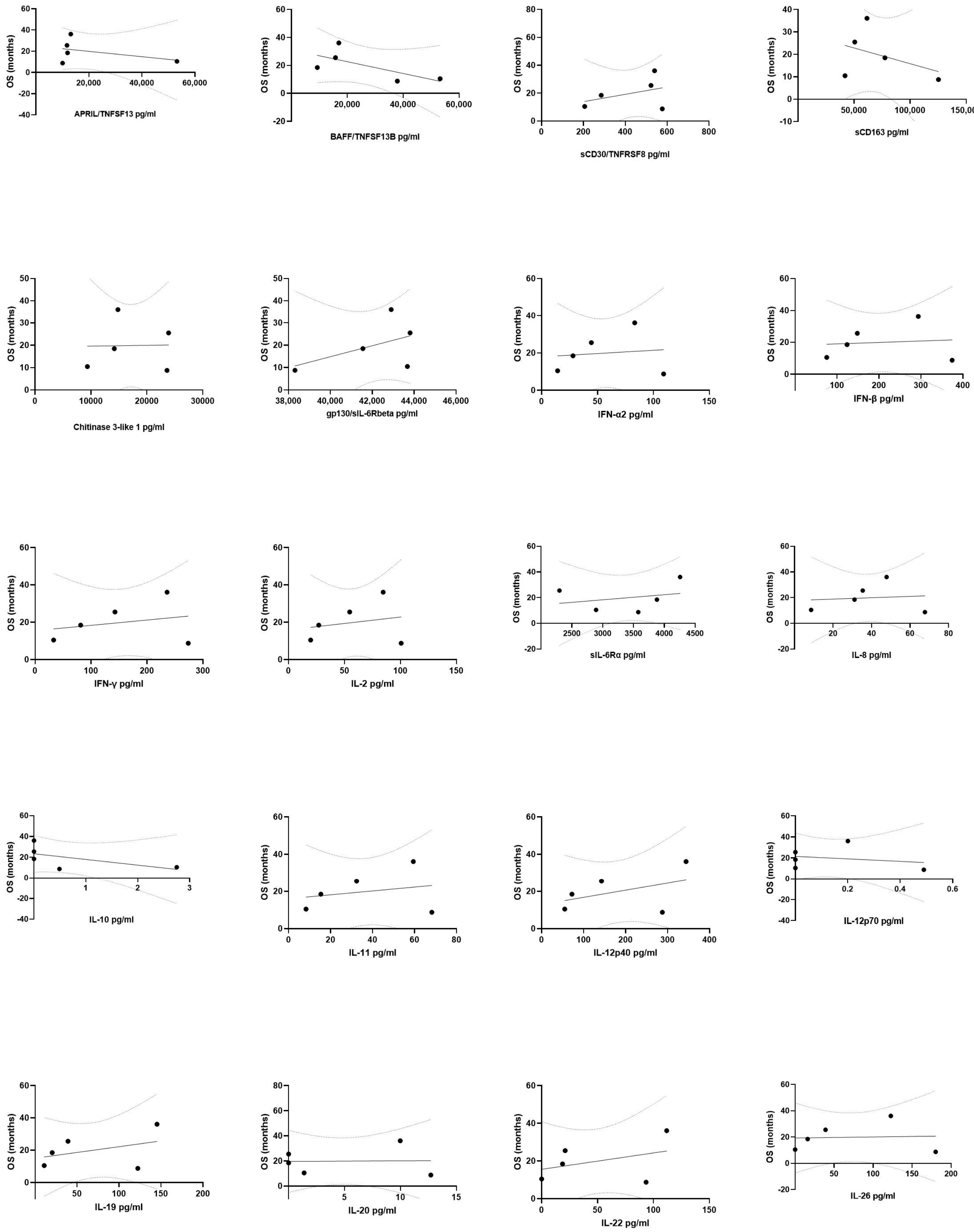
OS (months)
APRIL/TNFSF13 pg/ml
BAFF/TNFSF13B pg/ml
sCD30/TNFRSF8 pg/ml
sCD163 pg/ml
Chitinase 3-like 1 pg/ml
gp130/sIL-6Rbeta pg/ml
IFN-α2 pg/ml
IFN-β pg/ml
IFN-γ pg/ml
IL-2 pg/ml
sIL-6Rα pg/ml
IL-8 pg/ml
IL-10 pg/ml
IL-11 pg/ml
IL-12p40 pg/ml
IL-12p70 pg/ml
IL-19 pg/ml
IL-20 pg/ml
IL-22 pg/ml
IL-26 pg/ml

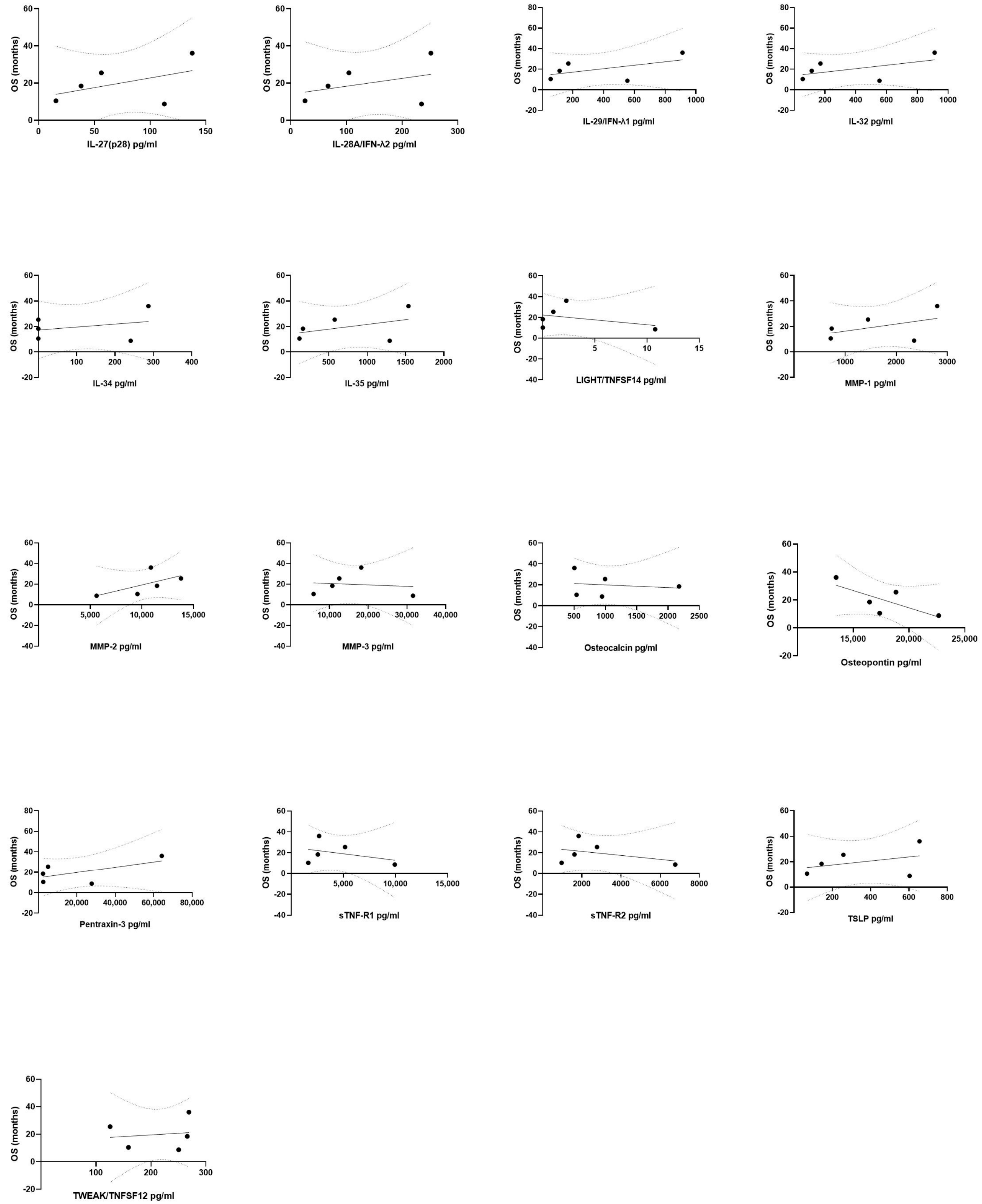
OS (months)
IL-27(p28) pg/ml
IL-28A/IFN-λ2 pg/ml
IL-29/IFN-λ1 pg/ml
IL-32 pg/ml
IL-34 pg/ml
IL-35 pg/ml
LIGHT/TNFSF14 pg/ml
MMP-1 pg/ml
MMP-2 pg/ml
MMP-3 pg/ml
Osteocalcin pg/ml
Osteopontin pg/ml
Pentraxin-3 pg/ml
sTNF-R1 pg/ml
sTNF-R2 pg/ml
TSLP pg/ml
TWEAK/TNFSF12 pg/ml

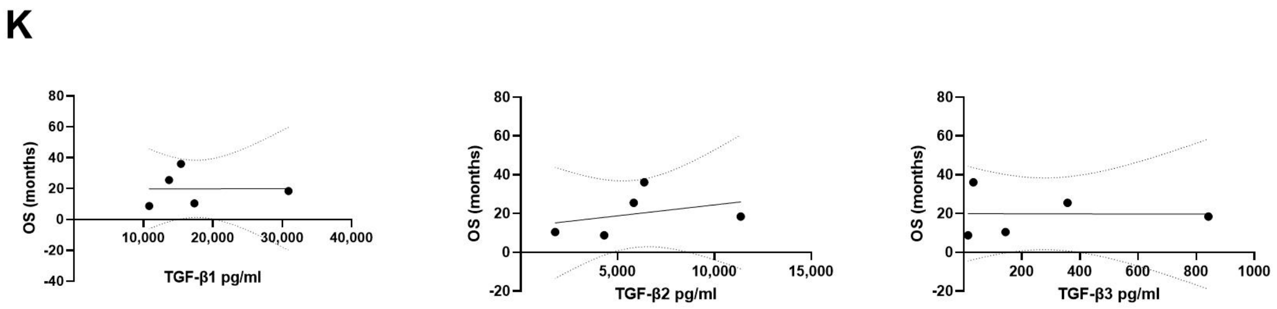
K
OS (months)
TGF-β1 pg/ml
10,000
20,000
30,000
40,000
TGF-β2 pg/ml
5,000
10,000
15,000
TGF-β3 pg/ml
200
400
600
800
1000
80
60
40
20
0
-20
-40

412

L

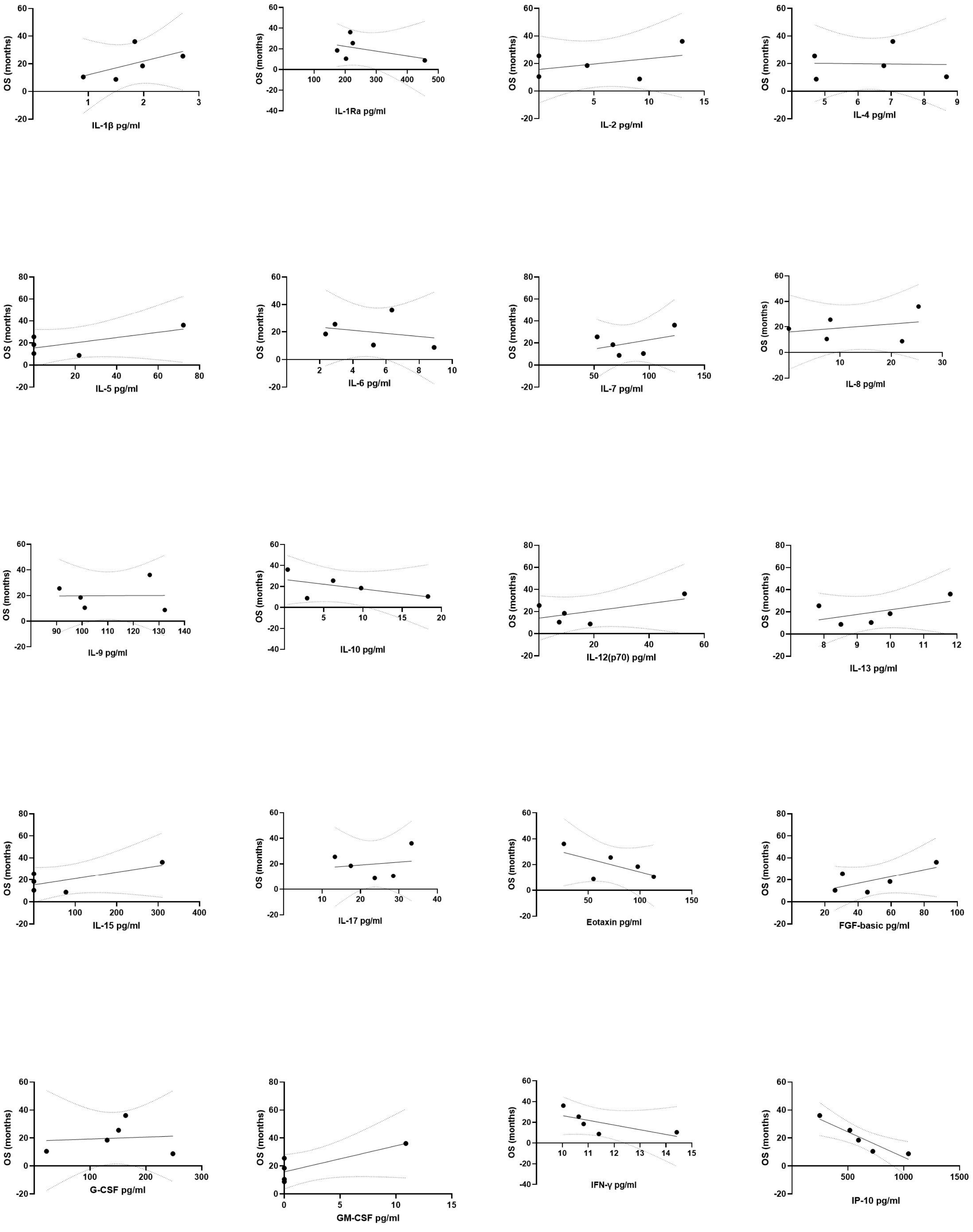
OS (months)
IL-1β pg/ml
IL-1Ra pg/ml
IL-2 pg/ml
IL-4 pg/ml
IL-5 pg/ml
IL-6 pg/ml
IL-7 pg/ml
IL-8 pg/ml
IL-9 pg/ml
IL-10 pg/ml
IL-12(p70) pg/ml
IL-13 pg/ml
IL-15 pg/ml
IL-17 pg/ml
Eotaxin pg/ml
FGF-basic pg/ml
G-CSF pg/ml
GM-CSF pg/ml
IFN-γ pg/ml
IP-10 pg/ml

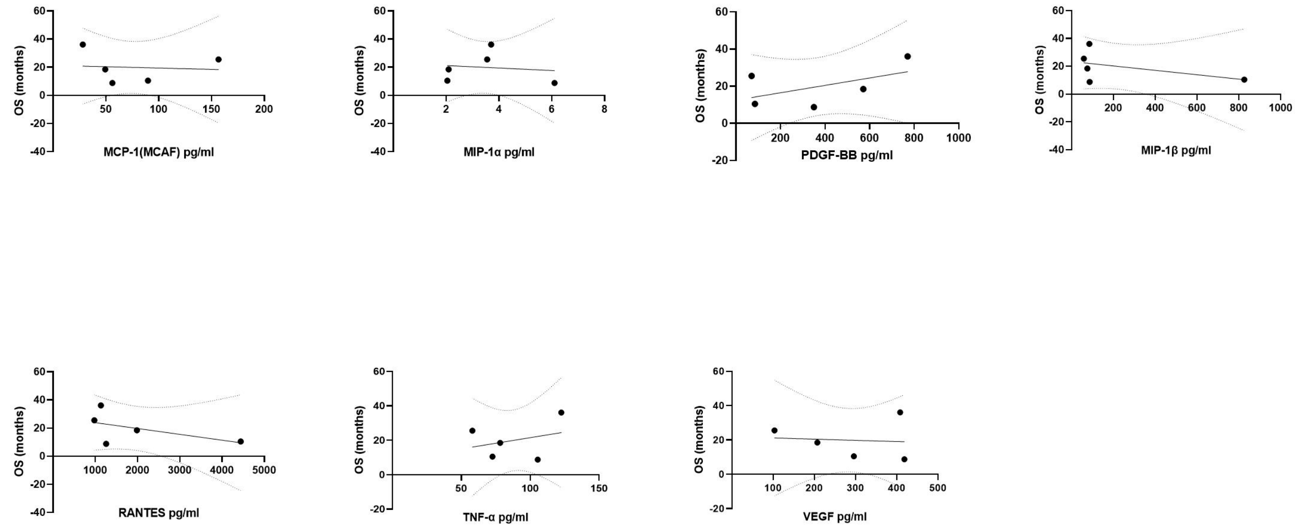

Footnote: Te: T effector; Th1: T helper 1; Th2: T helper 2; Th17: T helper 17; Th1/17: T helper 1/17; Tfh: T follicular helper; Tfh1: T follicular helper 1; Tfh2: T follicular helper 2; Tfh17: T follicular helper 17; Tfh1/17: T follicular helper 1/17; Treg: T regulatory; UM: unswitched memory; SM: switched memory; ASM: activated switched memory; DN: double negative; DN1: double negative 1; DN2: double negative 2; DN3: double negative 3; DN4: double negative 4; AcN: activated naïve; AnN: anergic naïve; RN: resting naïve; Ab: antibody secreting; T1/2: transitional 1/2; T3a: transitional 3a; MZP: marginal zone peripheral; Breg: B regulatory; AbS: antibody secreting; Imm: immature; Mon: monocytes.

Figure S10. Correlation studies between circulating immune cells (A, G: T-cell compartment; B, H: B-cell compartment; C, I: monocyte compartment) and concentrations of inflammatory and immune-related cytokines and chemokines (D, J: Pro Human Inflammation Panel I Assay; E, K: Pro TGF-β Immunoassay; F, L: Pro Human Cytokine Immunoassay) at 4-month follow-up and progression-free survival (PFS) (A-F) and overall survival (OS) (G-L) after therapy onset in patients with pancreatic adenocarcinoma (PDAC) of the chemotherapy (CT) arm. Correlation studies were performed using the Spearman's rho rank correlation test.

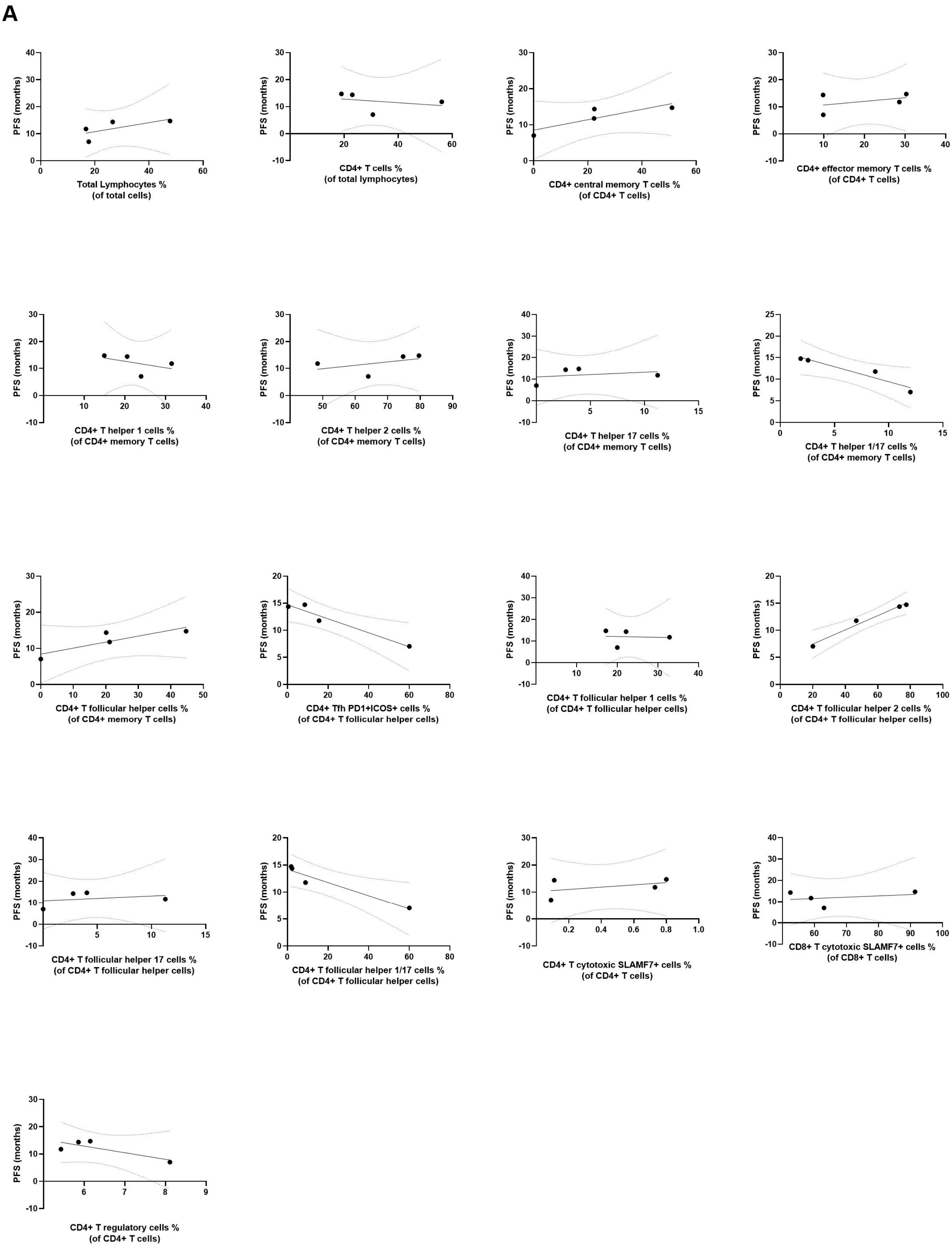
A
PFS (months)
Total Lymphocytes %
(of total cells)
CD4+ T cells %
(of total lymphocytes)
CD4+ central memory T cells %
(of CD4+ T cells)
CD4+ effector memory T cells %
(of CD4+ T cells)
CD4+ T helper 1 cells %
(of CD4+ memory T cells)
CD4+ T helper 2 cells %
(of CD4+ memory T cells)
CD4+ T helper 17 cells %
(of CD4+ memory T cells)
CD4+ T helper 1/17 cells %
(of CD4+ memory T cells)
CD4+ T follicular helper cells %
(of CD4+ memory T cells)
CD4+ Tfh PD1+ICOS+ cells %
(of CD4+ T follicular helper cells)
CD4+ T follicular helper 1 cells %
(of CD4+ T follicular helper cells)
CD4+ T follicular helper 2 cells %
(of CD4+ T follicular helper cells)
CD4+ T follicular helper 17 cells %
(of CD4+ T follicular helper cells)
CD4+ T follicular helper 1/17 cells %
(of CD4+ T follicular helper cells)
CD4+ T cytotoxic SLAMF7+ cells %
(of CD4+ T cells)
CD8+ T cytotoxic SLAMF7+ cells %
(of CD8+ T cells)
CD4+ T regulatory cells %
(of CD4+ T cells)

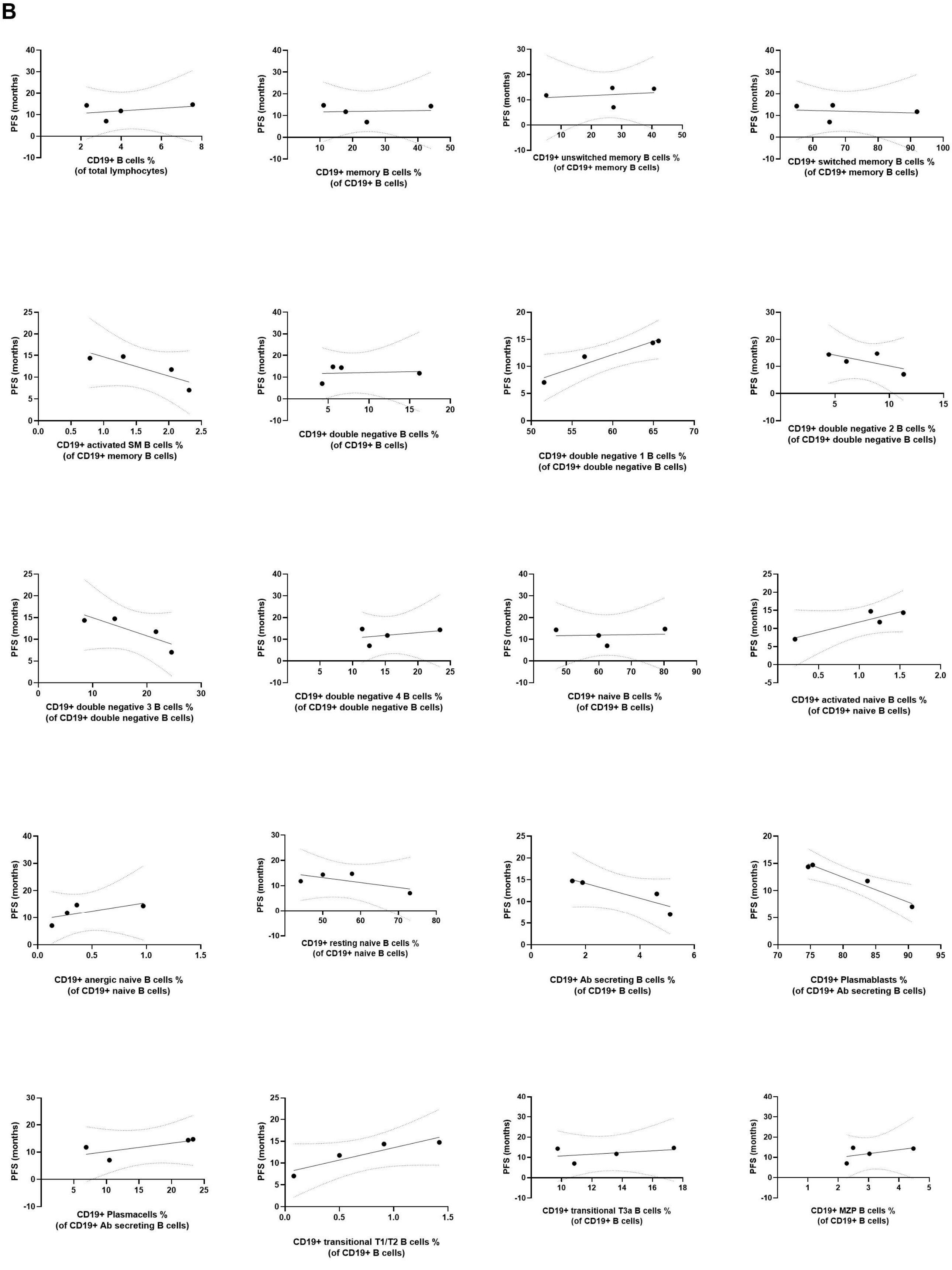
B
PFS (months)
CD19+ B cells %
(of total lymphocytes)
CD19+ memory B cells %
(of CD19+ B cells)
CD19+ unswitched memory B cells %
(of CD19+ memory B cells)
CD19+ switched memory B cells %
(of CD19+ memory B cells)
CD19+ activated SM B cells %
(of CD19+ memory B cells)
CD19+ double negative B cells %
(of CD19+ B cells)
CD19+ double negative 1 B cells %
(of CD19+ double negative B cells)
CD19+ double negative 2 B cells %
(of CD19+ double negative B cells)
CD19+ double negative 3 B cells %
(of CD19+ double negative B cells)
CD19+ double negative 4 B cells %
(of CD19+ double negative B cells)
CD19+ naive B cells %
(of CD19+ B cells)
CD19+ activated naive B cells %
(of CD19+ naive B cells)
CD19+ anergic naive B cells %
(of CD19+ naive B cells)
CD19+ resting naive B cells %
(of CD19+ naive B cells)
CD19+ Ab secreting B cells %
(of CD19+ B cells)
CD19+ Plasmablasts %
(of CD19+ Ab secreting B cells)
CD19+ Plasmacells %
(of CD19+ Ab secreting B cells)
CD19+ transitional T1/T2 B cells %
(of CD19+ B cells)
CD19+ transitional T3a B cells %
(of CD19+ B cells)
CD19+ MZP B cells %
(of CD19+ B cells)

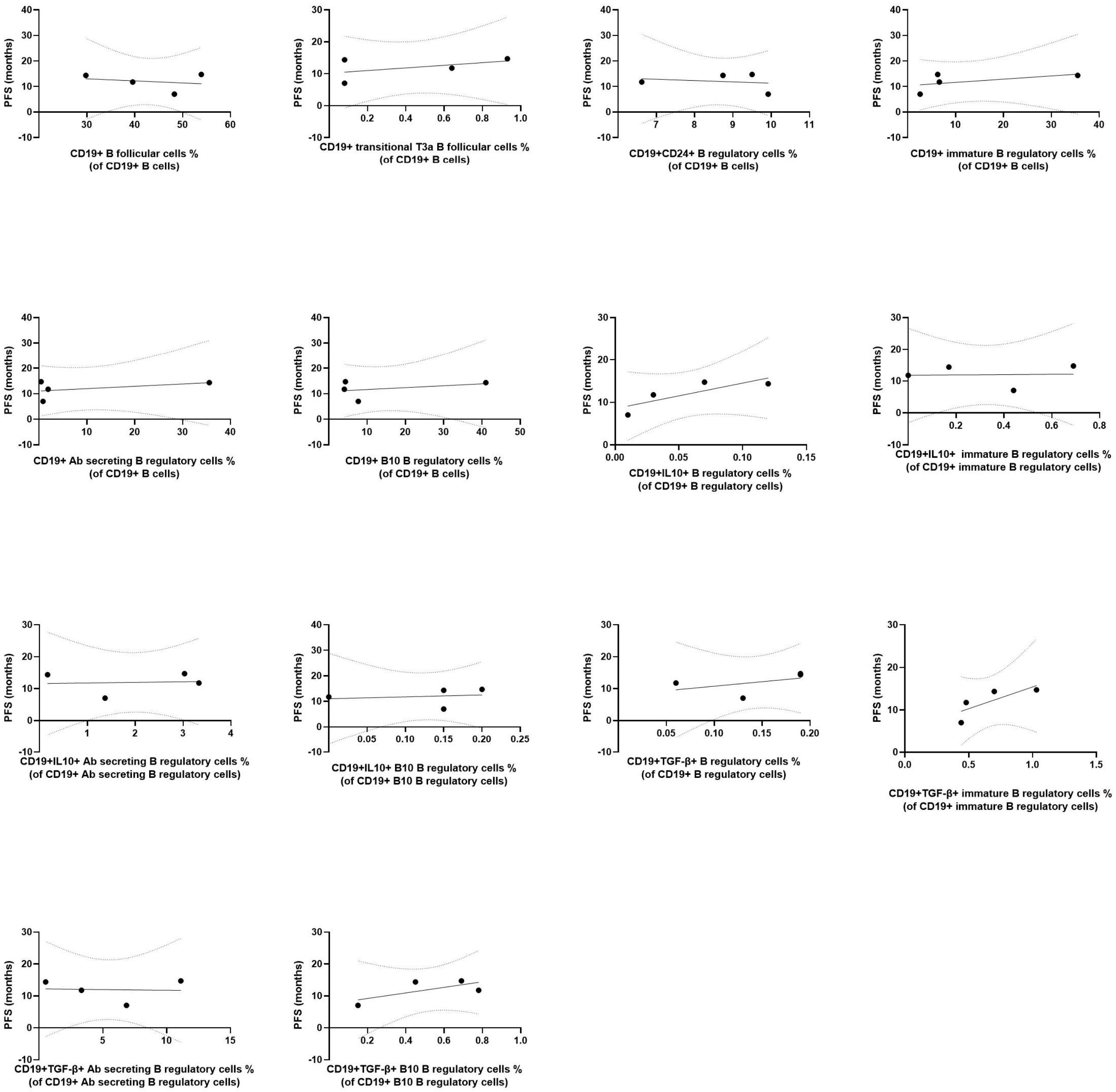
PFS (months)
CD19+ B follicular cells %
(of CD19+ B cells)
CD19+ transitional T3a B follicular cells %
(of CD19+ B cells)
CD19+CD24+ B regulatory cells %
(of CD19+ B cells)
CD19+ immature B regulatory cells %
(of CD19+ B cells)
CD19+ Ab secreting B regulatory cells %
(of CD19+ B cells)
CD19+ B10 B regulatory cells %
(of CD19+ B cells)
CD19+IL10+ B regulatory cells %
(of CD19+ B regulatory cells)
CD19+IL10+ immature B regulatory cells %
(of CD19+ immature B regulatory cells)
CD19+IL10+ Ab secreting B regulatory cells %
(of CD19+ Ab secreting B regulatory cells)
CD19+IL10+ B10 B regulatory cells %
(of CD19+ B10 B regulatory cells)
CD19+TGF-β+ B regulatory cells %
(of CD19+ B regulatory cells)
CD19+TGF-β+ immature B regulatory cells %
(of CD19+ immature B regulatory cells)
CD19+TGF-β+ Ab secreting B regulatory cells %
(of CD19+ Ab secreting B regulatory cells)
CD19+TGF-β+ B10 B regulatory cells %
(of CD19+ B10 B regulatory cells)

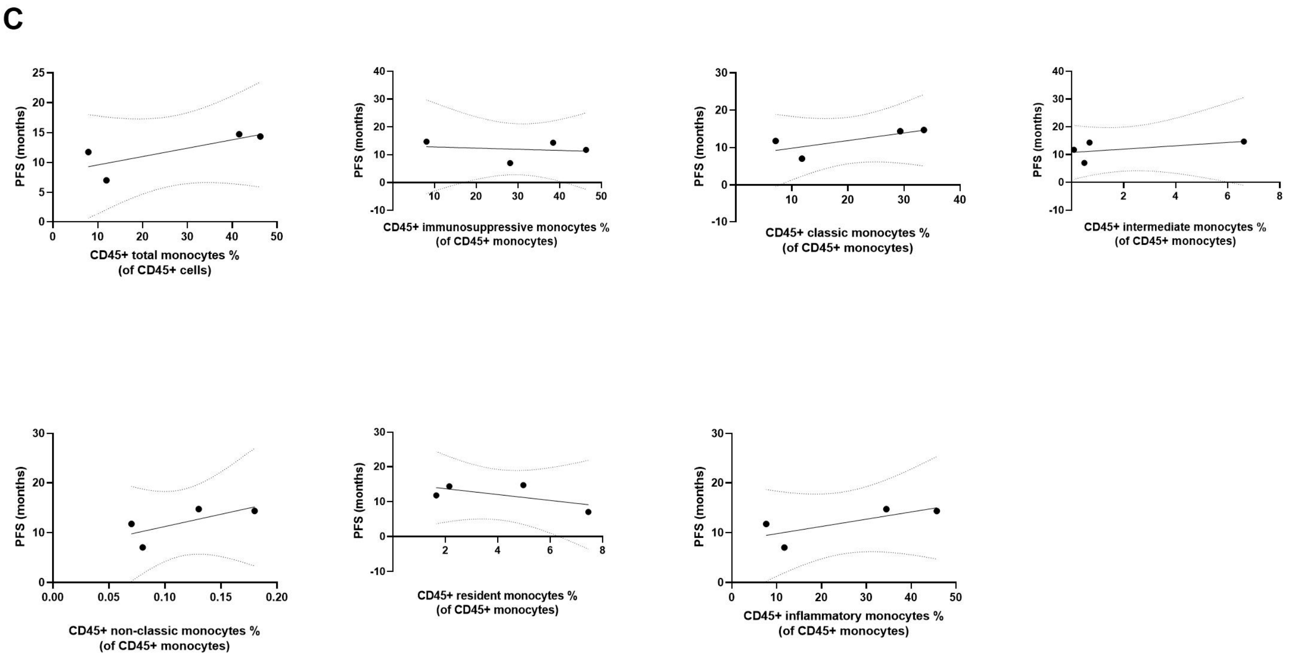
C
PFS (months)
CD45+ total monocytes %
(of CD45+ cells)
CD45+ immunosuppressive monocytes %
(of CD45+ monocytes)
CD45+ classic monocytes %
(of CD45+ monocytes)
CD45+ intermediate monocytes %
(of CD45+ monocytes)
CD45+ non-classic monocytes %
(of CD45+ monocytes)
CD45+ resident monocytes %
(of CD45+ monocytes)
CD45+ inflammatory monocytes %
(of CD45+ monocytes)

D

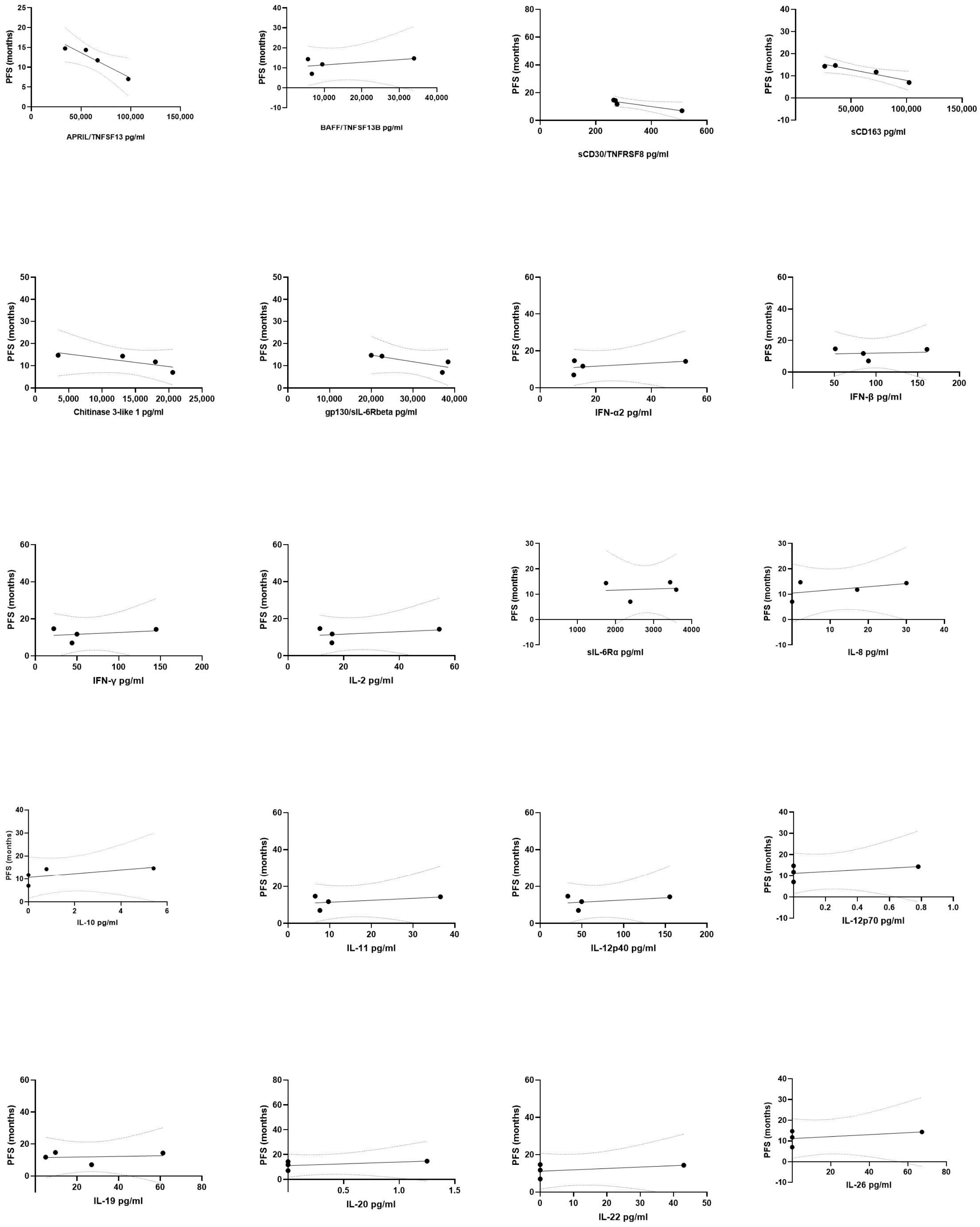
PFS (months)
APRIL/TNFSF13 pg/ml
BAFF/TNFSF13B pg/ml
sCD30/TNFRSF8 pg/ml
sCD163 pg/ml
Chitinase 3-like 1 pg/ml
gp130/sIL-6Rbeta pg/ml
IFN-α2 pg/ml
IFN-β pg/ml
IFN-γ pg/ml
IL-2 pg/ml
sIL-6Rα pg/ml
IL-8 pg/ml
IL-10 pg/ml
IL-11 pg/ml
IL-12p40 pg/ml
IL-12p70 pg/ml
IL-19 pg/ml
IL-20 pg/ml
IL-22 pg/ml
IL-26 pg/ml

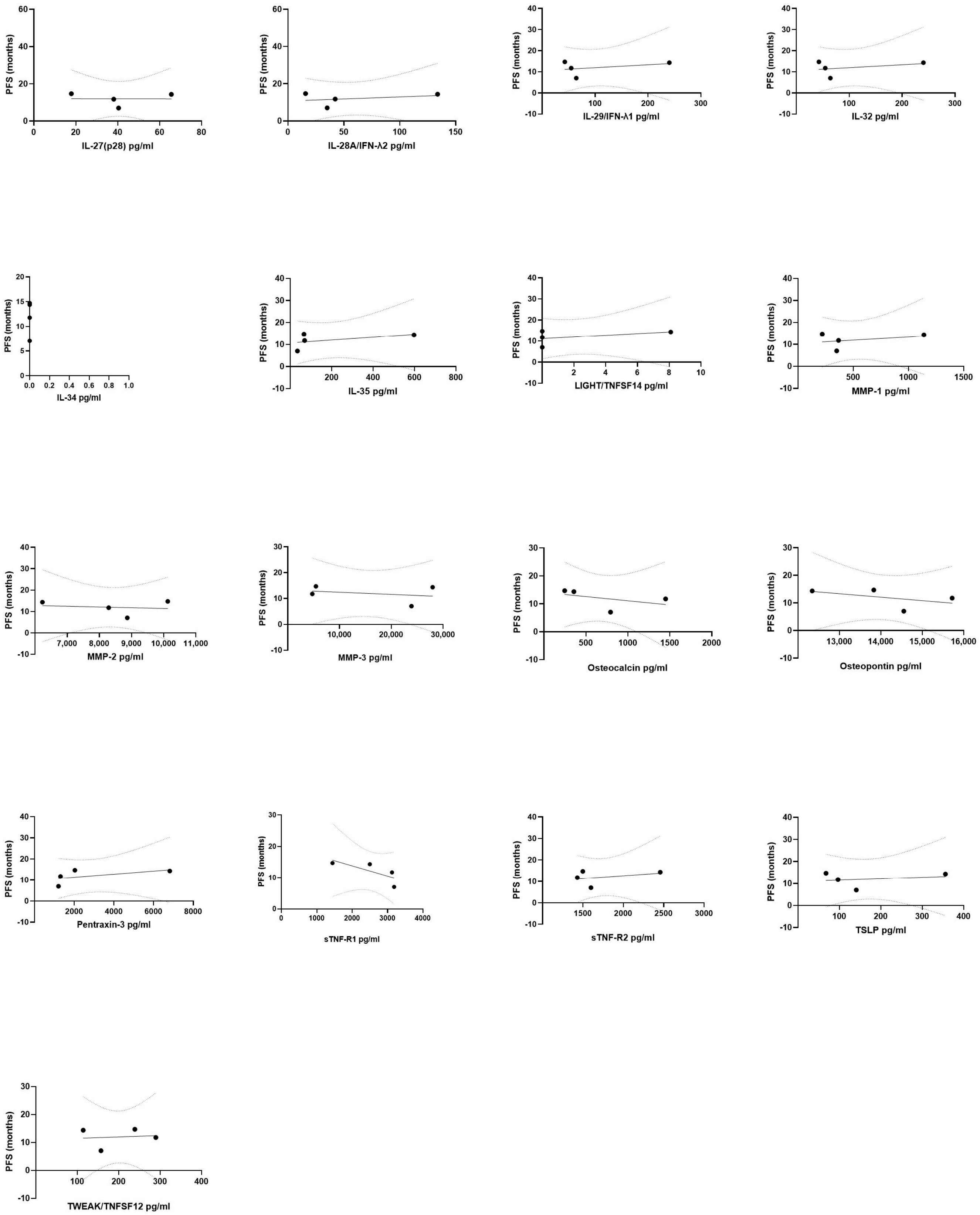
PFS (months)
IL-27(p28) pg/ml
IL-28A/IFN-λ2 pg/ml
IL-29/IFN-λ1 pg/ml
IL-32 pg/ml
IL-34 pg/ml
IL-35 pg/ml
LIGHT/TNFSF14 pg/ml
MMP-1 pg/ml
MMP-2 pg/ml
MMP-3 pg/ml
Osteocalcin pg/ml
Osteopontin pg/ml
Pentraxin-3 pg/ml
sTNF-R1 pg/ml
sTNF-R2 pg/ml
TSLP pg/ml
TWEAK/TNFSF12 pg/ml

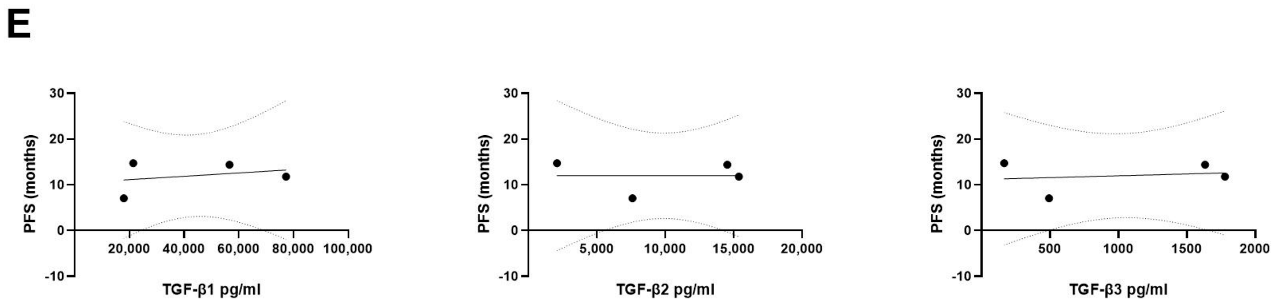

454

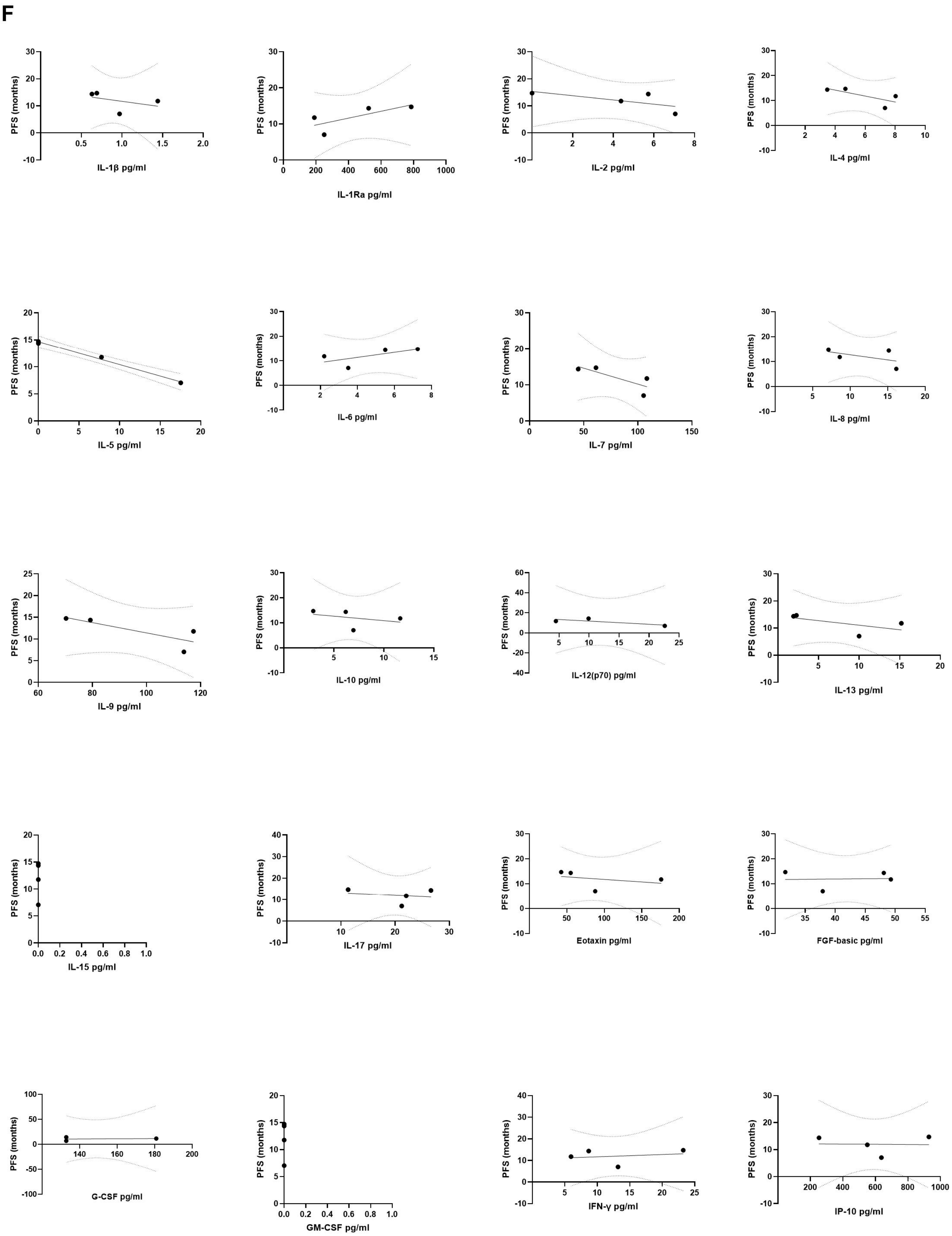
F
PFS (months)
IL-1β pg/ml
IL-1Ra pg/ml
IL-2 pg/ml
IL-4 pg/ml
IL-5 pg/ml
IL-6 pg/ml
IL-7 pg/ml
IL-8 pg/ml
IL-9 pg/ml
IL-10 pg/ml
IL-12(p70) pg/ml
IL-13 pg/ml
IL-15 pg/ml
IL-17 pg/ml
Eotaxin pg/ml
FGF-basic pg/ml
G-CSF pg/ml
GM-CSF pg/ml
IFN-γ pg/ml
IP-10 pg/ml

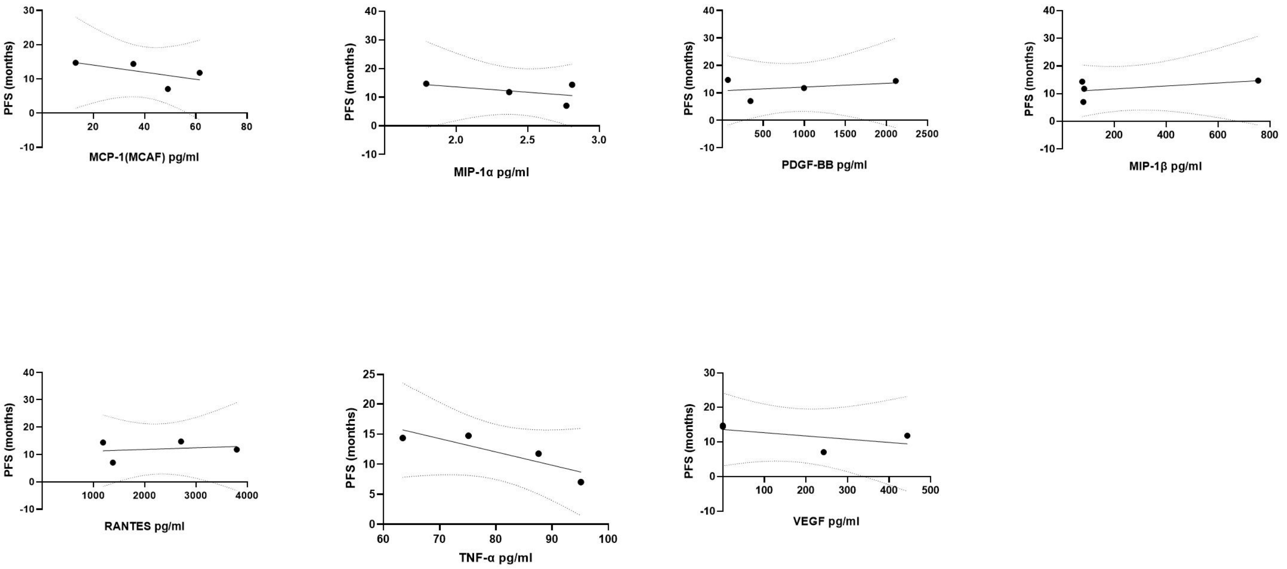
PFS (months)
MCP-1(MCAF) pg/ml
MIP-1α pg/ml
PDGF-BB pg/ml
MIP-1β pg/ml
RANTES pg/ml
TNF-α pg/ml
VEGF pg/ml

G

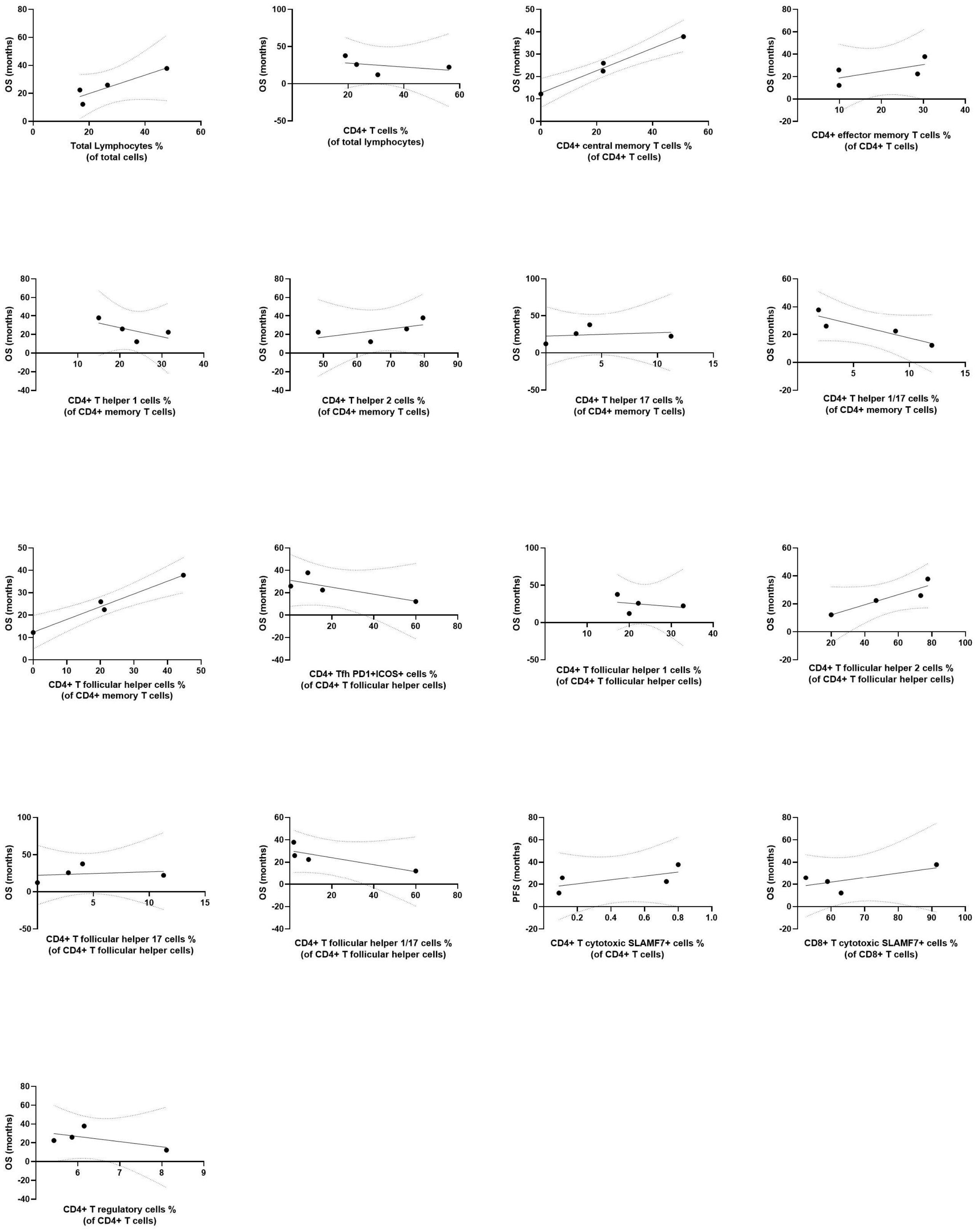
OS (months)
Total Lymphocytes %
(of total cells)
CD4+ T cells %
(of total lymphocytes)
CD4+ central memory T cells %
(of CD4+ T cells)
CD4+ effector memory T cells %
(of CD4+ T cells)
CD4+ T helper 1 cells %
(of CD4+ memory T cells)
CD4+ T helper 2 cells %
(of CD4+ memory T cells)
CD4+ T helper 17 cells %
(of CD4+ memory T cells)
CD4+ T helper 1/17 cells %
(of CD4+ memory T cells)
CD4+ T follicular helper cells %
(of CD4+ memory T cells)
CD4+ Tfh PD1+ICOS+ cells %
(of CD4+ T follicular helper cells)
CD4+ T follicular helper 1 cells %
(of CD4+ T follicular helper cells)
CD4+ T follicular helper 2 cells %
(of CD4+ T follicular helper cells)
CD4+ T follicular helper 17 cells %
(of CD4+ T follicular helper cells)
CD4+ T follicular helper 1/17 cells %
(of CD4+ T follicular helper cells)
PFS (months)
CD4+ T cytotoxic SLAMF7+ cells %
(of CD4+ T cells)
CD8+ T cytotoxic SLAMF7+ cells %
(of CD8+ T cells)
CD4+ T regulatory cells %
(of CD4+ T cells)

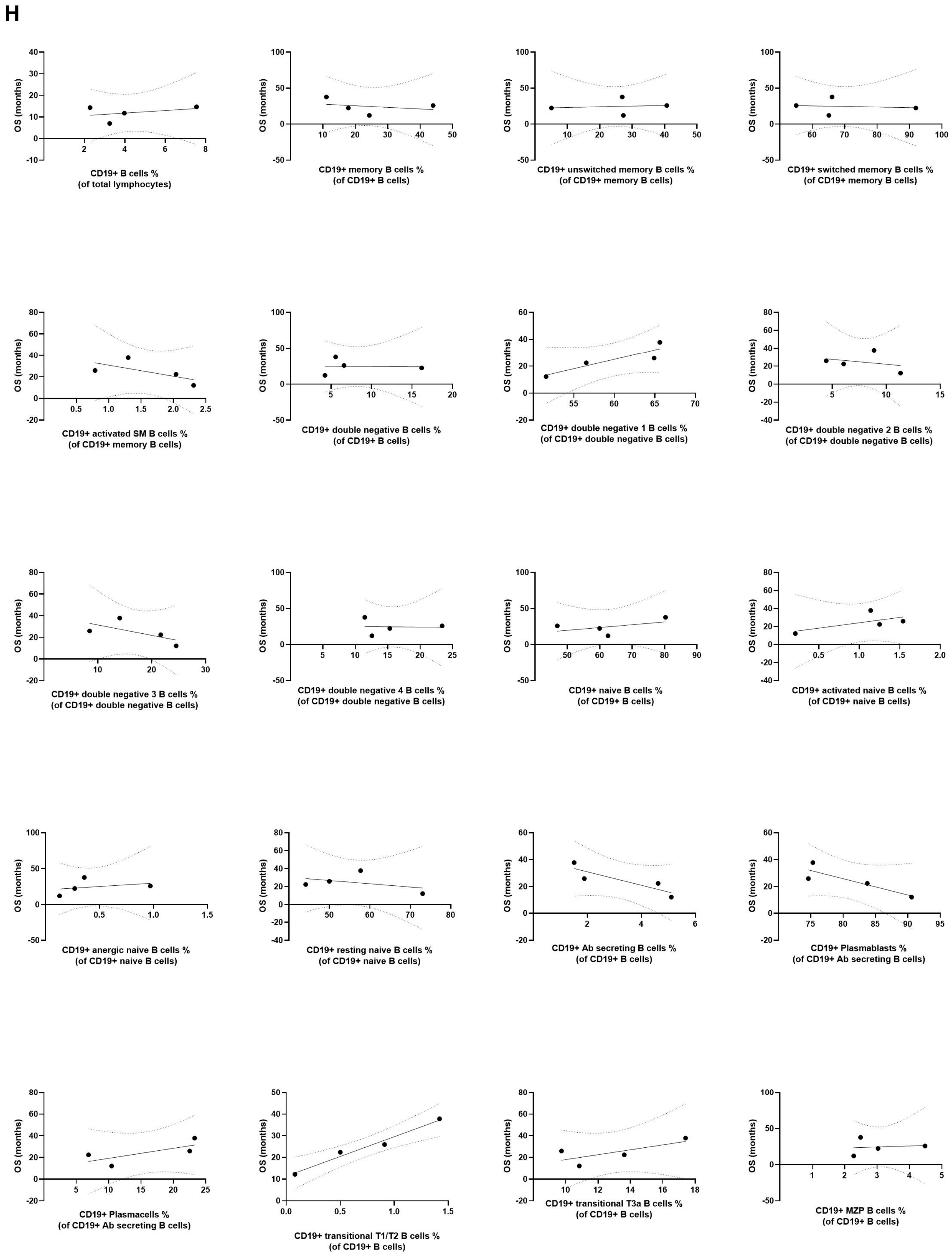
H
OS (months)
CD19+ B cells %
(of total lymphocytes)
CD19+ memory B cells %
(of CD19+ B cells)
CD19+ unswitched memory B cells %
(of CD19+ memory B cells)
CD19+ switched memory B cells %
(of CD19+ memory B cells)
CD19+ activated SM B cells %
(of CD19+ memory B cells)
CD19+ double negative B cells %
(of CD19+ B cells)
CD19+ double negative 1 B cells %
(of CD19+ double negative B cells)
CD19+ double negative 2 B cells %
(of CD19+ double negative B cells)
CD19+ double negative 3 B cells %
(of CD19+ double negative B cells)
CD19+ double negative 4 B cells %
(of CD19+ double negative B cells)
CD19+ naive B cells %
(of CD19+ B cells)
CD19+ activated naive B cells %
(of CD19+ naive B cells)
CD19+ anergic naive B cells %
(of CD19+ naive B cells)
CD19+ resting naive B cells %
(of CD19+ naive B cells)
CD19+ Ab secreting B cells %
(of CD19+ B cells)
CD19+ Plasmablasts %
(of CD19+ Ab secreting B cells)
CD19+ Plasmacells %
(of CD19+ Ab secreting B cells)
CD19+ transitional T1/T2 B cells %
(of CD19+ B cells)
CD19+ transitional T3a B cells %
(of CD19+ B cells)
CD19+ MZP B cells %
(of CD19+ B cells)

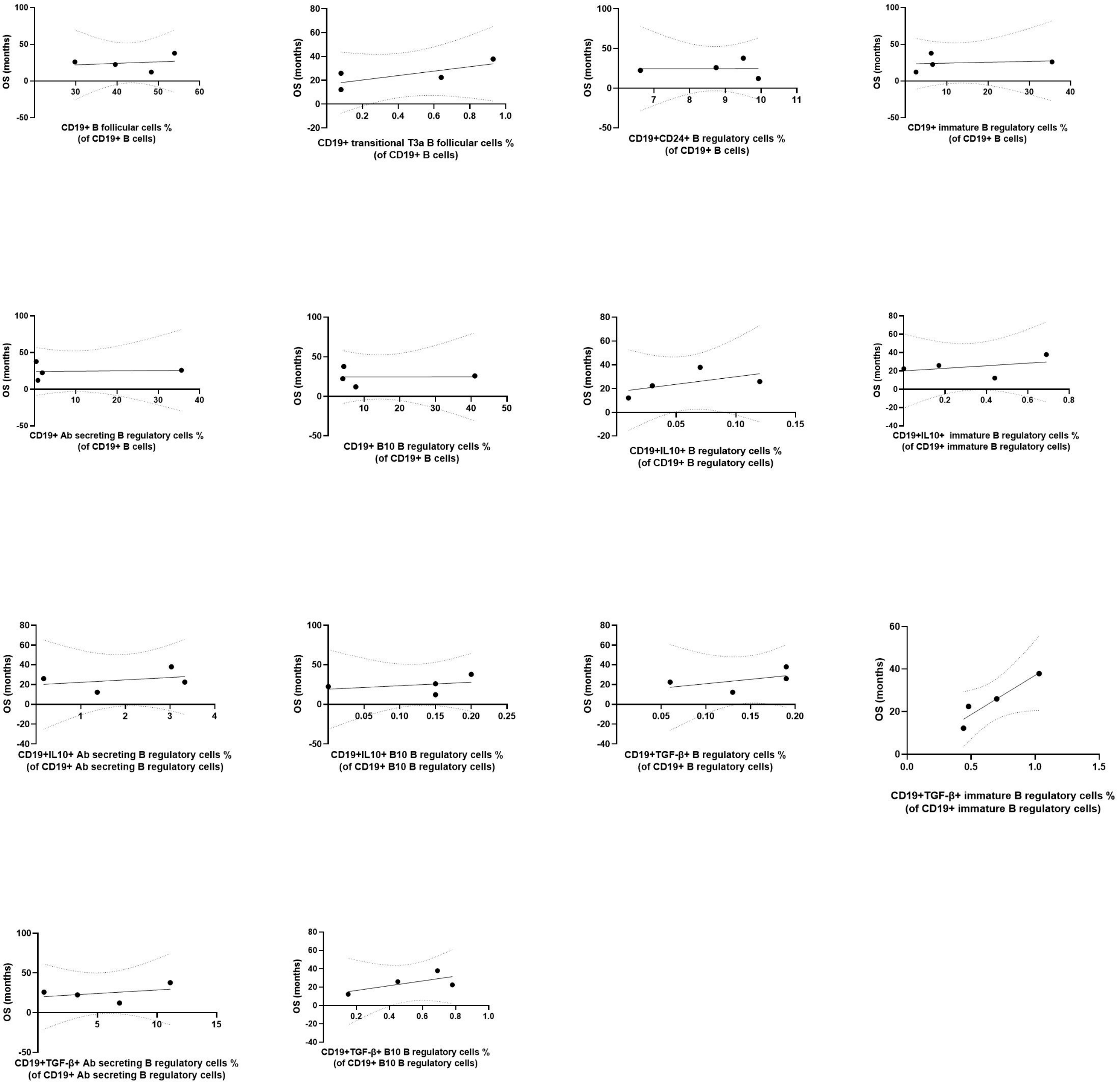
OS (months)
CD19+ B follicular cells %
(of CD19+ B cells)
CD19+ transitional T3a B follicular cells %
(of CD19+ B cells)
CD19+CD24+ B regulatory cells %
(of CD19+ B cells)
CD19+ immature B regulatory cells %
(of CD19+ B cells)
CD19+ Ab secreting B regulatory cells %
(of CD19+ B cells)
CD19+ B10 B regulatory cells %
(of CD19+ B cells)
CD19+IL10+ B regulatory cells %
(of CD19+ B regulatory cells)
CD19+IL10+ immature B regulatory cells %
(of CD19+ immature B regulatory cells)
CD19+IL10+ Ab secreting B regulatory cells %
(of CD19+ Ab secreting B regulatory cells)
CD19+IL10+ B10 B regulatory cells %
(of CD19+ B10 B regulatory cells)
CD19+TGF-β+ B regulatory cells %
(of CD19+ B regulatory cells)
CD19+TGF-β+ immature B regulatory cells %
(of CD19+ immature B regulatory cells)
CD19+TGF-β+ Ab secreting B regulatory cells %
(of CD19+ Ab secreting B regulatory cells)
CD19+TGF-β+ B10 B regulatory cells %
(of CD19+ B10 B regulatory cells)

462

I

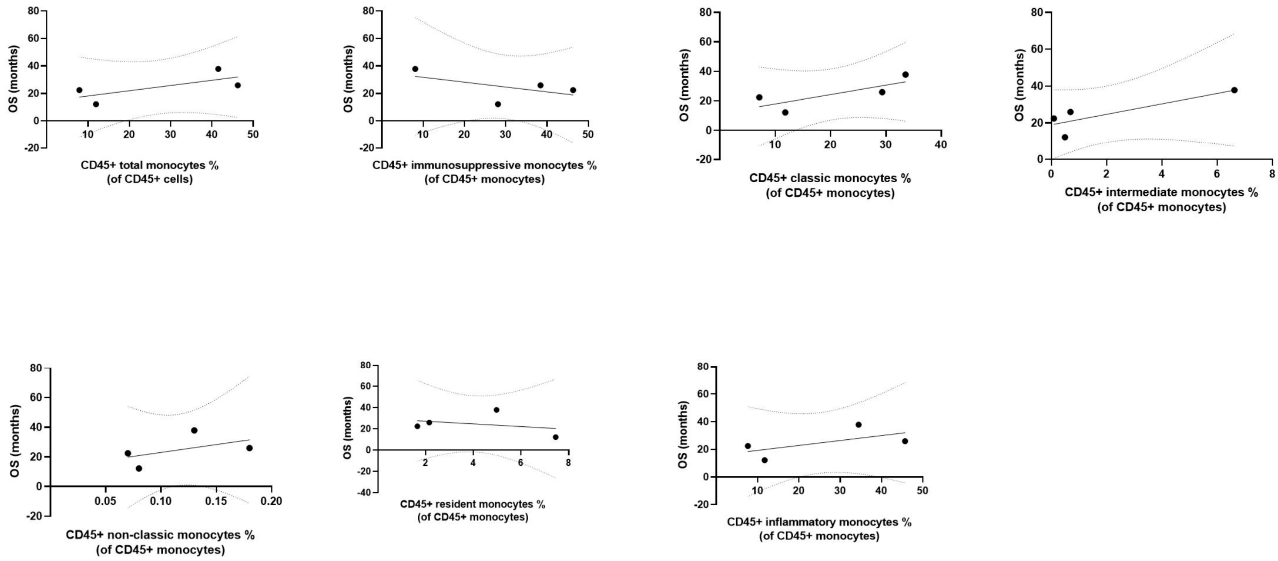
OS (months)
CD45+ total monocytes %
(of CD45+ cells)
CD45+ immunosuppressive monocytes %
(of CD45+ monocytes)
CD45+ classic monocytes %
(of CD45+ monocytes)
CD45+ intermediate monocytes %
(of CD45+ monocytes)
CD45+ non-classic monocytes %
(of CD45+ monocytes)
CD45+ resident monocytes %
(of CD45+ monocytes)
CD45+ inflammatory monocytes %
(of CD45+ monocytes)

J

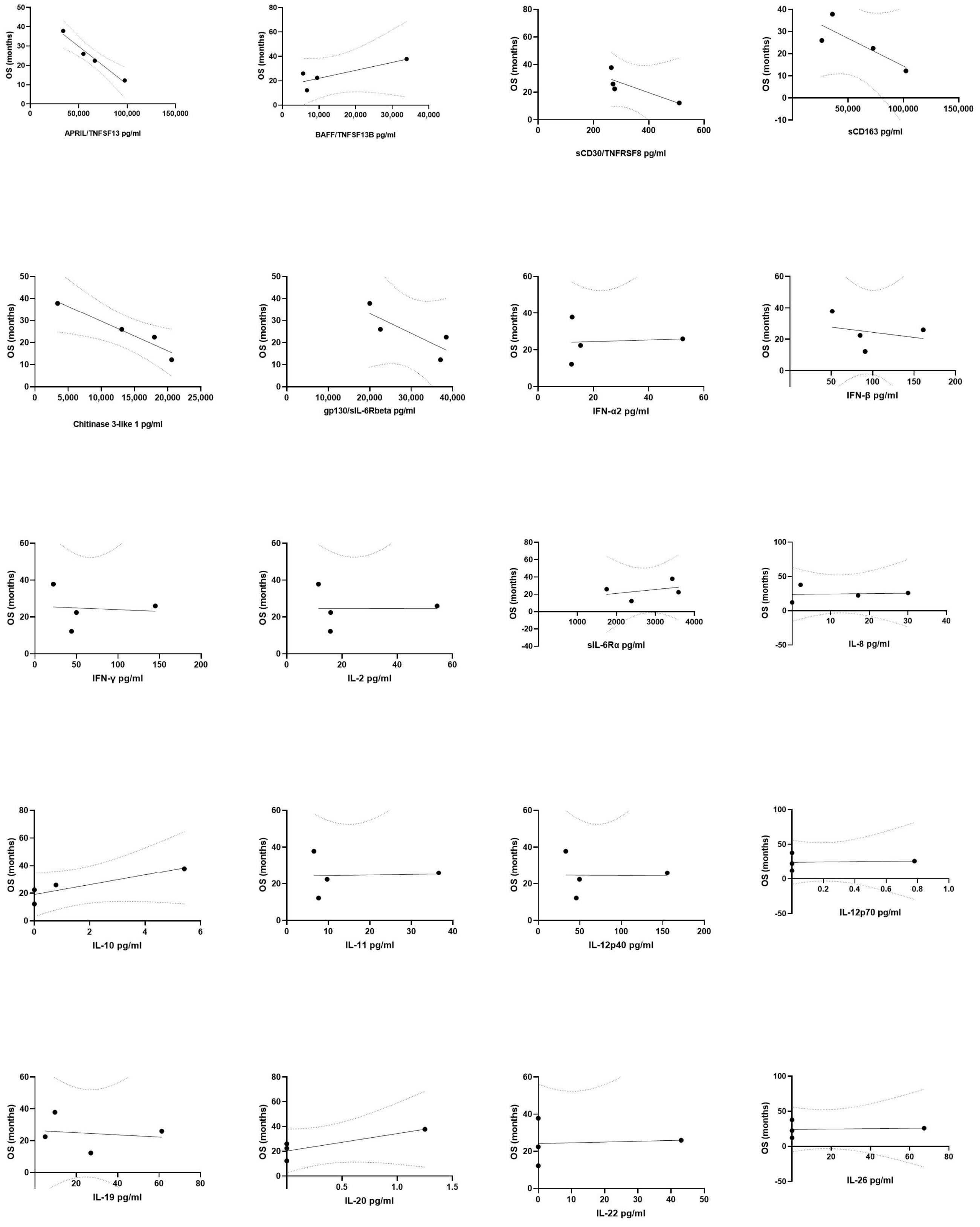
OS (months)
APRIL/TNFSF13 pg/ml
BAFF/TNFSF13B pg/ml
sCD30/TNFRSF8 pg/ml
sCD163 pg/ml
Chitinase 3-like 1 pg/ml
gp130/sIL-6Rbeta pg/ml
IFN-α2 pg/ml
IFN-β pg/ml
IFN-γ pg/ml
IL-2 pg/ml
sIL-6Rα pg/ml
IL-8 pg/ml
IL-10 pg/ml
IL-11 pg/ml
IL-12p40 pg/ml
IL-12p70 pg/ml
IL-19 pg/ml
IL-20 pg/ml
IL-22 pg/ml
IL-26 pg/ml

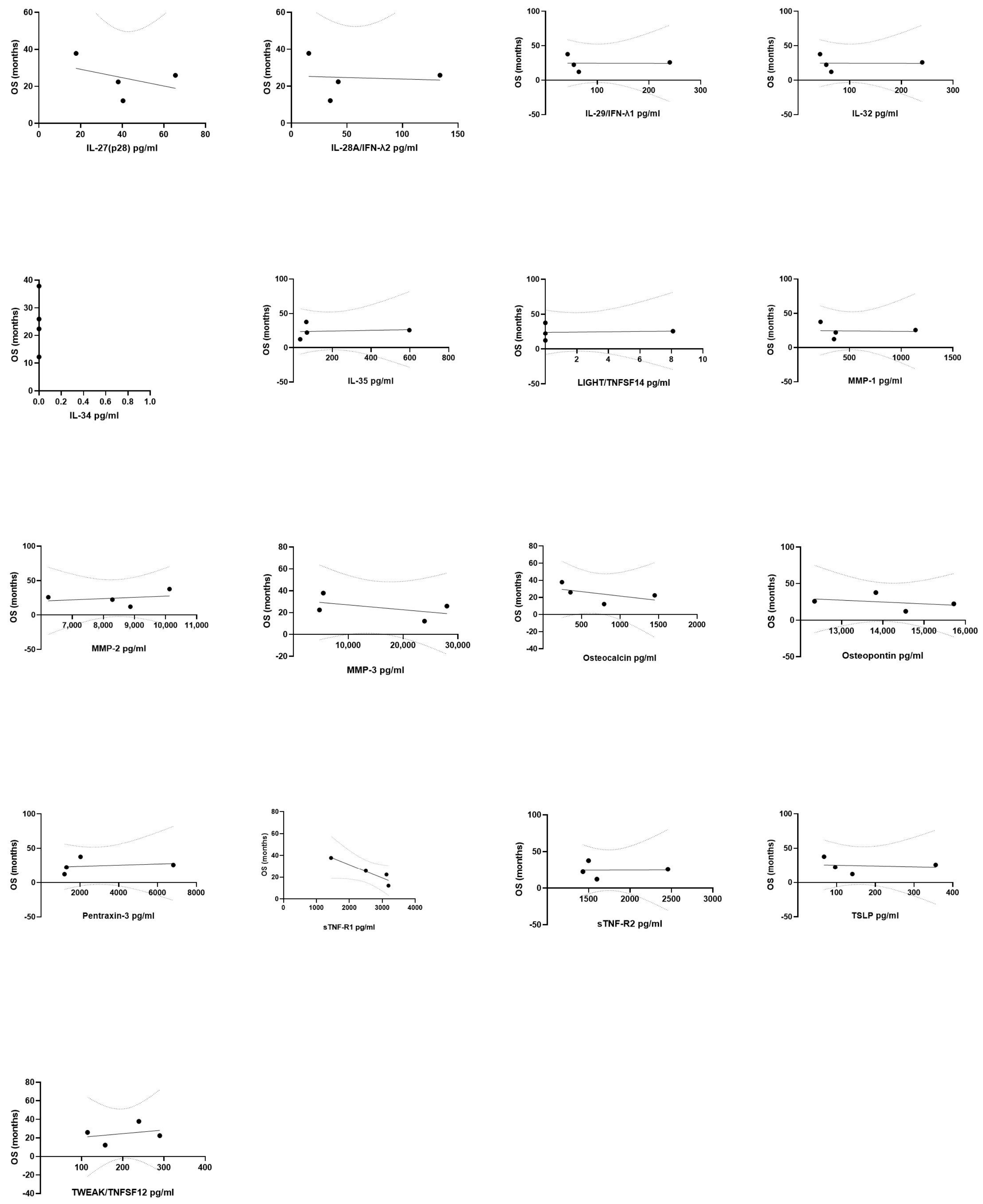
OS (months)
IL-27(p28) pg/ml
IL-28A/IFN-λ2 pg/ml
IL-29/IFN-λ1 pg/ml
IL-32 pg/ml
IL-34 pg/ml
IL-35 pg/ml
LIGHT/TNFSF14 pg/ml
MMP-1 pg/ml
MMP-2 pg/ml
MMP-3 pg/ml
Osteocalcin pg/ml
Osteopontin pg/ml
Pentraxin-3 pg/ml
sTNF-R1 pg/ml
sTNF-R2 pg/ml
TSLP pg/ml
TWEAK/TNFSF12 pg/ml

K

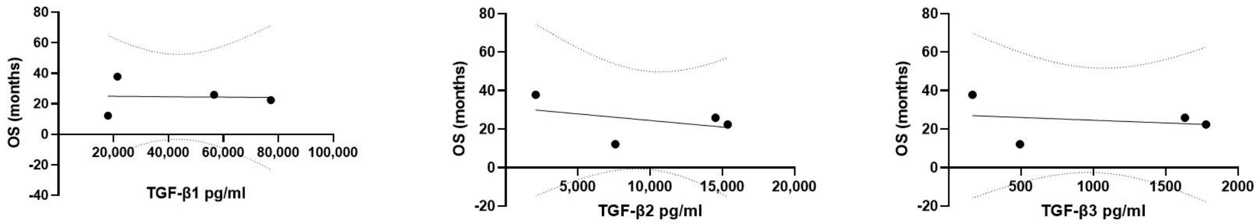

L

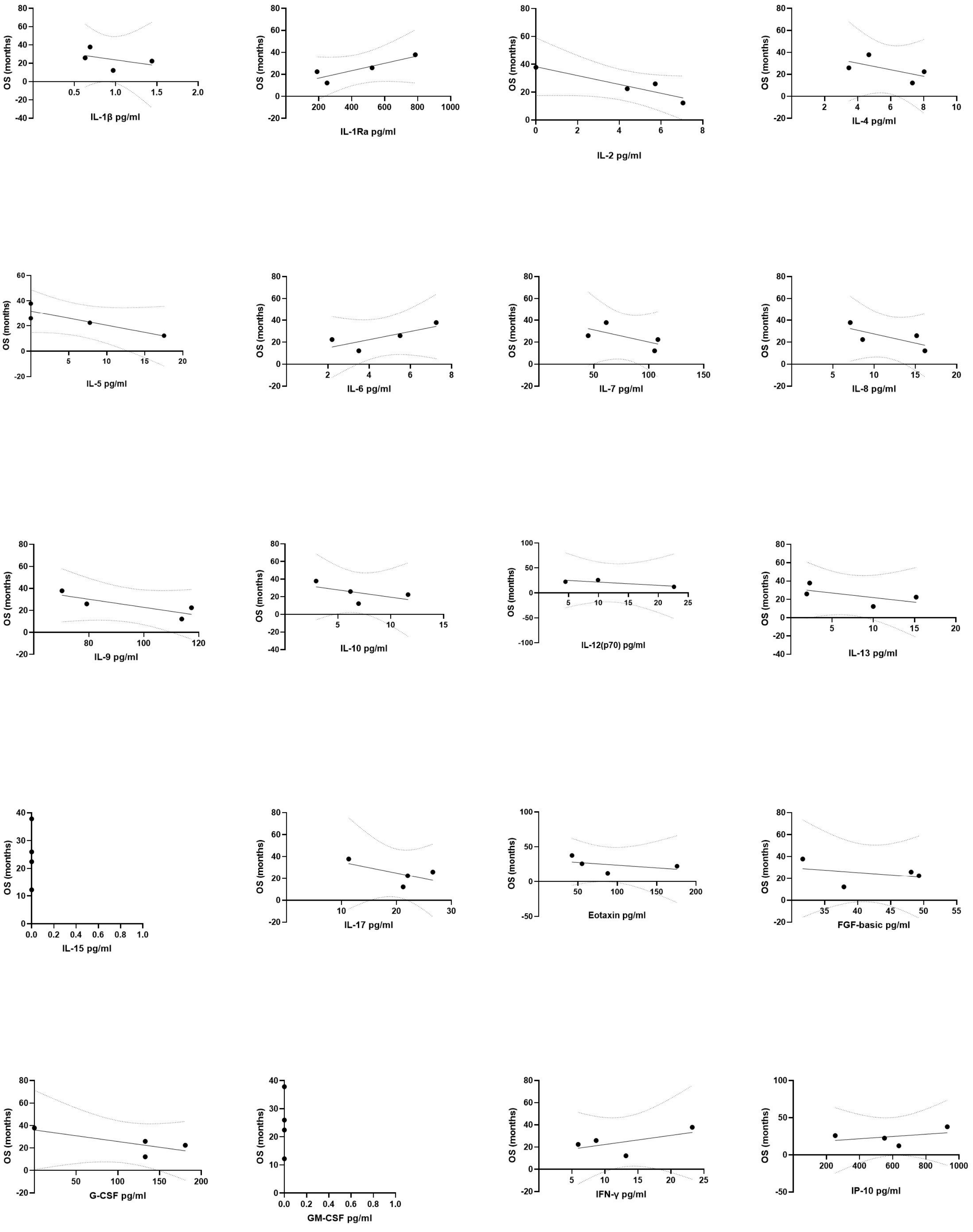
OS (months)
IL-1β pg/ml
IL-1Ra pg/ml
IL-2 pg/ml
IL-4 pg/ml
IL-5 pg/ml
IL-6 pg/ml
IL-7 pg/ml
IL-8 pg/ml
IL-9 pg/ml
IL-10 pg/ml
IL-12(p70) pg/ml
IL-13 pg/ml
IL-15 pg/ml
IL-17 pg/ml
Eotaxin pg/ml
FGF-basic pg/ml
G-CSF pg/ml
GM-CSF pg/ml
IFN-γ pg/ml
IP-10 pg/ml

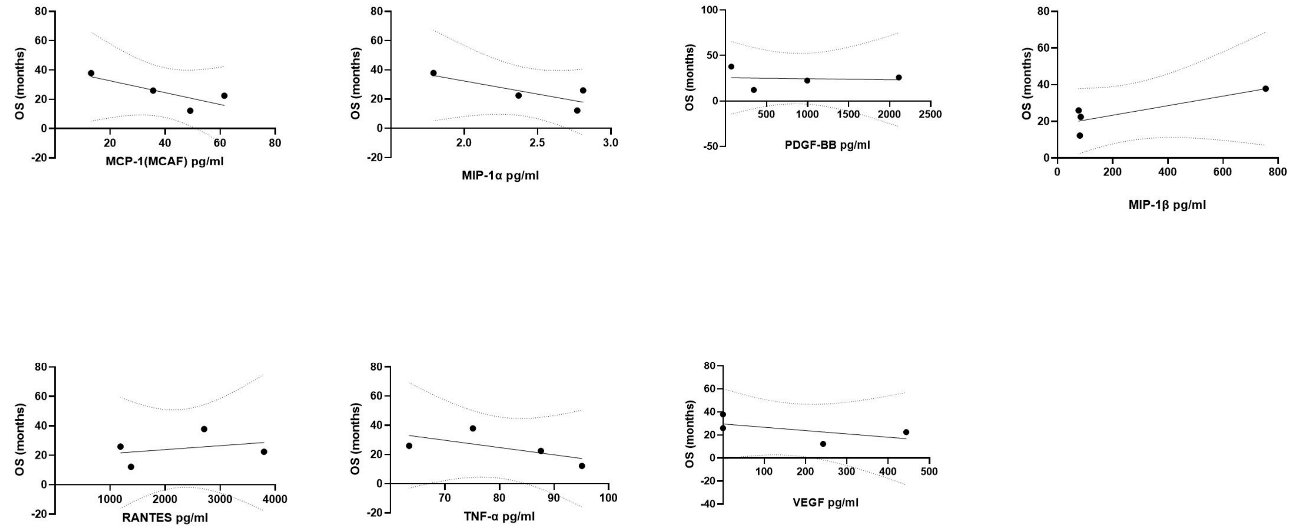

Footnote: Te: T effector; Th1: T helper 1; Th2: T helper 2; Th17: T helper 17; Th1/17: T helper 1/17; Tfh: T follicular helper; Tfh1: T follicular helper 1; Tfh2: T follicular helper 2; Tfh17: T follicular helper 17; Tfh1/17: T follicular helper 1/17; Treg: T regulatory; UM: unswitched memory; SM: switched memory; ASM: activated switched memory; DN: double negative; DN1: double negative 1; DN2: double negative 2; DN3: double negative 3; DN4: double negative 4; AcN: activated naïve; AnN: anergic naïve; RN: resting naïve; Ab: antibody secreting; T1/2: transitional 1/2; T3a: transitional 3a; MZP: marginal zone peripheral; Breg: B regulatory; AbS: antibody secreting; Imm: immature; Mon: monocytes.

Figure S11. Differences of circulating immune cells (A: T-cell compartment; B: B-cell compartment; C: monocyte compartment) and concentrations of inflammatory and immune-related cytokines and chemokines (D: Pro Human Inflammation Panel I Assay; E: Pro TGF-β Immunoassay; F: Pro Human Cytokine Immunoassay) between patients with pancreatic adenocarcinoma (PDAC) treated with HybridTherm Probe ablation plus chemotherapy (HTP-CT arm) and those treated with chemotherapy only (CT arm) at baseline. Inter-group variables were compared using the Welch two sample t-test: * = $p < 0.05$.

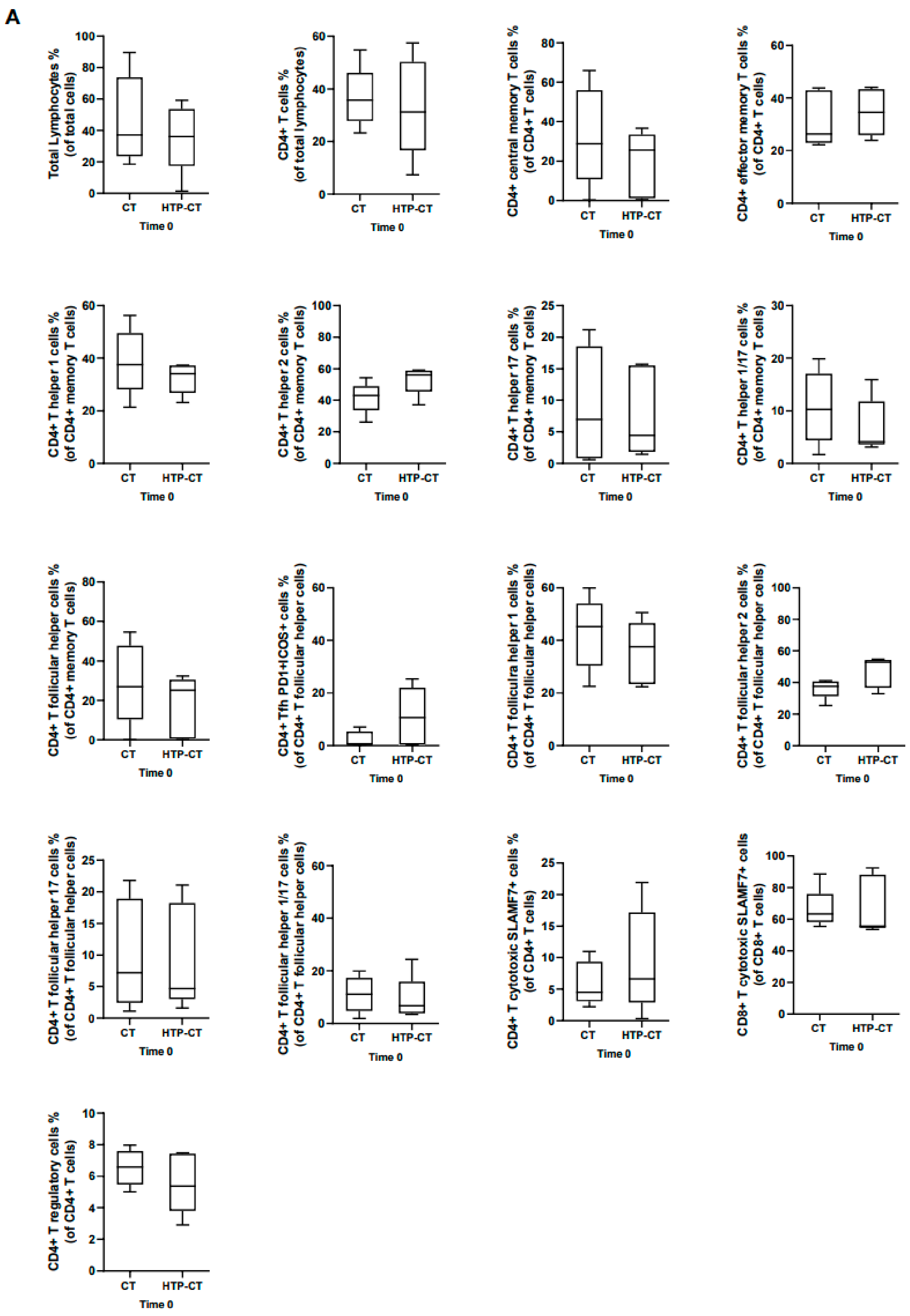
A
Total Lymphocytes %
(of total cells)
CD4+ T cells %
(of total lymphocytes)
CD4+ central memory T cells %
(of CD4+ T cells)
CD4+ effector memory T cells %
(of CD4+ T cells)
CD4+ T helper 1 cells %
(of CD4+ memory T cells)
CD4+ T helper 2 cells %
(of CD4+ memory T cells)
CD4+ T helper 17 cells %
(of CD4+ memory T cells)
CD4+ T helper 1/17 cells %
(of CD4+ memory T cells)
CD4+ T follicular helper cells %
(of CD4+ memory T cells)
CD4+ Tfh PD1+ICOS+ cells %
(of CD4+ T follicular helper cells)
CD4+ T follicura helper 1 cells %
(of CD4+ T follicular helper cells)
CD4+ T follicular helper 2 cells %
(of CD4+ T follicular helper cells)
CD4+ T follicular helper 17 cells %
(of CD4+ T follicular helper cells)
CD4+ T follicular helper 1/17 cells %
(of CD4+ T follicular helper cells)
CD4+ T cytotoxic SLAMF7+ cells %
(of CD4+ T cells)
CD8+ T cytotoxic SLAMF7+ cells
(of CD8+ T cells)
CD4+ T regulatory cells %
(of CD4+ T cells)
CT
HTP-CT
Time 0

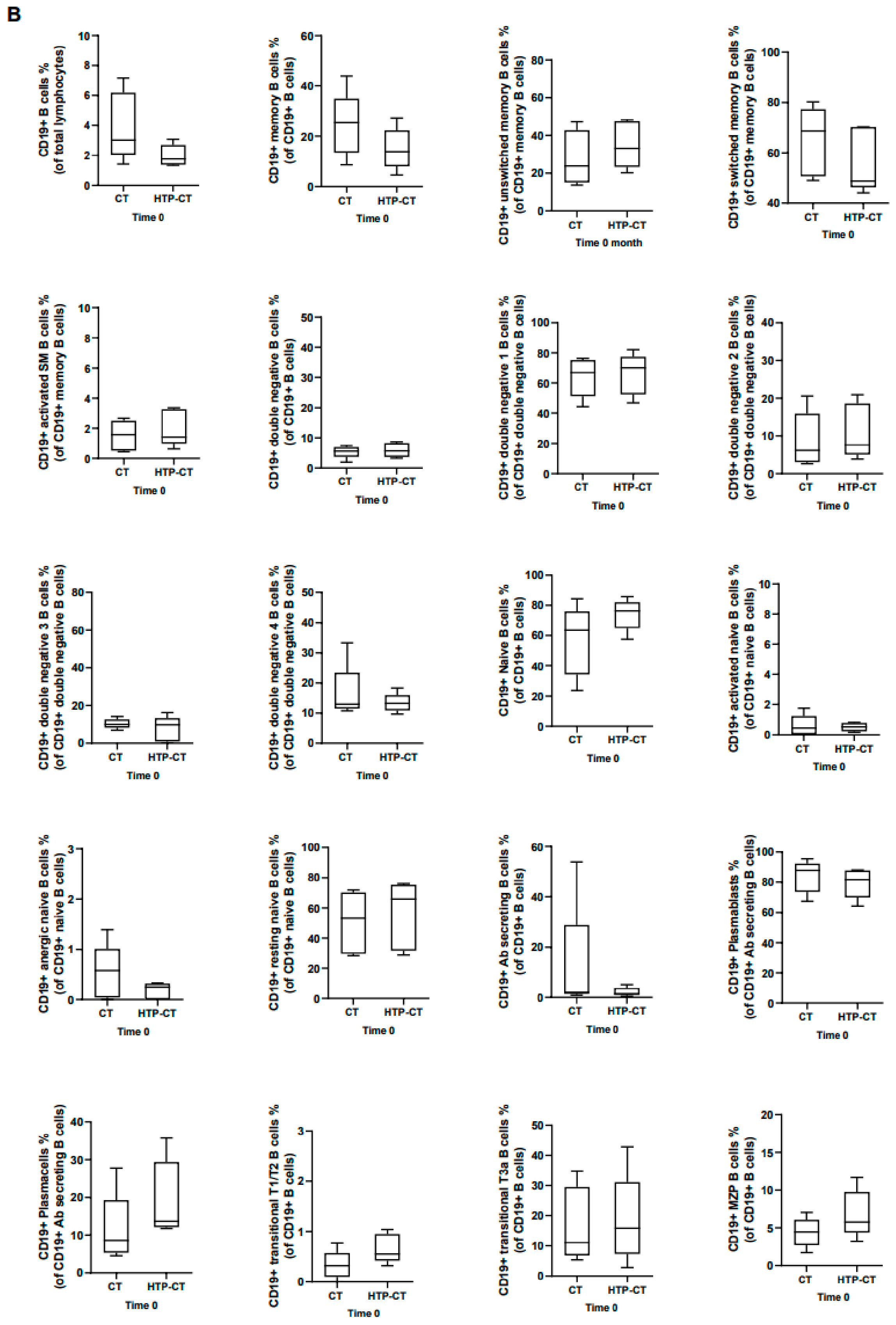
B
CD19+ B cells % (of total lymphocytes)
CD19+ memory B cells % (of CD19+ B cells)
CD19+ unswitched memory B cells % (of CD19+ memory B cells)
CD19+ switched memory B cells % (of CD19+ memory B cells)
CD19+ activated SM B cells % (of CD19+ memory B cells)
CD19+ double negative B cells % (of CD19+ B cells)
CD19+ double negative 1 B cells % (of CD19+ double negative B cells)
CD19+ double negative 2 B cells % (of CD19+ double negative B cells)
CD19+ double negative 3 B cells % (of CD19+ double negative B cells)
CD19+ double negative 4 B cells % (of CD19+ double negative B cells)
CD19+ Naive B cells % (of CD19+ B cells)
CD19+ activated naive B cells % (of CD19+ naive B cells)
CD19+ anergic naive B cells % (of CD19+ naive B cells)
CD19+ resting naive B cells % (of CD19+ naive B cells)
CD19+ Ab secreting B cells % (of CD19+ B cells)
CD19+ Plasmablasts % (of CD19+ Ab secreting B cells)
CD19+ Plasmacells % (of CD19+ Ab secreting B cells)
CD19+ transitional T1/T2 B cells % (of CD19+ B cells)
CD19+ transitional T3a B cells % (of CD19+ B cells)
CD19+ MZP B cells % (of CD19+ B cells)
CT
HTP-CT
Time 0
Time 0 month

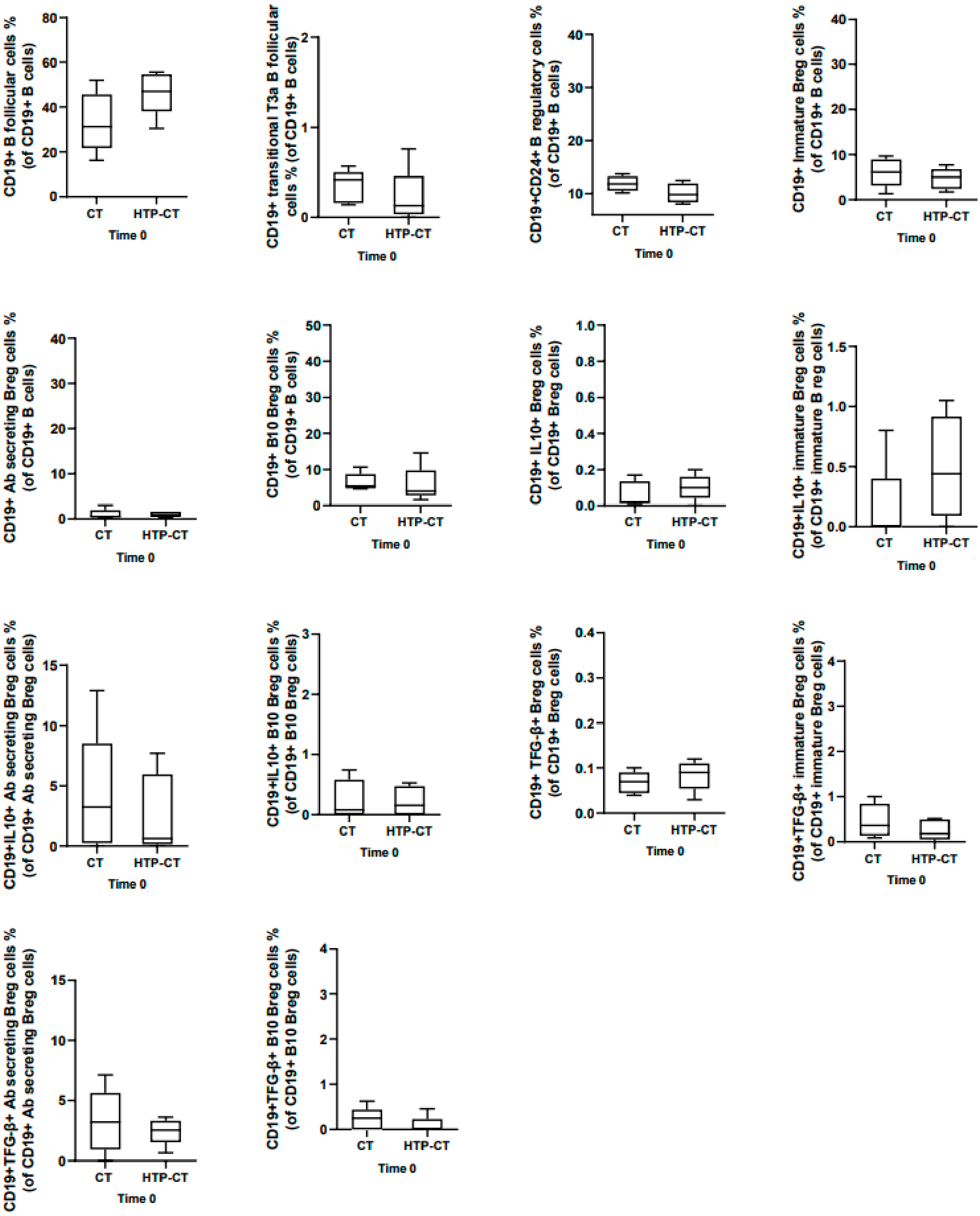
CD19+ B follicular cells % (of CD19+ B cells)
0 20 40 60 80
CT HTP-CT
Time 0
CD19+ transitional T3a B follicular cells % (of CD19+ B cells)
0 1 2
CT HTP-CT
Time 0
CD19+CD24+ B regulatory cells % (of CD19+ B cells)
10 20 30 40
CT HTP-CT
Time 0
CD19+ Immature Breg cells % (of CD19+ B cells)
0 10 20 30 40
CT HTP-CT
Time 0
CD19+ Ab secreting Breg cells % (of CD19+ B cells)
0 10 20 30 40
CT HTP-CT
Time 0
CD19+ B10 Breg cells % (of CD19+ B cells)
0 10 20 30 40 50
CT HTP-CT
Time 0
CD19+ IL10+ Breg cells % (of CD19+ Breg cells)
0.0 0.2 0.4 0.6 0.8 1.0
CT HTP-CT
Time 0
CD19+IL10+ immature Breg cells % (of CD19+ immature B reg cells)
0.0 0.5 1.0 1.5
CT HTP-CT
Time 0
CD19+IL10+ Ab secreting Breg cells % (of CD19+ Ab secreting Breg cells)
0 5 10 15
CT HTP-CT
Time 0
CD19+IL10+ B10 Breg cells % (of CD19+ B10 Breg cells)
0 1 2 3
CT HTP-CT
Time 0
CD19+ TFG-β+ Breg cells % (of CD19+ Breg cells)
0.0 0.1 0.2 0.3 0.4
CT HTP-CT
Time 0
CD19+TFG-β+ immature Breg cells % (of CD19+ immature Breg cells)
0 1 2 3 4
CT HTP-CT
Time 0
CD19+TFG-β+ Ab secreting Breg cells % (of CD19+ Ab secreting Breg cells)
0 5 10 15
CT HTP-CT
Time 0
CD19+TFG-β+ B10 Breg cells % (of CD19+ B10 Breg cells)
0 1 2 3 4
CT HTP-CT
Time 0

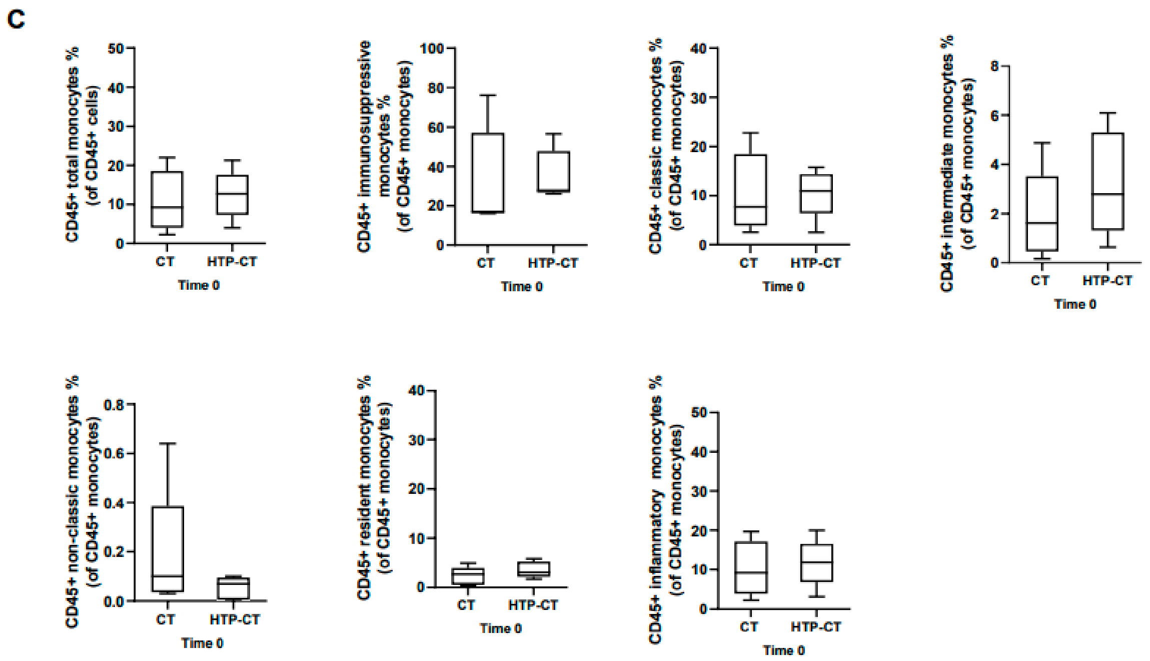
C
CD45+ total monocytes % (of CD45+ cells)
CD45+ immunosuppressive monocytes % (of CD45+ monocytes)
CD45+ classic monocytes % (of CD45+ monocytes)
CD45+ intermediate monocytes % (of CD45+ monocytes)
CD45+ non-classic monocytes % (of CD45+ monocytes)
CD45+ resident monocytes % (of CD45+ monocytes)
CD45+ inflammatory monocytes % (of CD45+ monocytes)
CT
HTP-CT
Time 0

513

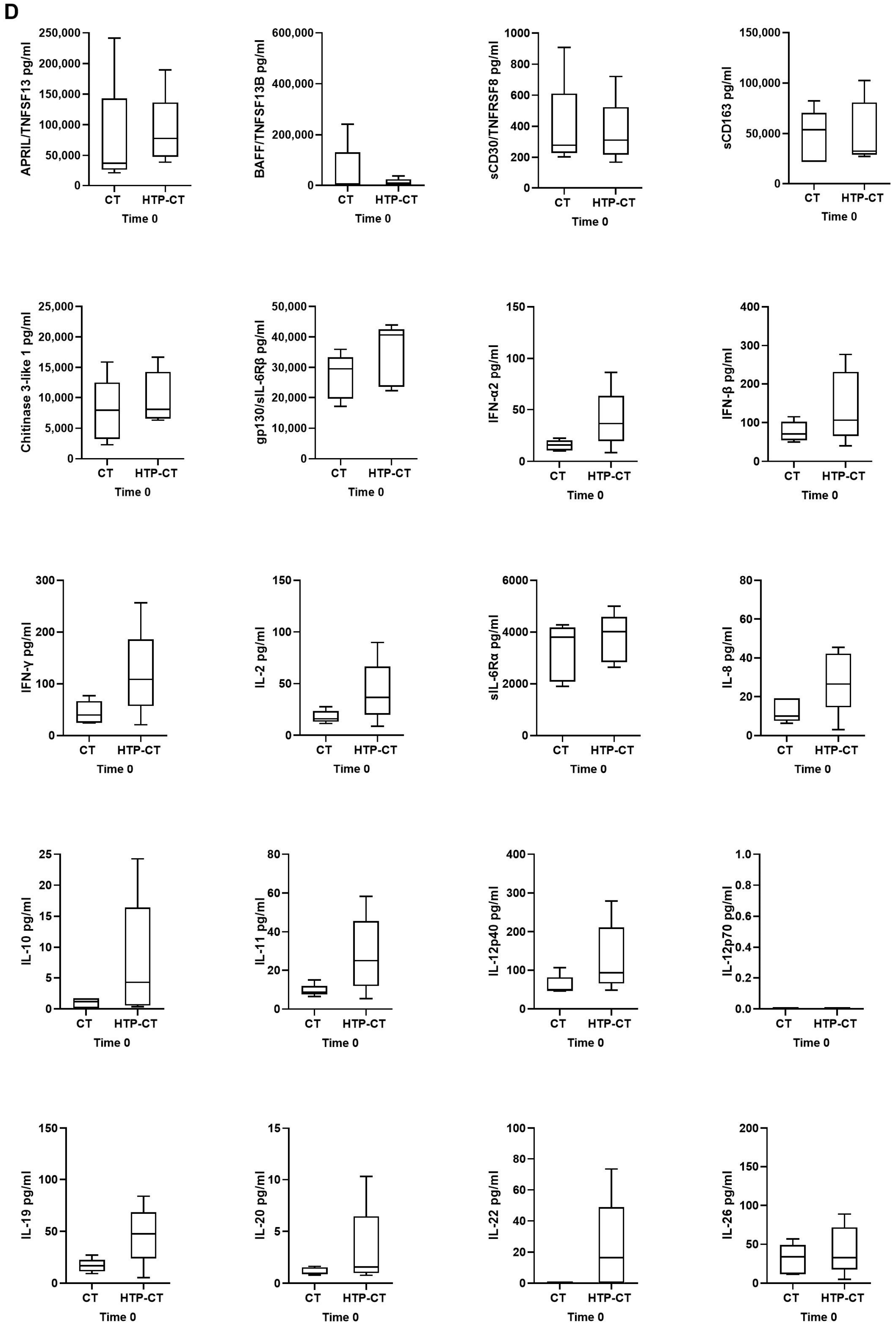
D
APRIL/TNFSF13 pg/ml
BAFF/TNFSF13B pg/ml
sCD30/TNFRSF8 pg/ml
sCD163 pg/ml
Chitinase 3-like 1 pg/ml
gp130/sIL-6Rβ pg/ml
IFN-α2 pg/ml
IFN-β pg/ml
IFN-γ pg/ml
IL-2 pg/ml
sIL-6Rα pg/ml
IL-8 pg/ml
IL-10 pg/ml
IL-11 pg/ml
IL-12p40 pg/ml
IL-12p70 pg/ml
IL-19 pg/ml
IL-20 pg/ml
IL-22 pg/ml
IL-26 pg/ml
CT
HTP-CT
Time 0

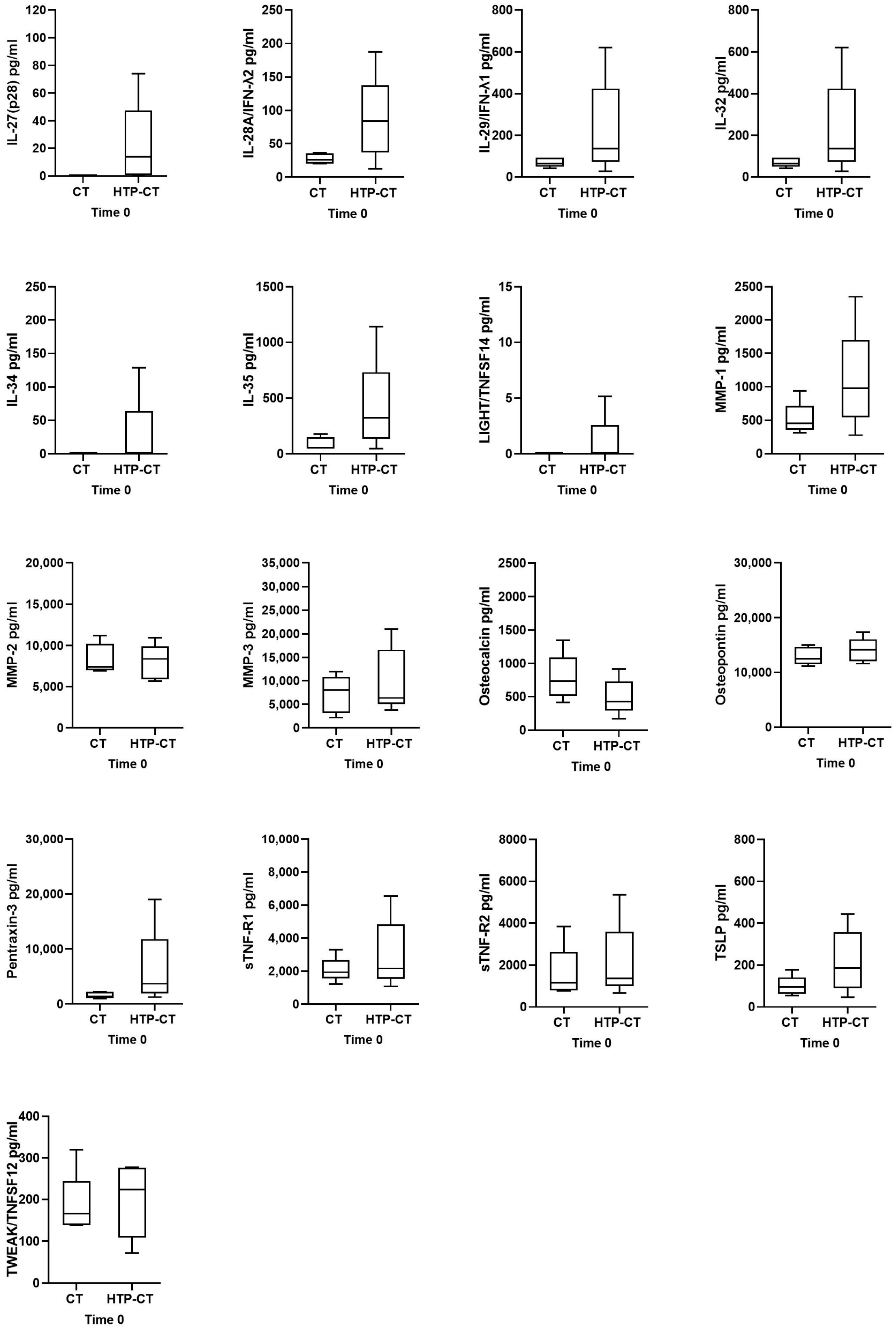
IL-27(p28) pg/ml
CT
HTP-CT
Time 0
IL-28A/IFN-λ2 pg/ml
CT
HTP-CT
Time 0
IL-29/IFN-λ1 pg/ml
CT
HTP-CT
Time 0
IL-32 pg/ml
CT
HTP-CT
Time 0
IL-34 pg/ml
CT
HTP-CT
Time 0
IL-35 pg/ml
CT
HTP-CT
Time 0
LIGHT/TNFSF14 pg/ml
CT
HTP-CT
Time 0
MMP-1 pg/ml
CT
HTP-CT
Time 0
MMP-2 pg/ml
CT
HTP-CT
Time 0
MMP-3 pg/ml
CT
HTP-CT
Time 0
Osteocalcin pg/ml
CT
HTP-CT
Time 0
Osteopontin pg/ml
CT
HTP-CT
Time 0
Pentraxin-3 pg/ml
CT
HTP-CT
Time 0
sTNF-R1 pg/ml
CT
HTP-CT
Time 0
sTNF-R2 pg/ml
CT
HTP-CT
Time 0
TSLP pg/ml
CT
HTP-CT
Time 0
TWEAK/TNFSF12 pg/ml
CT
HTP-CT
Time 0

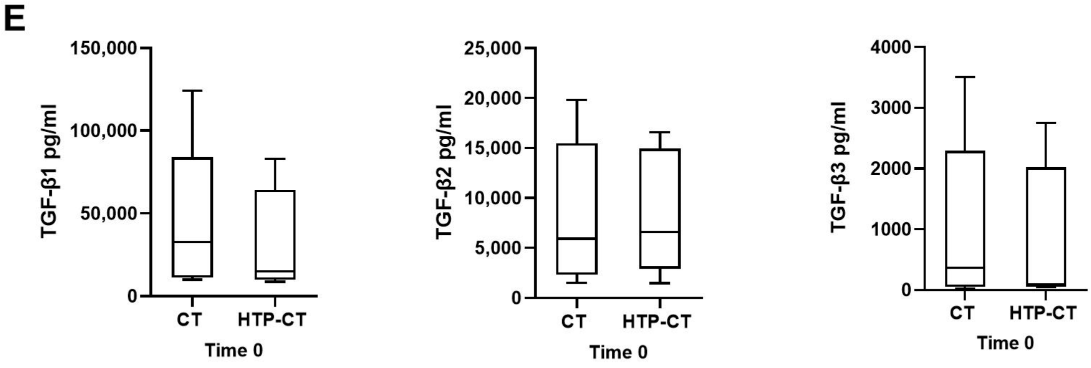
E
150,000
100,000
50,000
0
TGF-β1 pg/ml
CT
HTP-CT
Time 0
25,000
20,000
15,000
10,000
5,000
0
TGF-β2 pg/ml
CT
HTP-CT
Time 0
4000
3000
2000
1000
0
TGF-β3 pg/ml
CT
HTP-CT
Time 0

518

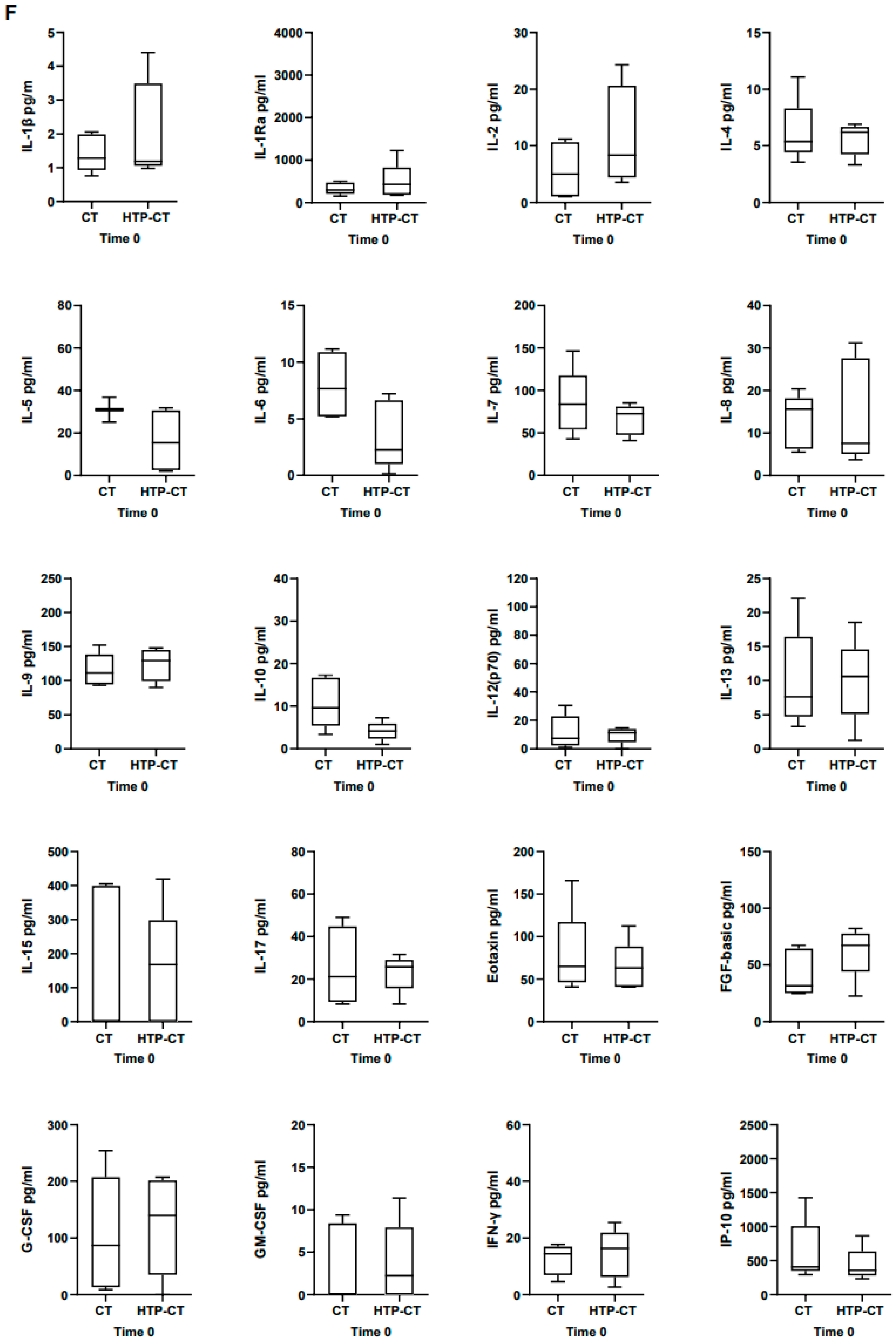
F
IL-1β pg/m
IL-1Ra pg/ml
IL-2 pg/ml
IL-4 pg/ml
IL-5 pg/ml
IL-6 pg/ml
IL-7 pg/ml
IL-8 pg/ml
IL-9 pg/ml
IL-10 pg/ml
IL-12(p70) pg/ml
IL-13 pg/ml
IL-15 pg/ml
IL-17 pg/ml
Eotaxin pg/ml
FGF-basic pg/ml
G-CSF pg/ml
GM-CSF pg/ml
IFN-γ pg/ml
IP-10 pg/ml
CT
HTP-CT
Time 0

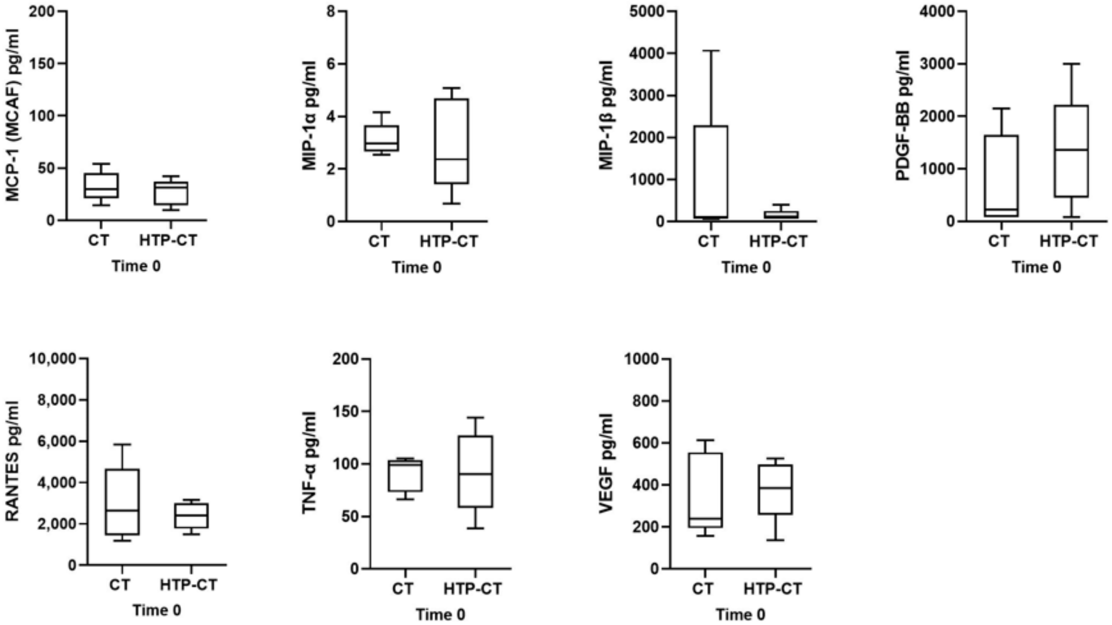

Footnote: Tfh: T follicular helper; SM: switched memory; Ab: antibody secreting; T1/2: type 1/2; T3a: type 3a; MZP: marginal zone peripheral; Breg: B regulatory.

Figure S12. Changes of circulating immune cells (A: T-cell compartment; B: B-cell compartment; C: monocyte compartment) and concentrations of inflammatory and immune-related cytokines and chemokines (D: Pro Human Inflammation Panel I Assay; E: Pro TGF-β Immunoassay; F: Pro Human Cytokine Immunoassay) between baseline (T0) and 4 months (T4M) after therapy onset in patients with pancreatic adenocarcinoma (PDAC) treated with chemotherapy only (CT arm). Square dot represents the patient in the CT arm who did not reach 4-month follow-up. Intra-group variables were compared using the paired t-test: * = $p < 0.05$.

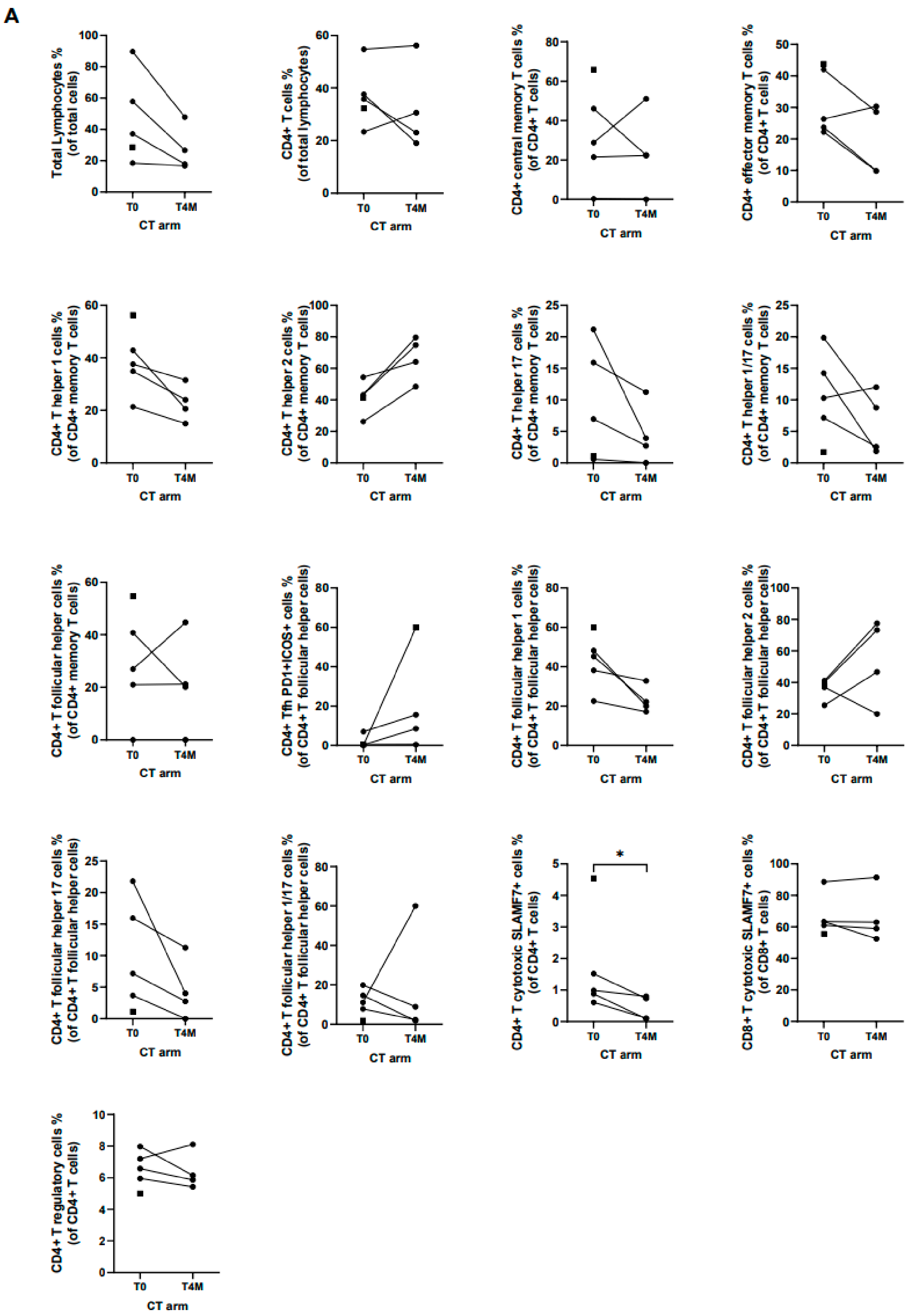

A
Total Lymphocytes %
(of total cells)
CD4+ T cells %
(of total lymphocytes)
CD4+ central memory T cells %
(of CD4+ T cells)
CD4+ effector memory T cells %
(of CD4+ T cells)
CD4+ T helper 1 cells %
(of CD4+ memory T cells)
CD4+ T helper 2 cells %
(of CD4+ memory T cells)
CD4+ T helper 17 cells %
(of CD4+ memory T cells)
CD4+ T helper 1/17 cells %
(of CD4+ memory T cells)
CD4+ T follicular helper cells %
(of CD4+ memory T cells)
CD4+ Tfh PD1+ICOS+ cells %
(of CD4+ T follicular helper cells)
CD4+ T follicular helper 1 cells %
(of CD4+ T follicular helper cells)
CD4+ T follicular helper 2 cells %
(of CD4+ T follicular helper cells)
CD4+ T follicular helper 17 cells %
(of CD4+ T follicular helper cells)
CD4+ T follicular helper 1/17 cells %
(of CD4+ T follicular helper cells)
CD4+ T cytotoxic SLAMF7+ cells %
(of CD4+ T cells)
CD8+ T cytotoxic SLAMF7+ cells %
(of CD8+ T cells)
CD4+ T regulatory cells %
(of CD4+ T cells)
T0
T4M
CT arm
*

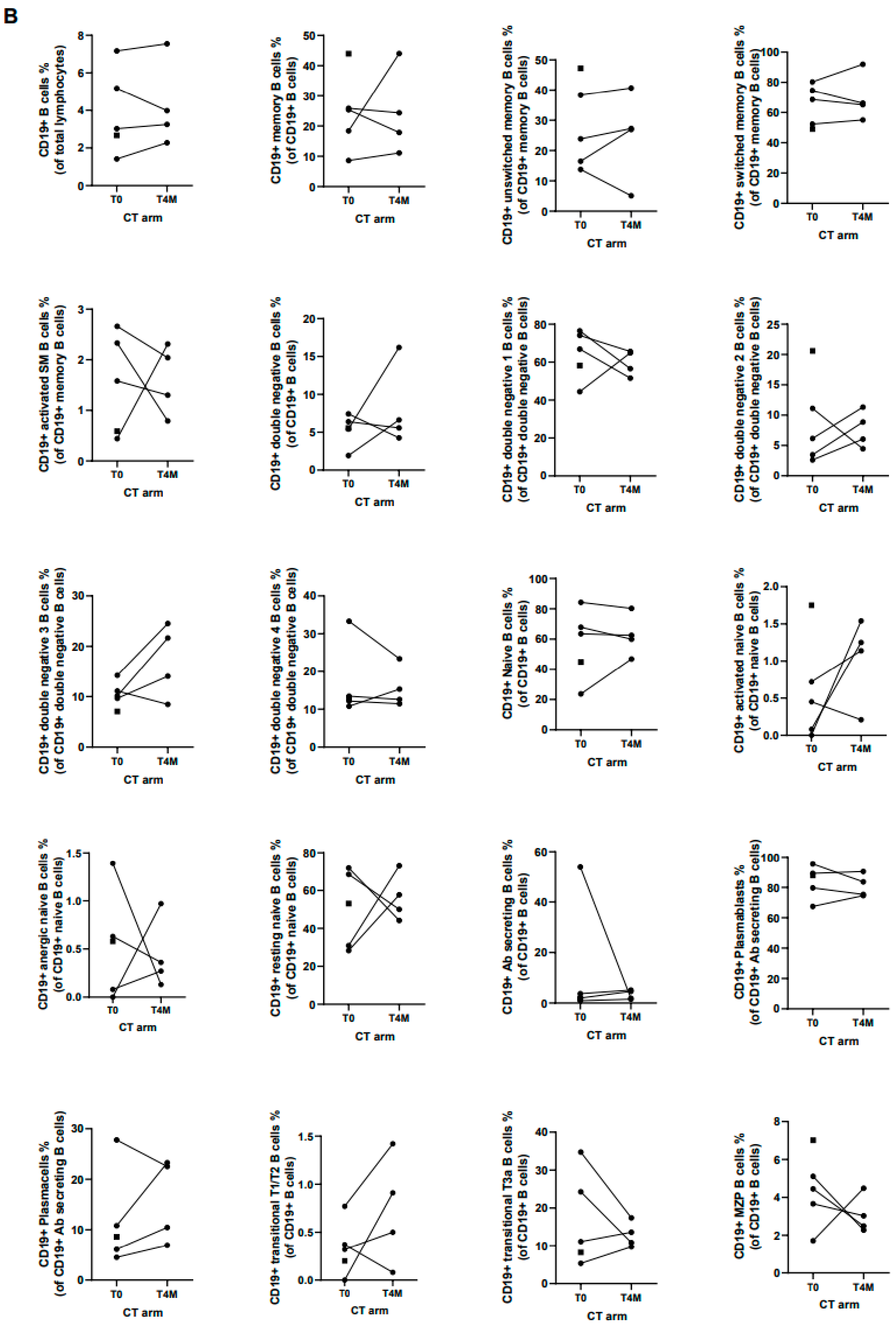
B
CD19+ B cells % (of total lymphocytes)
CD19+ memory B cells % (of CD19+ B cells)
CD19+ unswitched memory B cells % (of CD19+ memory B cells)
CD19+ switched memory B cells % (of CD19+ memory B cells)
CD19+ activated SM B cells % (of CD19+ memory B cells)
CD19+ double negative B cells % (of CD19+ B cells)
CD19+ double negative 1 B cells % (of CD19+ double negative B cells)
CD19+ double negative 2 B cells % (of CD19+ double negative B cells)
CD19+ double negative 3 B cells % (of CD19+ double negative B cells)
CD19+ double negative 4 B cells % (of CD19+ double negative B cells)
CD19+ Naive B cells % (of CD19+ B cells)
CD19+ activated naive B cells % (of CD19+ naive B cells)
CD19+ anergic naive B cells % (of CD19+ naive B cells)
CD19+ resting naive B cells % (of CD19+ naive B cells)
CD19+ Ab secreting B cells % (of CD19+ B cells)
CD19+ Plasmablasts % (of CD19+ Ab secreting B cells)
CD19+ Plasmacells % (of CD19+ Ab secreting B cells)
CD19+ transitional T1/T2 B cells % (of CD19+ B cells)
CD19+ transitional T3a B cells % (of CD19+ B cells)
CD19+ MZP B cells % (of CD19+ B cells)
T0
T4M
CT arm

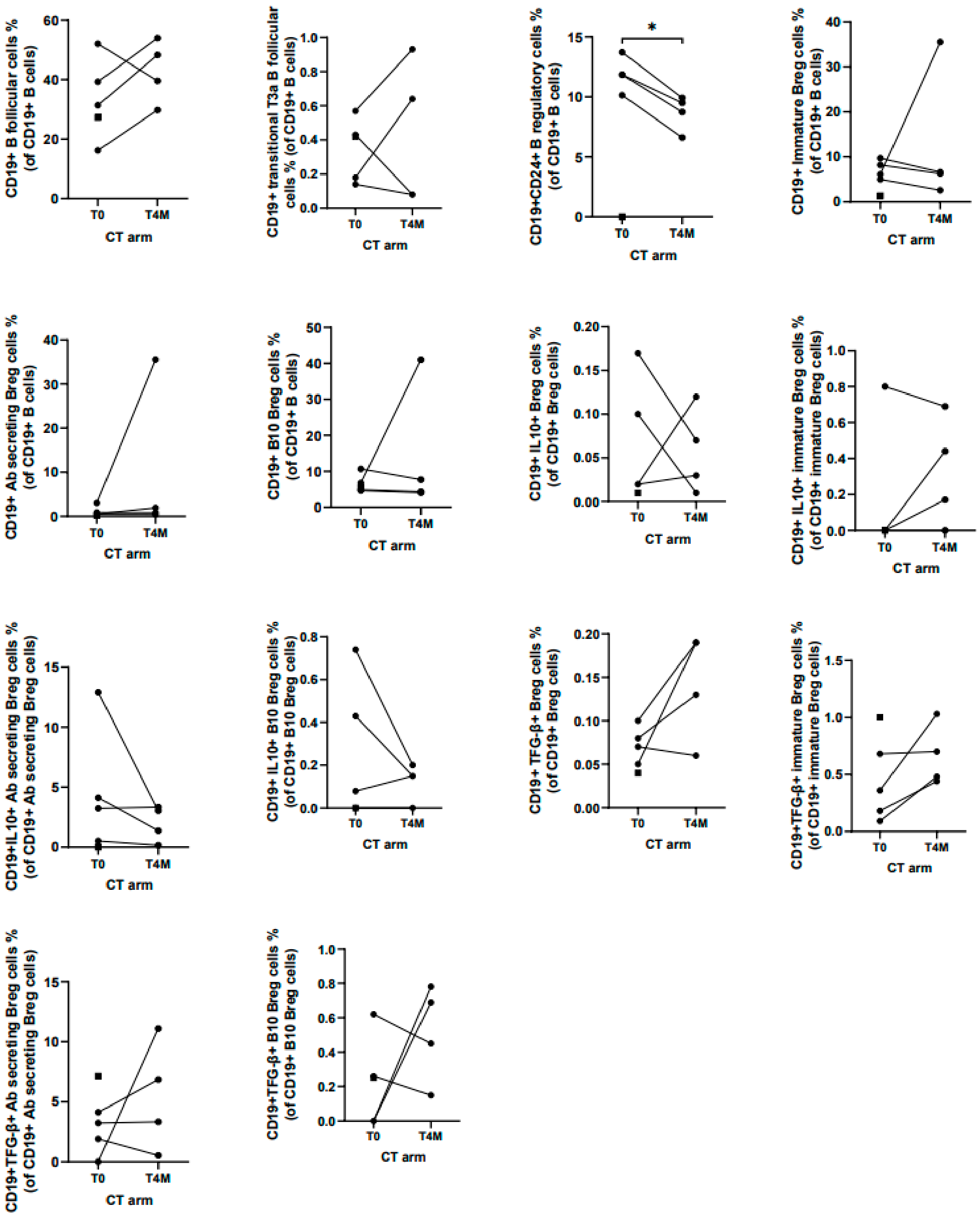
CD19+ B follicular cells % (of CD19+ B cells)
CD19+ transitional T3a B follicular cells % (of CD19+ B cells)
CD19+CD24+ B regulatory cells % (of CD19+ B cells)
CD19+ Immature Breg cells % (of CD19+ B cells)
CD19+ Ab secreting Breg cells % (of CD19+ B cells)
CD19+ B10 Breg cells % (of CD19+ B cells)
CD19+ IL10+ Breg cells % (of CD19+ Breg cells)
CD19+ IL10+ immature Breg cells % (of CD19+ immature Breg cells)
CD19+IL10+ Ab secreting Breg cells % (of CD19+ Ab secreting Breg cells)
CD19+ IL10+ B10 Breg cells % (of CD19+ B10 Breg cells)
CD19+ TFG-β+ Breg cells % (of CD19+ Breg cells)
CD19+TFG-β+ immature Breg cells % (of CD19+ immature Breg cells)
CD19+TFG-β+ Ab secreting Breg cells % (of CD19+ Ab secreting Breg cells)
CD19+TFG-β+ B10 Breg cells % (of CD19+ B10 Breg cells)
T0
T4M
CT arm
*

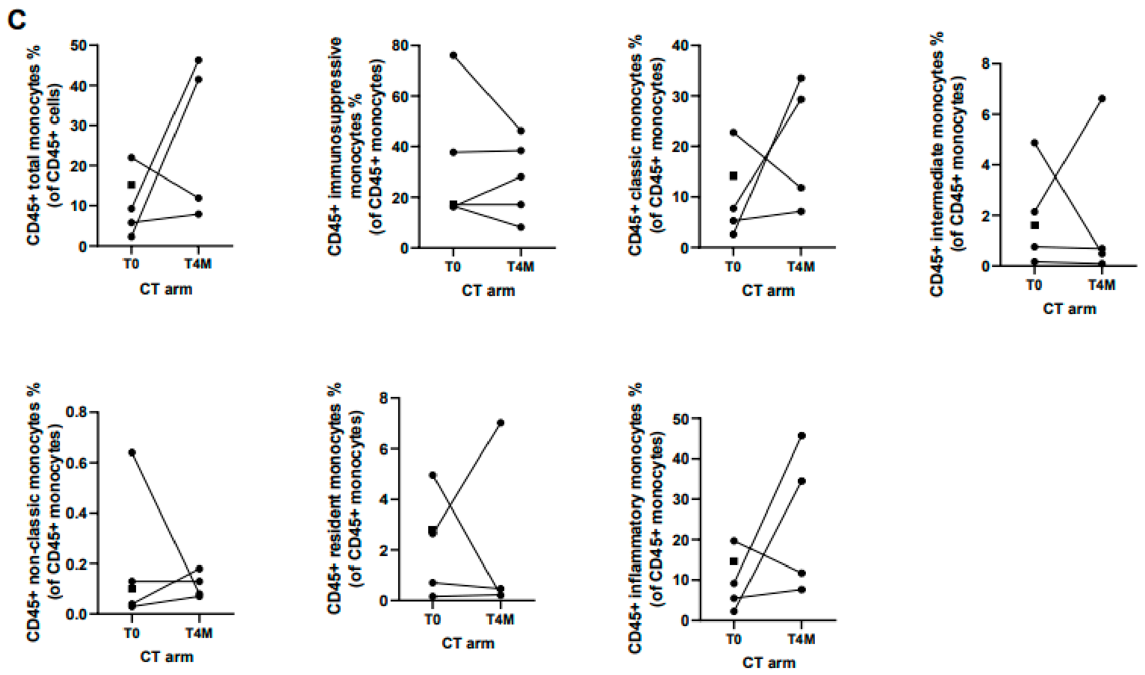
C
CD45+ total monocytes % (of CD45+ cells)
CD45+ immunosuppressive monocytes % (of CD45+ monocytes)
CD45+ classic monocytes % (of CD45+ monocytes)
CD45+ intermediate monocytes % (of CD45+ monocytes)
CD45+ non-classic monocytes % (of CD45+ monocytes)
CD45+ resident monocytes % (of CD45+ monocytes)
CD45+ inflammatory monocytes % (of CD45+ monocytes)
T0
T4M
CT arm

553

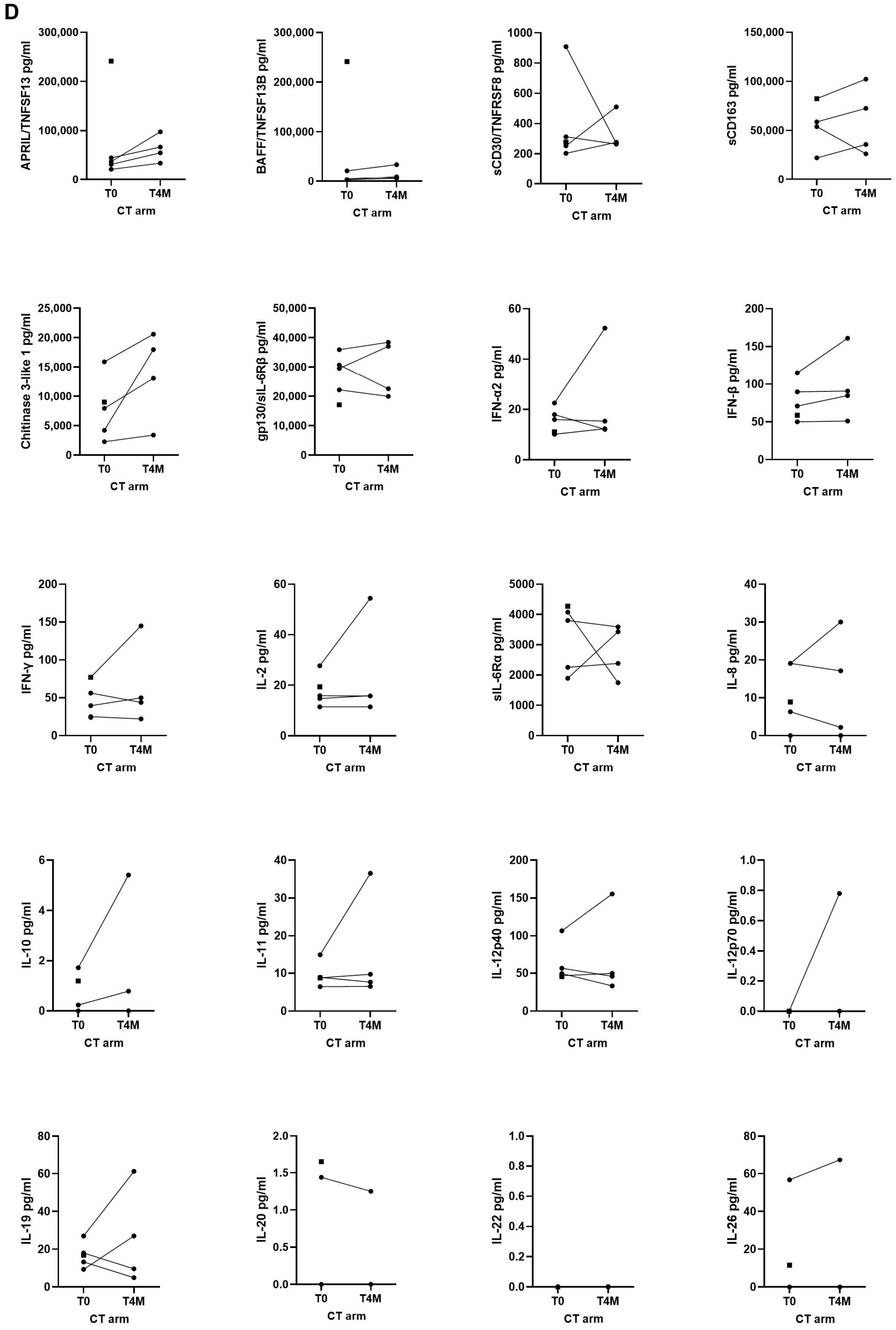
D
APRIL/TNFSF13 pg/ml
BAFF/TNFSF13B pg/ml
sCD30/TNFRSF8 pg/ml
sCD163 pg/ml
Chitinase 3-like 1 pg/ml
gp130/sIL-6Rβ pg/ml
IFN-α2 pg/ml
IFN-β pg/ml
IFN-γ pg/ml
IL-2 pg/ml
sIL-6Rα pg/ml
IL-8 pg/ml
IL-10 pg/ml
IL-11 pg/ml
IL-12p40 pg/ml
IL-12p70 pg/ml
IL-19 pg/ml
IL-20 pg/ml
IL-22 pg/ml
IL-26 pg/ml
T0
T4M
CT arm

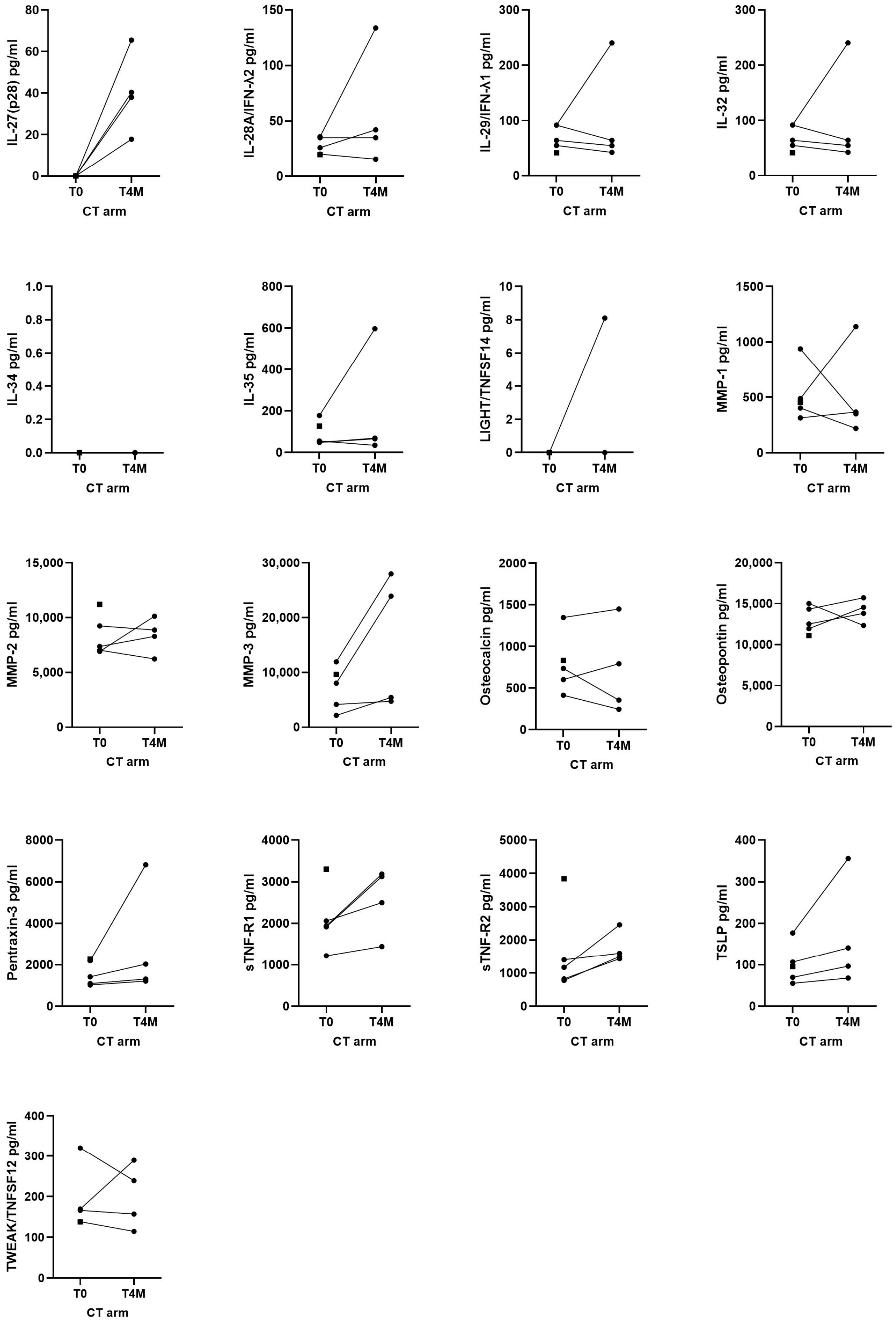
IL-27(p28) pg/ml
IL-28A/IFN-λ2 pg/ml
IL-29/IFN-λ1 pg/ml
IL-32 pg/ml
IL-34 pg/ml
IL-35 pg/ml
LIGHT/TNFSF14 pg/ml
MMP-1 pg/ml
MMP-2 pg/ml
MMP-3 pg/ml
Osteocalcin pg/ml
Osteopontin pg/ml
Pentraxin-3 pg/ml
sTNF-R1 pg/ml
sTNF-R2 pg/ml
TSLP pg/ml
TWEAK/TNFSF12 pg/ml
T0
T4M
CT arm

E

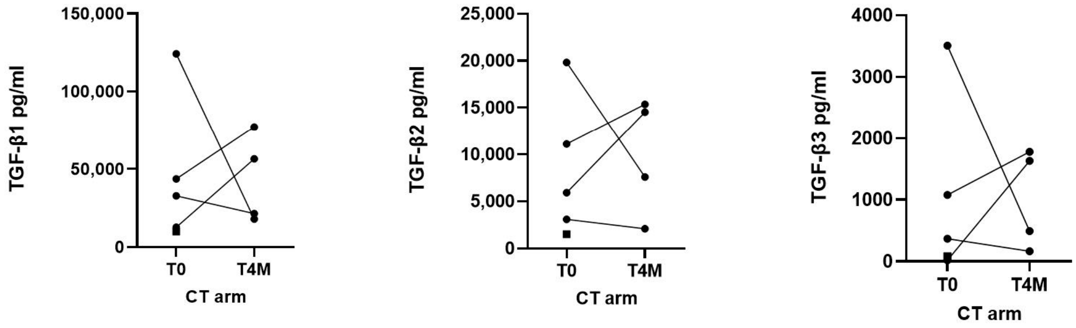
150,000
100,000
50,000
0
TGF-β1 pg/ml
T0
T4M
CT arm
25,000
20,000
15,000
10,000
5,000
0
TGF-β2 pg/ml
T0
T4M
CT arm
4000
3000
2000
1000
0
TGF-β3 pg/ml
T0
T4M
CT arm

558

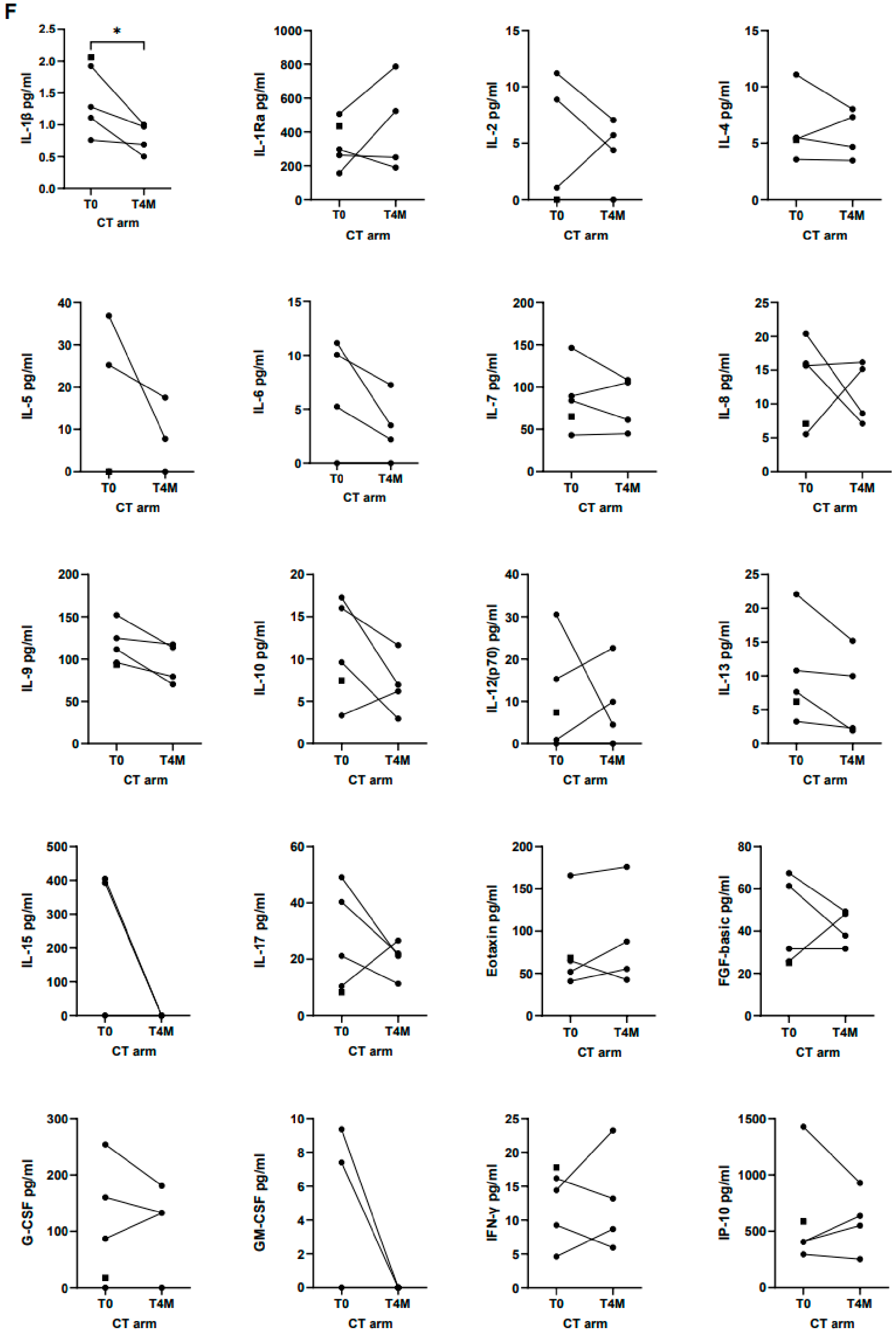
F
*
IL-1β pg/ml
IL-1Ra pg/ml
IL-2 pg/ml
IL-4 pg/ml
IL-5 pg/ml
IL-6 pg/ml
IL-7 pg/ml
IL-8 pg/ml
IL-9 pg/ml
IL-10 pg/ml
IL-12(p70) pg/ml
IL-13 pg/ml
IL-15 pg/ml
IL-17 pg/ml
Eotaxin pg/ml
FGF-basic pg/ml
G-CSF pg/ml
GM-CSF pg/ml
IFN-γ pg/ml
IP-10 pg/ml
T0
T4M
CT arm

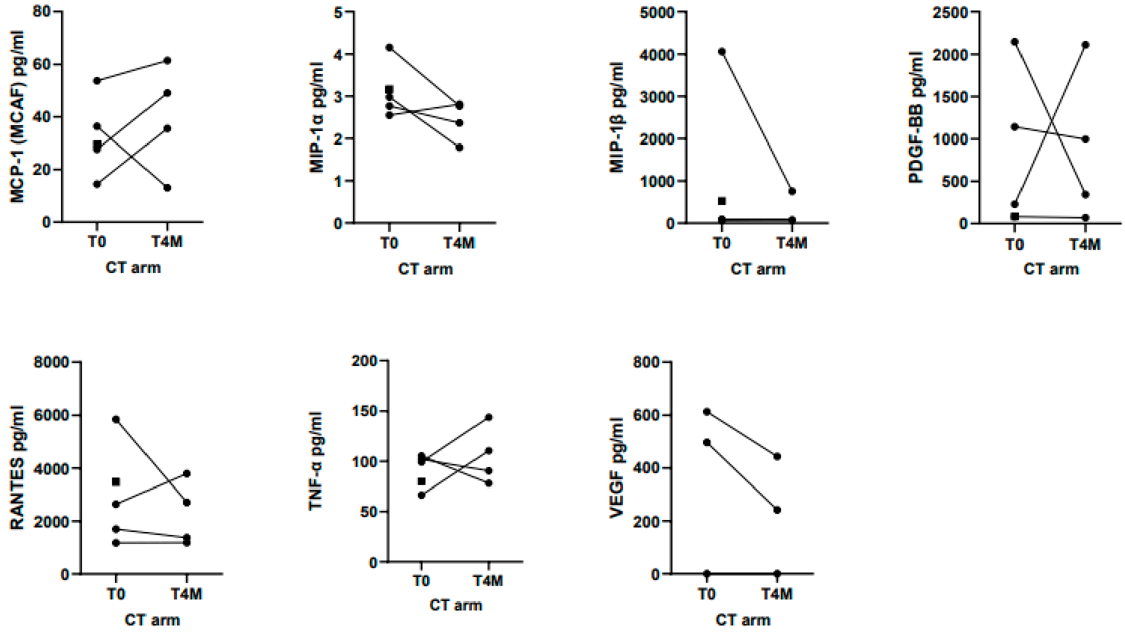

Footnote: Te: T effector; Th1: T helper 1; Th2: T helper 2; Th17: T helper 17; Th1/17: T helper 1/17; Tfh: T follicular helper; Tfh1: T follicular helper 1; Tfh2: T follicular helper 2; Tfh17: T follicular helper 17; Tfh1/17: T follicular helper 1/17; Treg: T regulatory; UM: unswitched memory; SM: switched memory; ASM: activated switched memory; DN: double negative; DN1: double negative 1; DN2: double negative 2; DN3: double negative 3; DN4: double negative 4; AcN: activated naïve; AnN: anergic naïve; RN: resting naïve; Ab: antibody secreting; T1/2: transitional 1/2; T3a: transitional 3a; MZP: marginal zone peripheral; Breg: B regulatory; AbS: antibody secreting; Imm: immature; Mon: monocytes.

Figure S13. Differences of circulating immune cells (A: T-cell compartment; B: B-cell compartment; C: monocyte compartment) and concentrations of inflammatory and immune-related cytokines and chemokines (D: Pro Human Inflammation Panel I Assay; E: Pro TGF-β Immunoassay; F: Pro Human Cytokine Immunoassay) between patients with pancreatic adenocarcinoma (PDAC) treated with HybridTherm Probe ablation plus chemotherapy (HTP-CT arm) and those treated with chemotherapy only (CT arm) at 4-months follow-up after therapy onset. Inter-group variables were compared using the Welch two sample t-test: * = $p < 0.05$.

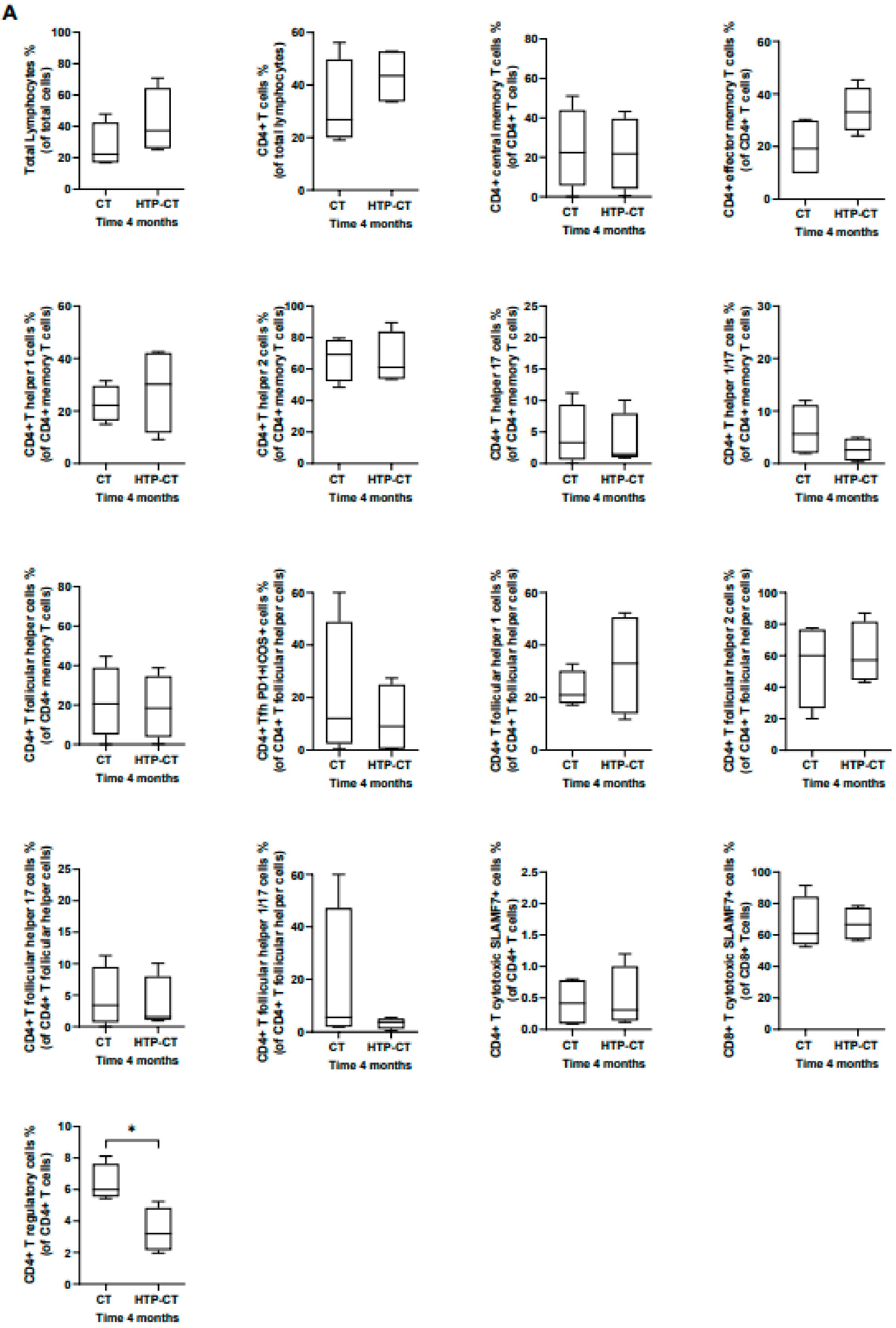
A
Total Lymphocytes % (of total cells)
CD4+ T cells % (of total lymphocytes)
CD4+ central memory T cells % (of CD4+ T cells)
CD4+ effector memory T cells % (of CD4+ T cells)
CD4+ T helper 1 cells % (of CD4+ memory T cells)
CD4+ T helper 2 cells % (of CD4+ memory T cells)
CD4+ T helper 17 cells % (of CD4+ memory T cells)
CD4+ T helper 1/17 cells % (of CD4+ memory T cells)
CD4+ T follicular helper cells % (of CD4+ memory T cells)
CD4+ Tfh PD1+ICOS+ cells % (of CD4+ T follicular helper cells)
CD4+ T follicular helper 1 cells % (of CD4+ T follicular helper cells)
CD4+ T follicular helper 2 cells % (of CD4+ T follicular helper cells)
CD4+ T follicular helper 17 cells % (of CD4+ T follicular helper cells)
CD4+ T follicular helper 1/17 cells % (of CD4+ T follicular helper cells)
CD4+ T cytotoxic SLAMF7+ cells % (of CD4+ T cells)
CD8+ T cytotoxic SLAMF7+ cells % (of CD8+ T cells)
CD4+ T regulatory cells % (of CD4+ T cells)
CT
HTP-CT
Time 4 months
*

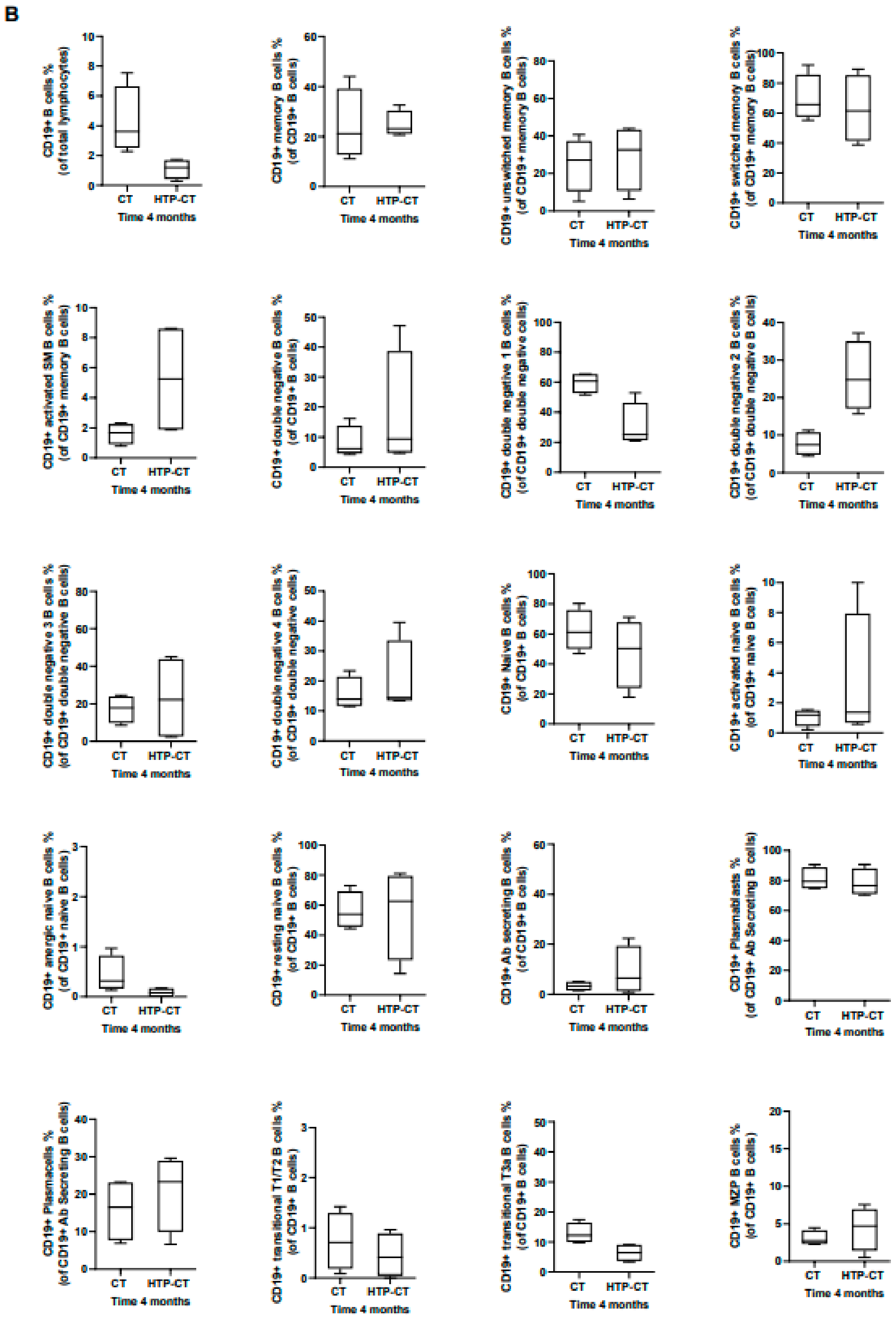
B
CD19+ B cells % (of total lymphocytes)
CD19+ memory B cells % (of CD19+ B cells)
CD19+ unswitched memory B cells % (of CD19+ memory B cells)
CD19+ switched memory B cells % (of CD19+ memory B cells)
CD19+ activated SM B cells % (of CD19+ memory B cells)
CD19+ double negative B cells % (of CD19+ B cells)
CD19+ double negative 1 B cells % (of CD19+ double negative cells)
CD19+ double negative 2 B cells % (of CD19+ double negative B cells)
CD19+ double negative 3 B cells % (of CD19+ double negative B cells)
CD19+ double negative 4 B cells % (of CD19+ double negative cells)
CD19+ Naive B cells % (of CD19+ B cells)
CD19+ activated naive B cells % (of CD19+ naive B cells)
CD19+ anergic naive B cells % (of CD19+ naive B cells)
CD19+ resting naive B cells % (of CD19+ B cells)
CD19+ Ab secreting B cells % (of CD19+ B cells)
CD19+ Plasmablasts % (of CD19+ Ab Secreting B cells)
CD19+ Plasmacells % (of CD19+ Ab Secreting B cells)
CD19+ transitional T1/T2 B cells % (of CD19+ B cells)
CD19+ transitional T3a B cells % (of CD19+ B cells)
CD19+ MZP B cells % (of CD19+ B cells)
CT
HTP-CT
Time 4 months

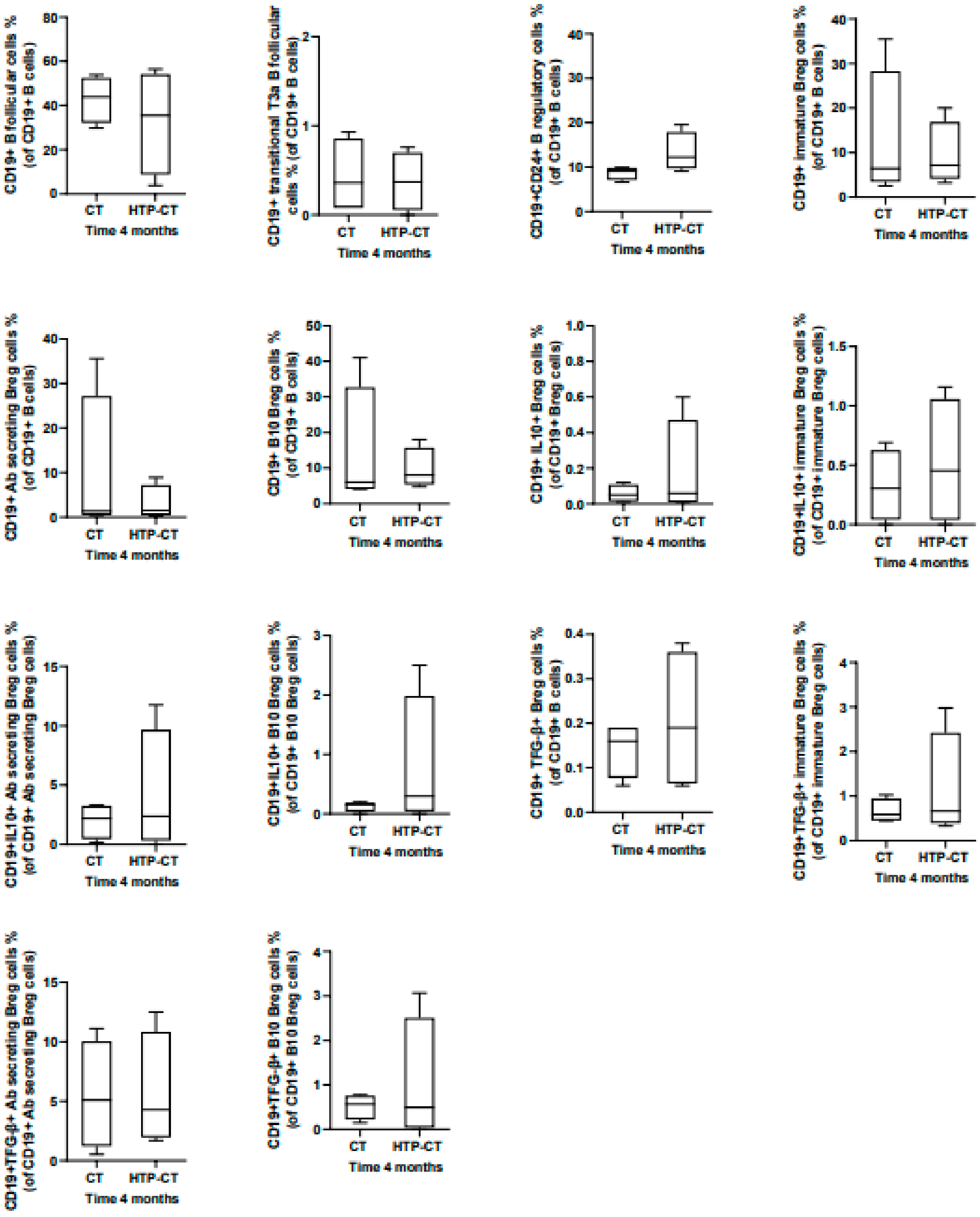
CD19+ B follicular cells % (of CD19+ B cells)
CD19+ transitional T3a B follicular cells % (of CD19+ B cells)
CD19+CD24+ B regulatory cells % (of CD19+ B cells)
CD19+ immature Breg cells % (of CD19+ B cells)
CD19+ Ab secreting Breg cells % (of CD19+ B cells)
CD19+ B10 Breg cells % (of CD19+ B cells)
CD19+ IL10+ Breg cells % (of CD19+ Breg cells)
CD19+IL10+ immature Breg cells % (of CD19+ immature Breg cells)
CD19+IL10+ Ab secreting Breg cells % (of CD19+ Ab secreting Breg cells)
CD19+IL10+ B10 Breg cells % (of CD19+ B10 Breg cells)
CD19+ TFG-β+ Breg cells % (of CD19+ B cells)
CD19+TFG-β+ immature Breg cells % (of CD19+ immature Breg cells)
CD19+TFG-β+ Ab secreting Breg cells % (of CD19+ Ab secreting Breg cells)
CD19+TFG-β+ B10 Breg cells % (of CD19+ B10 Breg cells)
CT
HTP-CT
Time 4 months

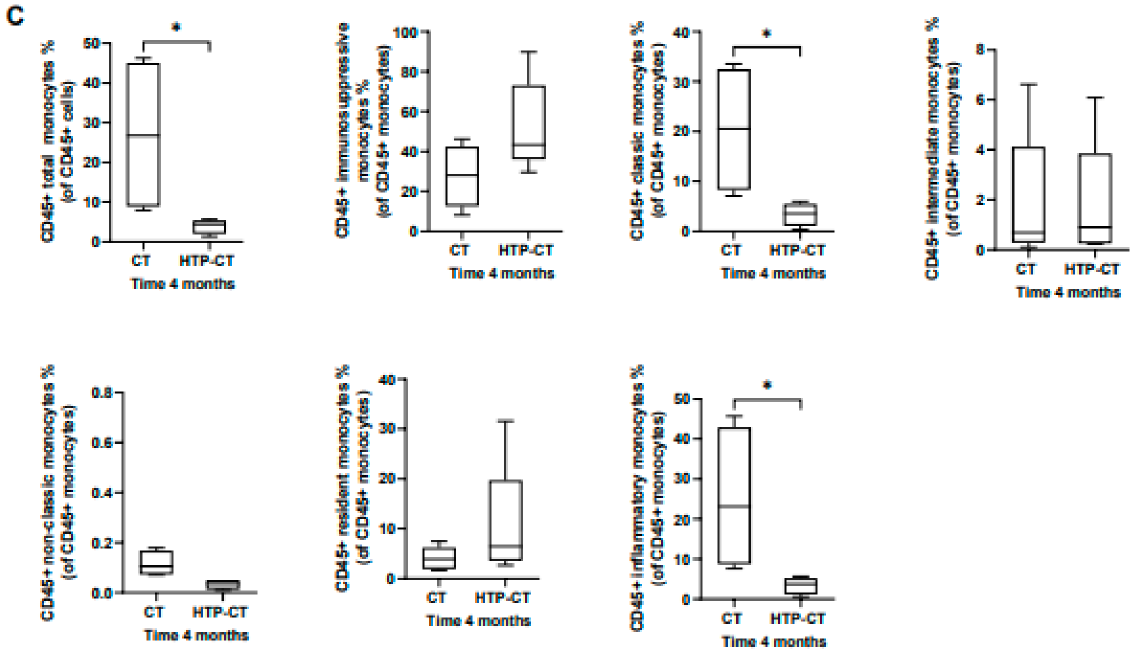
C
CD45+ total monocytes % (of CD45+ cells)
CD45+ immunosuppressive monocytes % (of CD45+ monocytes)
CD45+ classic monocytes % (of CD45+ monocytes)
CD45+ intermediate monocytes % (of CD45+ monocytes)
CD45+ non-classic monocytes % (of CD45+ monocytes)
CD45+ resident monocytes % (of CD45+ monocytes)
CD45+ inflammatory monocytes % (of CD45+ monocytes)
CT
HTP-CT
Time 4 months
*

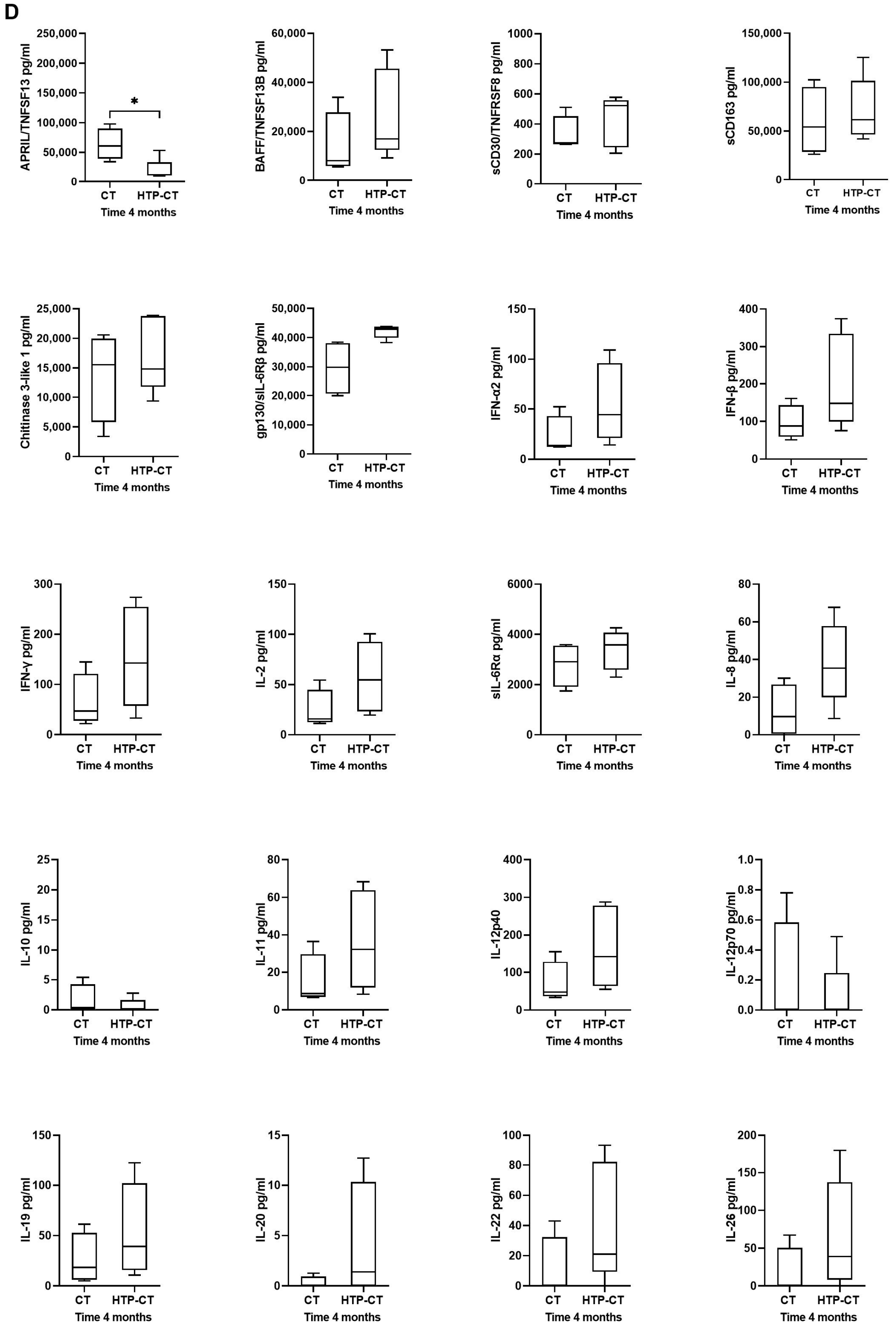
D
APRIL/TNFSF13 pg/ml
*
BAFF/TNFSF13B pg/ml
sCD30/TNFRSF8 pg/ml
sCD163 pg/ml
Chitinase 3-like 1 pg/ml
gp130/sIL-6Rβ pg/ml
IFN-α2 pg/ml
IFN-β pg/ml
IFN-γ pg/ml
IL-2 pg/ml
sIL-6Rα pg/ml
IL-8 pg/ml
IL-10 pg/ml
IL-11 pg/ml
IL-12p40
IL-12p70 pg/ml
IL-19 pg/ml
IL-20 pg/ml
IL-22 pg/ml
IL-26 pg/ml
CT
HTP-CT
Time 4 months

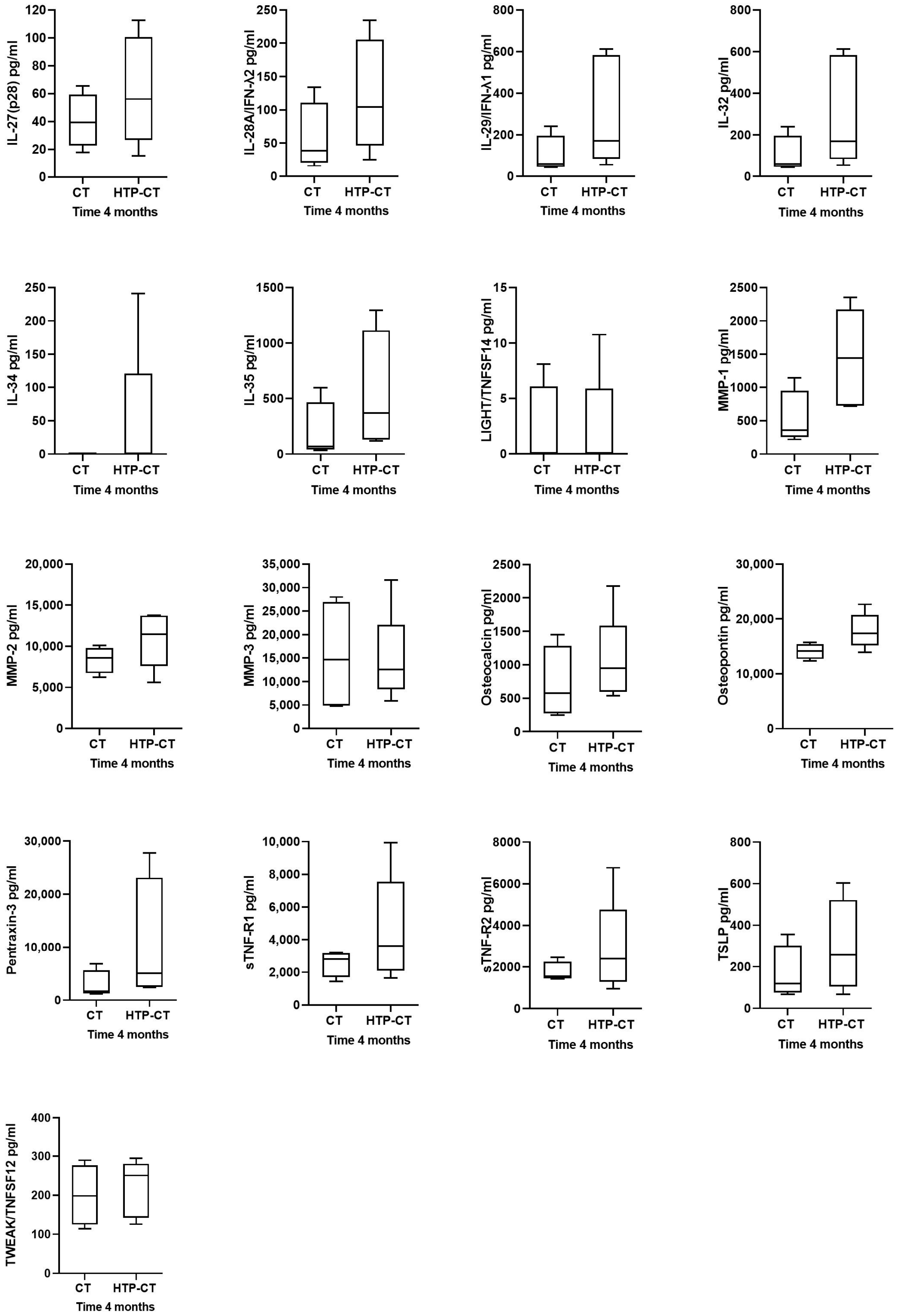
IL-27(p28) pg/ml
CT HTP-CT
Time 4 months
IL-28A/IFN-λ2 pg/ml
CT HTP-CT
Time 4 months
IL-29/IFN-λ1 pg/ml
CT HTP-CT
Time 4 months
IL-32 pg/ml
CT HTP-CT
Time 4 months
IL-34 pg/ml
CT HTP-CT
Time 4 months
IL-35 pg/ml
CT HTP-CT
Time 4 months
LIGHT/TNFSF14 pg/ml
CT HTP-CT
Time 4 months
MMP-1 pg/ml
CT HTP-CT
Time 4 months
MMP-2 pg/ml
CT HTP-CT
Time 4 months
MMP-3 pg/ml
CT HTP-CT
Time 4 months
Osteocalcin pg/ml
CT HTP-CT
Time 4 months
Osteopontin pg/ml
CT HTP-CT
Time 4 months
Pentraxin-3 pg/ml
CT HTP-CT
Time 4 months
sTNF-R1 pg/ml
CT HTP-CT
Time 4 months
sTNF-R2 pg/ml
CT HTP-CT
Time 4 months
TSLP pg/ml
CT HTP-CT
Time 4 months
TWEAK/TNFSF12 pg/ml
CT HTP-CT
Time 4 months

E

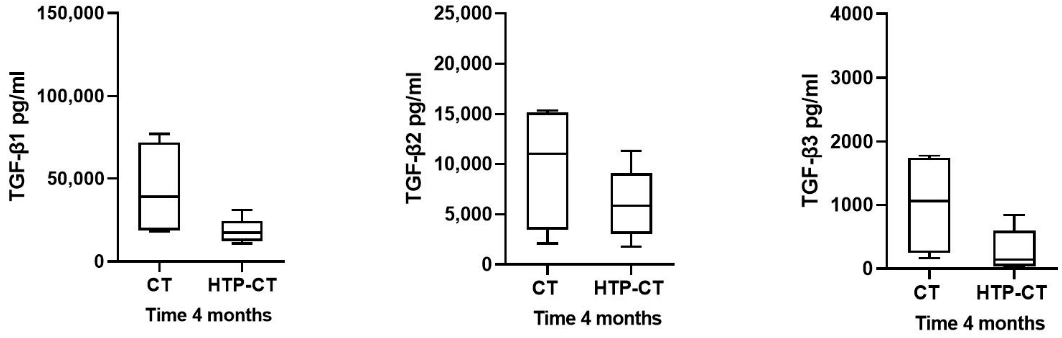
TGF-β1 pg/ml
150,000
100,000
50,000
0
CT
HTP-CT
Time 4 months
TGF-β2 pg/ml
25,000
20,000
15,000
10,000
5,000
0
CT
HTP-CT
Time 4 months
TGF-β3 pg/ml
4000
3000
2000
1000
0
CT
HTP-CT
Time 4 months

599

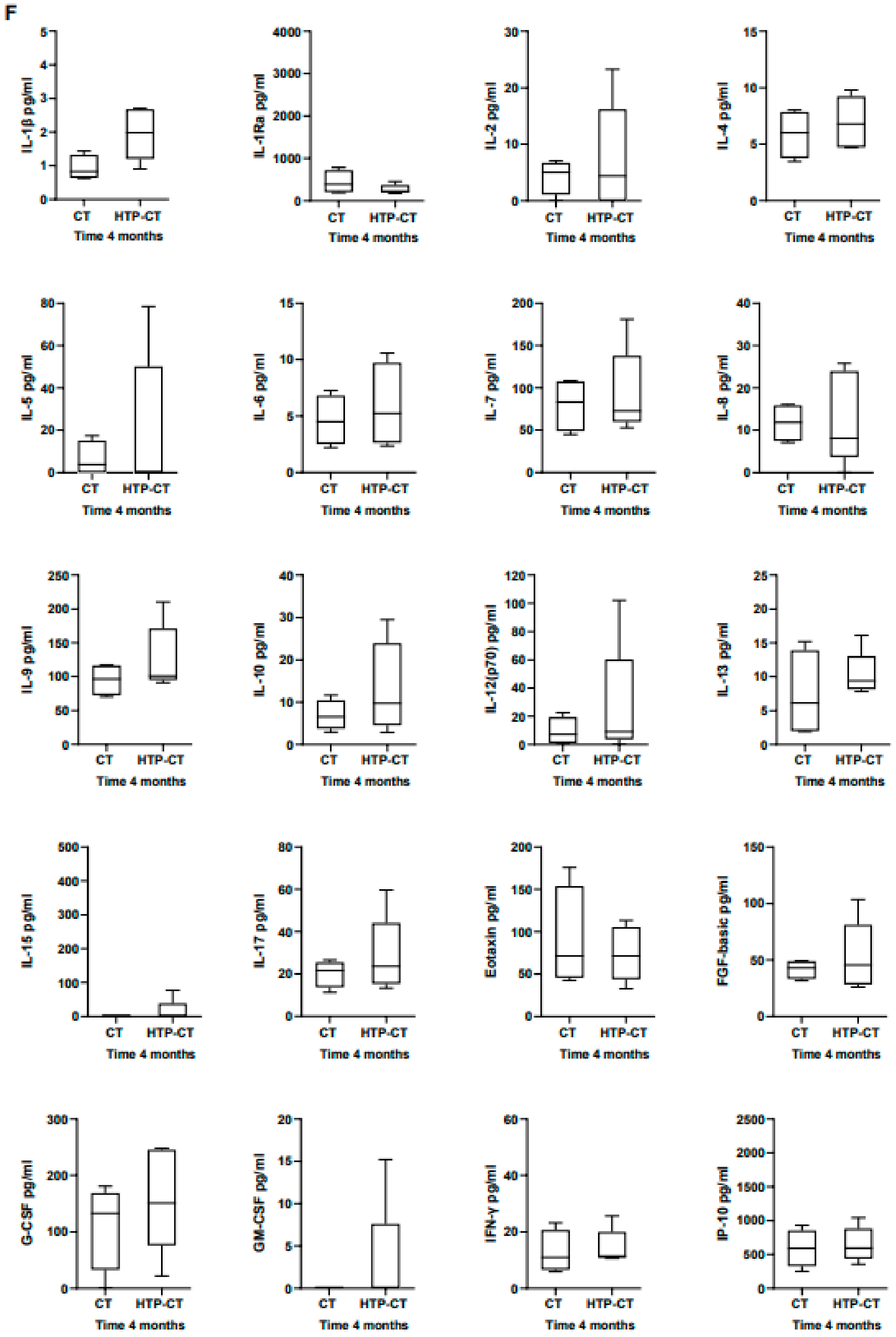
F
IL-1β pg/ml
IL-1Ra pg/ml
IL-2 pg/ml
IL-4 pg/ml
IL-5 pg/ml
IL-6 pg/ml
IL-7 pg/ml
IL-8 pg/ml
IL-9 pg/ml
IL-10 pg/ml
IL-12(p70) pg/ml
IL-13 pg/ml
IL-15 pg/ml
IL-17 pg/ml
Eotaxin pg/ml
FGF-basic pg/ml
G-CSF pg/ml
GM-CSF pg/ml
IFN-γ pg/ml
IP-10 pg/ml
CT
HTP-CT
Time 4 months

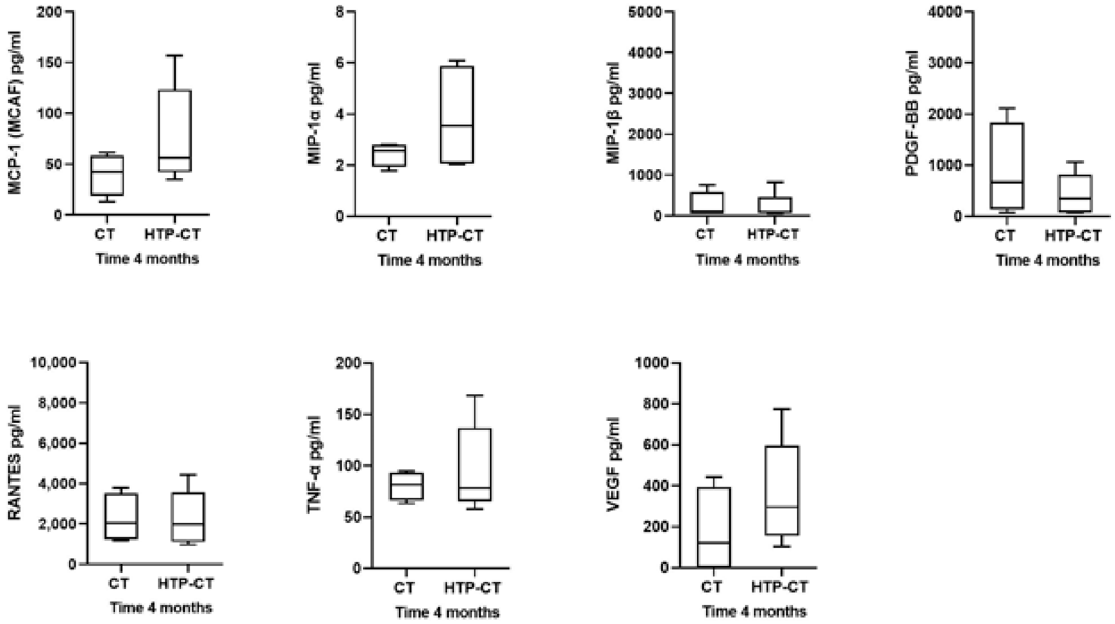

Footnote: Te: T effector; Th1: T helper 1; Th2: T helper 2; Th17: T helper 17; Th1/17: T helper 1/17; Tfh: T follicular helper; Tfh1: T follicular helper 1; Tfh2: T follicular helper 2; Tfh17: T follicular helper 17; Tfh1/17: T follicular helper 1/17; Treg: T regulatory; UM: unswitched memory; SM: switched memory; ASM: activated switched memory; DN: double negative; DN1: double negative 1; DN2: double negative 2; DN3: double negative 3; DN4: double negative 4; AcN: activated naïve; AnN: anergic naïve; RN: resting naïve; Ab: antibody secreting; T1/2: transitional 1/2; T3a: transitional 3a; MZP: marginal zone peripheral; Breg: B regulatory; AbS: antibody secreting; Imm: immature; Mon: monocytes.

Figure S14. Changes of circulating immune cells (A: T-cell compartment; B: B-cell compartment; C: monocyte compartment) and concentrations of inflammatory and immune-related cytokines and chemokines (D: Pro Human Inflammation Panel I Assay; E: Pro TGF-β Immunoassay; F: Pro Human Cytokine Immunoassay) between baseline (T0) and 4 months (T4M) after therapy onset in patients with pancreatic adenocarcinoma (PDAC) treated with HybridTherm Probe ablation plus chemotherapy (HTP-CT arm). Intra-group variables were compared using the paired t-test: * = $p < 0.05$.

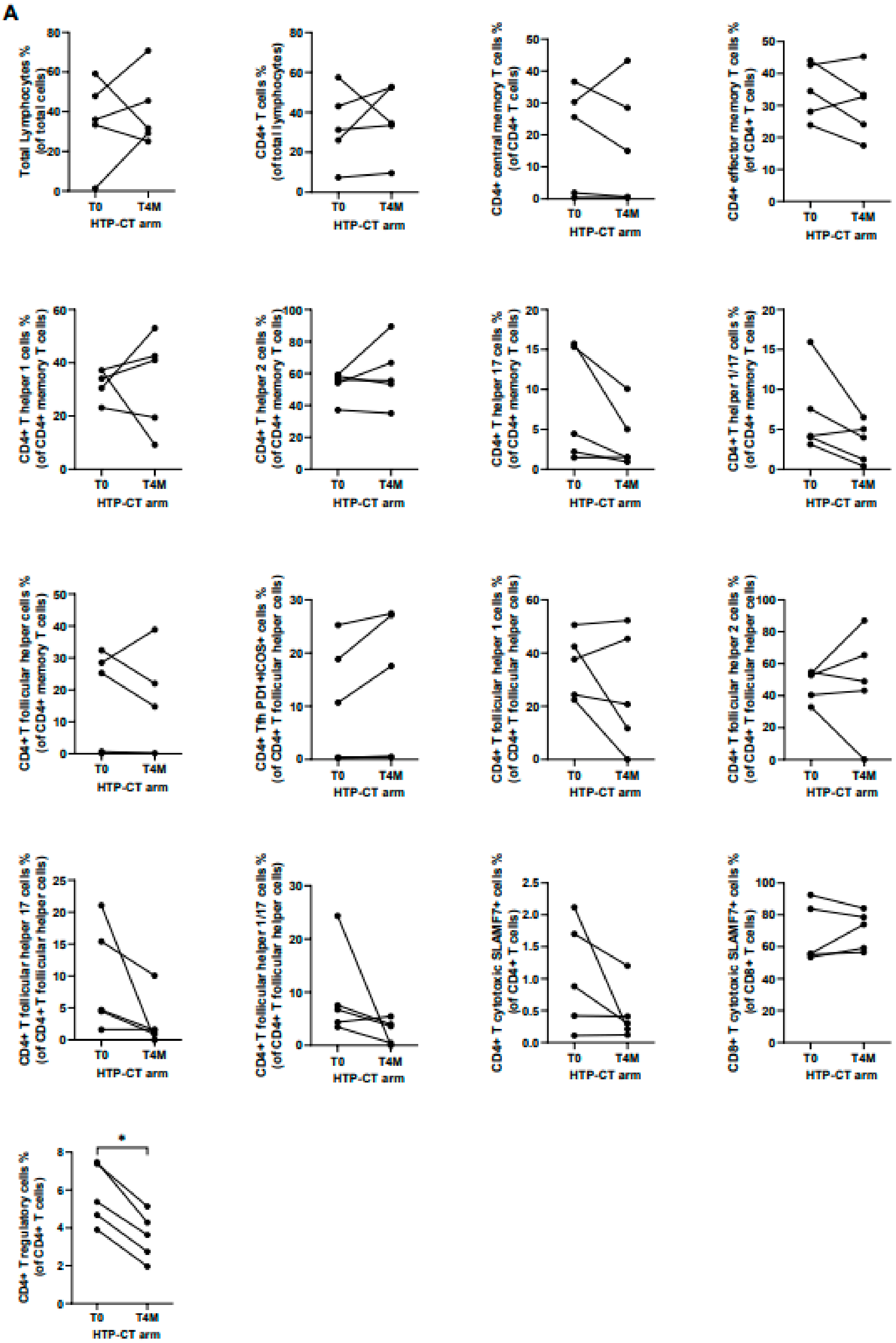
A
Total Lymphocytes % (of total cells)
CD4+ T cells % (of total lymphocytes)
CD4+ central memory T cells % (of CD4+ T cells)
CD4+ effector memory T cells % (of CD4+ T cells)
CD4+ T helper 1 cells % (of CD4+ memory T cells)
CD4+ T helper 2 cells % (of CD4+ memory T cells)
CD4+ T helper 17 cells % (of CD4+ memory T cells)
CD4+ T helper 1/17 cells % (of CD4+ memory T cells)
CD4+ T follicular helper cells % (of CD4+ memory T cells)
CD4+ Tfh PD1+ICOS+ cells % (of CD4+ T follicular helper cells)
CD4+ T follicular helper 1 cells % (of CD4+ T follicular helper cells)
CD4+ T follicular helper 2 cells % (of CD4+ T follicular helper cells)
CD4+ T follicular helper 17 cells % (of CD4+ T follicular helper cells)
CD4+ T follicular helper 1/17 cells % (of CD4+ T follicular helper cells)
CD4+ T cytotoxic SLAMF7+ cells % (of CD4+ T cells)
CD8+ T cytotoxic SLAMF7+ cells % (of CD8+ T cells)
CD4+ T regulatory cells % (of CD4+ T cells)
*
T0
T4M
HTP-CT arm

B

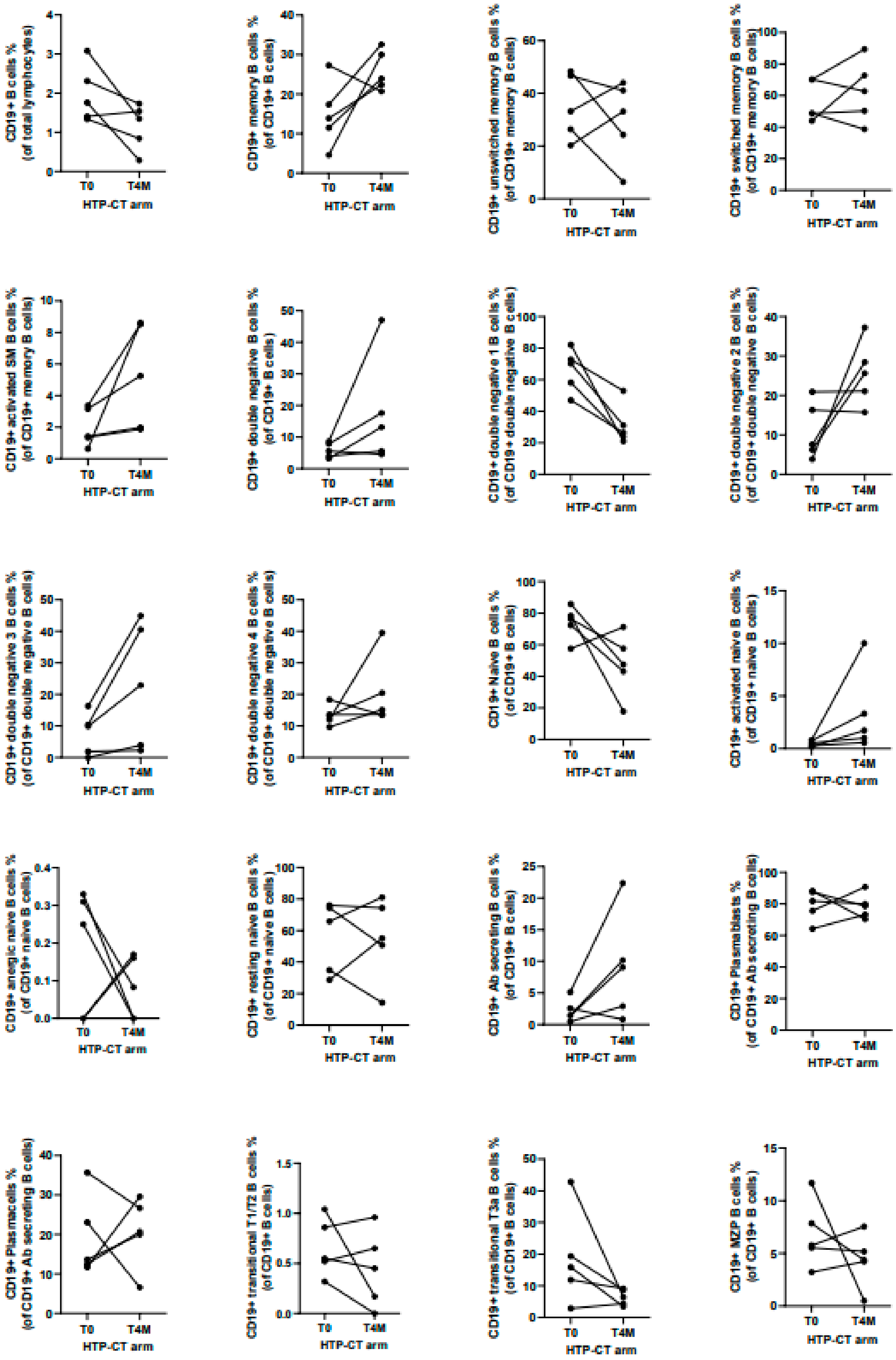
CD19+ B cells % (of total lymphocytes)
CD19+ memory B cells % (of CD19+ B cells)
CD19+ unswitched memory B cells % (of CD19+ memory B cells)
CD19+ switched memory B cells % (of CD19+ memory B cells)
CD19+ activated SM B cells % (of CD19+ memory B cells)
CD19+ double negative B cells % (of CD19+ B cells)
CD19+ double negative 1 B cells % (of CD19+ double negative B cells)
CD19+ double negative 2 B cells % (of CD19+ double negative B cells)
CD19+ double negative 3 B cells % (of CD19+ double negative B cells)
CD19+ double negative 4 B cells % (of CD19+ double negative B cells)
CD19+ Naive B cells % (of CD19+ B cells)
CD19+ activated naive B cells % (of CD19+ naive B cells)
CD19+ anergic naive B cells % (of CD19+ naive B cells)
CD19+ resting naive B cells % (of CD19+ naive B cells)
CD19+ Ab secreting B cells % (of CD19+ B cells)
CD19+ Plasmablasts % (of CD19+ Ab secreting B cells)
CD19+ Plasmacells % (of CD19+ Ab secreting B cells)
CD19+ transitional T1/T2 B cells % (of CD19+ B cells)
CD19+ transitional T3a B cells % (of CD19+ B cells)
CD19+ MZP B cells % (of CD19+ B cells)
T0
T4M
HTP-CT arm

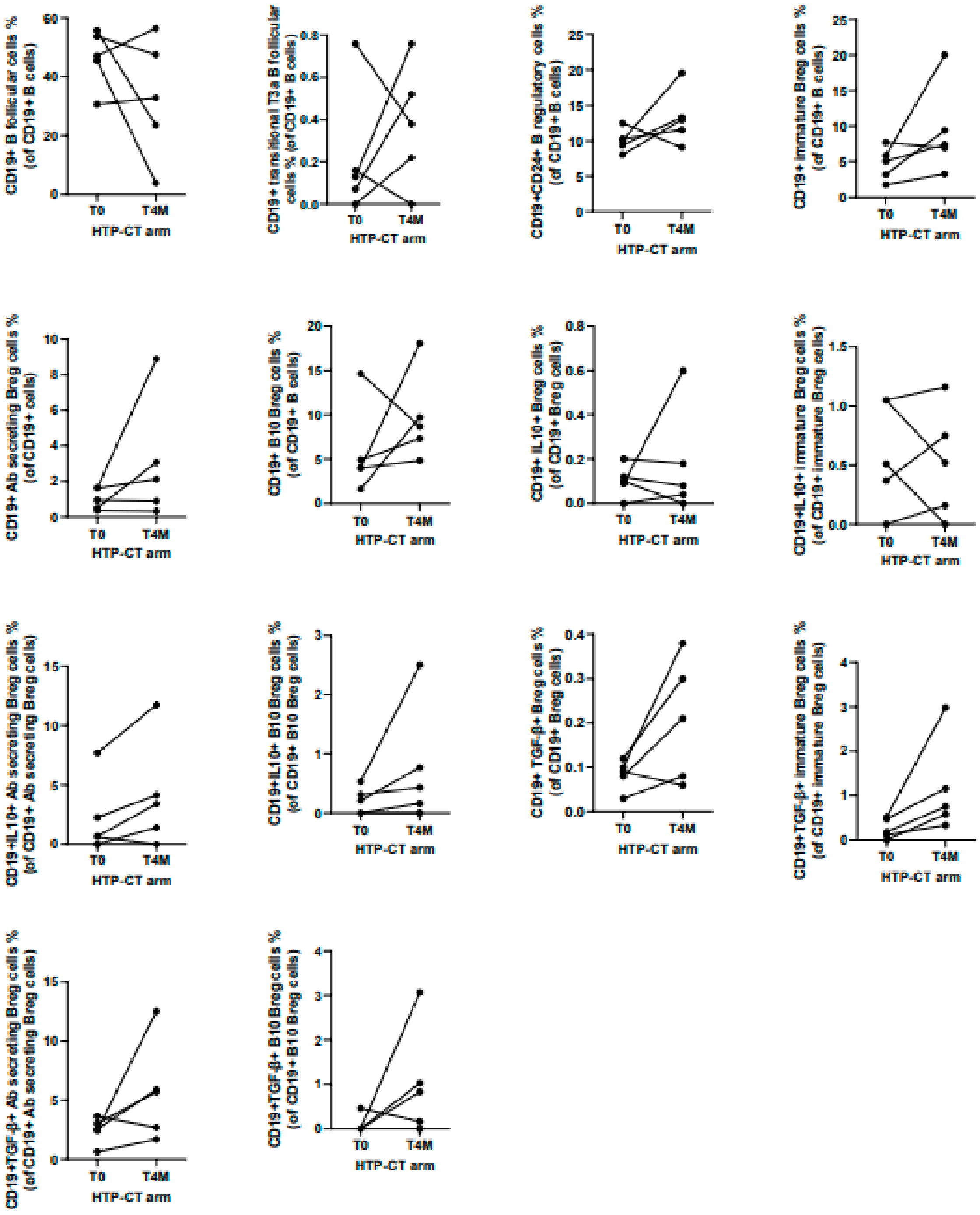
CD19+ B follicular cells % (of CD19+ B cells)
CD19+transitional T3a B follicular cells % (of CD19+ B cells)
CD19+CD24+ B regulatory cells % (of CD19+ B cells)
CD19+ immature Breg cells % (of CD19+ B cells)
CD19+ Ab secreting Breg cells % (of CD19+ cells)
CD19+ B10 Breg cells % (of CD19+ B cells)
CD19+ IL10+ Breg cells % (of CD19+ Breg cells)
CD19+IL10+ immature Breg cells % (of CD19+ immature Breg cells)
CD19+IL10+ Ab secreting Breg cells % (of CD19+ Ab secreting Breg cells)
CD19+IL10+ B10 Breg cells % (of CD19+ B10 Breg cells)
CD19+ TGF-β+ Breg cells % (of CD19+ Breg cells)
CD19+TGF-β+ immature Breg cells % (of CD19+ immature Breg cells)
CD19+TGF-β+ Ab secreting Breg cells % (of CD19+ Ab secreting Breg cells)
CD19+TGF-β+ B10 Breg cells % (of CD19+ B10 Breg cells)
T0
T4M
HTP-CT arm

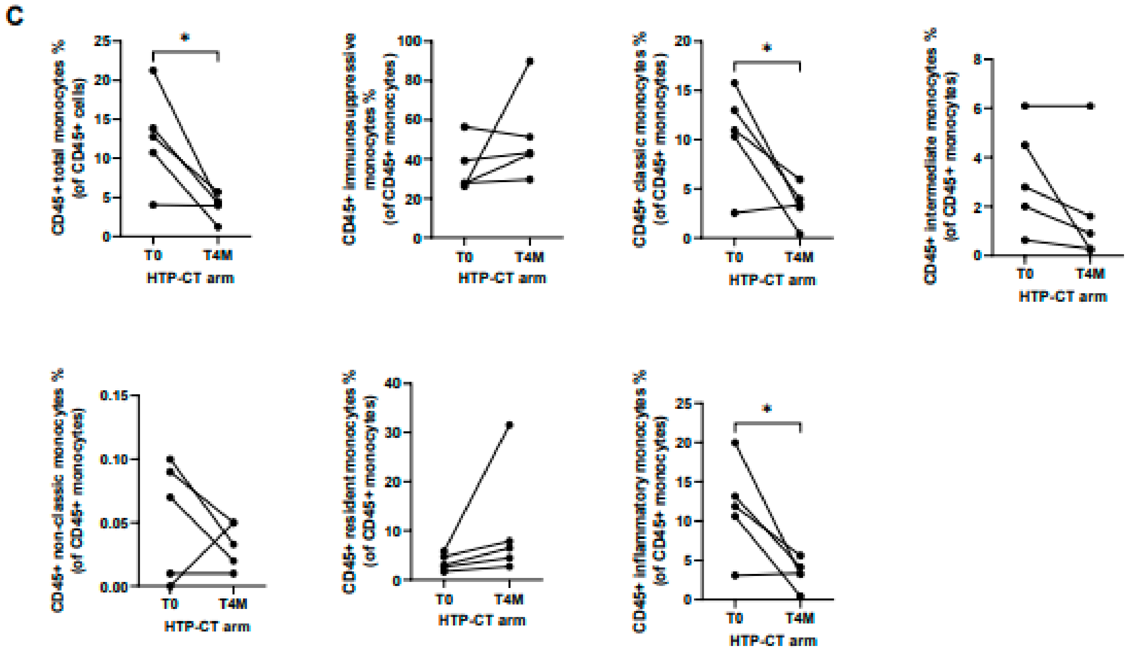
C
CD45+ total monocytes %
(of CD45+ cells)
CD45+ immunosuppressive monocytes %
(of CD45+ monocytes)
CD45+ classic monocytes %
(of CD45+ monocytes)
CD45+ intermediate monocytes %
(of CD45+ monocytes)
CD45+ non-classic monocytes %
(of CD45+ monocytes)
CD45+ resident monocytes %
(of CD45+ monocytes)
CD45+ inflammatory monocytes %
(of CD45+ monocytes)
T0
T4M
HTP-CT arm
*

D

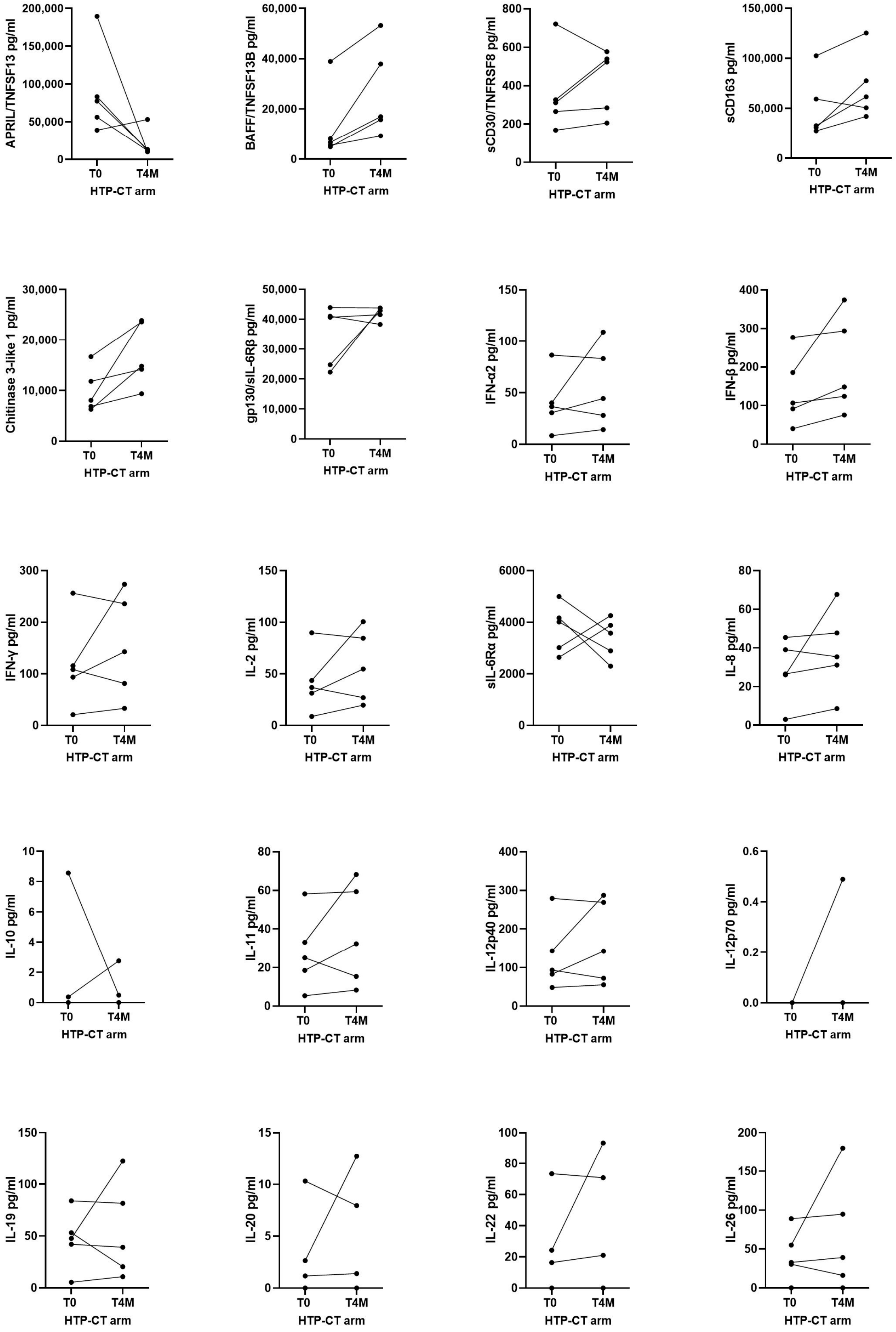
APRIL/TNFSF13 pg/ml
200,000
150,000
100,000
50,000
0
T0
T4M
HTP-CT arm
BAFF/TNFSF13B pg/ml
60,000
40,000
20,000
0
T0
T4M
HTP-CT arm
sCD30/TNFRSF8 pg/ml
800
600
400
200
0
T0
T4M
HTP-CT arm
sCD163 pg/ml
150,000
100,000
50,000
0
T0
T4M
HTP-CT arm
Chitinase 3-like 1 pg/ml
30,000
20,000
10,000
0
T0
T4M
HTP-CT arm
gp130/sIL-6Rβ pg/ml
50,000
40,000
30,000
20,000
10,000
0
T0
T4M
HTP-CT arm
IFN-α2 pg/ml
150
100
50
0
T0
T4M
HTP-CT arm
IFN-β pg/ml
400
300
200
100
0
T0
T4M
HTP-CT arm
IFN-γ pg/ml
300
200
100
0
T0
T4M
HTP-CT arm
IL-2 pg/ml
150
100
50
0
T0
T4M
HTP-CT arm
sIL-6Rα pg/ml
6000
4000
2000
0
T0
T4M
HTP-CT arm
IL-8 pg/ml
80
60
40
20
0
T0
T4M
HTP-CT arm
IL-10 pg/ml
10
8
6
4
2
0
T0
T4M
HTP-CT arm
IL-11 pg/ml
80
60
40
20
0
T0
T4M
HTP-CT arm
IL-12p40 pg/ml
400
300
200
100
0
T0
T4M
HTP-CT arm
IL-12p70 pg/ml
0.6
0.4
0.2
0.0
T0
T4M
HTP-CT arm
IL-19 pg/ml
150
100
50
0
T0
T4M
HTP-CT arm
IL-20 pg/ml
15
10
5
0
T0
T4M
HTP-CT arm
IL-22 pg/ml
100
80
60
40
20
0
T0
T4M
HTP-CT arm
IL-26 pg/ml
200
150
100
50
0
T0
T4M
HTP-CT arm

631

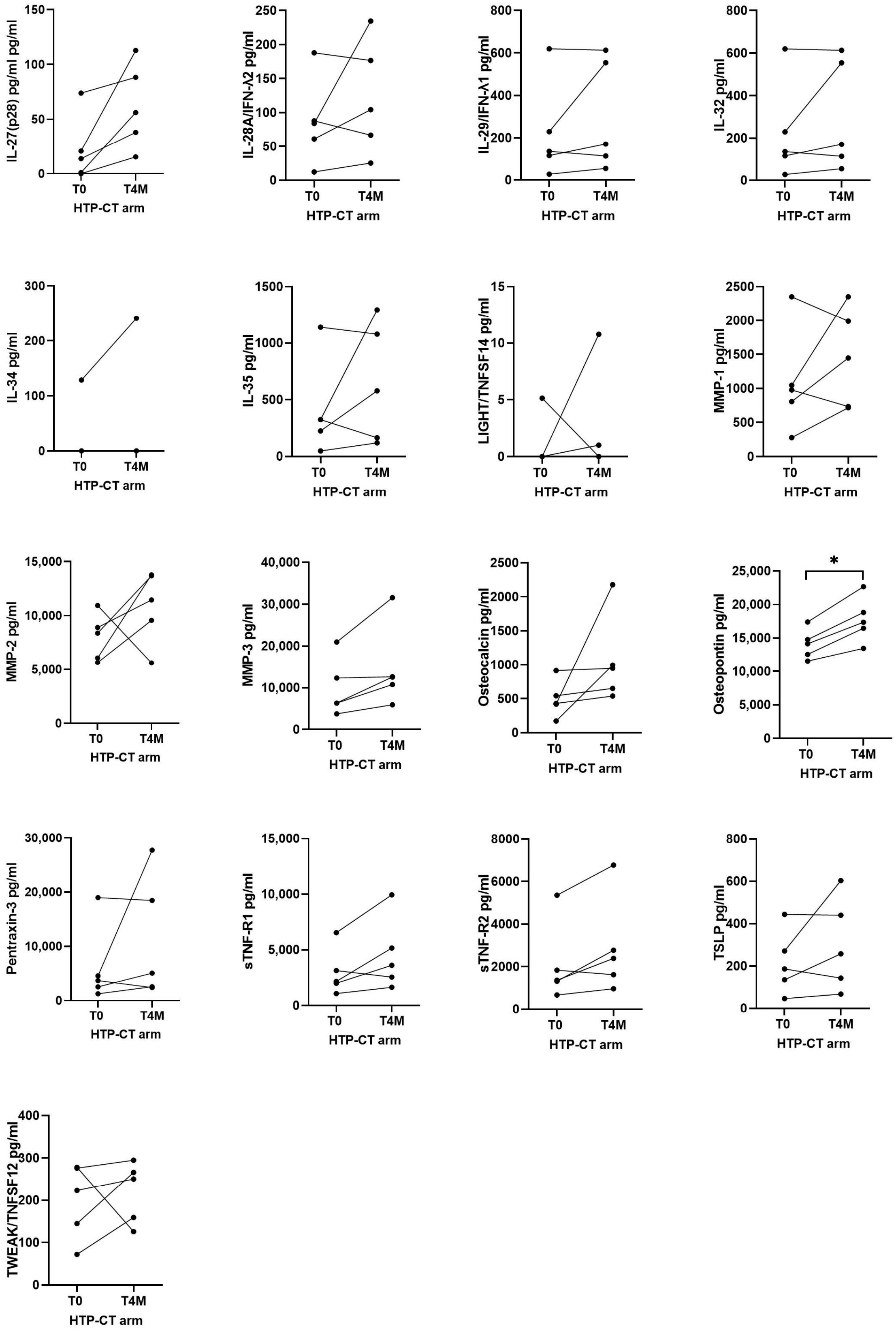
IL-27(p28) pg/ml pg/ml
IL-28A/IFN-λ2 pg/ml
IL-29/IFN-λ1 pg/ml
IL-32 pg/ml
IL-34 pg/ml
IL-35 pg/ml
LIGHT/TNFSF14 pg/ml
MMP-1 pg/ml
MMP-2 pg/ml
MMP-3 pg/ml
Osteocalcin pg/ml
Osteopontin pg/ml
*
Pentraxin-3 pg/ml
sTNF-R1 pg/ml
sTNF-R2 pg/ml
TSLP pg/ml
TWEAK/TNFSF12 pg/ml
T0
T4M
HTP-CT arm

E

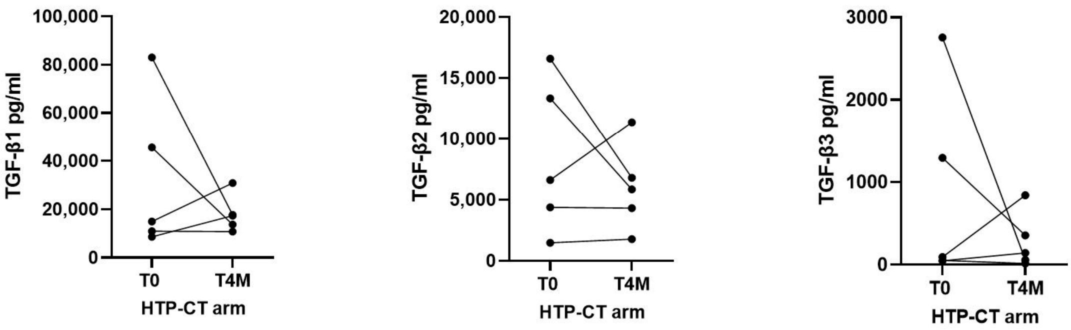
100,000
80,000
60,000
40,000
20,000
0
TGF-β1 pg/ml
T0
T4M
HTP-CT arm
20,000
15,000
10,000
5,000
0
TGF-β2 pg/ml
T0
T4M
HTP-CT arm
3000
2000
1000
0
TGF-β3 pg/ml
T0
T4M
HTP-CT arm

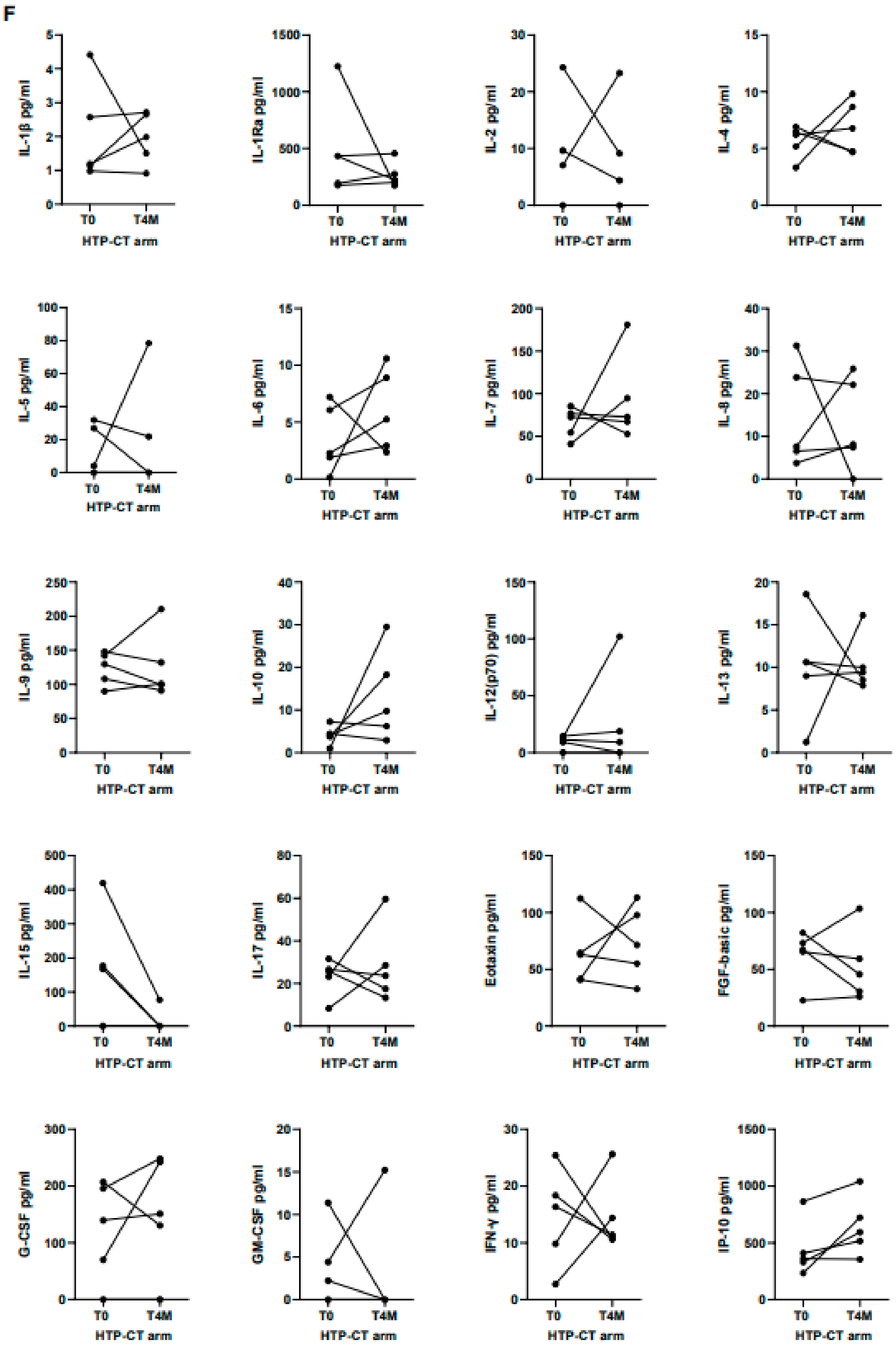
F
IL-1β pg/ml
IL-1Ra pg/ml
IL-2 pg/ml
IL-4 pg/ml
IL-5 pg/ml
IL-6 pg/ml
IL-7 pg/ml
IL-8 pg/ml
IL-9 pg/ml
IL-10 pg/ml
IL-12(p70) pg/ml
IL-13 pg/ml
IL-15 pg/ml
IL-17 pg/ml
Eotaxin pg/ml
FGF-basic pg/ml
G-CSF pg/ml
GM-CSF pg/ml
IFN-γ pg/ml
IP-10 pg/ml
T0
T4M
HTP-CT arm

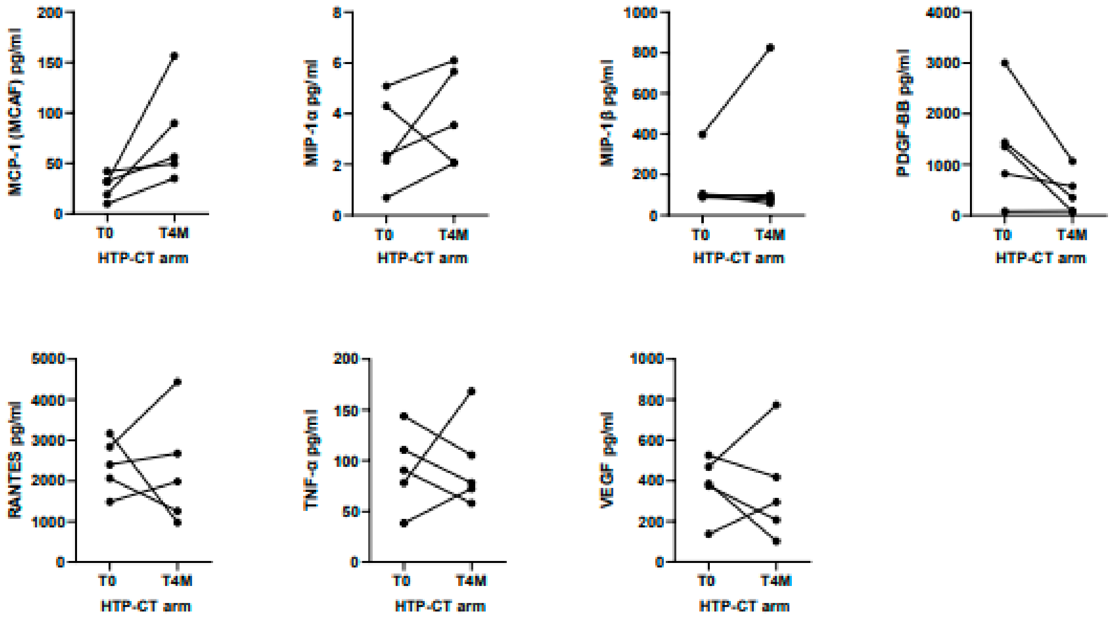

Footnote: Te: T effector; Th1: T helper 1; Th2: T helper 2; Th17: T helper 17; Th1/17: T helper 1/17; Tfh: T follicular helper; Tfh1: T follicular helper 1; Tfh2: T follicular helper 2; Tfh17: T follicular helper 17; Tfh1/17: T follicular helper 1/17; Treg: T regulatory; UM: unswitched memory; SM: switched memory; ASM: activated switched memory; DN: double negative; DN1: double negative 1; DN2: double negative 2; DN3: double negative 3; DN4: double negative 4; AcN: activated naïve; AnN: anergic naïve; RN: resting naïve; Ab: antibody secreting; T1/2: transitional 1/2; T3a: transitional 3a; MZP: marginal zone peripheral; Breg: B regulatory; AbS: antibody secreting; Imm: immature; Mon: monocytes.

Figure S15. Correlation studies between circulating immune cells and cytokines significantly different in patients with pancreatic adenocarcinoma (PDAC) treated with HybridTherm Probe ablation plus chemotherapy (HTP-CT arm) from those treated with chemotherapy only (CT arm) at 4-month follow-up, and the tumor volume (A) and serum levels of tumor marker CA19-9 (B). Correlation studies were performed using the Spearman's rho rank correlation test.

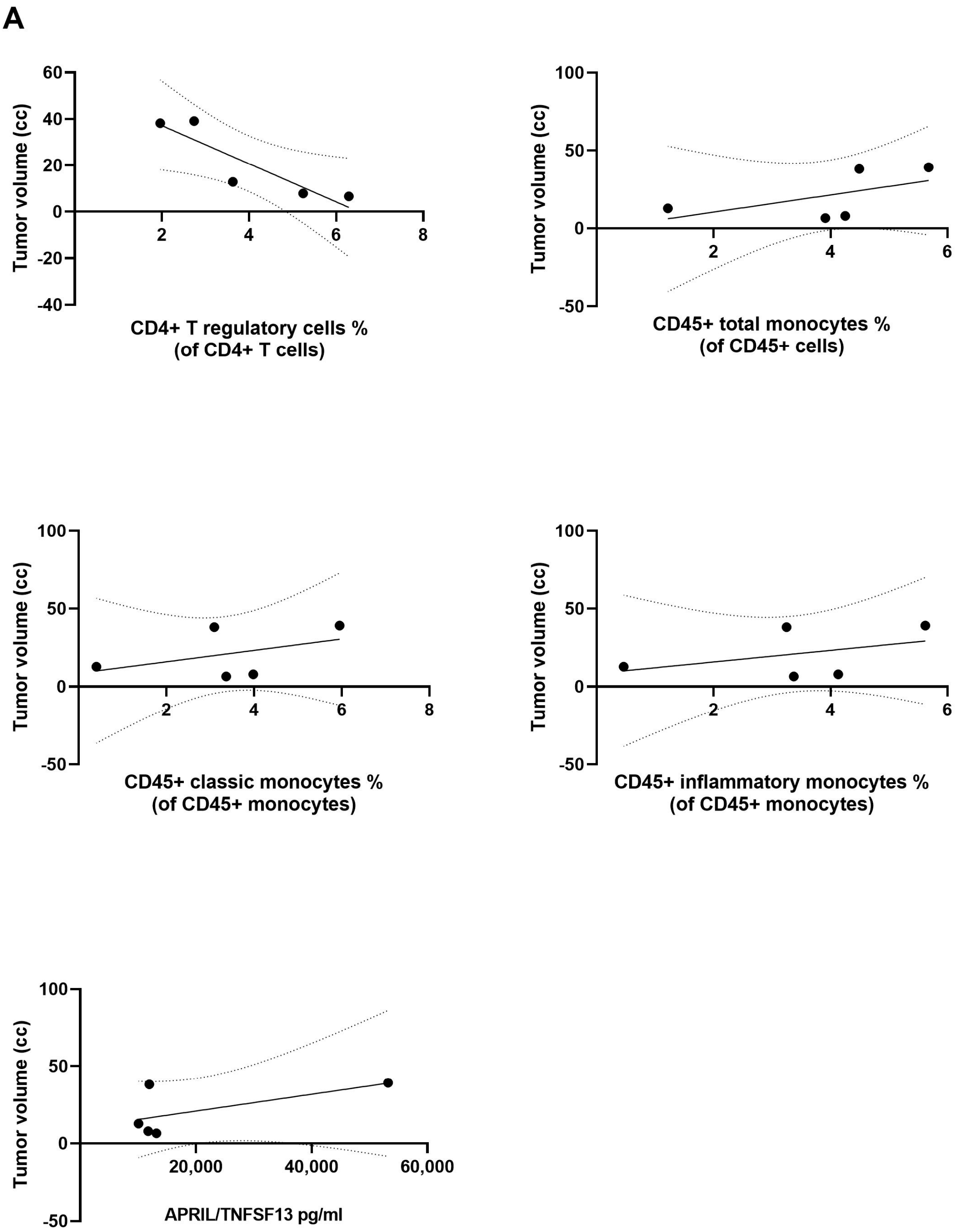

B

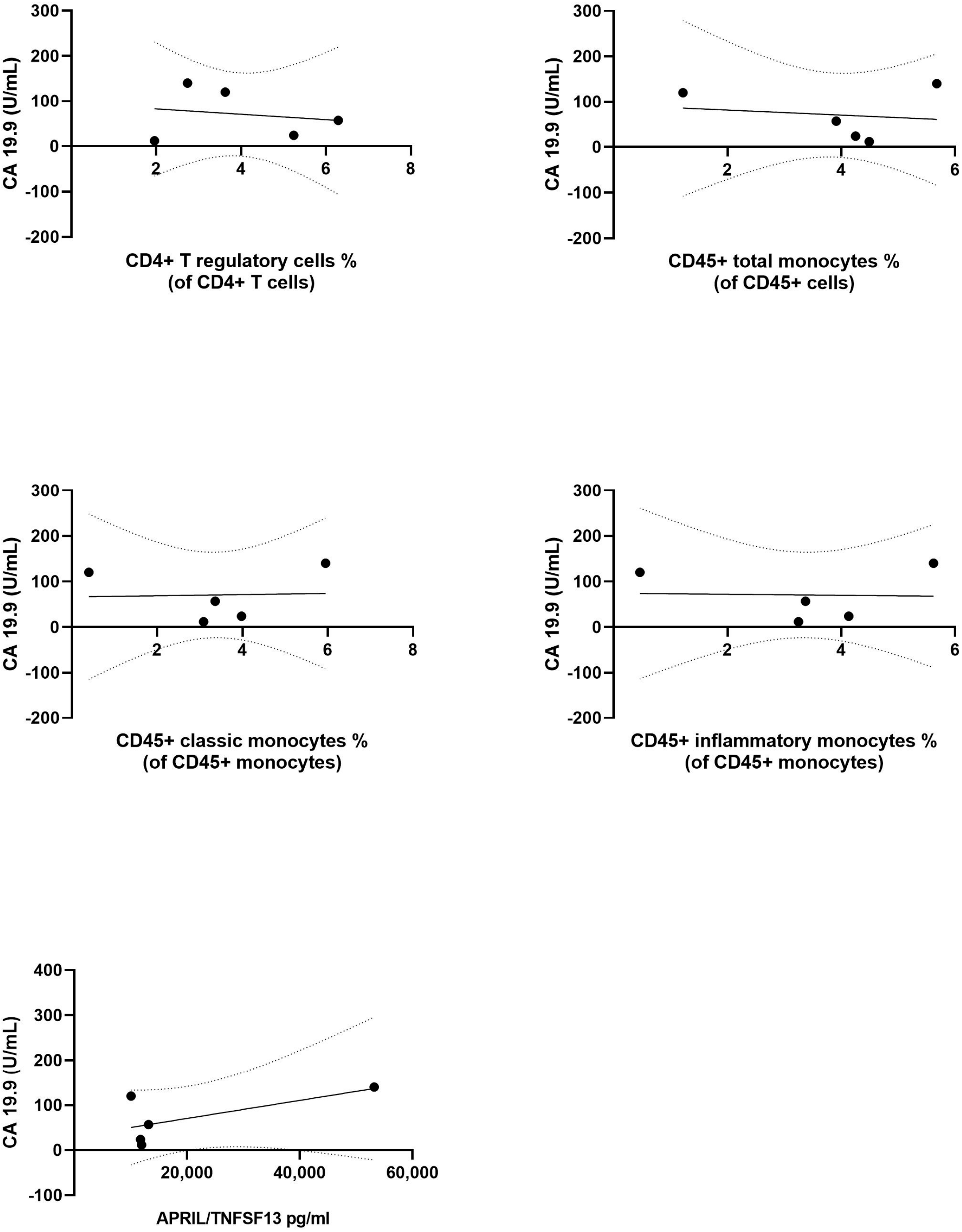
CA 19.9 (U/mL)
CD4+ T regulatory cells %
(of CD4+ T cells)
CA 19.9 (U/mL)
CD45+ total monocytes %
(of CD45+ cells)
CA 19.9 (U/mL)
CD45+ classic monocytes %
(of CD45+ monocytes)
CA 19.9 (U/mL)
CD45+ inflammatory monocytes %
(of CD45+ monocytes)
CA 19.9 (U/mL)
APRIL/TNFSF13 pg/ml
